# Inhibiting eukaryotic ribosome biogenesis

**DOI:** 10.1186/s12915-019-0664-2

**Published:** 2019-06-10

**Authors:** Dominik Awad, Michael Prattes, Lisa Kofler, Ingrid Rössler, Mathias Loibl, Melanie Pertl, Gertrude Zisser, Heimo Wolinski, Brigitte Pertschy, Helmut Bergler

**Affiliations:** 1 Institute of Molecular Biosciences, University of Graz, Humboldtstrasse 50/EG, A-8010 Graz, Austria; 2 Present address: Department of Cancer Systems Imaging, The University of Texas MD Anderson Cancer Center, Houston, TX USA

## Abstract

**Background:**

Ribosome biogenesis is a central process in every growing cell. In eukaryotes, it requires more than 250 non-ribosomal assembly factors, most of which are essential. Despite this large repertoire of potential targets, only very few chemical inhibitors of ribosome biogenesis are known so far. Such inhibitors are valuable tools to study this highly dynamic process and elucidate mechanistic details of individual maturation steps. Moreover, ribosome biogenesis is of particular importance for fast proliferating cells, suggesting its inhibition could be a valid strategy for treatment of tumors or infections.

**Results:**

We systematically screened ~ 1000 substances for inhibitory effects on ribosome biogenesis using a microscopy-based screen scoring ribosomal subunit export defects. We identified 128 compounds inhibiting maturation of either the small or the large ribosomal subunit or both. Northern blot analysis demonstrates that these inhibitors cause a broad spectrum of different rRNA processing defects.

**Conclusions:**

Our findings show that the individual inhibitors affect a wide range of different maturation steps within the ribosome biogenesis pathway. Our results provide for the first time a comprehensive set of inhibitors to study ribosome biogenesis by chemical inhibition of individual maturation steps and establish the process as promising druggable pathway for chemical intervention.

**Electronic supplementary material:**

The online version of this article (10.1186/s12915-019-0664-2) contains supplementary material, which is available to authorized users.

## Background

Ribosomes are essential nano-machines responsible for the synthesis of proteins. They are composed of a large and a small subunit, both containing ribosomal RNAs (rRNAs) and numerous ribosomal proteins. In eukaryotes, the formation of ribosomes is a complex, multi-compartmental process requiring a multitude of non-ribosomal assembly factors. Ribosome biogenesis is highly conserved among eukaryotes and best studied in the yeast *Saccharomyces cerevisiae* (reviewed in [1–4]). The initial steps of ribosome biogenesis take place in the nucleolus, a sub-compartment of the nucleus, in which the rRNA precursors are transcribed and loaded with assembly factors and ribosomal proteins. The small 5S rRNA of the large 60S subunit is transcribed separately by RNA polymerase III, while the 18S rRNA, constituent of the small 40S subunit, and the 25S and 5.8S rRNAs of the large subunit are transcribed together by RNA polymerase I in a polycistronic 35S transcript. This long pre-rRNA is co-transcriptionally recognized by a plethora of small subunit assembly factors forming a large 90S ribosomal precursor also termed the small subunit (SSU) processome ([5, 6] reviewed in [7]). After stepwise truncation at the 5′-end by endonucleases, cleavage at site A_2_ leads to 20S and 27SA_2_ pre-rRNAs, thereby separating small and large subunit assembly into independent pathways. The resulting pre-40S particles containing the 20S pre-rRNA are quickly exported into the cytoplasm, where the final maturation steps are accomplished by an endonucleolytic cleavage step at the 3′-end of the 18S rRNA (for a recent review of 40S assembly, see [8]). The process of pre-60S maturation is more complex, involving stepwise endo- and exonucleolytic 5′-end truncations of the 27SA_2_ pre-rRNA into the 27SA_3_ and the 27SB pre-rRNA (for a recent review of 60S assembly, see [9]). The 27SB pre-rRNA is subsequently split by endonucleolytic cleavage into a 5.8S precursor (7S pre-rRNA) and a 25S precursor (25.5S pre-rRNA). In the course of these maturation steps, pre-60S particles transit from the nucleolus to the nucleoplasm. While the mature 25S rRNA is finalized by 5′-3′ exonucleases in the nucleoplasm, processing of the 7S to the 5.8S rRNA occurs in several 3′-5′ exonucleolytic steps, first in the nucleoplasm and then, after nuclear export of the pre-60S particles, in the cytoplasm (see Additional file 1: Figure S1 for a schematic depiction of rRNA processing and [10, 11] for comprehensive reviews). Transport of export competent particles of both subunits through the nuclear pore complex depends on the Ran-GTP-dependent exportin Crm1 (XpoI) as well as specialized export factors like Mex67/Mtr2 or Arx1 [12, 13].

Along with the rRNA processing steps, pre-ribosomal particles undergo massive structural re-arrangements, as impressively evidenced by several recently published high-resolution cryo-electron microscopic structures of 90S, pre-60S, and pre-40S particles representing different maturation stages [14–21].

All these maturation steps are performed by more than 250, mostly essential assembly factors representing a wide variety of functions. Considering the number of involved factors, as well as the expenses for rRNA transcription and ribosomal protein synthesis, ribosome biogenesis represents a major activity in each cell [22]. Therefore, it is of particular importance for fast dividing cells and tightly linked to cell division and cell cycle progression. All these facts make ribosome biogenesis an exceptionally promising target for chemotherapeutical intervention during infectious with eukaryotic pathogens or neoplastic diseases [23–26]. Indeed, several established chemotherapeutic drugs were shown to also inhibit ribosome biogenesis [24], suggesting that this pathway is either their primary target or a second site target whose inhibition enhances the potential of chemotherapeutical agents. In both scenarios, inhibition of ribosome biogenesis can contribute to the anti-proliferative effect of chemotherapy.

While several inhibitors of rRNA transcription have been reported [23, 26], only very few inhibitors are known that directly target the ribosome biogenesis pathway downstream of transcription. We previously discovered that the drug diazaborine specifically inhibits large ribosomal subunit formation by preventing the cytoplasmic release of the shuttling pre-60S assembly factor Rlp24 by the AAA-ATPase Drg1 [27–29]. This release reaction is a prerequisite for all downstream maturation steps. Consequently, diazaborine treatment prevents the release and the recycling of all known shuttling pre-60S assembly factors. This results in depletion of shuttling pre-60S assembly factors in the nucleus, hence causing defects in early pre-60S maturation. Recently, another ribosome biogenesis inhibitor (ribozinoindole) was described, which targets the nuclear AAA-ATPase Mdn1 in *Schizosaccharomyces pombe* [30]. The homologous protein in *S. cerevisiae*, Rea1, is required for pre-60S release of the assembly factor Rsa4 and might play a role in a major structural transition of the pre-60S particle during nucleoplasmic maturation steps [15, 31].

In order to explore further promising druggable steps of ribosome biogenesis, we developed a microscopy-based screening approach to identify novel ribosome biogenesis inhibitors. In this study, we systematically screened ~ 1000 low molecular weight substances for inhibitory effects on the ribosome biogenesis pathway in *S. cerevisiae*. One hundred twenty-eight of these compounds led to a nuclear accumulation of fluorescently labeled reporter proteins for either the 40S, the 60S, or both ribosomal subunits. Northern blot analyses revealed that the individual substances affect various stages of pre-rRNA processing, suggesting a broad coverage of different targets along the pathway. By introducing a versatile set of novel ribosome biogenesis inhibitors, our results provide a promising starting point to study this essential pathway in depth by chemical inhibition of individual maturation steps. Moreover, our results provide a basis to establish the ribosome biogenesis pathway as target for chemotherapy.

## Results

### Screen setup

**Fig. 1:**
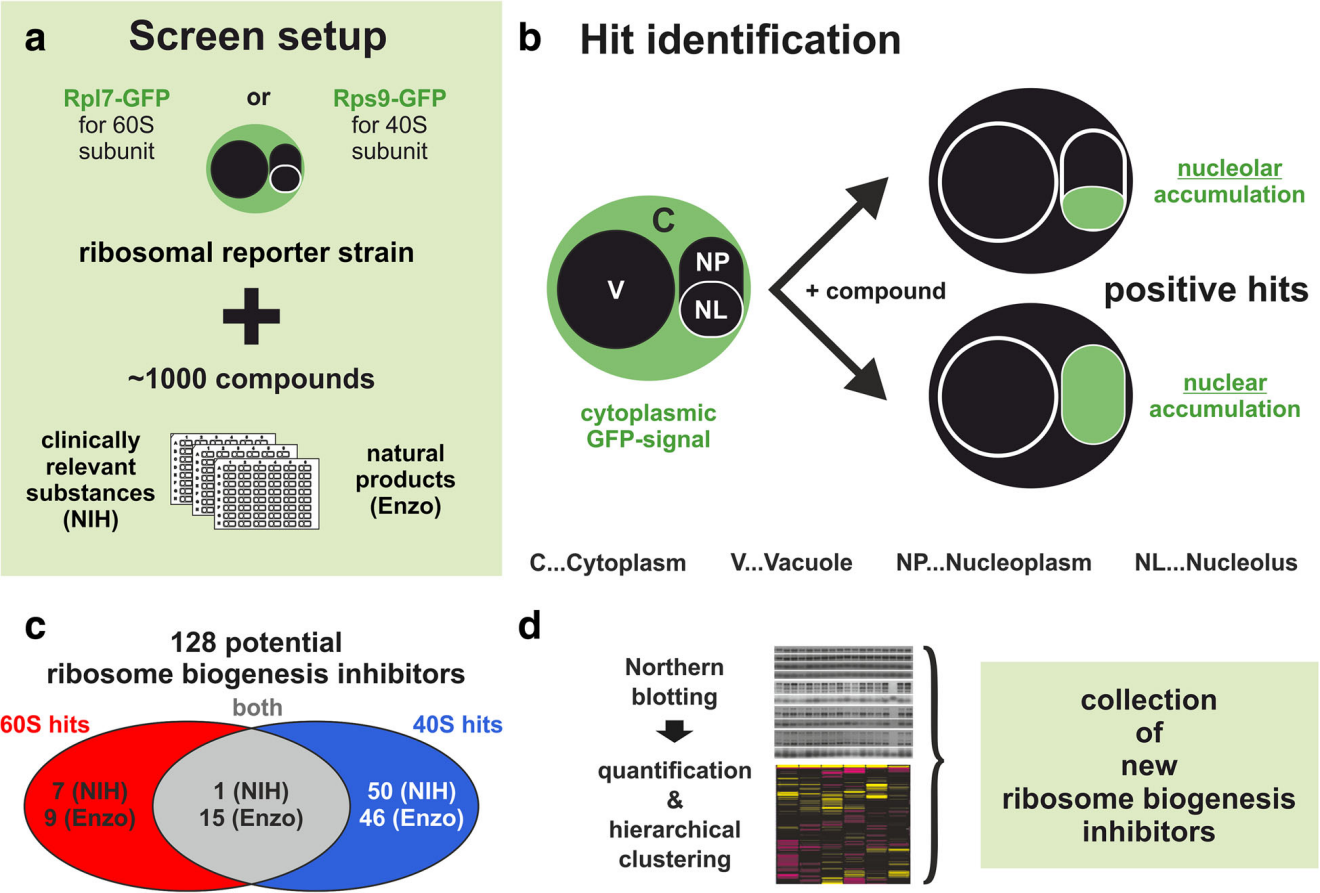
Screen setup to identify novel ribosome biogenesis inhibitors. **a** Reporter proteins for either the 40S (Rps9a) or the 60S (Rpl7a) ribosomal subunit were C-terminally fused to GFP. The resulting reporter strains were separately tested with ~ 1000 substances from two compound libraries (NIH clinical collection (Additional file 2: Table S1) and Screen-Well Natural Product Library Version 7.4 (Enzo Life sciences) (Additional file 2: Table S2)). **b** After treatment, cells were inspected by fluorescence microscopy. Ribosome biogenesis defects were identified by a shift of the steady-state GFP signal of the reporters from the cytoplasm into the nucleolus (NL) and/or the nucleoplasm (NP). **c** A total pool of 128 positively scoring inhibitors comprised 16 inhibitors specific for the 60S subunit, 96 specific for the 40S subunit and 16 affecting both subunits. **d** Positively scoring hits were further characterized by northern blot analysis of pre-rRNAs and subsequent hierarchical clustering based on quantification of processing intermediates

In order to identify novel inhibitors of the ribosome synthesis pathway, we designed a strategy that allowed us to systematically screen a large quantity of low molecular weight substances for effects on ribosome biogenesis in the yeast *S. cerevisiae* (summarized in Fig. 1). In total, we tested ~ 1000 substances from two different compound libraries. The “NIH clinical collection” comprises 446 small molecules that have already been used in human clinical trials (Additional file 2: Table S1). The “Screen-Well Natural Product Library Version 7.4” from Enzo Life Sciences provides 502 small molecules of natural origin (Additional file 2: Table S2). The rationale behind the screen was based on the observation that inhibition of the ribosome biogenesis pathway frequently causes ribosomal subunit export defects [32]. To score for such export defects, yeast strains expressing C-terminal GFP-tag fusions of a ribosomal protein of either the 60S or the 40S subunit were generated by chromosomal integration at the genomic loci. For the 60S subunit export screen, we used the large ribosomal subunit protein Rpl7 as reporter (uL30 according to a recently proposed new nomenclature [33]). Rpl7 is incorporated into pre-60S particles at an early maturation stage allowing to score for very early defects in 60S synthesis [34–39]. As 40S subunit reporter, we selected Rps9 (uS4), an early assembling 40S subunit ribosomal protein [16, 20, 40–42]. As both proteins have two paralogs in yeast, we chose the more abundant variants Rpl7a and Rps9a for GFP-tag fusions.

**Fig. 2:**
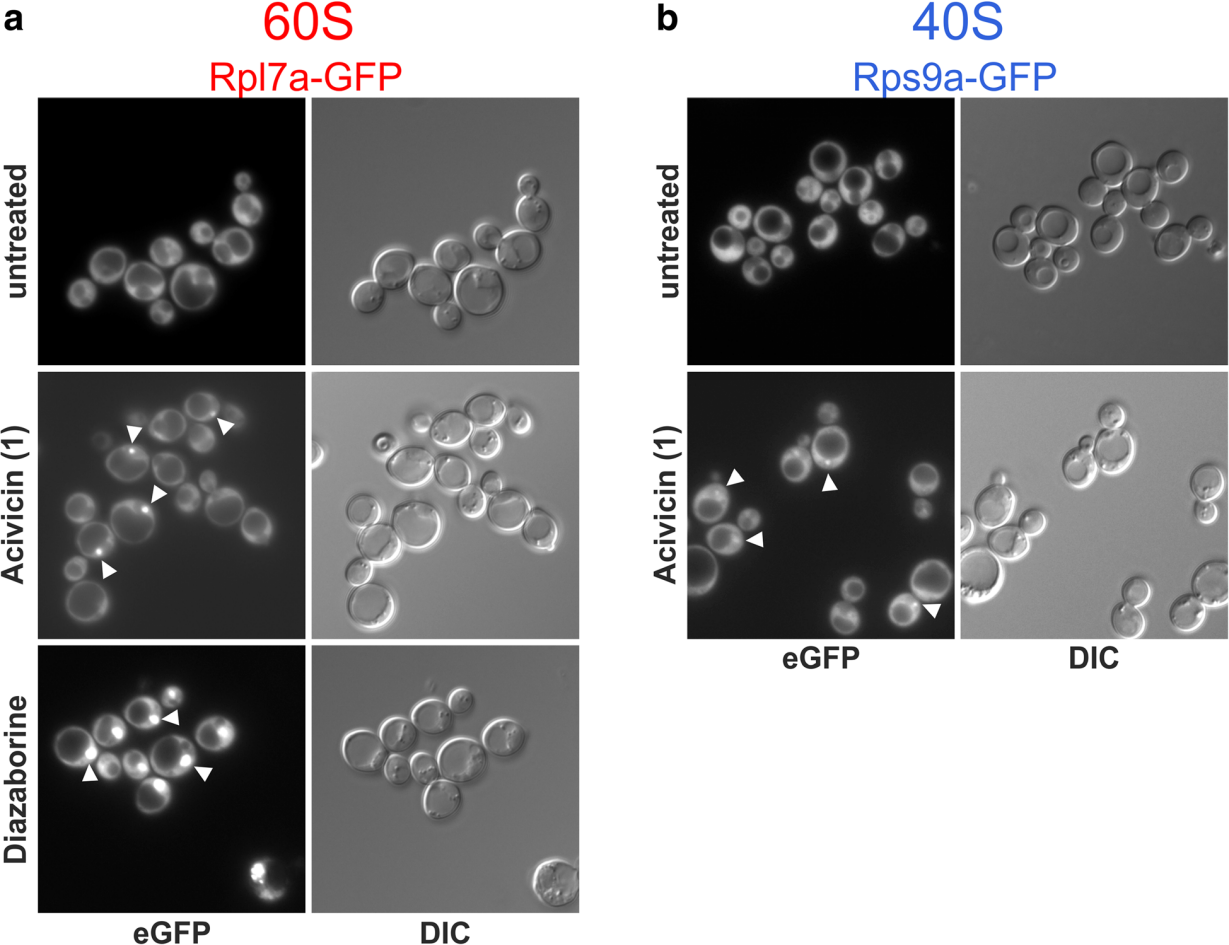
Controls for nucle(ol)ar accumulation of GFP-tagged ribosomal reporter proteins upon inhibitor treatment. Numbers in brackets denote the unique identifier of the compounds listed in Additional file 3: Table S3. **a** 60S subunit reporter Rpl7a-GFP. In the untreated cells, Rpl7a-GFP was exclusively localized in the cytoplasm, as is typical for ribosomal proteins due to the high concentration of mature ribosomes in the cytoplasm. Acivicin was discovered in the course of the 60S screen and causes nucleolar accumulation (indicated by white arrowheads) of the 60S reporter, suggesting a very early block in ribosome biogenesis. Treatment with diazaborine specifically blocks 60S maturation, resulting in nuclear accumulation of Rpl7a-GFP. **b** 40S subunit reporter Rps9a-GFP. While Rps9a-GFP was found exclusively in the cytoplasm in the untreated cells, it accumulated in the nucleolus upon treatment with acivicin (indicated by white arrowheads)

As a reference for inhibition of 60S subunit export, we treated cells with diazaborine, which causes nuclear accumulation of Rpl7a-GFP due to ribosome biogenesis inhibition ( [28], Fig. 2a). As no inhibitor of the 40S biogenesis pathway was available, we aimed at establishing a positive control causing nuclear accumulation of pre-40S subunits. In the course of the screen with the 60S subunit reporter Rpl7a-GFP, we found that treatment with acivicin resulted in the accumulation of Rpl7a-GFP in small dots in the nucleus which likely correspond to the nucleolus or a sub-fraction thereof (Fig. 2a). This defect is indicative of a very early blockage of ribosome biogenesis.

Since the initial steps of large and small subunit formation are interconnected, we reasoned that early ribosome biogenesis inhibition should affect both the 40S and the 60S biogenesis pathway. Indeed, acivicin treatment also caused nuclear accumulation of our 40S reporter Rps9a-GFP in a dotted structure (Fig. 2b). This demonstrates that the Rps9a-GFP reporter is suitable to detect inhibitor-induced ribosome biogenesis defects and that acivicin can be used as a reference for the 40S screen. DMSO alone, the solvent of the tested substances, caused no nuclear accumulation of the ribosomal reporter proteins in tested concentrations up to 6% (data not shown).

### Nucle(ol)ar accumulation of ribosomal subunit reporters identifies 128 potential ribosome biogenesis inhibitors

**Fig. 3:**
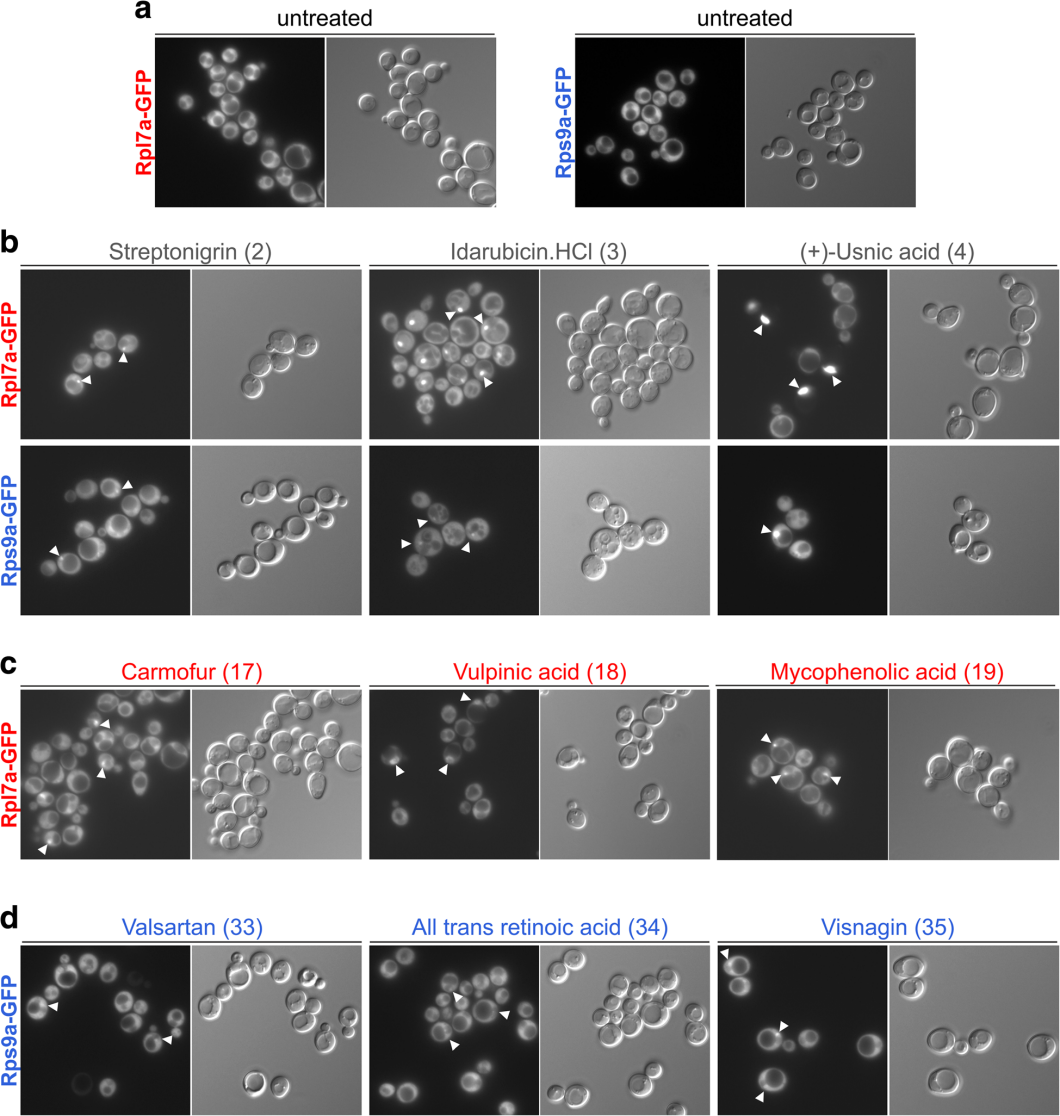
Examples of hits in the GFP-reporter screen. eGFP pictures are shown on the left, DIC pictures on the right. Inhibitor-induced signal accumulation is highlighted by white arrowheads pointing towards the sites of accumulation. Numbers in brackets denote the unique identifier of the identified hits listed in Additional file 3: Table S3. **a** Untreated cells show cytoplasmic localization of both GFP-tagged reporter proteins. **b** Examples of inhibitors (streptonigrin, idarubicin HCl, and (+)-usnic acid) inducing nuclear accumulation in both reporter strains (Rpl7a-GFP and Rps9a-GFP, substance names in grey letters). **c** Examples of inhibitors (carmofur, vulpinic acid, and mycophenolic acid) causing nuclear accumulation only of the 60S reporter Rpl7a-GFP (red letters). **d** Examples of inhibitors (valsartan, all-trans retinoic acid, and visnagin) causing nuclear accumulation only of the 40S reporter Rps9a-GFP (blue letters)

In the initial large-scale screen, the Rpl7a-GFP and Rps9a-GFP reporter strains were incubated with each of the 948 inhibitors for at least 3 h. Subsequently, the treated cells were inspected by fluorescence microscopy. All substances causing nucle(ol)ar accumulation of one or both reporter constructs were re-analyzed in two additional screening rounds. In total, 128 substances were confirmed as positive hits (Fig. 3 and Additional file 1: Figures S2-S8; see Additional file 3: Table S3 for a complete list of identified substances including references for documented activities against cancer cells [43–113]).

The observed phenotypes included accumulation in the entire nucleolus or smaller dotted structures within the nucleolus (Additional file 1: Figure S9). One of the tested substances from the NIH collection and 15 substances from the Enzo collection caused accumulation of both reporters in the nucleus, suggesting a very early block in ribosome biogenesis (Fig. 3b and Additional file 1: Figure S2). Seven of the NIH compounds and nine of the Enzo compounds caused nuclear accumulation of only the 60S subunit reporter Rpl7a-GFP (Fig. 3c and Additional file 1: Figure S3), whereas 50 of the NIH and 46 of the Enzo substances caused nuclear accumulation of the 40S subunit reporter Rps9a-GFP (Fig. 3d and Additional file 1: Figures S4-S8).

To conclude, our microscopy-based screen successfully identified 128 potential ribosome biogenesis inhibitors targeting either the maturation of the small 40S subunit, the large 60S subunit, or both.

### Inhibitors induce diverse pre-rRNA processing defects

**Fig. 4:**
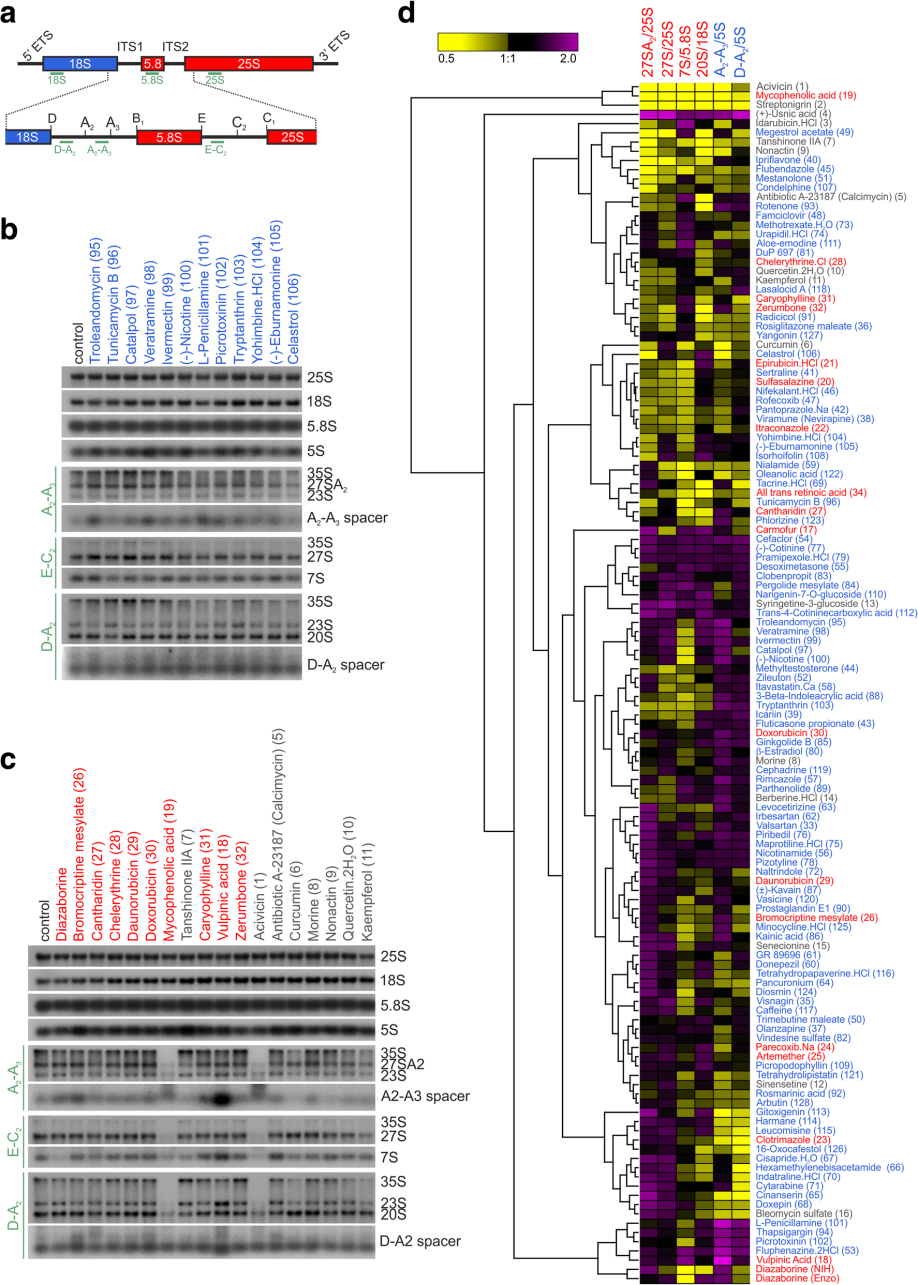
Inhibitors induce different rRNA processing defects. **a** Schematic picture of the longest rRNA precursor (35S pre-rRNA) containing the sequences of the mature 18S rRNA, the 5.8S rRNA and the 25S rRNA, which are interrupted and flanked by internal (ITS) and external (ETS) transcribed spacers, respectively. The region encompassing ITS1 and ITS2 is enlarged and the main processing sites (A_2_, A_3_, B_1_, C_1_, C_2_, D, and E) are indicated. Hybridization sites of probes used in the northern blotting experiment are indicated by green bars. The entire processing pathway is displayed in Additional file 1: Figure S1. **b**, **c** Examples of northern blots after treatment with substances found in the 40S reporter screen (**b**, blue lettering), in the 60S reporter screen (**c**, red lettering) or in both screens (**c**, grey lettering). The detected rRNA species are indicated on the right side, the probes used to detect the respective pre-rRNAs are indicated on the left side. The northern blots for all 128 compounds are shown in Additional file 1: Figures S10 and S11. **d** Hierarchical clustering of the indicated pre-rRNA/rRNA ratios. The color code in the heatmap indicates increased (purple) or decreased (yellow) levels of the respective precursors normalized to the mock control (DMSO) and then referenced to the respective mature rRNA in the same sample. Inhibitors found in the 60S reporter screen are marked by red lettering, inhibitors from the 40S screen are written in blue and inhibitors identified in both screens in grey. The control diazaborine was included once with the same DMSO concentration used in the screen with the NIH substances and once with the DMSO concentration used in the Enzo screen. Both conditions were found in the same cluster, demonstrating neglectable effects of the different DMSO concentrations

In order to further validate that the identified substances induce specific ribosome biogenesis defects and to elucidate the approximate stages of inhibition, we investigated their effect on pre-rRNA processing (Fig. 4). For this purpose, wild-type yeast cells were treated with each of the 128 identified substances for 30 min. Subsequently, total RNA was isolated and subjected to northern blotting using probes specifically hybridizing to either mature 25S, 18S, 5.8S, and 5S rRNAs or pre-rRNA spacer elements (Fig. 4a and Additional file 1: Figure S1). With this set of probes, we were able to monitor a variety of pre-rRNA precursors including (1) 35S pre-rRNA, detectable by all probes; (2) 27SA_2_ pre-rRNA; (3) total 27S pre-rRNA (including 27SA_2_, 27SA_3_, and 27SB forms); (4) 7S pre-rRNA; and (5) 20S pre-rRNA. Additionally, two of the probes also detected the 23S RNA, which is generated by aberrant cleavage at site A_3_ upon a delay of the early A_0_, A_1_, and A_2_ processing steps (Additional file 1: Figure S1 [114–116]). Additionally, two spacer fragments arising from endonucleolytic cleavages of 27SA_2_ and 20S pre-rRNAs, the A_2_-A_3_ spacer and the D-A_2_ spacer, respectively, were detected in our analyses. As several substances caused alterations in the levels of these two spacer fragments, we also included them in our analyses.

Altogether, these experiments revealed a number of different pre-rRNA processing defects caused by the tested inhibitors. Examples of blots are shown in Fig. 4b (40S hits) and c (60S hits), while the northern blot analyses for all 128 substances are displayed in Additional file 1: Figures S10 and S11. Signals detected in the northern blots were densitometrically quantified and pre-rRNA signals were normalized to the mock control (DMSO). Due to the high stability of mature ribosomes, mature rRNA levels are expected to be largely unaltered after the short treatment period of 30 min, allowing referencing of each precursor to the respective mature rRNA. Therefore, the calculated values represent ratios of pre-rRNA precursors relative to the respective mature rRNAs in each sample and are listed in Additional file 4: Table S4. Spacer fragments were referred to the 5S rRNA due to the similar size. Subsequently, we performed hierarchical clustering of the signal ratios (Fig. 4d). 35S and 23S pre-rRNAs were excluded from these analyses due to high variation in the quantifications caused by low levels in most samples and therefore low signal to noise ratios. The clustering analysis highlights that the tested inhibitors induced many different processing defects, leading either to accumulation or reduction of precursors. All substances causing accumulation or reduction of at least one pre-rRNA species by a factor of at least 1.5 are listed in Table 1.

**Table 1:** Inhibitors causing the strongest pre-rRNA processing phenotypes and the most affected intermediate (for a complete list of all changing pre-rRNAs, see Additional file 3: Table S3)

Strongest pre-rRNA phenotype (±>1.5x)	Microscopy screen		
	60S hit	40S + 60S hit	40S hit
All precursors gone	Mycophenolic acid (19)	Acivicin (1), streptonigrin (2)	
27SA_2_ ↓*		Tanshinone IIA (7)	Flubendazole (45)
27SA_2_ ↑	Carmofur (17)		Valsartan (33), levocetirizine (63), cinanserin (65), doxepin (68), cytarabine (71), trans-4-cotininecarboxylic acid (112), gitoxigenin (113)
27S↓		Acivicin (1)	Ipriflavone (40)
27S ↑		(+)-Usnic acid (4), syringetine-3-glucoside (13)	
7S↓			Tunicamycin B (96), catalpol (97)
7S ↑		Idarubicin HCl (3), cefaclor (54), desoximetasone (55)	Fluphenazine 2HCl (53)**, pergolide mesylate (84)
20S ↓		Antibiotic A-23187 (calcimycin) (5), nonactin (9)	All trans retinoic acid (34), megestrol acetate (49), rotenone (93)
A_2_-A_3_ sp. ↓		Curcumin (6)	
A_2_-A_3_ sp. ↑	Vulpinic acid (18)		Fluphenazine 2HCl (53)**, rimcazole (57), thapsigargin (94), troleandomycin (95), veratramine (98), (−)-nicotine (100), L-penicillamine (101), picrotoxinin (102),
D-A_2_ ↑			Icariin (39)
Additional strong pre-rRNA changes (manually curated)			
35S ↑	Parecoxib sodium (24), zerumbone (32)	Tanshinone IIA (7), morine (8), nonactin (9), senecionine (15), bleomycin sulfate (16)	Visnagin (35), zileuton (52), hexamethylenebisacetamide (66), indatraline HCl (70), cytarabine (71), naltrindole (72), uradipil HCl (74), DuP 697 (81), vindesine sulfate (82), clobenpropit (83), pergolide mesylate (84), catalpol (97), veratramine (98), ivermectin (99), (−)-nicotine (100), tryptanthrin (103), celastrol (106), isorhoifolin (108), narigenin-7-O-glucoside (110), leucomisine (115), tetrahydropapaverine HCl (116), tetrahydrolipistatin (121), phlorizine (123), diosmin (124),
Aberrant 23S **↑**	Carmofur (17), vulpinic acid (18)	Tanshinone IIA (7), berberine HCl (14)	Yangonin (127)

* ↑ denotes accumulation, ↓ denotes reduction of the respective precursor

**Listed twice due to equally strong effects

(Substance identifier no., compare Additional file 3: Table S3)

**Fig. 5:**
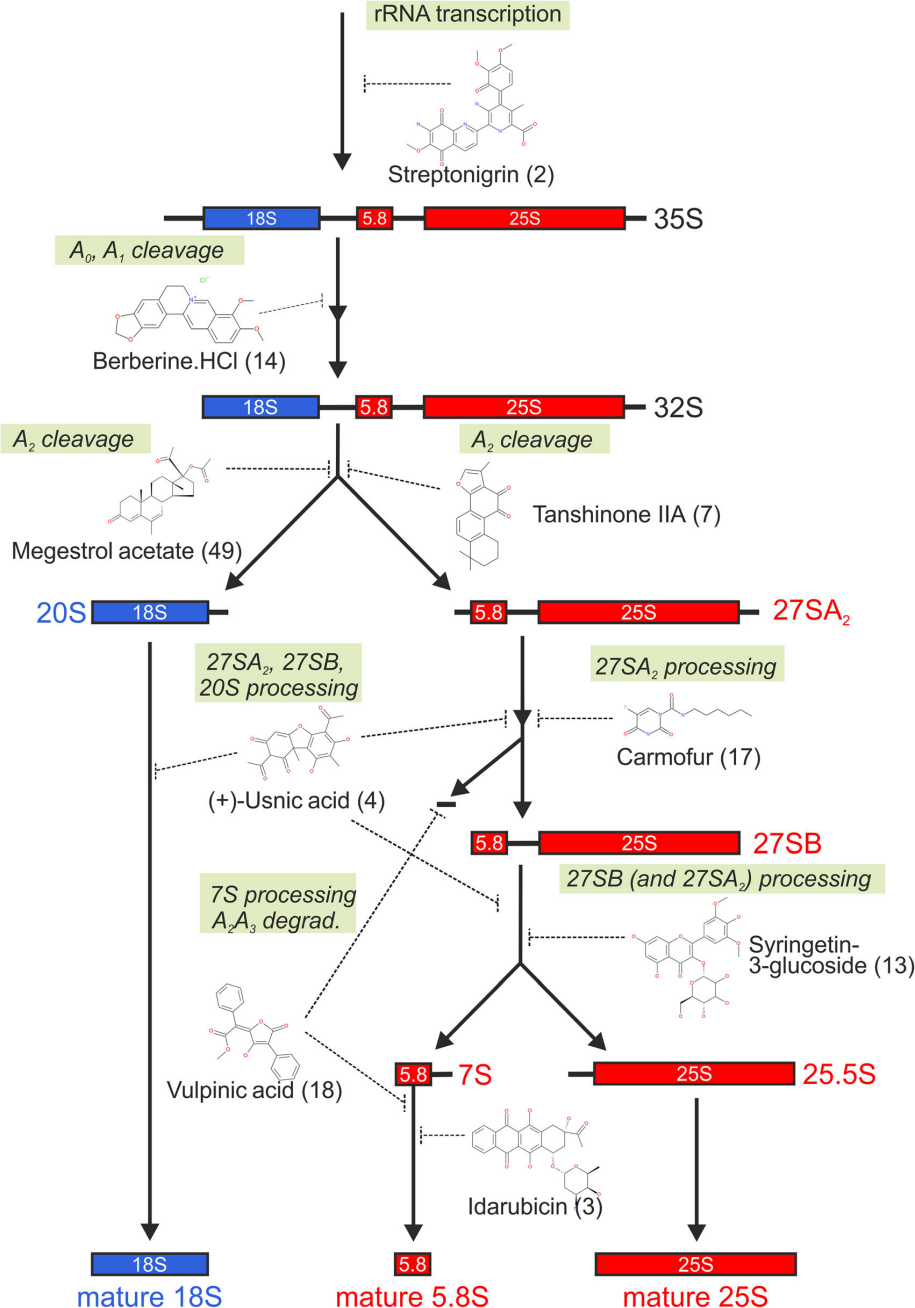
Identified inhibitors target different stages of rRNA processing. A simplified rRNA processing scheme showing processing from 35S pre-rRNA to the mature rRNAs (18S, 5.8S, and 25S) was complemented with examples of inhibitors and their potentially targeted ribosomal maturation steps. The predicted target steps were derived from the altered rRNA processing pattern in the northern blot analysis (Fig. 4, Table 1, and Additional file 1: Figures S10 and S11)

Although 35S and 23S signals were not considered for quantification and clustering analysis, substances leading to clear 35S or 23S accumulation were manually selected and are included in Table 1. For each of the different observed phenotypes, the respective inhibitor causing the strongest effect is additionally highlighted within the processing pathway in Fig. 5, emphasizing the good coverage of maturation steps targeted by the inhibitors.

### Inhibitors target a broad spectrum of maturation steps along the ribosome biogenesis pathway

The wide range of different rRNA processing defects indicates that the set of novel inhibitors covers large parts of the ribosome biogenesis pathway ranging from the transcriptional level to very early nucleolar steps to late subunit-specific steps in the nucleoplasm. Three substances, acivicin, mycophenolic acid, and streptonigrin, caused a drastic reduction of all pre-rRNAs (Fig. 4, Table 1). Since all rRNA precursors are affected, we conclude that these substances cause a general block in rRNA transcription rather than targeting processing of specific precursors. In contrast to a general blockage of transcription, all other substances led to a reduction of few or only one rRNA precursor(s). This reduction of specific precursors may either be an indication for a blockage at an upstream maturation step or for instability of an intermediate, leading to its degradation. Tanshinone IIA, for instance, mainly led to a reduction of 27SA_2_ pre-rRNA; as however, also 20S pre-rRNA levels were reduced, a maturation step upstream of the generation of these two precursors, for example A_2_ cleavage or earlier steps, might be affected. Likewise, megestrol acetate and also most of the other substances mainly leading to 20S reduction also showed reduced 27S levels to some extent, suggesting that they might also exert their main effects before separation of the 40S and 60S maturation pathways. Interestingly, multiple inhibitors, including berberine HCl, resulted in clear accumulation of the 23S rRNA, indicative of delays in A_0_, A_1_, and A_2_ processing. Only few of the tested substances caused significant 20S pre-rRNA accumulation ((+)-usnic acid, celastrol, epirubicin HCl, narigenin-7-O-glucoside, isorhoifolin, parecoxib Na, artemether, picropodophyllin, carmofur, trans-4-cotininecarboxylic acid). This is not surprising considering the facts that pre-40S particles contain only few assembly factors and moreover are exported rapidly after A_2_ cleavage, both minimizing the pool of potential targets to be identified by nuclear accumulation of a 40S subunit reporter.

In addition to substances interfering with very early processing events, several substances clearly affected later, 60S subunit-specific stages, deducible from their characteristic rRNA processing defect. Several of these compounds caused 27SA_2_ accumulation, indicative of an early 60S-specific processing defect, with the 5-FU derivative carmofur showing the strongest phenotype. Notably, we found that yeast cells, in which genes encoding components of the exosome (i.e., Rrp6, Rrp43 and Mtr4), a multi-subunit complex involved in 7S pre-rRNA processing, had been deleted, were hypersensitive to carmofur (Additional file 1: Figure S12). Moreover, previous experiments demonstrated that deletion of exosome components caused hypersensitivity to 5-FU [117, 118]. Together, these results strengthen the hypothesis that carmofur and 5-FU directly target the ribosome biogenesis pathway.

Another substance, syringetine-3-glucoside, showed the strongest accumulation of total 27S signal, although also 27SA_2_ accumulated, suggesting a delay in maturation of 27SA_2_ as well as 27SB precursors. Alternatively, the inhibitor-induced 27SB accumulation might cause a secondary block in processing of the earlier 27SA_2_.

Even later maturation steps were affected by vulpinic acid. This lichen secondary product led to accumulation of 7S pre-rRNA, but also of the aberrant 23S RNA, as well as to an accumulation of the small A_2_-A_3_ spacer fragment. A similar albeit weaker effect (accumulation of 7S, 23S, and A_2_-A_3_ spacer) was also observed with fluphenazine 2HCl. This slowdown in earlier processing steps (A_0_, A_1_, and A_2_) upon 7S accumulation is another demonstration that inhibition of late steps can rebound on earlier events [29].

A late pre-60S maturation defect was also observed for idarubicin, which caused the accumulation of 7S pre-rRNA, suggesting it inhibits nucleoplasmic 60S maturation steps. Idarubicin belongs to the compound group of rubicins, of which three additional members (daunorubicin, doxorubicin, and epirubicin) showed up in our screen. Since doxorubicin was reported previously to block rRNA transcription in human cells [24, 119] we tested two rubicins for effects on pre-ribosome maturation in mammalian cell culture. Indeed, doxorubicin and epirubicin lead to changed nucleolar morphology and nucleoplasmic accumulation of an Rpl27-GFP reporter construct in HeLa cells (Additional file 1: Figure S13).

In summary, the wide range of rRNA processing defects observed upon inhibitor treatment suggests that large parts of the ribosome maturation pathway can be targeted by low molecular weight inhibitors.

## Discussion

In this study, we performed a microscopy-based screen to identify a large set of novel inhibitors of the ribosome biogenesis pathway. In total, 128 substances caused accumulation of pre-ribosomal particles in the nucleus. Three of these substances interfered with rRNA transcription: streptonigrin, acivicin, and mycophenolic acid led to an almost complete disappearance of pre-rRNAs after 30 min of treatment (Fig. 4 and Additional file 1: Figure S11). Intriguingly, the accumulation of the reporter constructs in the absence of any detectable pre-rRNA may be an indication for a nucleolar deposition/retention system for ribosomal proteins. Acivicin and mycophenolic acid are known to inhibit purine and/or pyrimidine synthesis. Their effect on rRNA transcription can be explained by the fact that rRNA synthesis is presumably by far the biggest nucleotide consumer in growing cells. Consistent with this suggestion, the effects of these compounds on transcription are not specific for RNA polymerase I, since the *NRD1* mRNA in comparison to the long-lived *ACT1* mRNA showed a decrease after 30 min of treatment (Additional file 1: Figure S11). This indicates that also transcription by RNA polymerase II is affected. Streptonigrin acts differently and is known to complex with DNA and thereby affects not only transcription, but also replication [120].

Our main interest was however the identification of inhibitors targeting maturation steps downstream of transcription. Indeed, all other substances causing phenotypes in the northern blot analysis (Fig. 4 and Table 1) inhibited rRNA maturation and not transcription. This analysis shows that our inhibitors cover many different maturation steps along the ribosome biogenesis pathway.

It is noteworthy that many substances caused very early defects, obvious from accumulation of the GFP reporters, and in particular Rps9a-GFP, in small dots in the nucleolus (Additional file 1: Figure S9). The most likely explanation is that the majority of known ribosome assembly factors and therefore also the highest number of potential targets for inhibition participate in very early, nucleolar maturation steps. This suggests that many of the identified substances interfere with steps of ribosome biogenesis preceding A_2_ cleavage and/or incorporation of Rpl7. This would explain why only 33 compounds were identified to cause nuclear accumulation of the large subunit reporter Rpl7a-GFP, while 110 substances scored positive in the screen using the small subunit reporter Rps9a-GFP.

The validity of our results is confirmed by inhibitors identified in our screen which have already been linked to ribosome biogenesis. One prime example is the pyrimidine analogue carmofur, a derivative of 5-fluorouracil containing an additional carbamoyl moiety that allows oral administration of the drug [62, 117, 118]. This modification makes the spectrum of targets even broader since carmofur was shown to be also effective in 5-FU-resistant cells [121–124]. Both 5-FU and carmofur are widely used as chemotherapeutic agents despite the fact that their manifold effects on the cell are not fully understood. 5-FU is incorporated into RNA and interferes with multiple nucleotide-related pathways including rRNA transcription and processing [117, 125–128]. Based on their various effects on RNA metabolism, it was anticipated that these pyrimidine analogues might also affect the processing of pre-rRNAs [24, 117, 129]. Indeed, several studies suggested a link between the action of pyrimidine analogues and components of the exosome, which catalyzes the 3′-5′ trimming of the 7S pre-rRNA [117, 118, 126, 127, 130]. In line with these suggestions, we observed super-sensitivity of exosome mutants also to carmofur and detected increased levels of 7S pre-rRNA in our study, even though the most prominent effect of the drug was an accumulation of 27SA_2_ pre-rRNA (Fig. 4d). Notably, also the known exosome target *NRD1* mRNA [131] accumulated after carmofur treatment, further supporting a direct action of the compound on the exosome (Additional file 1: Figure S10). Interestingly, cantharidin also caused a strong accumulation of the *NRD1* mRNA. Treatment of mammalian cells with cantharidin was recently shown to result in overexpression of several components of the 3′-5′ decay pathway, including two core components of the exosome [132], which may be an indication for a connection of the drug target to the 3′-5′ RNA decay machinery.

Another interesting example for a substance group whose members scored as hits in our screen is the rubicins. These compounds are thought to block DNA replication by intercalating into the DNA or inhibiting topoisomerase II [133–136] and are widely used for clinical treatment of solid tumors. In addition to the reported effect of doxorubicin on pre-rRNA transcription in yeast and human fibrosarcoma cells [24, 119], we found that rubicins interfere with pre-ribosome maturation and export in HeLa cells. In our screen, the individual rubicins caused different patterns of pre-rRNA processing defects in yeast and hence seem to affect different maturation steps in ribosome biogenesis. However, it has to be noted that these differences may also be the result of different susceptibility of yeast to the individual rubicins. While a unified concentration and treatment period was necessary for this large-scale study, the MICs, as well as the optimal concentrations and treatment periods with the inhibitors, will have to be determined in detail in future studies.

Another compound previously linked to ribosome biogenesis is the lichen secondary metabolite vulpinic acid. Haplo-insufficiency profiling experiments previously showed that among other strains, the heterozygous deletion of the pre-60S assembly factor *YTM1* resulted in increased vulpinic acid sensitivity [137]. This result further supports that vulpinic acid targets ribosome biogenesis and suggests that the substance acts in close functional proximity to Ytm1. In our experiment, vulpinic acid treatment led to accumulation of the 7S and 23S pre-rRNAs, as well as a striking accumulation of the A_2_-A_3_ spacer fragment known to be degraded mainly by the 5′-3′ exonuclease Rat1 [138]. However, since the level of the A_0_-A_1_ spacer, which is also a target of Rat1, was unaffected by Vulpinic acid (data not shown), a direct inhibition of Rat1 is unlikely. Moreover, conditional *ytm1* mutants, *YTM1* depletion, or over-expression of a dominant negative *YTM1* allele showed different pre-rRNA processing patterns and mainly caused 27SB pre-rRNA accumulation [139, 140]. Therefore it is also unlikely that Ytm1 is the direct target of the inhibitor. Further studies will be necessary to reveal the precise target of vulpinic acid in ribosome biogenesis.

All these examples of compounds that were reported independently from our study to affect ribosome biogenesis validate the results of our screen. However, the vast majority of the small molecules identified in this screen were for the first time recognized as inhibitors of the ribosome biogenesis pathway. This demonstrates that our screening method can successfully be used to mine a complex pathway such as ribosome formation for potent and specific inhibitors. Since the identified inhibitors cause a broad range of pre-rRNA processing changes, they cover many different steps of the ribosomal maturation cascade. Therefore our study provides a comprehensive toolbox of novel inhibitors which allows to investigate the highly dynamic process of ribosome synthesis.

Ribosome biogenesis is tightly interwoven with numerous other pathways. This is supported by our observation that several inhibitors of other cellular processes also scored positive in our screen for ribosome biogenesis inhibitors, potentially revealing up to now unrecognized regulatory cross-talks. Tunicamycin B, for example, activates the unfolded protein response pathway and was additionally shown to downregulate transcription of ribosomal protein genes [141–143]. Similarly, methotrexate blocks dihydrofolate reductase [144], thereby affecting nucleotide synthesis, transcription of rRNA [24], and likely also formation of *S*-adenosylmethionine [145, 146] which is required for methylation of rRNA.

Although the identification of the precise targets of the here identified ribosome biogenesis inhibitors remains a task for future studies, selective inhibitors will become valuable tools to facilitate the exploration of hitherto not well-understood steps of the pathway. The high number of potential drug targets will also open up novel avenues for chemotherapy.

## Conclusions

The results from our screen provide for the first time a broad set of inhibitors targeting various steps of ribosome biogenesis. These compounds will not only prove valuable to investigate this highly interesting pathway but also to identify novel drug targets. Remarkably, many of the identified substances were previously shown to interfere with growth of tumor cells, are currently being investigated in clinical trials, or have already been used for clinical cancer treatment [24, 26] (see also Additional file 3: Table S3). This finding underlines the crucial importance of ribosome biogenesis for fast proliferating cells including tumor cells and eukaryotic pathogens. This suggests that targeting ribosome biogenesis might be an efficient strategy for treatment of infectious diseases or malignant tumors.

## Methods

### Yeast strains

In order to prevent interference of the Ade-pigment in the W303 *ade2* strain with fluorescence microscopy, wild-type *ADE2* was integrated into the strain by homologous recombination, resulting in the white W303 derivative C303. This strain was also used for northern blot analyses. To generate the small and the large subunit export reporter strains, the chromosomal copies of *RPL7a* and *RPS9a* were C-terminally fused to GFP by homologous recombination using a *HIS3*MX6 selection marker. The integration cassettes were generated by PCR using the pFA6a-*HIS3*MX6 plasmid [147] as template and 55 bp of gene-specific sequences as primer overhangs. For co-localization experiments, selected nuclear compartment markers (Nic96, Nop58, Hho1) were C-terminally fused to 3x-mCherry in the GFP-reporter strains, also done by homologous recombination. Plasmid pFA6a-3mcherry-hphNT1 served as template for the generation of the recombination cassette. The genotypes of all strains are listed in Additional file 5: Table S5 [148].

### Compound libraries

The NIH clinical collection, provided by the National Institute of Health (NIH), USA, included 446 clinically relevant compounds in a concentration of 10 mM dissolved in DMSO. The Screen-Well Natural Product Library Version 7.4 from Enzo Life Sciences included 502 individual purified compounds in DMSO with a final concentration of 2 mg/ml. Full lists of all tested inhibitors are provided in Additional file 2: Tables S1 and S2.

### Microscopy-based screening

Strains C303a Rpl7a-GFP and C303a Rps9a-GFP were grown in 96-well deep-well plates in synthetic dextrose medium lacking histidine (SD-his) at 28 °C to an OD_600_ of 0.4 (early log-phase). Subsequently, inhibitors were added at a final concentration of 50 μM and cells were further incubated for at least 3 h. Diazaborine was used as a control with a final concentration of 18 μM. DMSO alone, the solvent of the tested substances, caused no nuclear accumulation of the ribosomal reporter proteins in tested concentrations up to 6%. Consequently, untreated cells without DMSO served as negative control. Subsequently, fluorescence microscopy was performed using a Zeiss Axioskop Microscope with a narrow band enhanced GFP filter from Zeiss. At least four representative pictures were recorded for each substance, and pictures were independently evaluated by two researchers for nucle(ol)ar accumulation of the reporter proteins. All substances were screened twice, and compounds leading to nuclear accumulation of a reporter protein were confirmed in a third round of analysis.

For co-localization microscopy experiments, inhibitor-treated cells of strains additionally expressing 3x-mCherry tagged nuclear compartment marker proteins were investigated using a Leica DM6 B Microscope equipped with a × 100/1.4 Plan APO objective and narrow band GFP or RHOD ET filters. For imaging, the high-resolution DFC9000GT camera and the LASX premium software were used.

### RNA isolation and northern blotting

C303a cells were grown in SD medium at 30 °C to an OD_600_ of ~ 0.7 (log-phase). The inhibitors were added at a final concentration of 50 μM to 2 ml of culture each. Addition of the NIH inhibitors led to a final DMSO concentration of 0.5% in the culture. The Enzo inhibitors, provided by the company in 2 mg/ml concentration, were adjusted with DMSO to 50 μM resulting in final DMSO concentrations of ~ 2% in the culture. Separate negative controls were grown for the NIH and Enzo substances with 0.5% and 2% DMSO respectively. Additionally, diazaborine was used as a positive control for screens with both libraries and was added with the corresponding amounts of DMSO.

After inhibitor treatment for 30 min, cells were harvested and suspended in 200 μl lysis buffer (10 mM Tris-HCl pH 7.5, 10 mM EDTA, 0.5% SDS). After addition of 200 μl glass beads (0.5 mm diameter), cells were mechanically disrupted by vigorous shaking for 3 min. RNA was extracted from the lysates by phenol-chloroform-isoamylalcohol (25:24:1; three times), followed by chloroform-isoamylalcohol (24:1) extraction and ethanol precipitation. Three micrograms of RNA per sample was separated on 1.5% MOPS-agarose gels. The RNA was transferred overnight onto a Hybond-N nylon membrane (Amersham Biosciences) and then UV cross-linked to the membrane. Except for the E/C2, anti-*ACT1*, and anti-*NRD1* probes (37 °C), hybridization was performed overnight at 42 °C in 500 mM NaPO_4_ buffer, pH 7.2, 7% SDS, 1 mM EDTA using 5′-^32^P-labeled oligonucleotide probes with the following sequences: 18S rRNA: CATGGCTTAATCTTTGAGAC, 25S rRNA: CTCCGCTTATTGATATGC, 5.8S rRNA, GCGTTCTTCATCGATGC, 5S rRNA: GGTCACCCACTACACTACTCGG, A2-A3: TGTTACCTCTGGGCCC, E-C2: GGCCAGCAATTTCAAGTTA, D-A2: GACTCTCCATCTCTTGTCTTCTTG, anti-*ACT1*: CCGGCAGATTCCAAACCCAAAACAGAAGGATGGA, anti-*NRD1*: GCTCATCGGGGTATAAGTGGTGATTGTTTGTGC [131]. The membranes were washed three times for 20 min at 42 °C in 40 mM NaPO_4_ buffer, pH 7.2, 1% SDS. Membranes were regenerated by washing in 1% SDS. Each sample was analyzed two times with independent gels and hybridizations.

Signals were detected by autoradiography and quantified using the ImageLab 5.2 software (Biorad). Quantified signals were normalized using the signals of the mock control (DMSO), which was loaded at least once per 20 treated samples. Ratios of precursors to mature rRNAs were calculated. 27S pre-rRNAs were referenced to mature 25S rRNA levels, 20S pre-rRNA to mature 18S rRNA, and 7S pre-rRNA to mature 5.8S rRNA. The spacer fragments were referenced to the 5S pre-rRNA due to the similar size. Means of the two ratios were calculated from the two northern blot rounds (values from the two individual rounds and mean values are listed in Additional file 4: Table S4). The mean values were then transformed into logarithmic values (basis 2) and loaded into the Genesis software provided by the Institute for Genomics and Bioinformatics, Graz University of Technology [149]. The data were subjected to hierarchical clustering using the average linkage agglomeration rule.

### Fluorescence microscopy of HeLa^Rpl7-GFP^ cells

HeLa^Rpl27-GFP^ cells [150] stably expressing the ribosomal reporter protein Rpl27-GFP were cultured in Gibco FluoroBrite™ DMEM medium supplemented with 10% Fetal bovine serum and GlutaMax (all Thermo Scientific) for 24 h before treatment with 1 μM of the indicated compounds for 5 h and inspection using a Leica SP5 confocal microscope and a HCX PL APO × 25 objective.

## Additional files

Additional file 1: **Figure S1.** Yeast rRNA processing pathway. **Figure S2.** Inhibitors causing nuclear accumulation of both the Rpl7a-GFP (60S) and the Rps9a-GFP (40S) reporter. Related to Fig. 3. **Figure S3.** Inhibitors causing nuclear accumulation of the Rpl7a-GFP (60S) reporter. Related to Fig. 3. **Figure S4-S8.** Inhibitors causing nuclear accumulation of the Rps9a-GFP (40S) reporter. Related to Fig. 3. **Figure S9.** Different classes of localization phenotypes upon inhibitor treatment. **Figure S10.** rRNA processing phenotypes caused by the inhibitors from the NIH inhibitor collection. Related to Fig. 4. **Figure S11.** rRNA processing phenotypes caused by the inhibitors from the Enzo inhibitor collection. Related to Fig. 4. **Figure S12.** Deletion of exosome factors cause hypersensitivity to Carmofur. **Figure S13.** Treatment with doxorubicin and epirubicin causes nucleoplasmic accumulation of an Rpl27-GFP reporter and nucleolar fragmentation in HeLa cells. (DOCX 20739 kb)

Additional file 2: **Table S1.** Complete list of compounds contained in the “NIH clinical collection” as provided by the distributor. Table S2. Complete list of compounds contained in the “Enzo Natural Product Library” as provided by the distributor. (PDF 1086 kb)

Additional file 3: **Table S3.** All hits of the microscopy screen including a complete list of all changing pre-rRNAs (± > 1.5x) for substances listed in Table 1 and documented references to activities against cancer cells. (PDF 78 kb)

Additional file 4: **Table S4.** pre-rRNA precursor alterations after inhibitor treatment. Calculated ratios (pre-rRNA/mature rRNA) from quantifications of two northern blot experiments (round 1 and round 2 plus mean). The blots corresponding to round 1 are shown in Additional file 1: Figures S10 and S11. (XLSX 56 kb)

Additional file 5: **Table S5.** *Saccharomyces cerevisiae* strains used in this study. (PDF 244 kb)
