# Supplementary material for: Inhibiting eukaryotic ribosome biogenesis
Source: BMC Biol. 2019 Jun 10;17:46. doi: 10.1186/s12915-019-0664-2 (PMC6558755; doi:10.1186/s12915-019-0664-2)
Supplement: Supplementary file 1 — Figure S1. Yeast rRNA processing pathway. Figure S2. Inhibitors causing nuclear accumulation of both the Rpl7a-GFP (60S) and the Rps9a-GFP (40S) reporter. Related to Fig. 3. Figure S3. Inhibitors causing nuclear accumulation of the Rpl7a-GFP (60S) reporter. Related to Fig. 3. Figure S4-S8. Inhibitors causing nuclear accumulation of the Rps9a-GFP (40S) reporter. Related to Fig. 3. Figure S9. Different classes of localization phenotypes upon inhibitor treatment. Figure S10. rRNA processing phenotypes caused by the inhibitors from the NIH inhibitor collection. Related to Fig. 4. Figure S11. rRNA processing phenotypes caused by the inhibitors from the Enzo inhibitor collection. Related to Fig. 4. Figure S12. Deletion of exosome factors cause hypersensitivity to Carmofur. Figure S13. Treatment with doxorubicin and epirubicin causes nucleoplasmic accumulation of an Rpl27-GFP reporter and nucleolar fragmentation in HeLa cells. (DOCX 20739 kb) [file 12915_2019_664_MOESM1_ESM.docx]

**Additional file 1: Figures S1-S13**

**Inhibiting eukaryotic ribosome biogenesis**

**Dominik Awad^1,2^, Michael Prattes^1^, Lisa Kofler^1^, Ingrid Rössler^1^, Mathias Loibl^1^, Melanie Pertl^1^, Gertrude Zisser^1^, Heimo Wolinski^1^, Brigitte Pertschy^1*^ and Helmut Bergler^1*^**

***Corresponding authors**

**^1^ Institute of Molecular Biosciences, Humboldtstrasse 50/EG, University of Graz, A-8010 Graz, Austria**

**^2^ Present address: Department of Cancer Systems Imaging, The University of Texas MD Anderson Cancer Center, Houston, TX, USA**

***email:** [**brigitte.pertschy@uni-graz.at**](mailto:brigitte.pertschy@uni-graz.at) **(B.P.),** [**helmut.bergler@uni-graz.at**](mailto:helmut.bergler@uni-graz.at) **(H.B.)**


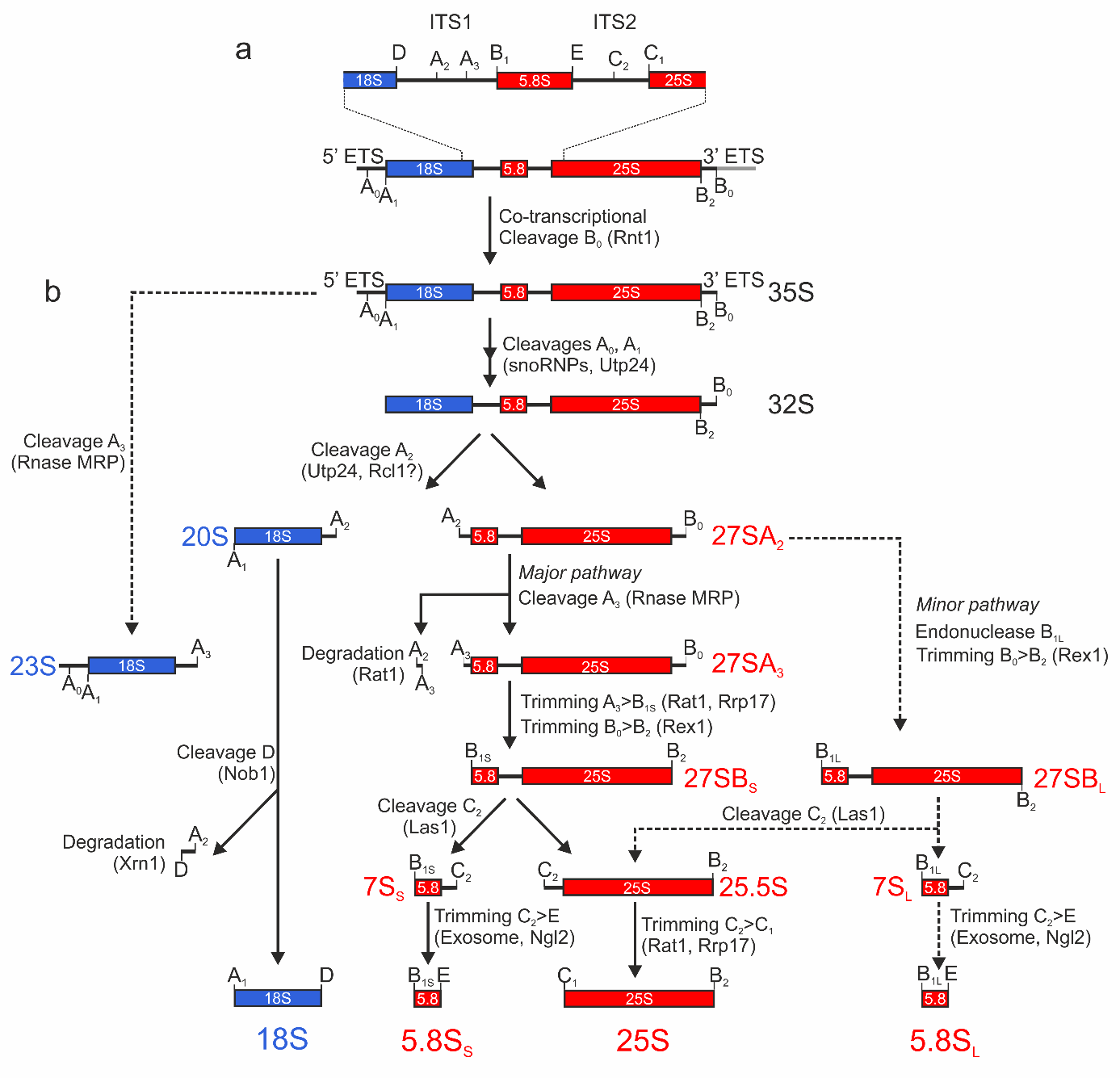


**Figure S1: Yeast rRNA processing pathway**. **(A) Schematic picture of one rDNA transcriptional unit.** 18S, 5.8S and 25S rRNA are transcribed together with external (ETS) and internal (ITS) transcribed spacers (black) by RNA polymerase I. A transcribed spacer, that is removed due to co-transcriptional cleavage at B_0_, releasing the 35S pre-rRNA, is indicated in grey. The 5S rRNA is transcribed in opposite direction by RNA polymerase III and is not depicted. **(B)** **rRNA processing scheme.** pre-rRNA intermediates and the two spacer fragments generated by endonucleolytic cleavage analyzed in this study (D/A_2_ and A_2_/A_3_) are indicated. The 35S pre-rRNA is released by co-transcriptional cleavage at B_0_ by the endonuclease Rnt1. Two consecutive endonucleolytic cleavages in the 5'-ETS, involving snoRNPs and Utp24 lead to generation of 32S pre-rRNA, sharing the 5'-end with the mature 18S rRNA. Subsequently, precursors for the 40S and 60S subunit are separated by endonucleolytic cleavage at processing site A_2_, which was suggested to be performed by either Utp24 or Rcl1. When early A_0_, A_1_ and A_2_ cleavages are blocked or delayed, 23S RNA can be generated by premature cleavage at site A_3_ (indicated on the left). A_0_, A_1_ and A_2_ cleavages can also occur co-transcriptionally, which is not depicted. A_2_ cleavage releases the 20S pre-rRNA, a 3'-extended 18S rRNA precursor, and the 27SA_2_ pre-rRNA containing the sequences for 5.8S and 25S rRNAs. 20S pre-rRNA containing 40S subunits are exported into the cytoplasm where the 3'-spacer fragment is removed by the endonuclease Nob1, resulting in the mature 18S rRNA. The cleaved off D-A_2_ spacer fragment is degraded by the cytoplasmic 5'-3' exonuclease Xrn1. 27SA_2_ pre-rRNA can further maturate in two alternative pathways. The major pathway involves cleavage at site A_3_ by the RNase MRP and subsequent 5'-3' processing mainly by exonucleases Rat1 and Rrp17, resulting in the more abundant 5.8S rRNA 5'-end at site B_1S_. In this pathway, an A_2_-A_3_ spacer fragment is generated that is subsequently degraded, mainly involving Rat1. The alternative 27S processing pathway is mediated by an unknown endonuclease and creates a slightly longer variant with the B_1S_ 5'-end of the 5.8S rRNA. Both resulting 27SB variants are then endonucleolytically cleaved at site C_2_ by Las1, resulting in the short 7SB_1S_ or the longer 7SB_1L_ variant of 7S and a 25.5S pre-rRNA. 5'-3' processing of the 25.5S pre-rRNA by Rat1 and Rrp17 results in the mature 25S rRNA. 3'-processing of both variants of 7S pre-rRNA occurs in several 3'-5' exonucleolytic processing steps mainly involving the nuclear exosome and, after nuclear export of pre-60S subunits, Ngl2. See also (10, 11) for comprehensive reviews on rRNA processing.

**
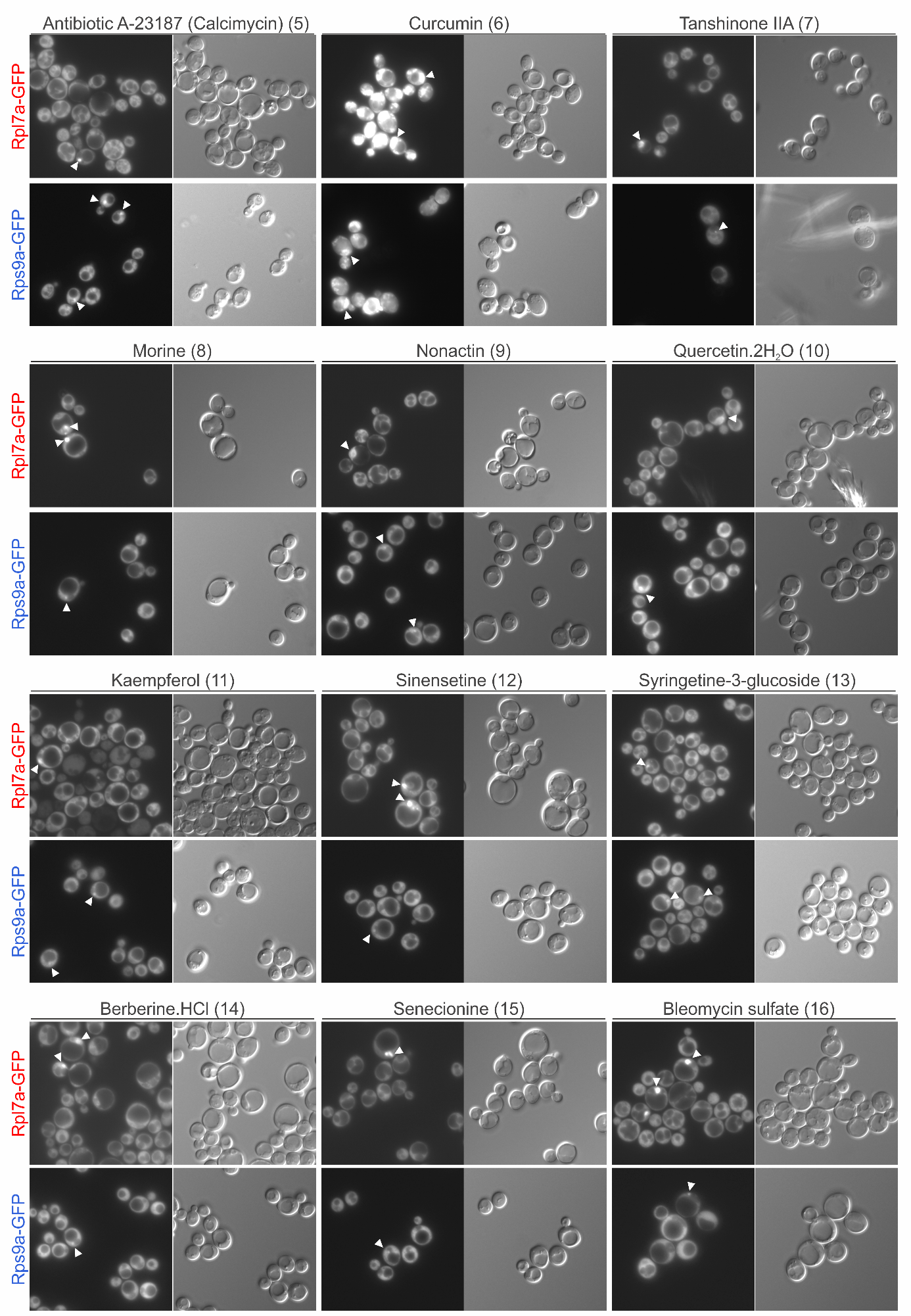
**

**Figure S2**: **Inhibitors causing nuclear accumulation of both the Rpl7a-GFP (60S) and the Rps9a-GFP (40S) reporter.** The eGFP pictures are shown on the left, the DIC pictures on the right, respectively. One representative picture is shown for each compound.


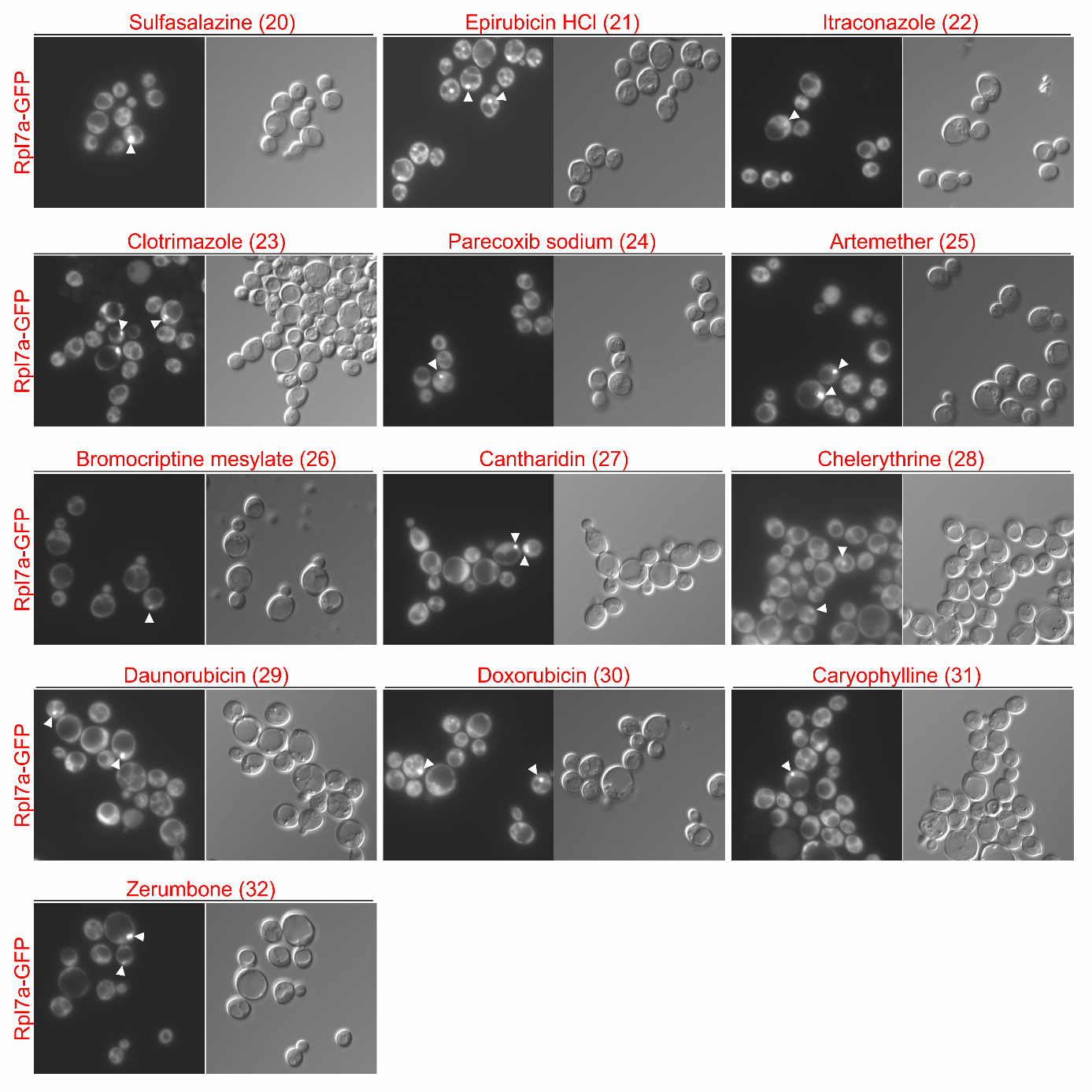


**Figure S3**: **Inhibitors causing nuclear accumulation of the Rpl7a-GFP (60S) reporter.** The eGFP pictures are shown on the left, the DIC pictures on the right, respectively. One representative picture is shown for each compound.


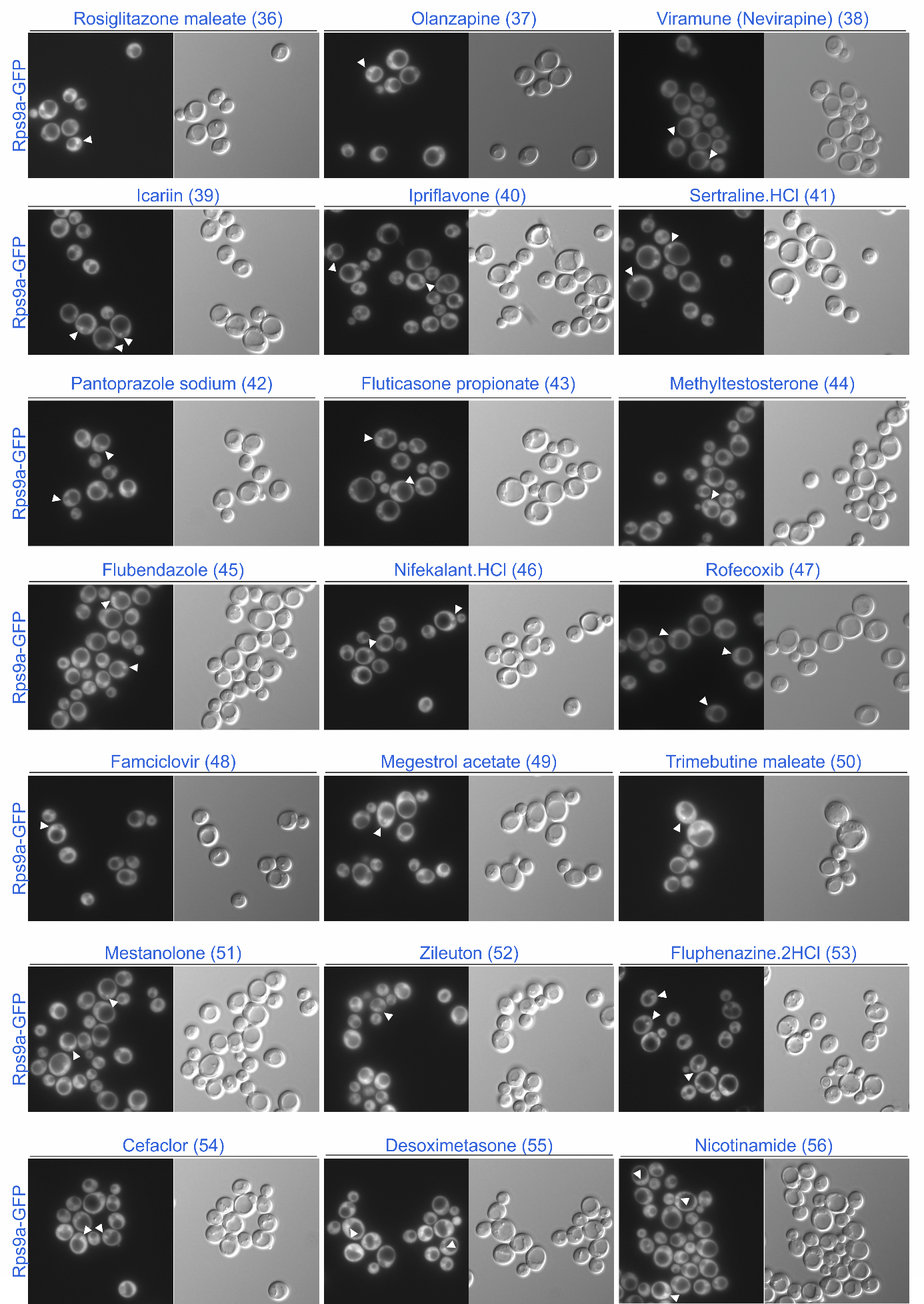


**Figure S4**: **Inhibitors causing nuclear accumulation of the Rps9a-GFP (40S) reporter.** The eGFP pictures are shown on the left, the DIC pictures on the right, respectively. One representative picture is shown for each compound.


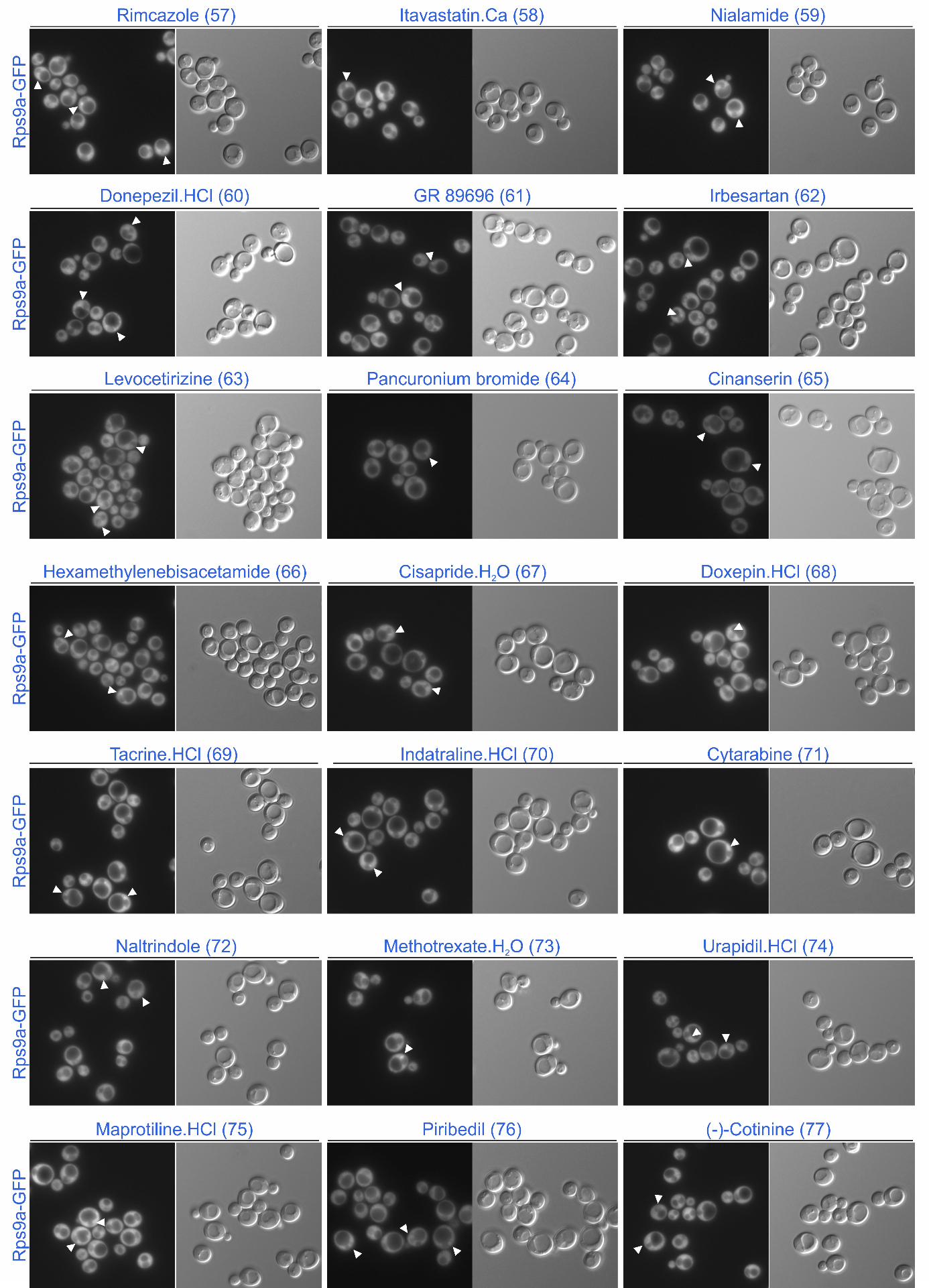


**Figure S5**: **Inhibitors causing nuclear accumulation of the Rps9a-GFP (40S) reporter (continued).** The eGFP pictures are shown on the left, the DIC pictures on the right, respectively. One representative picture is shown for each compound.


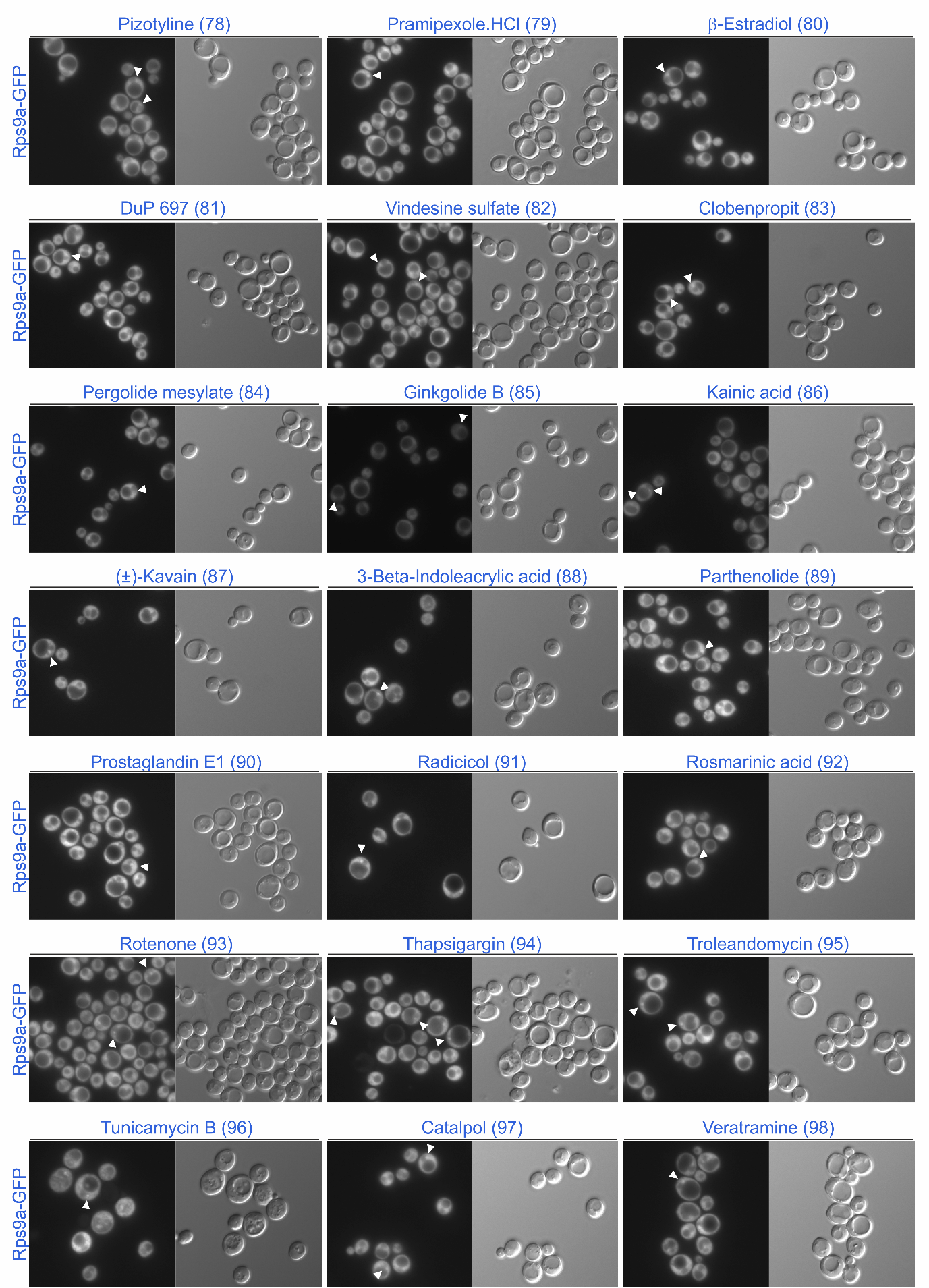


**Figure S6**: **Inhibitors causing nuclear accumulation of the Rps9a-GFP (40S) reporter (continued).** The eGFP pictures are shown on the left, the DIC pictures on the right, respectively. One representative picture is shown for each compound.


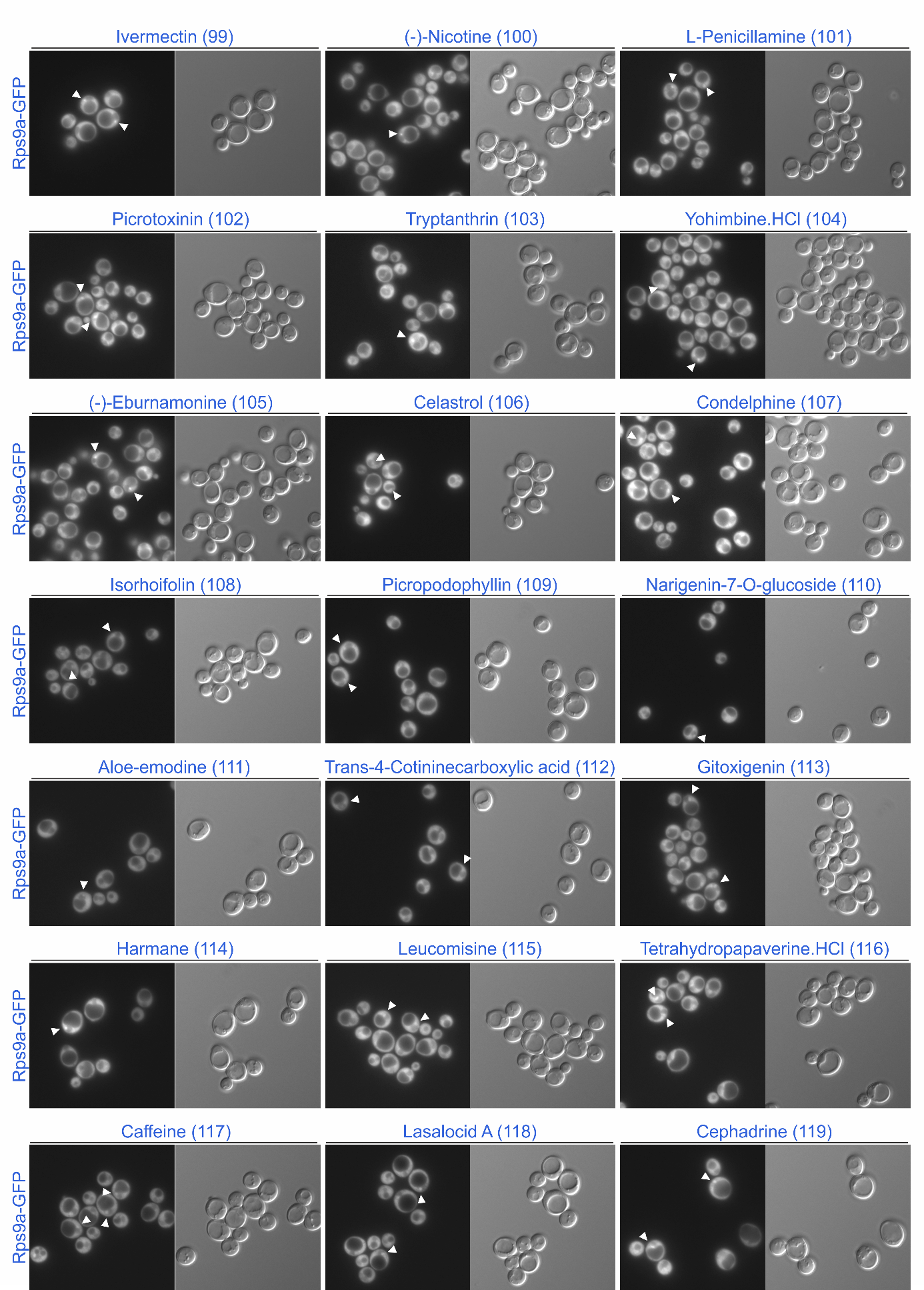


**Figure S7**: **Inhibitors causing nuclear accumulation of the Rps9a-GFP (40S) reporter (continued).** The eGFP pictures are shown on the left, the DIC pictures on the right, respectively. One representative picture is shown for each compound.


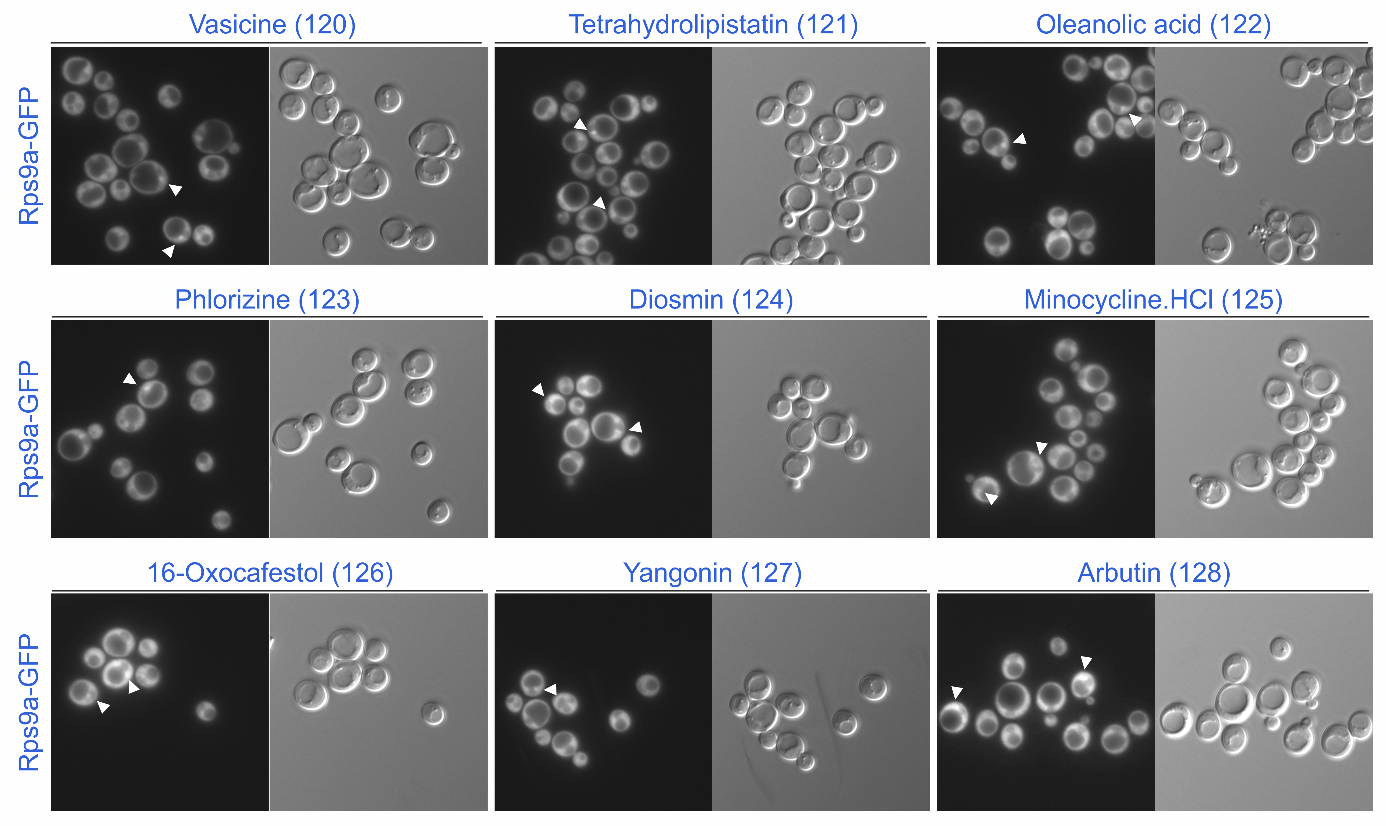


**Figure S8**: **Inhibitors causing nuclear accumulation of the Rps9a-GFP (40S) reporter (continued).** The eGFP pictures are shown on the left, the DIC pictures on the right, respectively. One representative picture is shown for each compound.


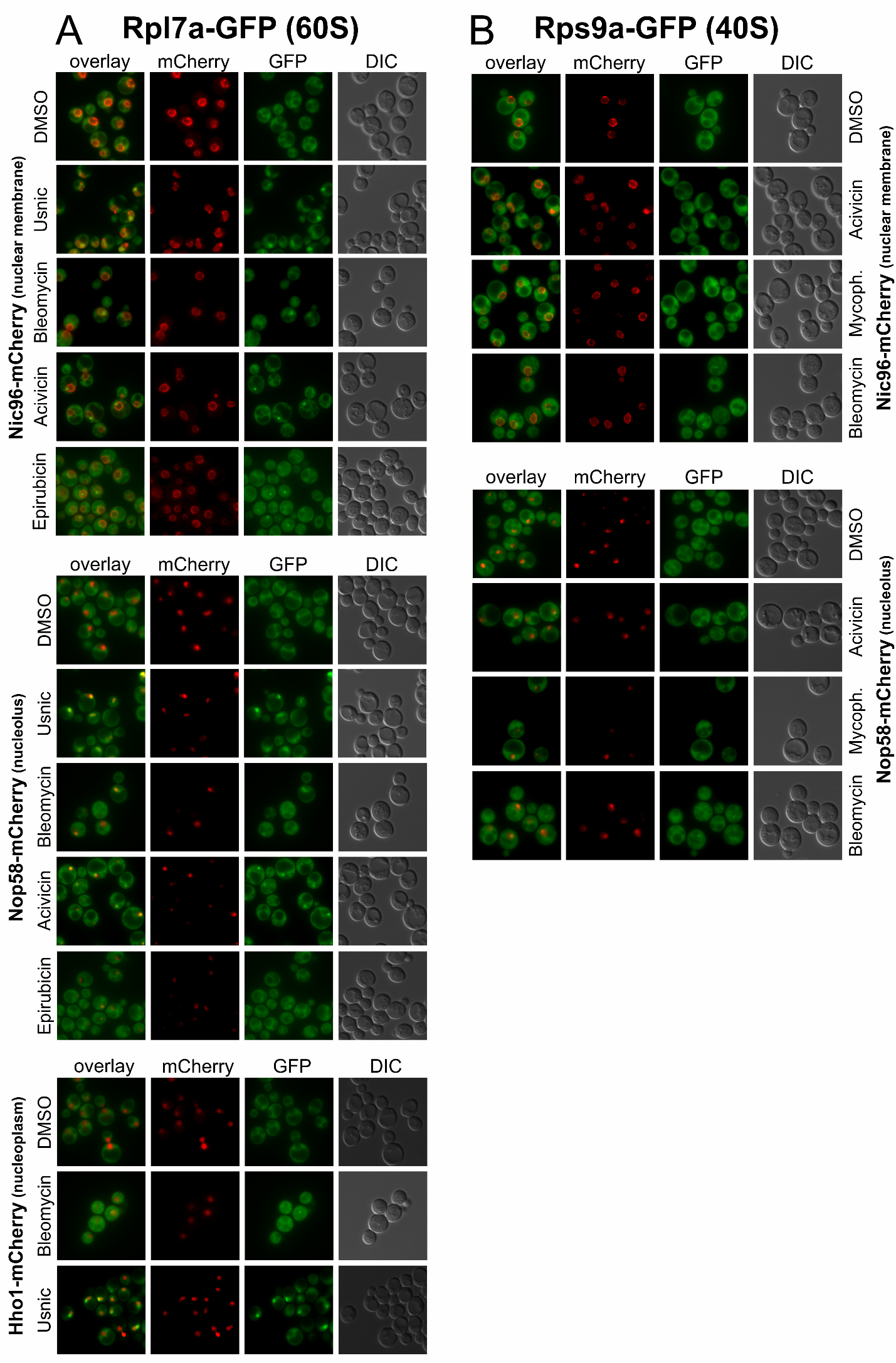


**Figure S9: Different classes of localization phenotypes upon inhibitor treatment.** To evaluate the exact localization of the GFP-signal accumulation upon inhibitor treatment, selected nuclear sub-compartment marker proteins were fused to a 3x-mCherry tag in the two ribosomal subunit reporter strains for the large 60S subunit **(Rpl7a-GFP, A)** and the small 40S subunit **(Rps9a-GFP, B)**. Nic96-mCherry served as nuclear membrane marker, Nop58-mCherry as marker for the nucleolus and Hho1-mCherry was used as marker for the nucleoplasm. The reporter strains were treated with selected compounds to display different classes of localization phenotypes. (Mycoph. = Mycophenolic acid; Bleomycin = Bleomycin sulfate; Usnic = Usnic acid).


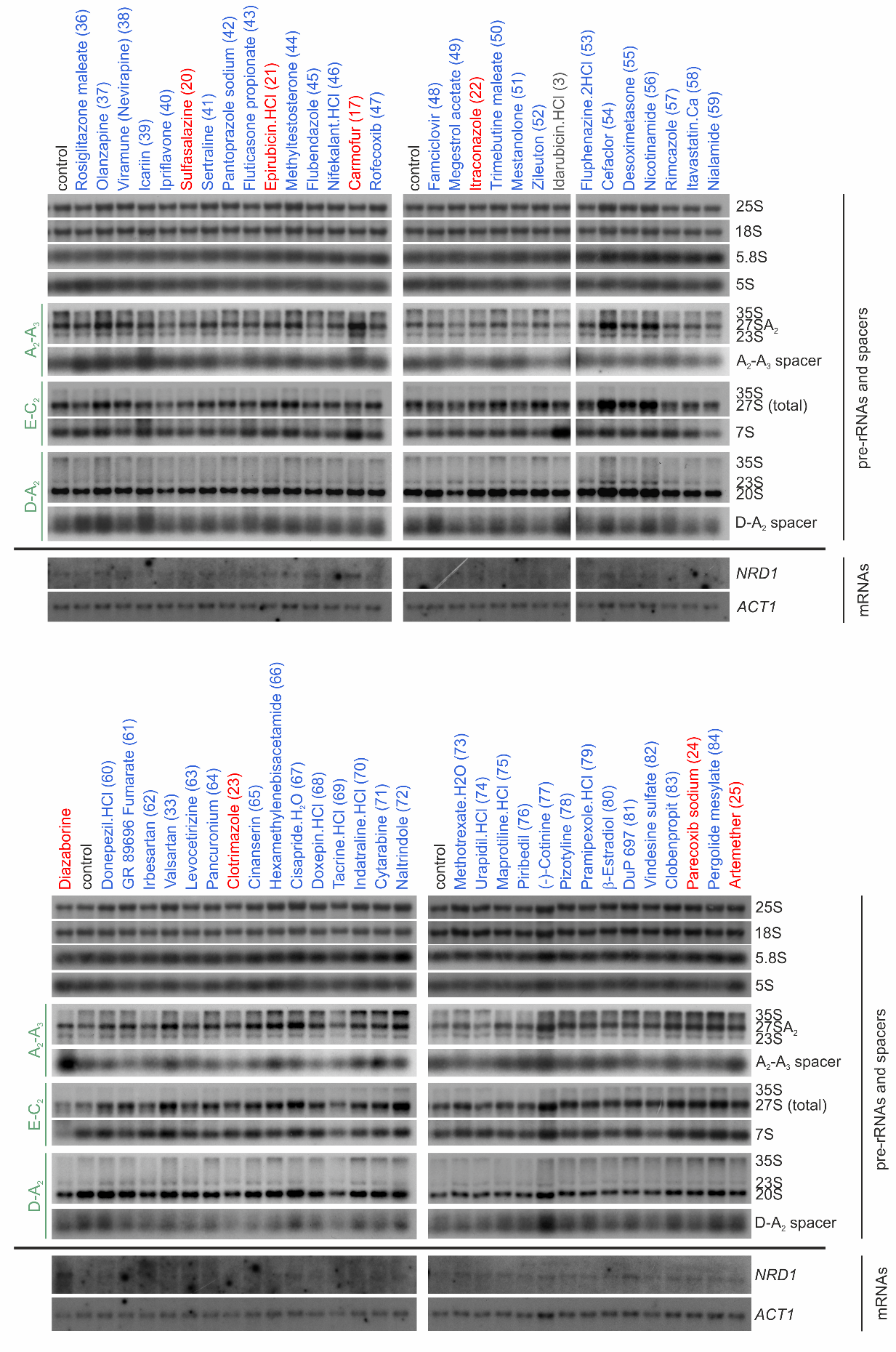


**Figure S10: rRNA processing phenotypes caused by the inhibitors from the NIH inhibitor collection.** Results from one of the two Northern blot analysis rounds are shown. The rRNA species detected are indicated on the right, the probes used to detect the respective pre-rRNAs are indicated on the left side. Inhibitors found in the 60S reporter screen are marked by red lettering, inhibitors from the 40S screen are written in blue and inhibitors identified in both screens in grey. In addition, the level of the exosome target *NRD1* mRNA was monitored with the long-lived *ACT1* mRNA as control.


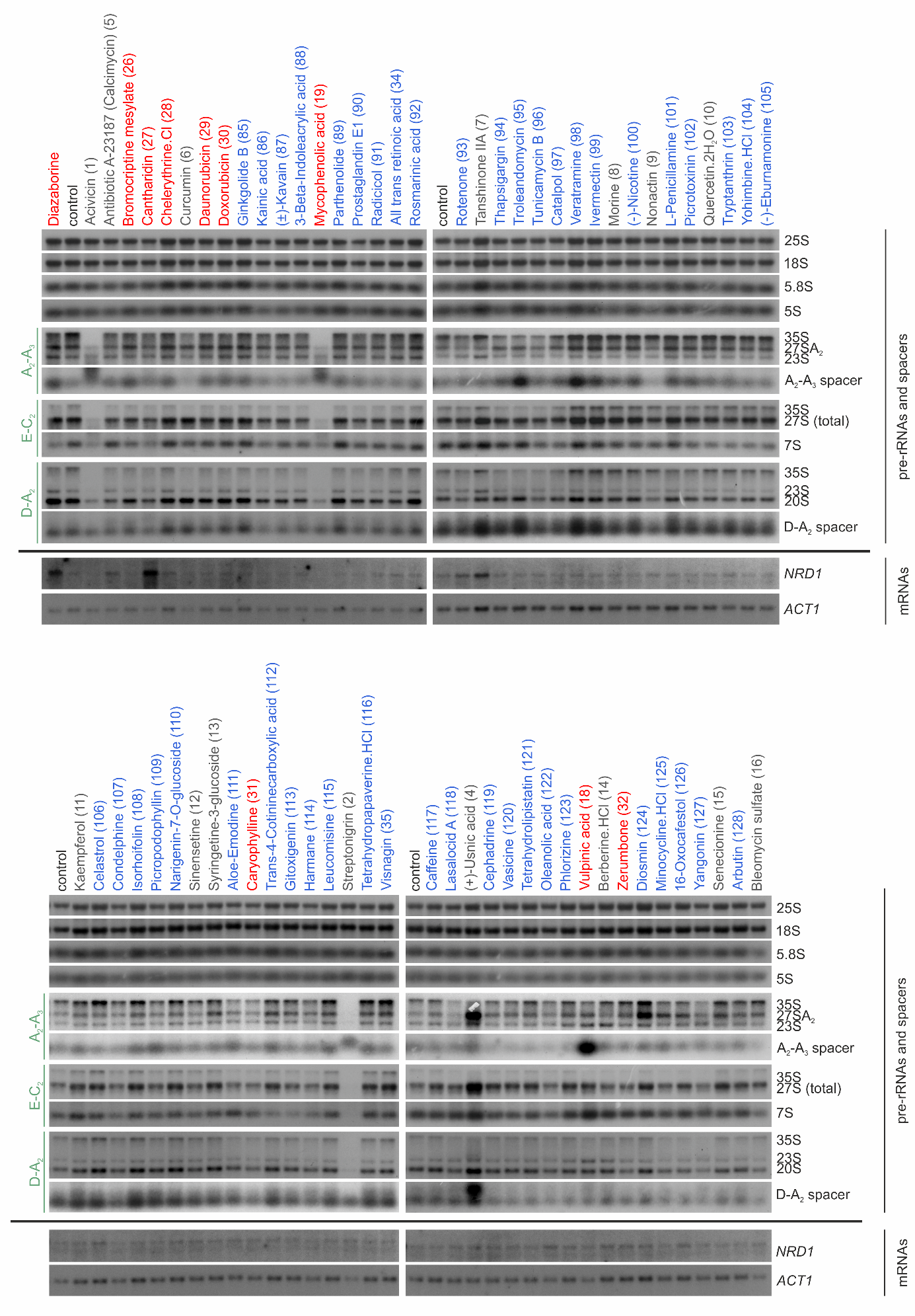


**Figure S11: rRNA processing phenotypes caused by the inhibitors from the Enzo inhibitor collection.** Results from one of the two Northern blot analysis rounds are shown. The rRNA species detected are indicated on the right, the probes used to detect the respective pre-rRNAs are indicated on the left side. Inhibitors found in the 60S reporter screen are marked by red lettering, inhibitors from the 40S screen are written in blue and inhibitors identified in both screens in grey. In addition, the level of the exosome target *NRD1* mRNA was monitored with the long-lived *ACT1* mRNA as control.


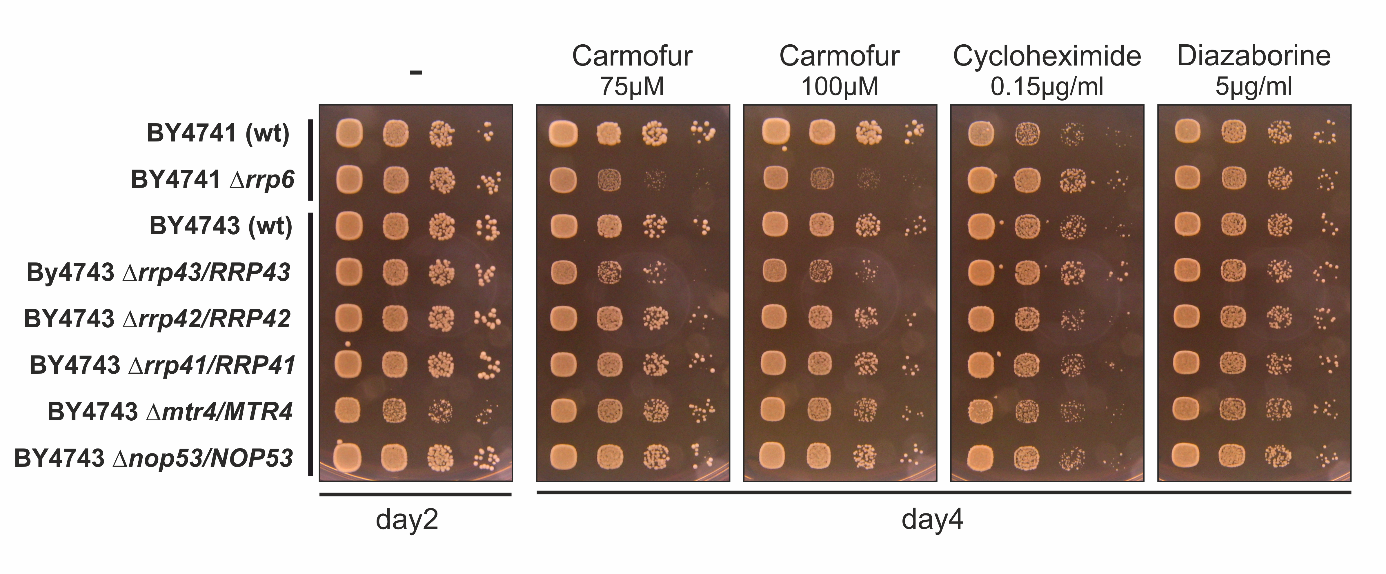


**Figure S12: Deletion of exosome factors cause hypersensitivity to Carmofur.** A haploid yeast strain carrying a deletion of the non-essential *RRP6*, or heterozygous diploid strains carrying *RRP43, RRP42, RRP41, MTR4,* or *NOP53*) deletions were spotted on agar plates containing either Carmofur, Cycloheximide, Diazaborine or no inhibitor (-) and incubated for the indicated number of days at 30°C.


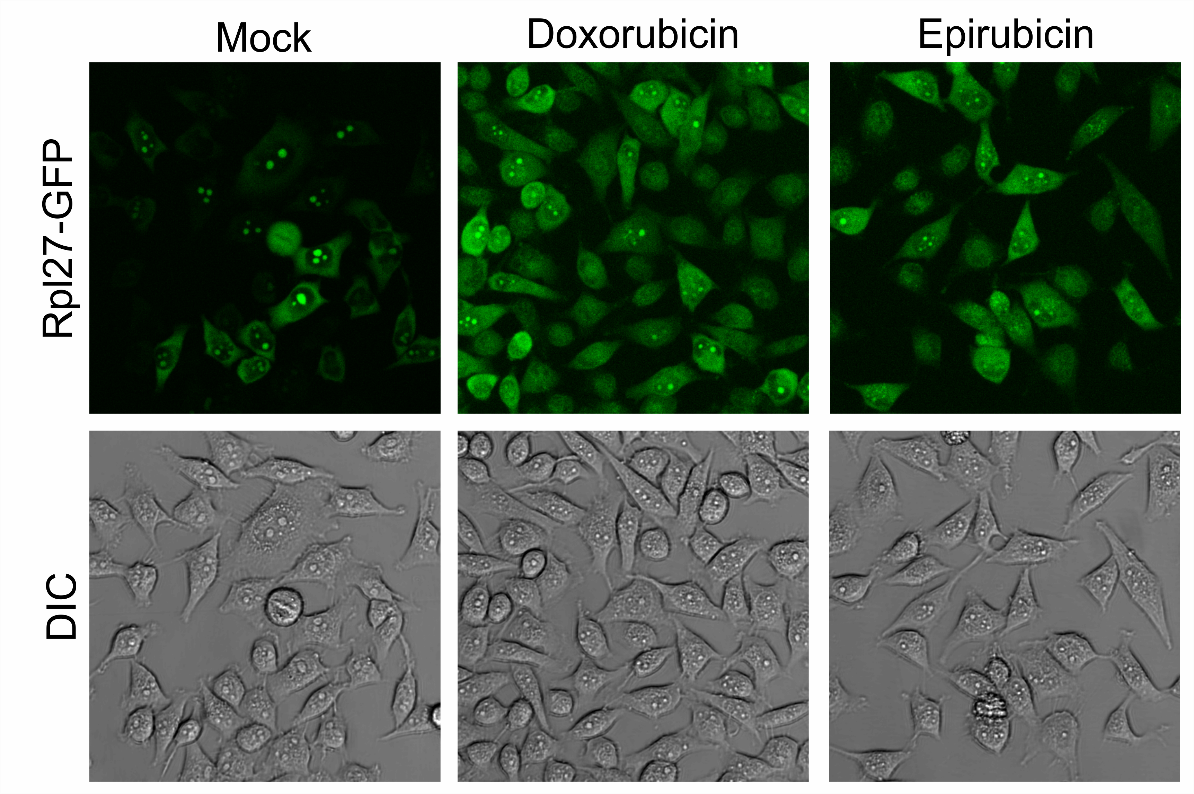


**Figure S13: Treatment with Doxorubicin and Epirubicin causes nucleoplasmic accumulation** **of an Rpl27-GFP reporter and nucleolar fragmentation in HeLa cells.** HeLa^Rpl27-GFP^ cells stably expressing the ribosomal reporter protein Rpl27-GFP were cultured in FluoroBrite^TM^ DMEM medium (Thermo scientific) for 24h before treatment with 1µM of the indicated compounds for 5h and inspection by Laser scanning microscopy.
