# Supplementary material for: Inhibiting eukaryotic ribosome biogenesis
Source: BMC Biol. 2019 Jun 10;17:46. doi: 10.1186/s12915-019-0664-2 (PMC6558755; doi:10.1186/s12915-019-0664-2)
Supplement: Supplementary file 2 — Table S1. Complete list of compounds contained in the “NIH clinical collection” as provided by the distributor. Table S2. Complete list of compounds contained in the “Enzo Natural Product Library” as provided by the distributor. (PDF 1086 kb) [file 12915_2019_664_MOESM2_ESM.pdf]

Table S1. NIH Clinical collection

NCC PLATE	PLATE BARCODE	WELL ID	STRUCTURE ID	SAMPLE ID	PUBCHEM SID	STRUCTURE SYNONYMS [1]	STRUCTURE SYNONYMS [2]	DATE	CONC	CONC UNIT	VOLUME	VOLUME UNIT	SOLVENT
**NGP-105-01**	NCP003323	A02	CPD000058423	SAM001246723	46386703	CPD000058423	BUPROPION HYDROCHLORIDE	18.Jun.13	10.0	mM	50.0	uL	DMSO
**NGP-105-01**	NCP003323	B02	CPD000471621	SAM001246718	46386698	CPD000471621	IRSOGLADINE MALEATE	18.Jun.13	10.0	mM	50.0	uL	DMSO
**NGP-105-01**	NCP003323	C02	CPD000466376	SAM001246733	46386746	CPD000466376	ACARBOSE	18.Jun.13	10.0	mM	50.0	uL	DMSO
**NGP-105-01**	NCP003323	D02	CPD000469294	SAM001246739	46386752	CPD000469294	BENPROPERINE PHOSPHATE	18.Jun.13	10.0	mM	50.0	uL	DMSO
**NGP-105-01**	NCP003323	E02	CPD000466378	SAM001246740	46386753	CPD000466378		18.Jun.13	10,1	mM	50.0	uL	DMSO
**NGP-105-01**	NCP003323	F02	CPD000058926	SAM001246743	46386756	CPD000058926		18.Jun.13	10.0	mM	50.0	uL	DMSO
**NGP-105-01**	NCP003323	G02	CPD000449280	SAM001246736	46386749	CPD000449280	Carvedilol	18.Jun.13	10.0	mM	50.0	uL	DMSO
**NGP-105-01**	NCP003323	H02	CPD000466379	SAM001246741	46386754	CPD000466379	LOMIFYLLINE	18.Jun.13	10.0	mM	50.0	uL	DMSO
**NGP-105-01**	NCP003323	A03	CPD000466380	SAM001246742	46386755	CPD000466380	PAZUFLOXACIN	18.Jun.13	10.0	mM	50.0	uL	DMSO
**NGP-105-01**	NCP003323	B03	CPD000466381	SAM001246745	46386758	CPD000466381	MIGLITOL	18.Jun.13	10.0	mM	50.0	uL	DMSO
**NGP-105-01**	NCP003323	C03	CPD000058373	SAM001246737	46386750	CPD000058373		18.Jun.13	10.0	mM	50.0	uL	DMSO
**NGP-105-01**	NCP003323	D03	CPD000466345	SAM001246652	46386668	CPD000466345	OLANZAPINE	18.Jun.13	9,9	mM	50.0	uL	DMSO
**NGP-105-01**	NCP003323	E03	CPD000449297	SAM001246653	46386669	CPD000449297	Nefazodone	18.Jun.13	10.0	mM	50.0	uL	DMSO
**NGP-105-01**	NCP003323	F03	CPD000469185	SAM001246654	46386670	CPD000469185	Moxifloxacin hydrochloride	18.Jun.13	10.0	mM	50.0	uL	DMSO
**NGP-105-01**	NCP003323	G03	CPD000469186	SAM001246655	46386712	CPD000469186	NELFINAVIR MESYLATE	18.Jun.13	10.0	mM	50.0	uL	DMSO
**NGP-105-01**	NCP003323	H03	CPD000469187	SAM001246656	46386713	CPD000469187	PRAVASTATIN Sodium	18.Jun.13	9,5	mM	50.0	uL	DMSO
**NGP-105-01**	NCP003323	A04	CPD000466344	SAM001246651	46386667	CPD000466344	Topotecan Hydrochloride	18.Jun.13	10.0	mM	50.0	uL	DMSO
**NGP-105-01**	NCP003323	B04	CPD000466303	SAM001246539	46386557	CPD000466303	LEVETIRACETAM	18.Jun.13	10,1	mM	50.0	uL	DMSO
**NGP-105-01**	NCP003323	C04	CPD000469142	SAM001246540	46386558	CPD000469142	PRAMIPEXOLE HCl	18.Jun.13	10.0	mM	50.0	uL	DMSO
**NGP-105-01**	NCP003323	D04	CPD000466323	SAM001246595	46386613	CPD000466323	RISPERIDONE	18.Jun.13	10.0	mM	50.0	uL	DMSO
**NGP-105-01**	NCP003323	E04	CPD000469167	SAM001246600	46386618	CPD000469167	pioglitazone hydrochloride	18.Jun.13	10.0	mM	50.0	uL	DMSO
**NGP-105-01**	NCP003323	F04	CPD000469147	SAM001246552	46386570	CPD000469147	Cilastatin sodium	18.Jun.13	10.0	mM	50.0	uL	DMSO
**NGP-105-01**	NCP003323	G04	CPD000466348	SAM001246661	46386718	CPD000466348	ARGATROBAN	18.Jun.13	10,1	mM	50.0	uL	DMSO
**NGP-105-01**	NCP003323	H04	CPD000466327	SAM001246603	46386621	CPD000466327	VALDECOXIB	18.Jun.13	10.0	mM	50.0	uL	DMSO
**NGP-105-01**	NCP003323	A05	CPD000466346	SAM001246658	46386715	CPD000466346	NAFTOPIDIL	18.Jun.13	10.0	mM	50.0	uL	DMSO
**NGP-105-01**	NCP003323	B05	CPD000156231	SAM001246662	46386719	CPD000156231	Nobiletin	18.Jun.13	10.0	mM	50.0	uL	DMSO
**NGP-105-01**	NCP003323	C05	CPD000466304	SAM001246541	46386559	CPD000466304	FINASTERIDE	18.Jun.13	10.0	mM	50.0	uL	DMSO
**NGP-105-01**	NCP003323	D05	CPD000469145	SAM001246549	46386567	CPD000469145	ZOLPIDEM TARTRATE	18.Jun.13	10.0	mM	50.0	uL	DMSO
**NGP-105-01**	NCP003323	E05	CPD000048458	SAM001246551	46386569	CPD000048458	Viramune	18.Jun.13	10.0	mM	50.0	uL	DMSO
**NGP-105-01**	NCP003323	F05	CPD000466325	SAM001246601	46386619	CPD000466325	TOPIRAMATE	18.Jun.13	10.0	mM	50.0	uL	DMSO

Table S1. NIH Clinical collection

NCC PLATE	PLATE BARCODE	WELL ID	STRUCTURE ID	SAMPLE ID	PUBCHEM SID	STRUCTURE SYNONYMS [1]	STRUCTURE SYNONYMS [2]	DATE	CONC	CONC UNIT	VOLUME	VOLUME UNIT	SOLVENT
**NGP-105-01**	NCP003323	G05	CPD000466350	SAM001246664	46386721	CPD000466350	VORICONAZOLE	18.Jun.13	9,9	mM	50.0	uL	DMSO
**NGP-105-01**	NCP003323	H05	CPD000469190	SAM001246665	46386722	CPD000469190	FENOLDOPAM MESYLATE	18.Jun.13	10.0	mM	50.0	uL	DMSO
**NGP-105-01**	NCP003323	A06	CPD000471612	SAM001246610	46386628	CPD000471612	ROSIGLITAZONE MALEATE	18.Jun.13	10.0	mM	50.0	uL	DMSO
**NGP-105-01**	NCP003323	B06	CPD000469191	SAM001246668	46386725	CPD000469191	ESCITALOPRAM OXALATE	18.Jun.13	10.0	mM	50.0	uL	DMSO
**NGP-105-01**	NCP003323	C06	CPD000058866	SAM001246609	46386627	CPD000058866		18.Jun.13	10.0	mM	50.0	uL	DMSO
**NGP-105-01**	NCP003323	D06	CPD000466354	SAM001246671	46386728	CPD000466354	LATANOPROST	18.Jun.13	10,3	mM	50.0	uL	DMSO
**NGP-105-01**	NCP003323	E06	CPD000058576	SAM001246673	46386730	CPD000058576		18.Jun.13	10,1	mM	50.0	uL	DMSO
**NGP-105-01**	NCP003323	F06	CPD000466298	SAM001246666	46386723	CPD000466298	Sertraline	18.Jun.13	10.0	mM	50.0	uL	DMSO
**NGP-105-01**	NCP003323	G06	CPD000466353	SAM001246670	46386727	CPD000466353	CALCIPOTRIOL	18.Jun.13	10,1	mM	50.0	uL	DMSO
**NGP-105-01**	NCP003323	H06	CPD000466308	SAM001246559	46386577	CPD000466308	EPIRUBICIN HYDROCHLORIDE	18.Jun.13	10.0	mM	50.0	uL	DMSO
**NGP-105-01**	NCP003323	A07	CPD000466329	SAM001246612	46386630	CPD000466329	BICALUTAMIDE	18.Jun.13	10.0	mM	50.0	uL	DMSO
**NGP-105-01**	NCP003323	B07	CPD000469192	SAM001246672	46386729	CPD000469192	BENIDIPINE HCl	18.Jun.13	10.0	mM	50.0	uL	DMSO
**NGP-105-01**	NCP003323	C07	CPD000466352	SAM001246669	46386726	CPD000466352	AMLEXANOX	18.Jun.13	10.0	mM	50.0	uL	DMSO
**NGP-105-01**	NCP003323	D07	CPD000469148	SAM001246554	46386572	CPD000469148	CERIVASTATIN SODIUM	18.Jun.13	10.0	mM	50.0	uL	DMSO
**NGP-105-01**	NCP003323	E07	CPD000466309	SAM001246560	46386578	CPD000466309	ICARIIN	18.Jun.13	10.0	mM	50.0	uL	DMSO
**NGP-105-01**	NCP003323	F07	CPD000466310	SAM001246562	46386580	CPD000466310	METHYLANDROS TENEDIOL	18.Jun.13	10,1	mM	50.0	uL	DMSO
**NGP-105-01**	NCP003323	G07	CPD000466307	SAM001246553	46386571	CPD000466307	TRIPTOLIDE	18.Jun.13	10.0	mM	50.0	uL	DMSO
**NGP-105-01**	NCP003323	H07	CPD000469170	SAM001246608	46386626	CPD000469170	ROSIGLITAZONE HCl	18.Jun.13	10.0	mM	50.0	uL	DMSO
**NGP-105-01**	NCP003323	A08	CPD000059106	SAM001246561	46386579	CPD000059106		18.Jun.13	10.0	mM	50.0	uL	DMSO
**NGP-105-01**	NCP003323	B08	CPD000466392	SAM001246769	46386782	CPD000466392	OLIGOMYCIN C	18.Jun.13	10.0	mM	50.0	uL	DMSO
**NGP-105-01**	NCP003323	C08	CPD000469199	SAM001246712	46386692	CPD000469199	BENAZEPRIL HYDROCHLORIDE	18.Jun.13	10.0	mM	50.0	uL	DMSO
**NGP-105-01**	NCP003323	D08	CPD000058877	SAM001246765	46386778	CPD000058877		18.Jun.13	10,1	mM	50.0	uL	DMSO
**NGP-105-01**	NCP003323	E08	CPD000059060	SAM001246714	46386694	CPD000059060	35212-22-7	18.Jun.13	10.0	mM	50.0	uL	DMSO
**NGP-105-01**	NCP003323	F08	CPD000058286	SAM001246770	46386783	CPD000058286	OXAPROZIN	18.Jun.13	10.0	mM	50.0	uL	DMSO
**NGP-105-01**	NCP003323	G08	CPD000058510	SAM001246766	46386779	CPD000058510		18.Jun.13	10.0	mM	50.0	uL	DMSO
**NGP-105-01**	NCP003323	H08	CPD000469200	SAM001246713	46386693	CPD000469200	MOSAPRIDE CITRATE	18.Jun.13	10.0	mM	50.0	uL	DMSO
**NGP-105-01**	NCP003323	A09	CPD000466391	SAM001246767	46386780	CPD000466391	Isoquercitrin	18.Jun.13	10.0	mM	50.0	uL	DMSO
**NGP-105-01**	NCP003323	B09	CPD000058450	SAM001246763	46386776	CPD000058450		18.Jun.13	10.0	mM	50.0	uL	DMSO
**NGP-105-01**	NCP003323	C09	CPD000469164	SAM001246593	46386611	CPD000469164		18.Jun.13	10.0	mM	50.0	uL	DMSO
**NGP-105-01**	NCP003323	D09	CPD000466394	SAM001246776	46386811	CPD000466394	HYPEROSIDE	18.Jun.13	8,8	mM	50.0	uL	DMSO
**NGP-105-01**	NCP003323	E09	CPD000466322	SAM001246589	46386607	CPD000466322	RIFABUTIN	18.Jun.13	10.0	mM	50.0	uL	DMSO

Table S1. NIH Clinical collection

NCC PLATE	PLATE BARCODE	WELL ID	STRUCTURE ID	SAMPLE ID	PUBCHEM SID	STRUCTURE SYNONYMS [1]	STRUCTURE SYNONYMS [2]	DATE	CONC	CONC UNIT	VOLUME	VOLUME UNIT	SOLVENT
**NGP-105-01**	NCP003323	F09	CPD000469141	SAM001246533	46386551	CPD000469141	ESMOLOL HYDROCHLORIDE	18.Jun.13	10.0	mM	50.0	uL	DMSO
**NGP-105-01**	NCP003323	G09	CPD000466321	SAM001246586	46386604	CPD000466321	TADALAFIL	18.Jun.13	10.0	mM	50.0	uL	DMSO
**NGP-105-01**	NCP003323	H09	CPD000058957	SAM001246587	46386605	CPD000058957		18.Jun.13	10.0	mM	50.0	uL	DMSO
**NGP-105-01**	NCP003323	A10	CPD000058570	SAM001246768	46386781	CPD000058570	DOXORUBICIN HYDROCHLORIDE	18.Jun.13	10.0	mM	50.0	uL	DMSO
**NGP-105-01**	NCP003323	B10	CPD000469209	SAM001246764	46386777	CPD000469209	MOXONIDINE HCl	18.Jun.13	10.0	mM	50.0	uL	DMSO
**NGP-105-01**	NCP003323	C10	CPD000058302	SAM001246711	46386691	CPD000058302		18.Jun.13	10.0	mM	50.0	uL	DMSO
**NGP-105-01**	NCP003323	D10	CPD000387024	SAM001246571	46386589	CPD000387024	PEFLOXACIN MESYLATE	18.Jun.13	10.0	mM	50.0	uL	DMSO
**NGP-105-01**	NCP003323	E10	CPD000469154	SAM001246572	46386590	CPD000469154	Venlafaxine hydrochloride	18.Jun.13	10.0	mM	50.0	uL	DMSO
**NGP-105-01**	NCP003323	F10	CPD000469592	SAM001246591	46386609	CPD000469592	Pantoprazole Sodium	18.Jun.13	10.0	mM	50.0	uL	DMSO
**NGP-105-01**	NCP003323	G10	CPD000469159	SAM001246583	46386601	CPD000469159	FLUTICASONE PROPIONATE	18.Jun.13	10.0	mM	50.0	uL	DMSO
**NGP-105-01**	NCP003323	H10	CPD000469161	SAM001246588	46386606	CPD000469161	Indinavir Sulfate	18.Jun.13	10.0	mM	50.0	uL	DMSO
**NGP-105-01**	NCP003323	A11	CPD000469160	SAM001246585	46386603	CPD000469160	Midazolam Hydrochloride	18.Jun.13	10.0	mM	50.0	uL	DMSO
**NGP-105-01**	NCP003323	B11	CPD000466319	SAM001246582	46386600	CPD000466319	LAMIVUDINE	18.Jun.13	9,9	mM	50.0	uL	DMSO
**NGP-105-01**	NCP003323	C11	CPD000469151	SAM001246564	46386582	CPD000469151	366-70-1	18.Jun.13	10.0	mM	50.0	uL	DMSO
**NGP-105-01**	NCP003323	D11	CPD000469280	SAM001246538	46386556	CPD000469280	ESOMEPRAZOLE Mg	18.Jun.13	10.0	mM	50.0	uL	DMSO
**NGP-105-01**	NCP003323	E11	CPD000059146	SAM001246530	46386548	CPD000059146	SULFASALAZINE	18.Jun.13	10.0	mM	50.0	uL	DMSO
**NGP-105-01**	NCP003323	F11	CPD000466313	SAM001246567	46386585	CPD000466313	TORASEMIDE	18.Jun.13	9,9	mM	50.0	uL	DMSO
**NGP-105-01**	NCP003323	G11	CPD000469156	SAM001246576	46386594	CPD000469156	tropisetron┬áhyd rochloride	18.Jun.13	10.0	mM	50.0	uL	DMSO
**NGP-105-01**	NCP003323	H11	CPD000326795	SAM001246534	46386552	CPD000326795	Ranolazine dihydrochloride	18.Jun.13	10.0	mM	50.0	uL	DMSO
**NGP-105-02**	NCP003403	A02	CPD000338536	SAM001246761	46386774	CPD000338536		18.Jun.13	10.0	mM	50.0	uL	DMSO
**NGP-105-02**	NCP003403	B02	CPD000466390	SAM001246762	46386775	CPD000466390	PIDOTIMOD	18.Jun.13	10.0	mM	50.0	uL	DMSO
**NGP-105-02**	NCP003403	C02	CPD000466386	SAM001246757	46386770	CPD000466386	RAMIPRIL	18.Jun.13	10.0	mM	50.0	uL	DMSO
**NGP-105-02**	NCP003403	D02	CPD000469284	SAM001246642	46386658	CPD000469284	FENPIVERINIUM BROMIDE	18.Jun.13	10,1	mM	50.0	uL	DMSO
**NGP-105-02**	NCP003403	E02	CPD000058610	SAM001246751	46386764	CPD000058610	19-Nortestosterone	18.Jun.13	10.0	mM	50.0	uL	DMSO
**NGP-105-02**	NCP003403	F02	CPD000466384	SAM001246752	46386765	CPD000466384		18.Jun.13	10.0	mM	50.0	uL	DMSO
**NGP-105-02**	NCP003403	G02	CPD000059047	SAM001246753	46386766	CPD000059047		18.Jun.13	10,5	mM	50.0	uL	DMSO
**NGP-105-02**	NCP003403	H02	CPD000048684	SAM001246754	46386767	CPD000048684		18.Jun.13	10.0	mM	50.0	uL	DMSO
**NGP-105-02**	NCP003403	A03	CPD000466385	SAM001246756	46386769	CPD000466385	TROXIPIDE	18.Jun.13	10,1	mM	50.0	uL	DMSO

Table S1. NIH Clinical collection

NCC PLATE	PLATE BARCODE	WELL ID	STRUCTURE ID	SAMPLE ID	PUBCHEM SID	STRUCTURE SYNONYMS [1]	STRUCTURE SYNONYMS [2]	DATE	CONC	CONC UNIT	VOLUME	VOLUME UNIT	SOLVENT
**NGP-105-02**	NCP003403	B03	CPD000466341	SAM001246647	46386663	CPD000466341	ACTARIT	18.Jun.13	10.0	mM	50.0	uL	DMSO
**NGP-105-02**	NCP003403	C03	CPD000469183	SAM001246645	46386661	CPD000469183	azelastine hydrochloride	18.Jun.13	10.0	mM	50.0	uL	DMSO
**NGP-105-02**	NCP003403	D03	CPD000466388	SAM001246759	46386772	CPD000466388	TOCAINIDE	18.Jun.13	10.0	mM	50.0	uL	DMSO
**NGP-105-02**	NCP003403	E03	CPD000499525	SAM001246760	46386773	CPD000499525	TAXIFOLIN-(+/-)	18.Jun.13	10,1	mM	50.0	uL	DMSO
**NGP-105-02**	NCP003403	F03	CPD000466387	SAM001246758	46386771	CPD000466387	LEVOFLOXACIN	18.Jun.13	10.0	mM	50.0	uL	DMSO
**NGP-105-02**	NCP003403	G03	CPD000469182	SAM001246643	46386659	CPD000469182	CEFATRIZINE PROPYLENE GLYCOL	18.Jun.13	10.0	mM	50.0	uL	DMSO
**NGP-105-02**	NCP003403	H03	CPD000466364	SAM001246700	46386682	CPD000466364	IDEBENONE	18.Jun.13	10,2	mM	50.0	uL	DMSO
**NGP-105-02**	NCP003403	A04	CPD000466366	SAM001246703	46386685	CPD000466366	LEVOSULPIRIDE	18.Jun.13	10.0	mM	50.0	uL	DMSO
**NGP-105-02**	NCP003403	B04	CPD000238142	SAM001246706	46386687	CPD000238142		18.Jun.13	10.0	mM	50.0	uL	DMSO
**NGP-105-02**	NCP003403	C04	CPD000466343	SAM001246649	46386665	CPD000466343	LETROZOLE	18.Jun.13	10.0	mM	50.0	uL	DMSO
**NGP-105-02**	NCP003403	D04	CPD000469184	SAM001246650	46386666	CPD000469184	MEROPENEM	18.Jun.13	10.0	mM	50.0	uL	DMSO
**NGP-105-02**	NCP003403	E04	CPD000466339	SAM001246637	46386654	CPD000466339	ORLISTAT	18.Jun.13	10.0	mM	50.0	uL	DMSO
**NGP-105-02**	NCP003403	F04	CPD000469179	SAM001246631	46386648	CPD000469179		18.Jun.13	10.0	mM	50.0	uL	DMSO
**NGP-105-02**	NCP003403	G04	CPD000059117	SAM001246694	46386677	CPD000059117		18.Jun.13	10.0	mM	50.0	uL	DMSO
**NGP-105-02**	NCP003403	H04	CPD000469197	SAM001246695	46386678	CPD000469197	CETRAXATE HCl	18.Jun.13	10.0	mM	50.0	uL	DMSO
**NGP-105-02**	NCP003403	A05	CPD000149316	SAM001246696	46386679	CPD000149316		18.Jun.13	10.0	mM	50.0	uL	DMSO
**NGP-105-02**	NCP003403	B05	CPD000058464	SAM001246697	46386680	CPD000058464		18.Jun.13	10,3	mM	50.0	uL	DMSO
**NGP-105-02**	NCP003403	C05	CPD000059145	SAM001246702	46386684	CPD000059145	N-Ethyl-o-crotonotoluidide	18.Jun.13	10,3	mM	50.0	uL	DMSO
**NGP-105-02**	NCP003403	D05	CPD000472526	SAM001246699	46386681	CPD000472526	Amfebutamone	18.Jun.13	10,1	mM	50.0	uL	DMSO
**NGP-105-02**	NCP003403	E05	CPD000466340	SAM001246638	46386655	CPD000466340	ALFUZOSIN	18.Jun.13	10.0	mM	50.0	uL	DMSO
**NGP-105-02**	NCP003403	F05	CPD000449309	SAM001246639	46386656	CPD000449309	Amisulpride	18.Jun.13	10.0	mM	50.0	uL	DMSO
**NGP-105-02**	NCP003403	G05	CPD000469292	SAM001246632	46386649	CPD000469292	LOFEPRAMINE	18.Jun.13	10.0	mM	50.0	uL	DMSO
**NGP-105-02**	NCP003403	H05	CPD000466362	SAM001246688	46386671	CPD000466362	PEROSPIRONE HCl	18.Jun.13	10.0	mM	50.0	uL	DMSO
**NGP-105-02**	NCP003403	A06	CPD000059010	SAM001246689	46386672	CPD000059010	DOCETAXEL	18.Jun.13	10.0	mM	50.0	uL	DMSO
**NGP-105-02**	NCP003403	B06	CPD000387107	SAM001246690	46386673	CPD000387107	HONOKIOL	18.Jun.13	10,1	mM	50.0	uL	DMSO
**NGP-105-02**	NCP003403	C06	CPD000469196	SAM001246691	46386674	CPD000469196	TOLTERODINE TARTRATE	18.Jun.13	10.0	mM	50.0	uL	DMSO
**NGP-105-02**	NCP003403	D06	CPD000466363	SAM001246692	46386675	CPD000466363	CARMOFUR	18.Jun.13	10,3	mM	50.0	uL	DMSO
**NGP-105-02**	NCP003403	E06	CPD000469181	SAM001246633	46386650	CPD000469181	PAROXETINE	18.Jun.13	9,7	mM	50.0	uL	DMSO
**NGP-105-02**	NCP003403	F06	CPD000466337	SAM001246634	46386651	CPD000466337	OLMESARTAN MEDOXOMIL	18.Jun.13	10.0	mM	50.0	uL	DMSO
**NGP-105-02**	NCP003403	G06	CPD000469593	SAM001246635	46386652	CPD000469593	LOSARTAN Potassium	18.Jun.13	10.0	mM	50.0	uL	DMSO
**NGP-105-02**	NCP003403	H06	CPD000466338	SAM001246636	46386653	CPD000466338	TEMOZOLOMIDE	18.Jun.13	10.0	mM	50.0	uL	DMSO
**NGP-105-02**	NCP003403	A07	CPD000058528	SAM001246682	46386739	CPD000058528		18.Jun.13	10.0	mM	50.0	uL	DMSO

Table S1. NIH Clinical collection

NCC PLATE	PLATE BARCODE	WELL ID	STRUCTURE ID	SAMPLE ID	PUBCHEM SID	STRUCTURE SYNONYMS [1]	STRUCTURE SYNONYMS [2]	DATE	CONC	CONC UNIT	VOLUME	VOLUME UNIT	SOLVENT
**NGP-105-02**	NCP003403	B07	CPD000469195	SAM001246686	46386743	CPD000469195	tosufloxacin tosilate	18.Jun.13	10.0	mM	50.0	uL	DMSO
**NGP-105-02**	NCP003403	C07	CPD000466361	SAM001246687	46386744	CPD000466361	MECILLINAM	18.Jun.13	10.0	mM	50.0	uL	DMSO
**NGP-105-02**	NCP003403	D07	CPD000469177	SAM001246626	46386643	CPD000469177	Atomoxetine hydrochloride	18.Jun.13	10.0	mM	50.0	uL	DMSO
**NGP-105-02**	NCP003403	E07	CPD000466336	SAM001246628	46386645	CPD000466336	ARTESUNATE	18.Jun.13	10.0	mM	50.0	uL	DMSO
**NGP-105-02**	NCP003403	F07	CPD000058959	SAM001246679	46386736	CPD000058959		18.Jun.13	10,1	mM	50.0	uL	DMSO
**NGP-105-02**	NCP003403	G07	CPD000469193	SAM001246674	46386731	CPD000469193	CEFPODOXIME PROXETIL	18.Jun.13	10.0	mM	50.0	uL	DMSO
**NGP-105-02**	NCP003403	H07	CPD000058803	SAM001246680	46386737	CPD000058803	Buflomedil HCl	18.Jun.13	10.0	mM	50.0	uL	DMSO
**NGP-105-02**	NCP003403	A08	CPD000012114	SAM001246614	46386631	CPD000012114	4-Chloro-N-(2-morpholin-4-yl-ethyl)-benzamide	18.Jun.13	10.0	mM	50.0	uL	DMSO
**NGP-105-02**	NCP003403	B08	CPD000466330	SAM001246616	46386633	CPD000466330	HALOMETASONE MONOHYDRATE	18.Jun.13	10.0	mM	50.0	uL	DMSO
**NGP-105-02**	NCP003403	C08	CPD000466357	SAM001246681	46386738	CPD000466357	TRICLABENDAZOLE	18.Jun.13	10.0	mM	50.0	uL	DMSO
**NGP-105-02**	NCP003403	D08	CPD000466331	SAM001246617	46386634	CPD000466331	ROFECOXIB	18.Jun.13	10.0	mM	50.0	uL	DMSO
**NGP-105-02**	NCP003403	E08	CPD000471619	SAM001246618	46386635	CPD000471619	BISOPROLOL FUMARATE	18.Jun.13	10.0	mM	50.0	uL	DMSO
**NGP-105-02**	NCP003403	F08	CPD000466334	SAM001246623	46386640	CPD000466334	EZETIMIBE	18.Jun.13	10.0	mM	50.0	uL	DMSO
**NGP-105-02**	NCP003403	G08	CPD000469176	SAM001246625	46386642	CPD000469176	TIAGABINE HCl	18.Jun.13	10.0	mM	50.0	uL	DMSO
**NGP-105-02**	NCP003403	H08	CPD000466355	SAM001246676	46386733	CPD000466355	idarubicin hydrochloride	18.Jun.13	10.0	mM	50.0	uL	DMSO
**NGP-105-02**	NCP003403	A09	CPD000466360	SAM001246685	46386742	CPD000466360	FLUBENDAZOLE	18.Jun.13	10.0	mM	50.0	uL	DMSO
**NGP-105-02**	NCP003403	B09	CPD000466356	SAM001246677	46386734	CPD000466356	TACROLIMUS	18.Jun.13	10.0	mM	50.0	uL	DMSO
**NGP-105-02**	NCP003403	C09	CPD000469208	SAM001246749	46386762	CPD000469208	VALACICLOVIR HYDROCHLORIDE	18.Jun.13	10.0	mM	50.0	uL	DMSO
**NGP-105-02**	NCP003403	D09	CPD000466382	SAM001246748	46386761	CPD000466382	CLARITHROMYCIN	18.Jun.13	10.0	mM	50.0	uL	DMSO
**NGP-105-02**	NCP003403	E09	CPD000466383	SAM001246750	46386763	CPD000466383	ARIPIPRAZOLE	18.Jun.13	10.0	mM	50.0	uL	DMSO
**NGP-105-02**	NCP003403	F09	CPD000471622	SAM001246747	46386760	CPD000471622	TRIMEBUTINE MALEATE	18.Jun.13	10.0	mM	50.0	uL	DMSO
**NGP-105-02**	NCP003403	G09	CPD000238198	SAM001246746	46386759	CPD000238198		18.Jun.13	10,1	mM	50.0	uL	DMSO
**NGP-105-02**	NCP003403	H09	CPD000466370	SAM001246719	46386699	CPD000466370	NISOLDIPINE	18.Jun.13	10.0	mM	50.0	uL	DMSO
**NGP-105-02**	NCP003403	A10	CPD000466371	SAM001246720	46386700	CPD000466371	PICEID	18.Jun.13	10.0	mM	50.0	uL	DMSO
**NGP-105-02**	NCP003403	B10	CPD000149359	SAM001246716	46386696	CPD000149359	1-(2-Methyl-5-nitro-imidazol-1-yl)-propan-2-ol	18.Jun.13	10.0	mM	50.0	uL	DMSO
**NGP-105-02**	NCP003403	C10	CPD000466369	SAM001246717	46386697	CPD000466369	Nifekalant hydrochloride	18.Jun.13	10.0	mM	50.0	uL	DMSO
**NGP-105-02**	NCP003403	D10	CPD000466372	SAM001246721	46386701	CPD000466372	NATEGLINIDE	18.Jun.13	10.0	mM	50.0	uL	DMSO
**NGP-105-02**	NCP003403	E10	CPD000058691	SAM001246722	46386702	CPD000058691		18.Jun.13	10.0	mM	50.0	uL	DMSO
**NGP-105-02**	NCP003403	F10	CPD000466374	SAM001246728	46386708	CPD000466374	ORMETOPRIM	18.Jun.13	10.0	mM	50.0	uL	DMSO
**NGP-105-02**	NCP003403	G10	CPD000466377	SAM001246738	46386751	CPD000466377	ZILEUTON	18.Jun.13	10.0	mM	50.0	uL	DMSO
**NGP-105-02**	NCP003403	H10	CPD000058350	SAM001246729	46386709	CPD000058350		18.Jun.13	10.0	mM	50.0	uL	DMSO
**NGP-105-02**	NCP003403	A11	CPD000058918	SAM001246730	46386710	CPD000058918		18.Jun.13	10.0	mM	50.0	uL	DMSO
**NGP-105-02**	NCP003403	B11	CPD000469293	SAM001246724	46386704	CPD000469293	OXICONAZOLE NITRATE	18.Jun.13	10.0	mM	50.0	uL	DMSO
**NGP-105-02**	NCP003403	C11	CPD000469235	SAM001246731	46386711	CPD000469235	KITASAMYCIN	18.Jun.13	10.0	mM	50.0	uL	DMSO
**NGP-105-02**	NCP003403	D11	CPD000466375	SAM001246732	46386745	CPD000466375	FAMCICLOVIR	18.Jun.13	10.0	mM	50.0	uL	DMSO

Table S1. NIH Clinical collection

NCC PLATE	PLATE BARCODE	WELL ID	STRUCTURE ID	SAMPLE ID	PUBCHEM SID	STRUCTURE SYNONYMS [1]	STRUCTURE SYNONYMS [2]	DATE	CONC	CONC UNIT	VOLUME	VOLUME UNIT	SOLVENT
**NGP-105-02**	NCP003403	E11	CPD000326828	SAM001246725	46386705	CPD000326828		18.Jun.13	10.0	mM	50.0	uL	DMSO
**NGP-105-02**	NCP003403	F11	CPD000466373	SAM001246726	46386706	CPD000466373	rufloxacin monohydrochloride	18.Jun.13	10.0	mM	50.0	uL	DMSO
**NGP-105-02**	NCP003403	G11	CPD000466389	SAM001246778	46386813	CPD000466389	TAXIFOLIN-(+)	18.Jun.13	10.0	mM	50.0	uL	DMSO
**NGP-105-02**	NCP003403	H11	CPD000469211	SAM001246782	46386816	CPD000469211	alosetron┬ámonohydrochl oride	18.Jun.13	10.0	mM	50.0	uL	DMSO
**NGP-105-03**	NCP003483	A02	CPD000059165	SAM001246806	46386830	CPD000059165	BESTATIN	18.Jun.13	10.0	mM	50.0	uL	DMSO
**NGP-105-03**	NCP003483	B02	CPD000469213	SAM001246774	46386786	CPD000469213	TOREMIFENE CITRATE	18.Jun.13	10.0	mM	50.0	uL	DMSO
**NGP-105-03**	NCP003483	C02	CPD000469214	SAM001246784	46386818	CPD000469214	GOSERELIN ACETATE	18.Jun.13	9,7	mM	50.0	uL	DMSO
**NGP-105-03**	NCP003483	D02	CPD000469212	SAM001246678	46386735	CPD000469212	SECOISOLARICIRESINOL	18.Jun.13	10.0	mM	50.0	uL	DMSO
**NGP-105-03**	NCP003483	E02	CPD000469217	SAM001246796	46386823	CPD000469217	RALTITREXED	18.Jun.13	10.0	mM	50.0	uL	DMSO
**NGP-105-03**	NCP003483	F02	CPD000469229	SAM001246792	46386821	CPD000469229	DOXAPRAM HYDROCHLORIDE	18.Jun.13	10,4	mM	50.0	uL	DMSO
**NGP-105-03**	NCP003483	G02	CPD000466294	SAM001247095	46387004	CPD000466294	RU 24969	18.Jun.13	10,1	mM	50.0	uL	DMSO
**NGP-105-03**	NCP003483	H02	CPD000112281	SAM001246865	46386939	CPD000112281	Brucine	18.Jun.13	10.0	mM	50.0	uL	DMSO
**NGP-105-03**	NCP003483	A03	CPD000059115	SAM001246862	46386936	CPD000059115	16502-01-5	18.Jun.13	10.0	mM	50.0	uL	DMSO
**NGP-105-03**	NCP003483	B03	CPD000058411	SAM001246863	46386937	CPD000058411		18.Jun.13	10.0	mM	50.0	uL	DMSO
**NGP-105-03**	NCP003483	D03	CPD000058746	SAM001246874	46386948	CPD000058746	NAPROXEN SODIUM	18.Jun.13	10.0	mM	50.0	uL	DMSO
**NGP-105-03**	NCP003483	E03	CPD000058904	SAM001246875	46386949	CPD000058904		18.Jun.13	10.0	mM	50.0	uL	DMSO
**NGP-105-03**	NCP003483	F03	CPD000058310	SAM001246877	46386951	CPD000058310	3-[3,5-DIBROMO-4-HYDROXYBENZOYL]-2-ETHYLBENZOFURAN	18.Jun.13	10.0	mM	50.0	uL	DMSO
**NGP-105-03**	NCP003483	G03	CPD000058300	SAM001246890	46386964	CPD000058300		18.Jun.13	10.0	mM	50.0	uL	DMSO
**NGP-105-03**	NCP003483	H03	CPD000058701	SAM001246892	46386966	CPD000058701		18.Jun.13	10.0	mM	50.0	uL	DMSO
**NGP-105-03**	NCP003483	A04	CPD000058715	SAM001246891	46386965	CPD000058715		18.Jun.13	10.0	mM	50.0	uL	DMSO
**NGP-105-03**	NCP003483	B04	CPD000058273	SAM001246876	46386950	CPD000058273		18.Jun.13	10.0	mM	50.0	uL	DMSO
**NGP-105-03**	NCP003483	C04	CPD000466922	SAM001246889	46386963	CPD000466922	Reichsteins substance S	18.Jun.13	10.0	mM	50.0	uL	DMSO
**NGP-105-03**	NCP003483	D04	CPD000059086	SAM001246894	46386968	CPD000059086	3-PYRIDINEMETHANOL	18.Jun.13	10.0	mM	50.0	uL	DMSO
**NGP-105-03**	NCP003483	E04	CPD000449283	SAM001246979	46386806	CPD000449283	Haloperidol	18.Jun.13	10.0	mM	50.0	uL	DMSO
**NGP-105-03**	NCP003483	F04	CPD000449279	SAM001247042	46386912	CPD000449279	Stiripentol	18.Jun.13	10.0	mM	50.0	uL	DMSO
**NGP-105-03**	NCP003483	G04	CPD000449303	SAM001247034	46386904	CPD000449303	Fluperlapine	18.Jun.13	10.0	mM	50.0	uL	DMSO
**NGP-105-03**	NCP003483	H04	CPD000058660	SAM001246887	46386961	CPD000058660		18.Jun.13	10.0	mM	50.0	uL	DMSO
**NGP-105-03**	NCP003483	A05	CPD000112358	SAM001246869	46386943	CPD000112358	Homoveratrylamine	18.Jun.13	10.0	mM	50.0	uL	DMSO
**NGP-105-03**	NCP003483	B05	CPD000058194	SAM001246913	46387025	CPD000058194		18.Jun.13	10.0	mM	50.0	uL	DMSO
**NGP-105-03**	NCP003483	C05	CPD000058741	SAM001246912	46387024	CPD000058741	XANTHINOL NICOTINATE	18.Jun.13	10.0	mM	50.0	uL	DMSO
**NGP-105-03**	NCP003483	D05	CPD000059111	SAM001246896	46386970	CPD000059111	SYNEPHRINE	18.Jun.13	10.0	mM	50.0	uL	DMSO
**NGP-105-03**	NCP003483	E05	CPD000058206	SAM001246888	46386962	CPD000058206	501-36-0	18.Jun.13	10.0	mM	50.0	uL	DMSO
**NGP-105-03**	NCP003483	F05	CPD000059093	SAM001246871	46386945	CPD000059093	118-71-8	18.Jun.13	10.0	mM	50.0	uL	DMSO
**NGP-105-03**	NCP003483	G05	CPD000059077	SAM001246854	46386887	CPD000059077		18.Jun.13	10.0	mM	50.0	uL	DMSO
**NGP-105-03**	NCP003483	H05	CPD000059011	SAM001246856	46386889	CPD000059011	ENROFLOXACIN	18.Jun.13	10.0	mM	50.0	uL	DMSO

Table S1. NIH Clinical collection

NCC PLATE	PLATE BARCODE	WELL ID	STRUCTURE ID	SAMPLE ID	PUBCHEM SID	STRUCTURE SYNONYMS [1]	STRUCTURE SYNONYMS [2]	DATE	CONC	CONC UNIT	VOLUME	VOLUME UNIT	SOLVENT
**NGP-105-03**	NCP003483	A06	CPD000058603	SAM001246857	46386890	CPD000058603		18.Jun.13	10.0	mM	50.0	uL	DMSO
**NGP-105-03**	NCP003483	B06	CPD000058250	SAM001246884	46386958	CPD000058250		18.Jun.13	10.0	mM	50.0	uL	DMSO
**NGP-105-03**	NCP003483	C06	CPD000059044	SAM001246858	46386891	CPD000059044		18.Jun.13	10.0	mM	50.0	uL	DMSO
**NGP-105-03**	NCP003483	D06	CPD000469136	SAM001246523	46386541	CPD000469136	duloxetine hydrochloride	18.Jun.13	10.0	mM	50.0	uL	DMSO
**NGP-105-03**	NCP003483	E06	CPD000469155	SAM001246573	46386591	CPD000469155	VARDENAFIL CITRATE	18.Jun.13	10.0	mM	50.0	uL	DMSO
**NGP-105-03**	NCP003483	F06	CPD000469137	SAM001246524	46386542	CPD000469137	Ropivacaine hydrochloride	18.Jun.13	9,9	mM	50.0	uL	DMSO
**NGP-105-03**	NCP003483	G06	CPD000466301	SAM001246525	46386543	CPD000466301	ANASTROZOLE	18.Jun.13	10.0	mM	50.0	uL	DMSO
**NGP-105-03**	NCP003483	H06	CPD000058462	SAM001246984	46386834	CPD000058462	KETOTIFEN FUMARATE	18.Jun.13	10.0	mM	50.0	uL	DMSO
**NGP-105-03**	NCP003483	A07	CPD000058769	SAM001246906	46387018	CPD000058769		18.Jun.13	10.0	mM	50.0	uL	DMSO
**NGP-105-03**	NCP003483	B07	CPD000466919	SAM001246907	46387019	CPD000466919	Pinacidil monohydrate	18.Jun.13	10.0	mM	50.0	uL	DMSO
**NGP-105-03**	NCP003483	C07	CPD000058266	SAM001246908	46387020	CPD000058266		18.Jun.13	10.0	mM	50.0	uL	DMSO
**NGP-105-03**	NCP003483	D07	CPD000112269	SAM001246909	46387021	CPD000112269		18.Jun.13	10.0	mM	50.0	uL	DMSO
**NGP-105-03**	NCP003483	E07	CPD000059045	SAM001246855	46386888	CPD000059045	92-84-2	18.Jun.13	10.0	mM	50.0	uL	DMSO
**NGP-105-03**	NCP003483	F07	CPD000058553	SAM001246526	46386544	CPD000058553		18.Jun.13	10.0	mM	50.0	uL	DMSO
**NGP-105-03**	NCP003483	G07	CPD000469138	SAM001246527	46386545	CPD000469138	Granisetron┬áHydrochloride	18.Jun.13	10.0	mM	50.0	uL	DMSO
**NGP-105-03**	NCP003483	H07	CPD000466293	SAM001247094	46387003	CPD000466293	Rimcazole	18.Jun.13	10,1	mM	50.0	uL	DMSO
**NGP-105-03**	NCP003483	A08	CPD000466292	SAM001247092	46387001	CPD000466292	Nafadotride	18.Jun.13	10.0	mM	50.0	uL	DMSO
**NGP-105-03**	NCP003483	B08	CPD000058856	SAM001246885	46386959	CPD000058856		18.Jun.13	10.0	mM	50.0	uL	DMSO
**NGP-105-03**	NCP003483	C08	CPD000471617	SAM001246557	46386575	CPD000471617	DEXCHLORPHENIRAMINE MALEATE	18.Jun.13	10.0	mM	50.0	uL	DMSO
**NGP-105-03**	NCP003483	D08	CPD000466288	SAM001247088	46386997	CPD000466288	Guanidine	18.Jun.13	9,9	mM	50.0	uL	DMSO
**NGP-105-03**	NCP003483	E08	CPD000466290	SAM001247090	46386999	CPD000466290	L-694,247	18.Jun.13	10.0	mM	50.0	uL	DMSO
**NGP-105-03**	NCP003483	F08	CPD000466284	SAM001247081	46386990	CPD000466284	AM-251	18.Jun.13	10.0	mM	50.0	uL	DMSO
**NGP-105-03**	NCP003483	G08	CPD000466289	SAM001247089	46386998	CPD000466289	HTMT	18.Jun.13	10.0	mM	50.0	uL	DMSO
**NGP-105-03**	NCP003483	H08	CPD000466286	SAM001247083	46386992	CPD000466286	Benzo[a]phenanthridine-10,11-diol, 5,6,6a,7,8,12b-hexahydro-, trans- [CAS]	18.Jun.13	10.0	mM	50.0	uL	DMSO
**NGP-105-03**	NCP003483	A09	CPD000466291	SAM001247091	46387000	CPD000466291	Methanesulfonamide, N-[4-[[1-[2-(6-methyl-2-pyridinyl)ethyl]-4-piperidinyl]carbonyl]phenyl]-, dihydrochloride [CAS]	18.Jun.13	9,9	mM	50.0	uL	DMSO
**NGP-105-03**	NCP003483	B09	CPD000466279	SAM001247076	46386985	CPD000466279	2H-Indol-2-one, 1,3-dihydro-1-phenyl-3,3-bis(4-pyridinylmethyl)-[CAS]	18.Jun.13	10.0	mM	50.0	uL	DMSO

Table S1. NIH Clinical collection

NCC PLATE	PLATE BARCODE	WELL ID	STRUCTURE ID	SAMPLE ID	PUBCHEM SID	STRUCTURE SYNONYMS [1]	STRUCTURE SYNONYMS [2]	DATE	CONC	CONC UNIT	VOLUME	VOLUME UNIT	SOLVENT
**NGP-105-03**	NCP003483	C09	CPD000466920	SAM001246898	46386972	CPD000466920	Beclomethasone	18.Jun.13	10.0	mM	50.0	uL	DMSO
**NGP-105-03**	NCP003483	D09	CPD000058847	SAM001246900	46386973	CPD000058847	73590-58-6	18.Jun.13	10.0	mM	50.0	uL	DMSO
**NGP-105-03**	NCP003483	E09	CPD000469228	SAM001246842	46386881	CPD000469228	DOLASETRON MESYLATE	18.Jun.13	10,4	mM	50.0	uL	DMSO
**NGP-105-03**	NCP003483	F09	CPD000449310	SAM001246841	46386880	CPD000449310	Zolmitriptan	18.Jun.13	10.0	mM	50.0	uL	DMSO
**NGP-105-03**	NCP003483	G09	CPD000469223	SAM001246852	46386886	CPD000469223	TREMULACIN	18.Jun.13	10.0	mM	50.0	uL	DMSO
**NGP-105-03**	NCP003483	H09	CPD000469227	SAM001246846	46386883	CPD000469227	DACTINOMYCIN	18.Jun.13	10.0	mM	50.0	uL	DMSO
**NGP-105-03**	NCP003483	A10	CPD000449308	SAM001246847	46386884	CPD000449308	Tramadol	18.Jun.13	10.0	mM	50.0	uL	DMSO
**NGP-105-03**	NCP003483	B10	CPD000469226	SAM001246815	46386831	CPD000469226	CHLORDIAZEPOXIDE	18.Jun.13	10.0	mM	50.0	uL	DMSO
**NGP-105-03**	NCP003483	C10	CPD000469225	SAM001246816	46386832	CPD000469225	CEFIXIME TRIHYDRATE	18.Jun.13	10.0	mM	50.0	uL	DMSO
**NGP-105-03**	NCP003483	D10	CPD000469224	SAM001246818	46386833	CPD000469224		18.Jun.13	10.0	mM	50.0	uL	DMSO
**NGP-105-03**	NCP003483	E10	CPD000469232	SAM001246820	46386875	CPD000469232	Lofexidine hydrochloride	18.Jun.13	10.0	mM	50.0	uL	DMSO
**NGP-105-03**	NCP003483	F10	CPD000469221	SAM001246804	46386828	CPD000469221	BALSALAZIDE	18.Jun.13	10.0	mM	50.0	uL	DMSO
**NGP-105-03**	NCP003483	G10	CPD000469220	SAM001246802	46386826	CPD000469220	OLOPATADINE HYDROCHLORIDE	18.Jun.13	10.0	mM	50.0	uL	DMSO
**NGP-105-03**	NCP003483	H10	CPD000469287	SAM001246803	46386827	CPD000469287	ITAVASTATIN Ca	18.Jun.13	10.0	mM	50.0	uL	DMSO
**NGP-105-03**	NCP003483	A11	CPD000058334	SAM001246882	46386956	CPD000058334		18.Jun.13	10.0	mM	50.0	uL	DMSO
**NGP-105-03**	NCP003483	B11	CPD000058431	SAM001246883	46386957	CPD000058431		18.Jun.13	10.0	mM	50.0	uL	DMSO
**NGP-105-03**	NCP003483	C11	CPD000469230	SAM001246822	46386877	CPD000469230	HOMOHARRINGTONINE	18.Jun.13	10.0	mM	50.0	uL	DMSO
**NGP-105-03**	NCP003483	D11	CPD000058318	SAM001246879	46386953	CPD000058318	50-22-6	18.Jun.13	10.0	mM	50.0	uL	DMSO
**NGP-105-03**	NCP003483	E11	CPD000471625	SAM001246821	46386876	CPD000471625	VECURONIUM BROMIDE	18.Jun.13	10.0	mM	50.0	uL	DMSO
**NGP-105-03**	NCP003483	F11	CPD000469219	SAM001246801	46386825	CPD000469219	TIBOLONE	18.Jun.13	10.0	mM	50.0	uL	DMSO
**NGP-105-03**	NCP003483	G11	CPD000058212	SAM001246860	46386893	CPD000058212	98-92-0	18.Jun.13	10.0	mM	50.0	uL	DMSO
**NGP-105-03**	NCP003483	H11	CPD000059131	SAM001246861	46386894	CPD000059131		18.Jun.13	10.0	mM	50.0	uL	DMSO
**NGP-105-04**	NCP003563	A02	CPD000058612	SAM001246886	46386960	CPD000058612		18.Jun.13	10.0	mM	50.0	uL	DMSO
**NGP-105-04**	NCP003563	B02	CPD000058726	SAM001246873	46386947	CPD000058726		18.Jun.13	10.0	mM	50.0	uL	DMSO
**NGP-105-04**	NCP003563	C02	CPD000058572	SAM001246914	46387026	CPD000058572	1,1-DIMETHYL-4-PHENYLPIPERAZINIUM IODIDE	18.Jun.13	10.0	mM	50.0	uL	DMSO
**NGP-105-04**	NCP003563	D02	CPD000058507	SAM001246893	46386967	CPD000058507		18.Jun.13	10.0	mM	50.0	uL	DMSO
**NGP-105-04**	NCP003563	E02	CPD000059128	SAM001246866	46386940	CPD000059128	72-33-3	18.Jun.13	10.0	mM	50.0	uL	DMSO
**NGP-105-04**	NCP003563	F02	CPD000059142	SAM001246868	46386942	CPD000059142	BENACTYZINE HYDROCHLORIDE	18.Jun.13	10.0	mM	50.0	uL	DMSO
**NGP-105-04**	NCP003563	G02	CPD000059100	SAM001246867	46386941	CPD000059100		18.Jun.13	10.0	mM	50.0	uL	DMSO
**NGP-105-04**	NCP003563	H02	CPD000059158	SAM001246870	46386944	CPD000059158	79-43-6	18.Jun.13	10.0	mM	50.0	uL	DMSO
**NGP-105-04**	NCP003563	A03	CPD000466283	SAM001247080	46386989	CPD000466283	Altanserin	18.Jun.13	10.0	mM	50.0	uL	DMSO
**NGP-105-04**	NCP003563	B03	CPD000466281	SAM001247078	46386987	CPD000466281	Acetamide, 2-amino-N-(1-methyl-1,2-diphenylethyl)-, (+/-)- [CAS]	18.Jun.13	10.0	mM	50.0	uL	DMSO

Table S1. NIH Clinical collection

NCC PLATE	PLATE BARCODE	WELL ID	STRUCTURE ID	SAMPLE ID	PUBCHEM SID	STRUCTURE SYNONYMS [1]	STRUCTURE SYNONYMS [2]	DATE	CONC	CONC UNIT	VOLUME	VOLUME UNIT	SOLVENT
**NGP-105-04**	NCP003563	C03	CPD000058420	SAM001247084	46386993	CPD000058420		18.Jun.13	10.0	mM	50.0	uL	DMSO
**NGP-105-04**	NCP003563	D03	CPD000466311	SAM001246563	46386581	CPD000466311		18.Jun.13	10.0	mM	50.0	uL	DMSO
**NGP-105-04**	NCP003563	E03	CPD000466285	SAM001247082	46386991	CPD000466285	Azasetron	18.Jun.13	10.0	mM	50.0	uL	DMSO
**NGP-105-04**	NCP003563	F03	CPD000466287	SAM001247087	46386996	CPD000466287	GR 89696	18.Jun.13	10.0	mM	50.0	uL	DMSO
**NGP-105-04**	NCP003563	G03	CPD000058773	SAM001246897	46386971	CPD000058773	DELTA1-HYDROCORTISONE 21-HEMISUCCINATE SODIUM SALT	18.Jun.13	10.0	mM	50.0	uL	DMSO
**NGP-105-04**	NCP003563	H03	CPD000058392	SAM001246872	46386946	CPD000058392		18.Jun.13	10.0	mM	50.0	uL	DMSO
**NGP-105-04**	NCP003563	A04	CPD000058366	SAM001246531	46386549	CPD000058366		18.Jun.13	10.0	mM	50.0	uL	DMSO
**NGP-105-04**	NCP003563	B04	CPD000469290	SAM001246578	46386596	CPD000469290	SAQUINAVIR MESYLATE	18.Jun.13	10.0	mM	50.0	uL	DMSO
**NGP-105-04**	NCP003563	C04	CPD000058970	SAM001246775	46386787	CPD000058970	60628-96-8	18.Jun.13	10.0	mM	50.0	uL	DMSO
**NGP-105-04**	NCP003563	D04	CPD000469158	SAM001246579	46386597	CPD000469158	SUMATRIPTAN SUCCINATE	18.Jun.13	10.0	mM	50.0	uL	DMSO
**NGP-105-04**	NCP003563	E04	CPD000466314	SAM001246574	46386592	CPD000466314	EXEMESTANE	18.Jun.13	10.0	mM	50.0	uL	DMSO
**NGP-105-04**	NCP003563	F04	CPD000466367	SAM001246708	46386689	CPD000466367	NITAZOXANIDE	18.Jun.13	10.0	mM	50.0	uL	DMSO
**NGP-105-04**	NCP003563	G04	CPD000058398	SAM001246536	46386554	CPD000058398		18.Jun.13	10.0	mM	50.0	uL	DMSO
**NGP-105-04**	NCP003563	H04	CPD000471623	SAM001246777	46386812	CPD000471623	QUETIAPINE HEMIFUMARATE	18.Jun.13	10.0	mM	50.0	uL	DMSO
**NGP-105-04**	NCP003563	A05	CPD000112560	SAM001246528	46386546	CPD000112560	RUTIN	18.Jun.13	10.0	mM	50.0	uL	DMSO
**NGP-105-04**	NCP003563	B05	CPD000466317	SAM001246580	46386598	CPD000466317	PENCICLOVIR	18.Jun.13	10.0	mM	50.0	uL	DMSO
**NGP-105-04**	NCP003563	C05	CPD000466393	SAM001246772	46386784	CPD000466393	CALCITRIOL	18.Jun.13	10.0	mM	50.0	uL	DMSO
**NGP-105-04**	NCP003563	D05	CPD000469140	SAM001246532	46386550	CPD000469140	DIPHENOXYLATE	18.Jun.13	10.0	mM	50.0	uL	DMSO
**NGP-105-04**	NCP003563	E05	CPD000449307	SAM001247005	46386855	CPD000449307	Felbamate	18.Jun.13	10.0	mM	50.0	uL	DMSO
**NGP-105-04**	NCP003563	F05	CPD000058855	SAM001247013	46386863	CPD000058855		18.Jun.13	10.0	mM	50.0	uL	DMSO
**NGP-105-04**	NCP003563	G05	CPD000035998	SAM001247011	46386861	CPD000035998		18.Jun.13	10.0	mM	50.0	uL	DMSO
**NGP-105-04**	NCP003563	H05	CPD000466277	SAM001247074	46386983	CPD000466277	1H-Imidazole-5-carboxylic acid, 1-(1-phenylethyl)-, ethyl ester, (R)- [CAS]	18.Jun.13	10,1	mM	50.0	uL	DMSO
**NGP-105-04**	NCP003563	A06	CPD000466395	SAM001246783	46386817	CPD000466395	RITONAVIR	18.Jun.13	10.0	mM	50.0	uL	DMSO
**NGP-105-04**	NCP003563	B06	CPD000469210	SAM001246780	46386815	CPD000469210	vinorelbine┬átartrate	18.Jun.13	10.0	mM	50.0	uL	DMSO
**NGP-105-04**	NCP003563	C06	CPD000466335	SAM001246624	46386641	CPD000466335	LINEZOLID	18.Jun.13	10.0	mM	50.0	uL	DMSO
**NGP-105-04**	NCP003563	D06	CPD000469203	SAM001246727	46386707	CPD000469203	LOMERIZINE DiHCl	18.Jun.13	10.0	mM	50.0	uL	DMSO
**NGP-105-04**	NCP003563	E06	CPD000466351	SAM001246667	46386724	CPD000466351	EFAVIRENZ	18.Jun.13	10,1	mM	50.0	uL	DMSO
**NGP-105-04**	NCP003563	F06	CPD000466306	SAM001246548	46386566	CPD000466306	IRBESARTAN	18.Jun.13	10,1	mM	50.0	uL	DMSO
**NGP-105-04**	NCP003563	G06	CPD000466305	SAM001246546	46386564	CPD000466305		18.Jun.13	10.0	mM	50.0	uL	DMSO
**NGP-105-04**	NCP003563	H06	CPD000238204	SAM001246555	46386573	CPD000238204		18.Jun.13	10.0	mM	50.0	uL	DMSO
**NGP-105-04**	NCP003563	A07	CPD000440694	SAM001246605	46386623	CPD000440694		18.Jun.13	10.0	mM	50.0	uL	DMSO
**NGP-105-04**	NCP003563	B07	CPD000469144	SAM001246547	46386565	CPD000469144	roxatidine acetate┬áhydrochloride	18.Jun.13	10.0	mM	50.0	uL	DMSO

Table S1. NIH Clinical collection

NCC PLATE	PLATE BARCODE	WELL ID	STRUCTURE ID	SAMPLE ID	PUBCHEM SID	STRUCTURE SYNONYMS [1]	STRUCTURE SYNONYMS [2]	DATE	CONC	CONC UNIT	VOLUME	VOLUME UNIT	SOLVENT
**NGP-105-04**	NCP003563	C07	CPD000471616	SAM001246556	46386574	CPD000471616	DEXBROMPHENIRAMINE MALEATE	18.Jun.13	10.0	mM	50.0	uL	DMSO
**NGP-105-04**	NCP003563	D07	CPD000469168	SAM001246604	46386622	CPD000469168	anagrelide hydrochloride	18.Jun.13	10.0	mM	50.0	uL	DMSO
**NGP-105-04**	NCP003563	E07	CPD000471618	SAM001246606	46386624	CPD000471618	TEGASEROD MALEATE	18.Jun.13	10.0	mM	50.0	uL	DMSO
**NGP-105-04**	NCP003563	F07	CPD000058475	SAM001246611	46386629	CPD000058475	MILRINONE	18.Jun.13	10,1	mM	50.0	uL	DMSO
**NGP-105-04**	NCP003563	G07	CPD000466315	SAM001246575	46386593	CPD000466315	LEVOCETIRIZINE	18.Jun.13	10.0	mM	50.0	uL	DMSO
**NGP-105-04**	NCP003563	H07	CPD000326936	SAM001246599	46386617	CPD000326936	Citalopram	18.Jun.13	10.0	mM	50.0	uL	DMSO
**NGP-105-04**	NCP003563	A08	CPD000048468	SAM001246558	46386576	CPD000048468	Ticlopidine Hydrochloride	18.Jun.13	10.0	mM	50.0	uL	DMSO
**NGP-105-04**	NCP003563	B08	CPD000469165	SAM001246594	46386612	CPD000469165	sodium┬áloxoprofen	18.Jun.13	10.0	mM	50.0	uL	DMSO
**NGP-105-04**	NCP003563	C08	CPD000466316	SAM001246577	46386595	CPD000466316	ZAFIRLUKAST	18.Jun.13	10.0	mM	50.0	uL	DMSO
**NGP-105-04**	NCP003563	D08	CPD000469152	SAM001246565	46386583	CPD000469152	Terbinafine hydrochloride	18.Jun.13	10.0	mM	50.0	uL	DMSO
**NGP-105-04**	NCP003563	E08	CPD000466320	SAM001246584	46386602	CPD000466320	ISRADIPINE	18.Jun.13	9,9	mM	50.0	uL	DMSO
**NGP-105-04**	NCP003563	F08	CPD000466318	SAM001246581	46386599	CPD000466318	VALSARTAN	18.Jun.13	10.0	mM	50.0	uL	DMSO
**NGP-105-04**	NCP003563	G08	CPD000449291	SAM001247048	46386918	CPD000449291	Piroxicam	18.Jun.13	10.0	mM	50.0	uL	DMSO
**NGP-105-04**	NCP003563	H08	CPD000469282	SAM001246629	46386646	CPD000469282		18.Jun.13	10.0	mM	50.0	uL	DMSO
**NGP-105-04**	NCP003563	A09	CPD000449286	SAM001246992	46386842	CPD000449286	Physostigmine	18.Jun.13	10.0	mM	50.0	uL	DMSO
**NGP-105-04**	NCP003563	B09	CPD000466278	SAM001247075	46386984	CPD000466278	1H-Indole-2-propanoic acid, 1-[(4-chlorophenyl)methyl]-3-[(1,1-dimethylethyl)thio]-Alpha,Alpha-dimethyl-5-(1-methylethyl)- [CAS]	18.Jun.13	10.0	mM	50.0	uL	DMSO
**NGP-105-04**	NCP003563	C09	CPD000058436	SAM001247030	46386900	CPD000058436	562-10-7	18.Jun.13	10.0	mM	50.0	uL	DMSO
**NGP-105-04**	NCP003563	D09	CPD000449266	SAM001247035	46386905	CPD000449266	Milnacipran	18.Jun.13	10.0	mM	50.0	uL	DMSO
**NGP-105-04**	NCP003563	E09	CPD000449315	SAM001247017	46386867	CPD000449315	5-fluoro-2-pyrimidone	18.Jun.13	10.0	mM	50.0	uL	DMSO
**NGP-105-04**	NCP003563	F09	CPD000466271	SAM001247022	46386872	CPD000466271	Chlorpheniramine	18.Jun.13	10.0	mM	50.0	uL	DMSO
**NGP-105-04**	NCP003563	G09	CPD000466333	SAM001246621	46386638	CPD000466333	DOFETILIDE	18.Jun.13	10.0	mM	50.0	uL	DMSO
**NGP-105-04**	NCP003563	H09	CPD000471620	SAM001246675	46386732	CPD000471620	FORMOTEROL FUMARATE DIHYDRATE	18.Jun.13	10.0	mM	50.0	uL	DMSO
**NGP-105-04**	NCP003563	A10	CPD000525252	SAM001246615	46386632	CPD000525252	RIZATRIPTAN BENZOATE	18.Jun.13	10.0	mM	50.0	uL	DMSO
**NGP-105-04**	NCP003563	B10	CPD000466332	SAM001246620	46386637	CPD000466332	RIFAPENTINE	18.Jun.13	10.0	mM	50.0	uL	DMSO
**NGP-105-04**	NCP003563	C10	CPD000469178	SAM001246630	46386647	CPD000469178	LOTEPREDNOL ETABONATE	18.Jun.13	10.0	mM	50.0	uL	DMSO
**NGP-105-04**	NCP003563	D10	CPD000466359	SAM001246684	46386741	CPD000466359	ENALAPRILAT	18.Jun.13	10.0	mM	50.0	uL	DMSO
**NGP-105-04**	NCP003563	E10	CPD000449292	SAM001246627	46386644	CPD000449292	Donepezil	18.Jun.13	10.0	mM	50.0	uL	DMSO
**NGP-105-04**	NCP003563	F10	CPD000238177	SAM001246755	46386768	CPD000238177		18.Jun.13	10.0	mM	50.0	uL	DMSO
**NGP-105-04**	NCP003563	G10	CPD000466365	SAM001246701	46386683	CPD000466365		18.Jun.13	10.0	mM	50.0	uL	DMSO
**NGP-105-04**	NCP003563	H10	CPD000466326	SAM001246602	46386620	CPD000466326		18.Jun.13	10.0	mM	50.0	uL	DMSO
**NGP-105-04**	NCP003563	A11	CPD000469143	SAM001246542	46386560	CPD000469143	ITOPRIDE HCl	18.Jun.13	10.0	mM	50.0	uL	DMSO
**NGP-105-04**	NCP003563	B11	CPD000466324	SAM001246597	46386615	CPD000466324	RIFAXIMIN	18.Jun.13	10.0	mM	50.0	uL	DMSO

Table S1. NIH Clinical collection

NCC PLATE	PLATE BARCODE	WELL ID	STRUCTURE ID	SAMPLE ID	PUBCHEM SID	STRUCTURE SYNONYMS [1]	STRUCTURE SYNONYMS [2]	DATE	CONC	CONC UNIT	VOLUME	VOLUME UNIT	SOLVENT
**NGP-105-04**	NCP003563	C11	CPD000469188	SAM001246657	46386714	CPD000469188	MONTELUKAST SODIUM	18.Jun.13	9,6	mM	50.0	uL	DMSO
**NGP-105-04**	NCP003563	D11	CPD000058253	SAM001246779	46386814	CPD000058253	2',3'-DIDEOXYCYTIDINE	18.Jun.13	10.0	mM	50.0	uL	DMSO
**NGP-105-04**	NCP003563	E11	CPD000466276	SAM001247073	46386982	CPD000466276	1H-Imidazol-2-amine, N-(2,6-dichlorophenyl)-4,5-dihydro- [CAS]	18.Jun.13	10.0	mM	50.0	uL	DMSO
**NGP-105-04**	NCP003563	F11	CPD000466280	SAM001247077	46386986	CPD000466280	6H-Pyrido[2,3-b][1,4]benzodiazepin-6-one, 11-[[2-[(diethylamino)methyl]-1-piperidinyl]acetyl]-5,11-dihydro- [CAS]	18.Jun.13	10.0	mM	50.0	uL	DMSO
**NGP-105-04**	NCP003563	G11	CPD000449316	SAM001246964	46386791	CPD000449316	3'-deoxydenosine	18.Jun.13	10.0	mM	50.0	uL	DMSO
**NGP-105-04**	NCP003563	H11	CPD000449296	SAM001246980	46386807	CPD000449296	Ifenprodil	18.Jun.13	10.0	mM	50.0	uL	DMSO
**NGP-105-05**	NCP003643	A02	CPD000145728	SAM001247020	46386870	CPD000145728	5-Amino-2-hydroxy-benzoic acid	18.Jun.13	10.0	mM	50.0	uL	DMSO
**NGP-105-05**	NCP003643	B02	CPD000466269	SAM001246991	46386841	CPD000466269	Paroxetine	18.Jun.13	10.0	mM	50.0	uL	DMSO
**NGP-105-05**	NCP003643	C02	CPD000058465	SAM001247050	46386920	CPD000058465	LOBELINE HYDROCHLORIDE	18.Jun.13	10.0	mM	50.0	uL	DMSO
**NGP-105-05**	NCP003643	D02	CPD000449329	SAM001247049	46386919	CPD000449329	L-Ornithine, N5-[imino(methylamino)methyl]-[CAS]	18.Jun.13	10.0	mM	50.0	uL	DMSO
**NGP-105-05**	NCP003643	E02	CPD000058461	SAM001246596	46386614	CPD000058461		18.Jun.13	10.0	mM	50.0	uL	DMSO
**NGP-105-05**	NCP003643	F02	CPD000449321	SAM001247025	46386895	CPD000449321	Oxiranecarboxylic acid, 2-[6-(4-chlorophenoxy)hexyl]-, ethyl ester- [CAS]	18.Jun.13	10.0	mM	50.0	uL	DMSO
**NGP-105-05**	NCP003643	G02	CPD000449288	SAM001247031	46386901	CPD000449288	Epigallocatechin gallate	18.Jun.13	10,3	mM	50.0	uL	DMSO
**NGP-105-05**	NCP003643	H02	CPD000449275	SAM001247072	46386981	CPD000449275	Raclopride	18.Jun.13	10.0	mM	50.0	uL	DMSO
**NGP-105-05**	NCP003643	A03	CPD000449271	SAM001247069	46386978	CPD000449271	Zacopride	18.Jun.13	10.0	mM	50.0	uL	DMSO
**NGP-105-05**	NCP003643	B03	CPD000449276	SAM001247068	46386977	CPD000449276	SKF 83566	18.Jun.13	10.0	mM	50.0	uL	DMSO
**NGP-105-05**	NCP003643	C03	CPD000449274	SAM001246965	46386792	CPD000449274	AM 404	18.Jun.13	10.0	mM	50.0	uL	DMSO
**NGP-105-05**	NCP003643	D03	CPD000449281	SAM001247063	46386933	CPD000449281	Nalbuphine	18.Jun.13	10.0	mM	50.0	uL	DMSO
**NGP-105-05**	NCP003643	E03	CPD000059053	SAM001246962	46386789	CPD000059053	PILOCARPINE HYDROCHLORIDE	18.Jun.13	10.0	mM	50.0	uL	DMSO
**NGP-105-05**	NCP003643	F03	CPD000058291	SAM001246963	46386790	CPD000058291		18.Jun.13	10.0	mM	50.0	uL	DMSO
**NGP-105-05**	NCP003643	G03	CPD000042823	SAM001246961	46386788	CPD000042823	Flurbiprofen	18.Jun.13	10.0	mM	50.0	uL	DMSO
**NGP-105-05**	NCP003643	H03	CPD000059136	SAM001247015	46386865	CPD000059136	3-HYDROXY-1,2-DIMETHYL-4(1H)-PYRIDONE	18.Jun.13	10.0	mM	50.0	uL	DMSO
**NGP-105-05**	NCP003643	A04	CPD000058470	SAM001247061	46386931	CPD000058470	Loxapine	18.Jun.13	10.0	mM	50.0	uL	DMSO

Table S1. NIH Clinical collection

NCC PLATE	PLATE BARCODE	WELL ID	STRUCTURE ID	SAMPLE ID	PUBCHEM SID	STRUCTURE SYNONYMS [1]	STRUCTURE SYNONYMS [2]	DATE	CONC	CONC UNIT	VOLUME	VOLUME UNIT	SOLVENT
**NGP-105-05**	NCP003643	B04	CPD000326694	SAM001247062	46386932	CPD000326694	d-3-Methoxy-N-methylmorphinan hydrobromide	18.Jun.13	10.0	mM	50.0	uL	DMSO
**NGP-105-05**	NCP003643	C04	CPD000449282	SAM001247059	46386929	CPD000449282	Duloxetine	18.Jun.13	10.0	mM	50.0	uL	DMSO
**NGP-105-05**	NCP003643	D04	CPD000449320	SAM001247060	46386930	CPD000449320	Glycine, N-[2-[(acetylthio)methyl]-1-oxo-3-phenylpropyl]-,phenylmethyl ester [CAS]	18.Jun.13	10.0	mM	50.0	uL	DMSO
**NGP-105-05**	NCP003643	E04	CPD000449318	SAM001247057	46386927	CPD000449318	Benzeneacetic acid, 2-[(2,6-dichlorophenyl)amino]-, monosodium salt [CAS]	18.Jun.13	10.0	mM	50.0	uL	DMSO
**NGP-105-05**	NCP003643	F04	CPD000058345	SAM001247039	46386909	CPD000058345		18.Jun.13	10.0	mM	50.0	uL	DMSO
**NGP-105-05**	NCP003643	G04	CPD000058961	SAM001247033	46386903	CPD000058961	FAMOTIDINE	18.Jun.13	10,2	mM	50.0	uL	DMSO
**NGP-105-05**	NCP003643	H04	CPD000449299	SAM001246999	46386849	CPD000449299	SR 57227A	18.Jun.13	10.0	mM	50.0	uL	DMSO
**NGP-105-05**	NCP003643	A05	CPD000466270	SAM001247003	46386853	CPD000466270	Pancuronium	18.Jun.13	10.0	mM	50.0	uL	DMSO
**NGP-105-05**	NCP003643	B05	CPD000058175	SAM001247010	46386860	CPD000058175	443-48-1	18.Jun.13	10.0	mM	50.0	uL	DMSO
**NGP-105-05**	NCP003643	C05	CPD000449327	SAM001246967	46386794	CPD000449327	Benzeneacetic acid, Alpha-(hydroxymethyl)-, 9-methyl-3-oxa-9-azatricyclo[3.3.1.02,4]non-7-yl ester, [7(S)-(1Alpha,2 ,4 ,5Alpha,7)]- [CAS]	18.Jun.13	10.0	mM	50.0	uL	DMSO
**NGP-105-05**	NCP003643	D05	CPD000449323	SAM001246968	46386795	CPD000449323	Benzeneacetonitrile, Alpha-[3-[[2-(3,4-dimethoxyphenyl)ethyl]methylamino]propyl]-3,4-dimethoxy-Alpha-(1-methylethyl)-, (R)- [CAS]	18.Jun.13	10.0	mM	50.0	uL	DMSO
**NGP-105-05**	NCP003643	E05	CPD000449328	SAM001246969	46386796	CPD000449328		18.Jun.13	10.0	mM	50.0	uL	DMSO
**NGP-105-05**	NCP003643	F05	CPD000449294	SAM001246970	46386797	CPD000449294	zucapsaicin	18.Jun.13	10.0	mM	50.0	uL	DMSO
**NGP-105-05**	NCP003643	G05	CPD000058513	SAM001246971	46386798	CPD000058513	SALBUTAMOL SULFATE	18.Jun.13	10.0	mM	50.0	uL	DMSO
**NGP-105-05**	NCP003643	H05	CPD000057879	SAM001246972	46386799	CPD000057879	(+/-)-Vesamicol hydrochloride	18.Jun.13	10.0	mM	50.0	uL	DMSO
**NGP-105-05**	NCP003643	A06	CPD000469289	SAM001246993	46386843	CPD000469289	Picrotin - Picrotoxinin	18.Jun.13	10.0	mM	50.0	uL	DMSO
**NGP-105-05**	NCP003643	B06	CPD000449268	SAM001247000	46386850	CPD000449268	Terazosin	18.Jun.13	10.0	mM	50.0	uL	DMSO
**NGP-105-05**	NCP003643	C06	CPD000449319	SAM001247027	46386897	CPD000449319	diphenylcyclopropenone	18.Jun.13	10.0	mM	50.0	uL	DMSO
**NGP-105-05**	NCP003643	D06	CPD000449326	SAM001247016	46386866	CPD000449326	4-Thiazolidinecarboxylic acid, 2-oxo-, (R)- [CAS]	18.Jun.13	10.0	mM	50.0	uL	DMSO
**NGP-105-05**	NCP003643	E06	CPD000466274	SAM001247051	46386921	CPD000466274	Mesoridazine	18.Jun.13	10.0	mM	50.0	uL	DMSO

Table S1. NIH Clinical collection

NCC	PLATE	PLATE BARCODE	WELL ID	STRUCTURE ID	SAMPLE ID	PUBCHEM SID	STRUCTURE SYNONYMS [1]	STRUCTURE SYNONYMS [2]	DATE	CONC	CONC UNIT	VOLUME	VOLUME UNIT	SOLVENT
**NGP-105-05**		NCP003643	F06	CPD000449313	SAM001247053	46386923	CPD000449313	3(2H)-Pyridazinone, 6-[4-(difluoromethoxy)-3-methoxyphenyl]- [CAS]	18.Jun.13	10.0	mM	50.0	uL	DMSO
**NGP-105-05**		NCP003643	G06	CPD000466275	SAM001247054	46386924	CPD000466275	10H-Phenothiazine, 2-chloro-10-[3-(4-methyl-1-piperazinyl)propyl- [CAS]	18.Jun.13	10.0	mM	50.0	uL	DMSO
**NGP-105-05**		NCP003643	H06	CPD000449322	SAM001247055	46386925	CPD000449322	1H-Cyclopenta[b]quinolin-9-amine, 2,3,5,6,7,8-hexahydro-, monohydrochloride- [CAS]	18.Jun.13	10.0	mM	50.0	uL	DMSO
**NGP-105-05**		NCP003643	A07	CPD000058306	SAM001247056	46386926	CPD000058306	CLOTRIMAZOLE	18.Jun.13	10.0	mM	50.0	uL	DMSO
**NGP-105-05**		NCP003643	B07	CPD000058255	SAM001246987	46386837	CPD000058255	79794-75-5	18.Jun.13	10.0	mM	50.0	uL	DMSO
**NGP-105-05**		NCP003643	C07	CPD000058500	SAM001247037	46386907	CPD000058500	Phenelzine sulfate	18.Jun.13	10.0	mM	50.0	uL	DMSO
**NGP-105-05**		NCP003643	D07	CPD000449311	SAM001246997	46386847	CPD000449311	Riluzole	18.Jun.13	10.0	mM	50.0	uL	DMSO
**NGP-105-05**		NCP003643	E07	CPD000449312	SAM001247004	46386854	CPD000449312	Naltrindole	18.Jun.13	10.0	mM	50.0	uL	DMSO
**NGP-105-05**		NCP003643	F07	CPD000449277	SAM001247026	46386896	CPD000449277	Nornicotine	18.Jun.13	10.0	mM	50.0	uL	DMSO
**NGP-105-05**		NCP003643	G07	CPD000449269	SAM001247052	46386922	CPD000449269	Bifemelane	18.Jun.13	10.0	mM	50.0	uL	DMSO
**NGP-105-05**		NCP003643	H07	CPD000449284	SAM001246973	46386800	CPD000449284	CGS 15943	18.Jun.13	10.0	mM	50.0	uL	DMSO
**NGP-105-05**		NCP003643	A08	CPD000449287	SAM001246974	46386801	CPD000449287	Cinanserin	18.Jun.13	10.0	mM	50.0	uL	DMSO
**NGP-105-05**		NCP003643	B08	CPD000449272	SAM001246975	46386802	CPD000449272	Cisapride	18.Jun.13	10.0	mM	50.0	uL	DMSO
**NGP-105-05**		NCP003643	C08	CPD000449273	SAM001246981	46386808	CPD000449273	Indatraline	18.Jun.13	10.0	mM	50.0	uL	DMSO
**NGP-105-05**		NCP003643	D08	CPD000058520	SAM001247045	46386915	CPD000058520	25332-39-2	18.Jun.13	10.0	mM	50.0	uL	DMSO
**NGP-105-05**		NCP003643	E08	CPD000449301	SAM001246995	46386845	CPD000449301	Prazosin	18.Jun.13	10.0	mM	50.0	uL	DMSO
**NGP-105-05**		NCP003643	F08	CPD000058525	SAM001247001	46386851	CPD000058525	URAPIDIL HYDROCHLORIDE	18.Jun.13	10.0	mM	50.0	uL	DMSO
**NGP-105-05**		NCP003643	G08	CPD000449278	SAM001247007	46386857	CPD000449278	(-)-Cotinine	18.Jun.13	10.0	mM	50.0	uL	DMSO
**NGP-105-05**		NCP003643	H08	CPD000058313	SAM001247014	46386864	CPD000058313	D-CYCLOSERINE	18.Jun.13	10.0	mM	50.0	uL	DMSO
**NGP-105-05**		NCP003643	A09	CPD000466268	SAM001246977	46386804	CPD000466268	Fluvoxamine	18.Jun.13	10.0	mM	50.0	uL	DMSO
**NGP-105-05**		NCP003643	B09	CPD000449270	SAM001246976	46386803	CPD000449270	Doxepin	18.Jun.13	10.0	mM	50.0	uL	DMSO
**NGP-105-05**		NCP003643	C09	CPD000059133	SAM001247046	46386916	CPD000059133		18.Jun.13	10.0	mM	50.0	uL	DMSO
**NGP-105-05**		NCP003643	D09	CPD000058908	SAM001247023	46386873	CPD000058908	(+)-3-HYDROXY-N-METHYLMORPHINAN D-TARTRATE	18.Jun.13	9,9	mM	50.0	uL	DMSO
**NGP-105-05**		NCP003643	E09	CPD000058555	SAM001246988	46386838	CPD000058555	LY 171883	18.Jun.13	10.0	mM	50.0	uL	DMSO
**NGP-105-05**		NCP003643	F09	CPD000148117	SAM001246989	46386839	CPD000148117	Maprotiline hydrochloride	18.Jun.13	10.0	mM	50.0	uL	DMSO
**NGP-105-05**		NCP003643	G09	CPD000466272	SAM001247038	46386908	CPD000466272	Pizotyline	18.Jun.13	10.0	mM	50.0	uL	DMSO
**NGP-105-05**		NCP003643	H09	CPD000059126	SAM001247032	46386902	CPD000059126	BETA-ESTRADIOL	18.Jun.13	10.0	mM	50.0	uL	DMSO
**NGP-105-05**		NCP003643	A10	CPD000059046	SAM001247019	46386869	CPD000059046	N,N'-DIACETYL-1,6-DIAMINOHEXANE	18.Jun.13	10.0	mM	50.0	uL	DMSO
**NGP-105-05**		NCP003643	B10	CPD000058353	SAM001247024	46386874	CPD000058353	147-24-0	18.Jun.13	10.0	mM	50.0	uL	DMSO
**NGP-105-05**		NCP003643	C10	CPD000449267	SAM001246978	46386805	CPD000449267	Galanthamine	18.Jun.13	10.0	mM	50.0	uL	DMSO

Table S1. NIH Clinical collection

NCC PLATE	PLATE BARCODE	WELL ID	STRUCTURE ID	SAMPLE ID	PUBCHEM SID	STRUCTURE SYNONYMS [1]	STRUCTURE SYNONYMS [2]	DATE	CONC	CONC UNIT	VOLUME	VOLUME UNIT	SOLVENT
**NGP-105-05**	NCP003643	D10	CPD000449290	SAM001246982	46386809	CPD000449290	Indomethacin	18.Jun.13	10.0	mM	50.0	uL	DMSO
**NGP-105-05**	NCP003643	E10	CPD000059171	SAM001247028	46386898	CPD000059171	TETRAETHYLTHIURAM DISULFIDE	18.Jun.13	10.0	mM	50.0	uL	DMSO
**NGP-105-05**	NCP003643	F10	CPD000449302	SAM001246994	46386844	CPD000449302	Piribedil	18.Jun.13	10.0	mM	50.0	uL	DMSO
**NGP-105-05**	NCP003643	G10	CPD000058460	SAM001246983	46386810	CPD000058460		18.Jun.13	10.0	mM	50.0	uL	DMSO
**NGP-105-05**	NCP003643	H10	CPD000058623	SAM001247047	46386917	CPD000058623		18.Jun.13	10.0	mM	50.0	uL	DMSO
**NGP-105-05**	NCP003643	A11	CPD000449325	SAM001246996	46386846	CPD000449325	Pyrazinecarboxamide, 3,5-diamino-N-(aminoiminomethyl)-6-chloro-[CAS]	18.Jun.13	10.0	mM	50.0	uL	DMSO
**NGP-105-05**	NCP003643	B11	CPD000059105	SAM001247002	46386852	CPD000059105	9-AMINO-1,2,3,4-TETRAHYDROACRIDINE HYDROCHLORIDE	18.Jun.13	10.0	mM	50.0	uL	DMSO
**NGP-105-05**	NCP003643	C11	CPD000058319	SAM001247008	46386858	CPD000058319	ETHYNYLESTRADIOL	18.Jun.13	10.0	mM	50.0	uL	DMSO
**NGP-105-05**	NCP003643	D11	CPD000449317	SAM001247012	46386862	CPD000449317	2(1H)-Pyrimidinone, 4-amino-1-y-D-arabinofuranosyl- [CAS]	18.Jun.13	10.0	mM	50.0	uL	DMSO
**NGP-105-05**	NCP003643	E11	CPD000449324	SAM001246985	46386835	CPD000449324	L-Glutamic acid, N-[4-[[(2,4-diamino-6-pteridinyl)methyl]methylamino]benzoyl]- [CAS]	18.Jun.13	10.0	mM	50.0	uL	DMSO
**NGP-105-05**	NCP003643	F11	CPD000449305	SAM001247043	46386913	CPD000449305	TFMPP	18.Jun.13	10.0	mM	50.0	uL	DMSO
**NGP-105-05**	NCP003643	G11	CPD000449298	SAM001247006	46386856	CPD000449298	Pramipexole	18.Jun.13	10.0	mM	50.0	uL	DMSO
**NGP-105-05**	NCP003643	H11	CPD000058189	SAM001247018	46386868	CPD000058189		18.Jun.13	10.0	mM	50.0	uL	DMSO
**NGP-105-06**	NCP003723	A02	CPD000466297	SAM001247099	46387008	CPD000466297	SDM25N	18.Jun.13	10,1	mM	50.0	uL	DMSO
**NGP-105-06**	NCP003723	B02	CPD000466300	SAM001247103	46387012	CPD000466300	5-Nonyloxytryptamine	18.Jun.13	9,9	mM	50.0	uL	DMSO
**NGP-105-06**	NCP003723	C02	CPD000466296	SAM001247097	46387006	CPD000466296	SB 205607	18.Jun.13	9,8	mM	50.0	uL	DMSO
**NGP-105-06**	NCP003723	D02	CPD000058344	SAM001246921	46501387	CPD000058344		18.Jun.13	10.0	mM	50.0	uL	DMSO
**NGP-105-06**	NCP003723	E02	CPD000238180	SAM001246641	46386657	CPD000238180		18.Jun.13	10.0	mM	50.0	uL	DMSO
**NGP-105-06**	NCP003723	F02	CPD000468734	SAM001247067	46386976	CPD000468734	PD 81723	18.Jun.13	10.0	mM	50.0	uL	DMSO
**NGP-105-06**	NCP003723	G02	CPD000469222	SAM001246805	46386829	CPD000469222		18.Jun.13	10.0	mM	50.0	uL	DMSO
**NGP-105-06**	NCP003723	H02	CPD000058445	SAM001247071	46386980	CPD000058445		18.Jun.13	10.0	mM	50.0	uL	DMSO
**NGP-105-06**	NCP003723	A03	CPD000466299	SAM001247101	46387010	CPD000466299	Thiophene, 5-bromo-2-(4-fluorophenyl)-3-[4-(methylsulfonyl)phenyl]- [CAS]	18.Jun.13	10.0	mM	50.0	uL	DMSO
**NGP-105-06**	NCP003723	B03	CPD000466295	SAM001247096	46387005	CPD000466295	Salmeterol	18.Jun.13	10.0	mM	50.0	uL	DMSO
**NGP-105-06**	NCP003723	C03	CPD000326935	SAM001247098	46387007	CPD000326935	R(+)-SCH-23390 hydrochloride	18.Jun.13	9,9	mM	50.0	uL	DMSO
**NGP-105-06**	NCP003723	D03	CPD000059075	SAM001246922	46387028	CPD000059075	DEHYDROEPIANDROSTERONE	18.Jun.13	10.0	mM	50.0	uL	DMSO

Table S1. NIH Clinical collection

NCC PLATE	PLATE BARCODE	WELL ID	STRUCTURE ID	SAMPLE ID	PUBCHEM SID	STRUCTURE SYNONYMS [1]	STRUCTURE SYNONYMS [2]	DATE	CONC	CONC UNIT	VOLUME	VOLUME UNIT	SOLVENT
**NGP-105-06**	NCP003723	E03	CPD000112594	SAM001246840	46386879	CPD000112594	Prostaglandin E1	18.Jun.13	10.0	mM	50.0	uL	DMSO
**NGP-105-06**	NCP003723	F03	CPD000058878	SAM001246590	46386608	CPD000058878		18.Jun.13	10.0	mM	50.0	uL	DMSO
**NGP-105-06**	NCP003723	G03	CPD000468732	SAM001247065	46386935	CPD000468732	CCPA	18.Jun.13	10.0	mM	50.0	uL	DMSO
**NGP-105-06**	NCP003723	H03	CPD000468733	SAM001247066	46386975	CPD000468733	CGS 12066B	18.Jun.13	10.0	mM	50.0	uL	DMSO
**NGP-105-06**	NCP003723	A04	CPD000469153	SAM001246568	46386586	CPD000469153	VINDESINE SULFATE	18.Jun.13	9,7	mM	50.0	uL	DMSO
**NGP-105-06**	NCP003723	B04	CPD000058540	SAM001246570	46386588	CPD000058540	VINCRISTINE SULFATE	18.Jun.13	10.0	mM	50.0	uL	DMSO
**NGP-105-06**	NCP003723	C04	CPD000466342	SAM001246648	46386664	CPD000466342	LACIDIPINE	18.Jun.13	10.0	mM	50.0	uL	DMSO
**NGP-105-06**	NCP003723	D04	CPD000466347	SAM001246659	46386716	CPD000466347		18.Jun.13	10.0	mM	50.0	uL	DMSO
**NGP-105-06**	NCP003723	E04	CPD000469285	SAM001246707	46386688	CPD000469285	AMPIROXICAM	18.Jun.13	10.0	mM	50.0	uL	DMSO
**NGP-105-06**	NCP003723	F04	CPD000466368	SAM001246710	46386690	CPD000466368	GLIMEPIRIDE	18.Jun.13	10.0	mM	50.0	uL	DMSO
**NGP-105-06**	NCP003723	G04	CPD000469198	SAM001246705	46386686	CPD000469198	Amlodipine	18.Jun.13	10.0	mM	50.0	uL	DMSO
**NGP-105-06**	NCP003723	H04	CPD000469174	SAM001246619	46386636	CPD000469174	RABEPRAZOLE	18.Jun.13	10.0	mM	50.0	uL	DMSO
**NGP-105-06**	NCP003723	A05	CPD000058704	SAM001246878	46386952	CPD000058704	CLOFAZIMINE	18.Jun.13	10.0	mM	50.0	uL	DMSO
**NGP-105-06**	NCP003723	B05	CPD000469166	SAM001246598	46386616	CPD000469166	Irinotecan hydrochloride	18.Jun.13	10.0	mM	50.0	uL	DMSO
**NGP-105-06**	NCP003723	C05	CPD000058469	SAM001246544	46386562	CPD000058469	103577-45-3	18.Jun.13	10,1	mM	50.0	uL	DMSO
**NGP-105-06**	NCP003723	D05	CPD000149358	SAM001246545	46386563	CPD000149358	8-Chloro-11-piperidin-4-ylidene-6,11-dihydro-5H-benzo[5,6]cyclohepta[1,2-b]pyridine	18.Jun.13	10.0	mM	50.0	uL	DMSO
**NGP-105-06**	NCP003723	E05	CPD000058772	SAM001246904	46386974	CPD000058772	1,3,5(10)-ESTRATRIEN-3-OL-17-ONE SULPHATE, SODIUM SALT	18.Jun.13	10.0	mM	50.0	uL	DMSO
**NGP-105-06**	NCP003723	F05	CPD000058481	SAM001246881	46386955	CPD000058481		18.Jun.13	10.0	mM	50.0	uL	DMSO
**NGP-105-06**	NCP003723	G05	CPD000112002	SAM001246880	46386954	CPD000112002		18.Jun.13	10.0	mM	50.0	uL	DMSO
**NGP-105-06**	NCP003723	H05	CPD000238156	SAM001247105	46387014	CPD000238156	Sibutramine	18.Jun.13	10.0	mM	50.0	uL	DMSO
**NGP-105-06**	NCP003723	A06	CPD000469632	SAM001247107	46387016	CPD000469632		18.Jun.13	10.0	mM	50.0	uL	DMSO
**NGP-105-06**	NCP003723	B06	CPD000469231	SAM001246851	46386885	CPD000469231		18.Jun.13	10.0	mM	50.0	uL	DMSO
**NGP-105-06**	NCP003723	C06	CPD000472527	SAM001246592	46386610	CPD000472527	Sibutramine hydrochloride	18.Jun.13	10.0	mM	50.0	uL	DMSO
**NGP-105-06**	NCP003723	D06	CPD000058410	SAM001246833	46386878	CPD000058410		18.Jun.13	10.0	mM	50.0	uL	DMSO
**NGP-105-06**	NCP003723	E06	CPD000469633	SAM001247108	46387017	CPD000469633	8-Azaspiro[4.5]decane-7,9-dione, 8-[2-[[(2,3-dihydro-1,4-benzodioxin-2-yl)methyl]amino]ethyl]-, monomethanesulfonate [CAS]	18.Jun.13	10.0	mM	50.0	uL	DMSO
**NGP-105-06**	NCP003723	F06	CPD000469631	SAM001247106	46387015	CPD000469631	Adenosine, N-(2-hydroxycyclopentyl)-, (1S-trans)- [CAS]	18.Jun.13	10.0	mM	50.0	uL	DMSO
**NGP-105-06**	NCP003723	G06	CPD000058296	SAM001246646	46386662	CPD000058296	19774-82-4	18.Jun.13	9,5	mM	50.0	uL	DMSO
**NGP-105-06**	NCP003723	H06	CPD000336944	SAM001246644	46386660	CPD000336944		18.Jun.13	10.0	mM	50.0	uL	DMSO
**NGP-105-06**	NCP003723	A07	CPD000469175	SAM001246622	46386639	CPD000469175	IMATINIB MESYLATE	18.Jun.13	10.0	mM	50.0	uL	DMSO
**NGP-105-06**	NCP003723	B07	CPD000468736	SAM001247102	46387011	CPD000468736	Metylperon	18.Jun.13	10.0	mM	50.0	uL	DMSO
**NGP-105-06**	NCP003723	C07	CPD000469594	SAM001246773	46386785	CPD000469594	Parecoxib sodium	18.Jun.13	9,8	mM	50.0	uL	DMSO
**NGP-105-06**	NCP003723	D07	CPD000058504	SAM001247070	46386979	CPD000058504		18.Jun.13	10.0	mM	50.0	uL	DMSO

Table S1. NIH Clinical collection

NCC PLATE	PLATE BARCODE	WELL ID	STRUCTURE ID	SAMPLE ID	PUBCHEM SID	STRUCTURE SYNONYMS [1]	STRUCTURE SYNONYMS [2]	DATE	CONC	CONC UNIT	VOLUME	VOLUME UNIT	SOLVENT
**NGP-105-06**	NCP003723	E07	CPD000471626	SAM001246793	46386822	CPD000471626	ATRACURIUM BESYLATE	18.Jun.13	10.0	mM	50.0	uL	DMSO
**NGP-105-06**	NCP003723	F07	CPD000469218	SAM001246799	46386824	CPD000469218	ARTEMETHER	18.Jun.13	10.0	mM	50.0	uL	DMSO

Table S1. NIH Clinical collection

NCC PLATE	PLATE BARCODE	WELL ID	STRUCTURE REAL MF	STRUCTURE REAL AMW	PARENT AMW	PARENT EMW	SAMPLE SUPPLIER	SUPPLIER STRUCTURE ID	NUM H DONORS	NUM H ACCEPTORS
**NGP-105-01**	NCP003323	A02	C13H18ClNO.HCl	2.762.070	2.397.461	2.391.076	Sequoia Research Products Ltd.	SRP03446b	1	2
**NGP-105-01**	NCP003323	B02	C9H7Cl2N5.C4H4O4	3.721.665	2.560.945	2.550.078	Sequoia Research Products Ltd.	SRP01510i	4	5
**NGP-105-01**	NCP003323	C02	C25H43NO18	6.456.149	6.456.149	6.452.480	Sequoia Research Products Ltd.	SRP00375a	14	19
**NGP-105-01**	NCP003323	D02	C21H27NO.H3P04	4.074.485	3.094.531	3.092.092	Sequoia Research Products Ltd.	SRP01065b	0	2
**NGP-105-01**	NCP003323	E02	C10H13NO2	1.792.195	1.792.195	1.790.946	Sequoia Research Products Ltd.	SRP014251p	2	3
**NGP-105-01**	NCP003323	F02	C12H21N.HCl	2.157.676	1.793.067	1.791.673	Sequoia Research Products Ltd.	SRP02040m	2	1
**NGP-105-01**	NCP003323	G02	C24H26N2O4	4.064.830	4.064.830	4.061.892	Sequoia Research Products Ltd.	SRP01625c	3	6
**NGP-105-01**	NCP003323	H02	C13H18N4O3	2.783.120	2.783.120	2.781.378	Sequoia Research Products Ltd.	SRP01571l	0	7
**NGP-105-01**	NCP003323	A03	C16H15FN2O4	3.183.054	3.183.054	3.181.015	Sequoia Research Products Ltd.	SRP010775p	3	6
**NGP-105-01**	NCP003323	B03	C8H17NO5	2.072.277	2.072.277	2.071.106	Sequoia Research Products Ltd.	SRP06560m	5	6
**NGP-105-01**	NCP003323	C03	C18H17NO5	3.273.377	3.273.377	3.271.106	Sequoia Research Products Ltd.	SRP02572t	2	6
**NGP-105-01**	NCP003323	D03	C17H20N4S	3.124.338	3.124.338	3.121.408	Sequoia Research Products Ltd.	SRP01035o	1	4
**NGP-105-01**	NCP003323	E03	C25H32ClN5O2.HCl	5.064.772	4.700.163	4.692.244	Sequoia Research Products Ltd.	SRP03327n	0	7
**NGP-105-01**	NCP003323	F03	C21H24FN3O4.HCl	4.379.000	4.014.391	4.011.750	Sequoia Research Products Ltd.	SRP06644m	2	7
**NGP-105-01**	NCP003323	G03	C32H45N3O4S.CH4O3S	6.638.954	5.677.897	5.673.130	Sequoia Research Products Ltd.	SRP03330n	4	7
**NGP-105-01**	NCP003323	H03	C23H35O7.Na+	4.465.186	4.235.288	4.232.382	Sequoia Research Products Ltd.	SRP02590p	3	7
**NGP-105-01**	NCP003323	A04	C23H23N3O5.HCl	4.579.150	4.214.541	4.211.637	Sequoia Research Products Ltd.	SRP02510t	2	8
**NGP-105-01**	NCP003323	B04	C8H14N2O2	1.702.122	1.702.122	1.701.055	Sequoia Research Products Ltd.	SRP01381l	2	4
**NGP-105-01**	NCP003323	C04	C10H17N3S.HCl	2.477.870	2.113.261	2.111.143	Sequoia Research Products Ltd.	SRP02587p	3	3
**NGP-105-01**	NCP003323	D04	C23H27FN4O2	4.104.930	4.104.930	4.102.118	Sequoia Research Products Ltd.	SRP01295r	0	6
**NGP-105-01**	NCP003323	E04	C19H20N2O3S.HCl	3.929.015	3.564.406	3.561.194	Sequoia Research Products Ltd.	SRP02425p	1	5
**NGP-105-01**	NCP003323	F04	C16H25N2O5S.Na+	3.804.362	3.574.464	3.571.484	Sequoia Research Products Ltd.	SRP035055c	4	7
**NGP-105-01**	NCP003323	G04	C23H36N6O5S	5.086.382	5.086.382	5.082.467	Sequoia Research Products Ltd.	SRP07170a	7	11
**NGP-105-01**	NCP003323	H04	C16H14N2O3S	3.143.596	3.143.596	3.140.725	Sequoia Research Products Ltd.	SRP00825v	2	5
**NGP-105-01**	NCP003323	A05	C24H28N2O3	3.924.996	3.924.996	3.922.099	Sequoia Research Products Ltd.	SRP00825n	1	5
**NGP-105-01**	NCP003323	B05	C21H22O8	4.024.022	4.024.022	4.021.314	Sequoia Research Products Ltd.	SRP05400n	0	8
**NGP-105-01**	NCP003323	C05	C23H36N2O2	3.725.532	3.725.532	3.722.776	Sequoia Research Products Ltd.	SRP00500f	2	4
**NGP-105-01**	NCP003323	D05	C19H21N3O.0.5C4H6O6	3.824.407	3.073.965	3.071.684	Sequoia Research Products Ltd.	SRP01500z	0	4
**NGP-105-01**	NCP003323	E05	C15H14N4O	2.663.032	2.663.032	2.661.167	Sequoia Research Products Ltd.	SRP03408n	1	5
**NGP-105-01**	NCP003323	F05	C12H21NO8S	3.393.619	3.393.619	3.390.987	Sequoia Research Products Ltd.	SRP02505t	2	9
**NGP-105-01**	NCP003323	G05	C16H14F3N5O	3.493.162	3.493.162	3.491.150	Sequoia Research Products Ltd.	SRP01070v	1	6
**NGP-105-01**	NCP003323	H05	C16H16ClNO3.CH4O3S	4.018.676	3.057.619	3.050.818	Sequoia Research Products Ltd.	SRP00426r	4	4
**NGP-105-01**	NCP003323	A06	C18H19N3O3S.C4H4O4	4.735.003	3.574.283	3.571.147	Sequoia Research Products Ltd.	SRP01315r	1	6
**NGP-105-01**	NCP003323	B06	C20H21FN2O.C2H2O4	4.144.341	3.243.992	3.241.637	Sequoia Research Products Ltd.	SRP01460e	0	3
**NGP-105-01**	NCP003323	C06	C18H26O5	3.224.030	3.224.030	3.221.780	Sequoia Research Products Ltd.	SRP00600z	3	5
**NGP-105-01**	NCP003323	D06	C26H40O5	4.326.030	4.326.030	4.322.875	Sequoia Research Products Ltd.	SRP01328l	3	5
**NGP-105-01**	NCP003323	E06	C10H12N4O3	2.362.310	2.362.310	2.360.909	Sequoia Research Products Ltd.	SRP02715d	2	7
**NGP-105-01**	NCP003323	F06	C17H17Cl2N.HCl	3.426.966	3.062.357	3.050.738	Sequoia Research Products Ltd.	SRP01325s	1	1
**NGP-105-01**	NCP003323	G06	C27H40O3	4.126.152	4.126.152	4.122.977	Sequoia Research Products Ltd.	SRP01060c	3	3

Table S1. NIH Clinical collection

NCC PLATE	PLATE BARCODE	WELL ID	STRUCTURE REAL MF	STRUCTURE REAL AMW	PARENT AMW	PARENT EMW	SAMPLE SUPPLIER	SUPPLIER STRUCTURE ID	NUM H DONORS	NUM H ACCEPTORS
**NGP-105-01**	NCP003323	H06	C27H29NO11.HCl	5.799.900	5.435.291	5.431.740	Sequoia Research Products Ltd.	SRP01310e	7	12
**NGP-105-01**	NCP003323	A07	C18H14F4N2O4S	4.303.748	4.303.748	4.300.610	Sequoia Research Products Ltd.	SRP02002b	2	6
**NGP-105-01**	NCP003323	B07	C28H31N3O6.HCl	5.420.334	5.055.725	5.052.212	Sequoia Research Products Ltd.	SRP010651b	1	9
**NGP-105-01**	NCP003323	C07	C16H14N2O4	2.982.990	2.982.990	2.980.953	Sequoia Research Products Ltd.	SRP05620a	3	6
**NGP-105-01**	NCP003323	D07	C26H33FNO5.Na+	4.815.419	4.585.521	4.582.342	Sequoia Research Products Ltd.	SRP02030c	2	6
**NGP-105-01**	NCP003323	E07	C33H40O15	6.766.740	6.766.740	6.762.367	Sequoia Research Products Ltd.	SRP00325i	8	15
**NGP-105-01**	NCP003323	F07	C20H32O2	3.044.748	3.044.748	3.042.402	Sequoia Research Products Ltd.	SRP02900m	2	2
**NGP-105-01**	NCP003323	G07	C20H24O6	3.604.084	3.604.084	3.601.572	Sequoia Research Products Ltd.	SRP02915t	1	6
**NGP-105-01**	NCP003323	H07	C18H19N3O3S.HCl	3.938.892	3.574.283	3.571.147	Sequoia Research Products Ltd.	SRP01314r	1	6
**NGP-105-01**	NCP003323	A08	C8H9FN2O3	2.001.700	2.001.700	2.000.597	Sequoia Research Products Ltd.	SRP01185t	1	5
**NGP-105-01**	NCP003323	B08	C45H74O10	7.750.810	7.750.810	7.745.281	Sequoia Research Products Ltd.	SRP01150o	4	10
**NGP-105-01**	NCP003323	C08	C24H28N2O5.HCl	4.609.593	4.244.984	4.241.998	Sequoia Research Products Ltd.	SRP01060b	2	7
**NGP-105-01**	NCP003323	D08	C21H32O3	3.324.852	3.324.852	3.322.351	Sequoia Research Products Ltd.	SRP01300o	2	3
**NGP-105-01**	NCP003323	E08	C18H16O3	2.803.242	2.803.242	2.801.099	Sequoia Research Products Ltd.	SRP01425i	0	3
**NGP-105-01**	NCP003323	F08	C18H15NO3	2.933.229	2.933.229	2.931.051	Sequoia Research Products Ltd.	SRP012475o	1	4
**NGP-105-01**	NCP003323	G08	C16H21NO3	2.753.489	2.753.489	2.751.521	Sequoia Research Products Ltd.	SRP013047r	1	4
**NGP-105-01**	NCP003323	H08	C21H25ClFN3O3.C6H8O7	6.140.247	4.219.007	4.211.568	Sequoia Research Products Ltd.	SRP066435m	3	6
**NGP-105-01**	NCP003323	A09	C21H20O12	4.643.838	4.643.838	4.640.954	Sequoia Research Products Ltd.	SRP01950i	8	12
**NGP-105-01**	NCP003323	B09	C15H14FN3O3	3.032.937	3.032.937	3.031.019	Sequoia Research Products Ltd.	SRP01035f	0	6
**NGP-105-01**	NCP003323	C09	C13H12N2O2.HCl	2.647.121	2.282.512	2.280.898	Sequoia Research Products Ltd.	SRP01500o	1	4
**NGP-105-01**	NCP003323	D09	C21H20O12	4.643.838	4.643.838	4.640.954	Sequoia Research Products Ltd.	SRP03990h	8	12
**NGP-105-01**	NCP003323	E09	C46H62N4O11	8.470.222	8.470.222	8.464.415	Sequoia Research Products Ltd.	SRP01275r	5	15
**NGP-105-01**	NCP003323	F09	C16H25NO4.HCl	3.318.412	2.953.803	2.951.783	Sequoia Research Products Ltd.	SRP01475e	2	5
**NGP-105-01**	NCP003323	G09	C22H19N3O4	3.894.117	3.894.117	3.891.375	Sequoia Research Products Ltd.	SRP000800t	1	7
**NGP-105-01**	NCP003323	H09	C15H15NO2S	2.733.505	2.733.505	2.730.823	Sequoia Research Products Ltd.	SRP06607m	2	3
**NGP-105-01**	NCP003323	A10	C27H29NO11.HCl	5.799.900	5.435.291	5.431.740	Sequoia Research Products Ltd.	SRP04600d	7	12
**NGP-105-01**	NCP003323	B10	C9H12ClN5O.HCl	2.781.418	2.416.809	2.410.730	Sequoia Research Products Ltd.	SRP06645m	2	6
**NGP-105-01**	NCP003323	C10	C15H11N3O3	2.812.713	2.812.713	2.810.800	Sequoia Research Products Ltd.	SRP03417n	1	6
**NGP-105-01**	NCP003323	D10	C17H20FN3O3.CH4O3S	4.294.694	3.333.637	3.331.488	Sequoia Research Products Ltd.	SRP01078p	1	6
**NGP-105-01**	NCP003323	E10	C17H27NO2.HCl	3.138.694	2.774.085	2.772.041	Sequoia Research Products Ltd.	SRP01015v	1	3
**NGP-105-01**	NCP003323	F10	C16H14F2N3O4S.Na+	4.053.524	3.823.626	3.820.673	Sequoia Research Products Ltd.	SRP01075p	0	7
**NGP-105-01**	NCP003323	G10	C25H31F3O5S	5.005.753	5.005.753	5.001.844	Sequoia Research Products Ltd.	SRP01977f	1	5
**NGP-105-01**	NCP003323	H10	C36H47N5O4.H2O4S	7.118.816	6.138.031	6.133.628	Sequoia Research Products Ltd.	SRP00675i	4	9
**NGP-105-01**	NCP003323	A11	C18H13ClFN3.HCl	3.622.344	3.257.735	3.250.782	Sequoia Research Products Ltd.	SRP065525m	0	3
**NGP-105-01**	NCP003323	B11	C8H11N3O3S	2.292.543	2.292.543	2.290.521	Sequoia Research Products Ltd.	SRP01125l	3	6
**NGP-105-01**	NCP003323	C11	C12H19N3O.HCl	2.577.644	2.213.035	2.211.528	Sequoia Research Products Ltd.	SRP03580p	3	4
**NGP-105-01**	NCP003323	D11	C34H36MgN6O6S2	7.131.236	7.131.236	7.125.187	Sequoia Research Products Ltd.	SRP01485e	0	12
**NGP-105-01**	NCP003323	E11	C18H14N4O5S	3.983.938	3.983.938	3.980.684	Sequoia Research Products Ltd.	SRP01700s	3	9
**NGP-105-01**	NCP003323	F11	C16H20N4O3S	3.484.210	3.484.210	3.481.256	Sequoia Research Products Ltd.	SRP02525t	3	7
**NGP-105-01**	NCP003323	G11	C17H20N2O2.HCl	3.208.201	2.843.592	2.841.524	Sequoia Research Products Ltd.	SRP02985t	1	4

Table S1. NIH Clinical collection

NCC PLATE	PLATE BARCODE	WELL ID	STRUCTURE REAL MF	STRUCTURE REAL AMW	PARENT AMW	PARENT EMW	SAMPLE SUPPLIER	SUPPLIER STRUCTURE ID	NUM H DONORS	NUM H ACCEPTORS
**NGP-105-01**	NCP003323	H11	C24H33N3O4.2HCl	5.004.675	4.275.457	4.272.471	Sequoia Research Products Ltd.	SRP010131r	2	7
**NGP-105-02**	NCP003403	A02	C16H17N3O4S	3.473.897	3.473.897	3.470.939	Sequoia Research Products Ltd.	SRP01880c	4	7
**NGP-105-02**	NCP003403	B02	C9H12N2O4S	2.442.660	2.442.660	2.440.517	Sequoia Research Products Ltd.	SRP02360p	2	6
**NGP-105-02**	NCP003403	C02	C23H32N2O5	4.165.194	4.165.194	4.162.311	Sequoia Research Products Ltd.	SRP0101151r	2	7
**NGP-105-02**	NCP003403	D02	C22H29N2O.Br-	4.173.908	3.374.868	3.372.279	Sequoia Research Products Ltd.	SRP00470f	2	3
**NGP-105-02**	NCP003403	E02	C18H26O2	2.744.048	2.744.048	2.741.932	Sequoia Research Products Ltd.	SRP00900n	1	2
**NGP-105-02**	NCP003403	F02	C12H21N5O2S2	3.314.523	3.314.523	3.311.136	Sequoia Research Products Ltd.	SRP05385n	2	7
**NGP-105-02**	NCP003403	G02	C4H4FN3O	1.290.939	1.290.939	1.290.338	Sequoia Research Products Ltd.	SRP01027f	3	4
**NGP-105-02**	NCP003403	H02	C15H12N2O2	2.522.732	2.522.732	2.520.898	Sequoia Research Products Ltd.	SRP012480o	2	4
**NGP-105-02**	NCP003403	A03	C15H22N2O4	2.943.520	2.943.520	2.941.579	Sequoia Research Products Ltd.	SRP02990t	2	6
**NGP-105-02**	NCP003403	B03	C10H11NO3	1.932.029	1.932.029	1.930.738	Sequoia Research Products Ltd.	SRP02538a	2	4
**NGP-105-02**	NCP003403	C03	C22H24ClN3O.HCl	4.183.674	3.819.065	3.811.607	Sequoia Research Products Ltd.	SRP07805a	0	4
**NGP-105-02**	NCP003403	D03	C11H16N2O	1.922.618	1.922.618	1.921.262	Sequoia Research Products Ltd.	SRP02375t	3	3
**NGP-105-02**	NCP003403	E03	C15H12O7	3.042.568	3.042.568	3.040.583	Sequoia Research Products Ltd.	SRP01176t	5	7
**NGP-105-02**	NCP003403	F03	C18H20FN3O4	3.613.741	3.613.741	3.611.437	Sequoia Research Products Ltd.	SRP01385l	1	7
**NGP-105-02**	NCP003403	G03	C18H18N6O5S2.C3H8O2	5.385.936	4.624.992	4.620.780	Sequoia Research Products Ltd.	SRP01886c	6	11
**NGP-105-02**	NCP003403	H03	C19H30O5	3.384.460	3.384.460	3.382.093	Sequoia Research Products Ltd.	SRP00400i	1	5
**NGP-105-02**	NCP003403	A04	C15H23N3O4S	3.414.267	3.414.267	3.411.409	Sequoia Research Products Ltd.	SRP01395l	3	7
**NGP-105-02**	NCP003403	B04	C9H8N2O2	1.761.752	1.761.752	1.760.585	Sequoia Research Products Ltd.	SRP01090p	2	4
**NGP-105-02**	NCP003403	C04	C17H11N5	2.853.085	2.853.085	2.851.014	Sequoia Research Products Ltd.	SRP01285l	0	5
**NGP-105-02**	NCP003403	D04	C17H25N3O5S	3.834.641	3.834.641	3.831.514	Sequoia Research Products Ltd.	SRP02110m	3	8
**NGP-105-02**	NCP003403	E04	C29H53NO5	4.957.467	4.957.467	4.953.923	Sequoia Research Products Ltd.	SRP01240o	1	6
**NGP-105-02**	NCP003403	F04	C18H19N3O.HCl	3.298.304	2.933.695	2.931.528	Sequoia Research Products Ltd.	SRP01235o	0	4
**NGP-105-02**	NCP003403	G04	C21H28O2	3.124.538	3.124.538	3.122.089	Sequoia Research Products Ltd.	SRP01390l	1	2
**NGP-105-02**	NCP003403	H04	C17H23NO4.HCl	3.418.362	3.053.753	3.051.627	Sequoia Research Products Ltd.	SRP02040c	3	5
**NGP-105-02**	NCP003403	A05	C17H13ClN4	3.087.708	3.087.708	3.080.828	Sequoia Research Products Ltd.	SRP04905a	0	4
**NGP-105-02**	NCP003403	B05	C9H7Cl2N5	2.560.945	2.560.945	2.550.078	Sequoia Research Products Ltd.	SRP01130l	4	5
**NGP-105-02**	NCP003403	C05	C13H17NO	2.032.851	2.032.851	2.031.310	Sequoia Research Products Ltd.	SRP04515c	0	2
**NGP-105-02**	NCP003403	D05	C13H18ClNO	2.397.461	2.397.461	2.391.076	Sequoia Research Products Ltd.	SRP03725a	1	2
**NGP-105-02**	NCP003403	E05	C19H27N5O4	3.894.561	3.894.561	3.892.063	Sequoia Research Products Ltd.	SRP04165a	3	9
**NGP-105-02**	NCP003403	F05	C17H27N3O4S	3.694.807	3.694.807	3.691.722	Sequoia Research Products Ltd.	SRP05612a	3	7
**NGP-105-02**	NCP003403	G05	C26H27ClN2O.HCl	4.554.287	4.189.678	4.181.811	Sequoia Research Products Ltd.	SRP015615l	0	3
**NGP-105-02**	NCP003403	H05	C23H30N4O2S.HCl	4.630.395	4.265.786	4.262.089	Sequoia Research Products Ltd.	SRP01320P	0	6
**NGP-105-02**	NCP003403	A06	C43H53NO14	8.078.953	8.078.953	8.073.466	Sequoia Research Products Ltd.	SRP04571d	5	15
**NGP-105-02**	NCP003403	B06	C18H18O2	2.663.408	2.663.408	2.661.306	Sequoia Research Products Ltd.	SRP02135h	2	2
**NGP-105-02**	NCP003403	C06	C22H31NO.C4H6O6	4.755.845	3.254.961	3.252.405	Sequoia Research Products Ltd.	SRP02390t	1	2
**NGP-105-02**	NCP003403	D06	C11H16FN3O3	2.572.657	2.572.657	2.571.175	Sequoia Research Products Ltd.	SRP01460c	2	6
**NGP-105-02**	NCP003403	E06	C20H22FNO3	3.433.993	3.433.993	3.431.583	Sequoia Research Products Ltd.	SRP04325m	0	4
**NGP-105-02**	NCP003403	F06	C24H26N6O3	4.465.104	4.465.104	4.462.066	Sequoia Research Products Ltd.	SRP01205o	3	9

Table S1. NIH Clinical collection

NCC PLATE	PLATE BARCODE	WELL ID	STRUCTURE REAL MF	STRUCTURE REAL AMW	PARENT AMW	PARENT EMW	SAMPLE SUPPLIER	SUPPLIER STRUCTURE ID	NUM H DONORS	NUM H ACCEPTORS
**NGP-105-02**	NCP003403	G06	C22H22ClN6O.K+	4.610.089	4.219.106	4.211.543	Sequoia Research Products Ltd.	SRP01580l	1	7
**NGP-105-02**	NCP003403	H06	C6H6N6O2	1.941.530	1.941.530	1.940.552	Sequoia Research Products Ltd.	SRP011925t	2	8
**NGP-105-02**	NCP003403	A07	C20H30O2	3.024.588	3.024.588	3.022.245	Sequoia Research Products Ltd.	SRP04150m	1	2
**NGP-105-02**	NCP003403	B07	C19H15F3N4O3.C7H8O3S	5.765.485	4.043.493	4.041.096	Sequoia Research Products Ltd.	SRP02535t	3	7
**NGP-105-02**	NCP003403	C07	C15H23N3O3S	3.254.273	3.254.273	3.251.460	Sequoia Research Products Ltd.	SRP01690m	1	6
**NGP-105-02**	NCP003403	D07	C17H21NO.HCl	2.918.220	2.553.611	2.551.623	Sequoia Research Products Ltd.	SRP07328a	1	2
**NGP-105-02**	NCP003403	E07	C19H28O8	3.844.282	3.844.282	3.841.784	Sequoia Research Products Ltd.	SRP073035a	1	8
**NGP-105-02**	NCP003403	F07	C35H38Cl2N8O4	7.056.462	7.056.462	7.042.393	Sequoia Research Products Ltd.	SRP02500i	0	12
**NGP-105-02**	NCP003403	G07	C21H27N5O9S2	5.575.951	5.575.951	5.571.250	Sequoia Research Products Ltd.	SRP018875c	3	14
**NGP-105-02**	NCP003403	H07	C17H25NO4.HCl	3.438.522	3.073.913	3.071.783	Sequoia Research Products Ltd.	SRP03422b	0	5
**NGP-105-02**	NCP003403	A08	C13H17ClN2O2	2.687.442	2.687.442	2.680.978	Sequoia Research Products Ltd.	SRP06605m	1	4
**NGP-105-02**	NCP003403	B08	C22H27ClF2O5	4.449.049	4.449.049	4.441.515	Sequoia Research Products Ltd.	SRP01205h	3	5
**NGP-105-02**	NCP003403	C08	C14H9Cl3N2OS	3.596.578	3.596.578	3.579.501	Sequoia Research Products Ltd.	SRP02865t	1	3
**NGP-105-02**	NCP003403	D08	C17H14O4S	3.143.566	3.143.566	3.140.612	Sequoia Research Products Ltd.	SRP013045r	0	4
**NGP-105-02**	NCP003403	E08	C18H31NO4.0.5C4H4O4	3.834.863	3.254.503	3.252.253	Sequoia Research Products Ltd.	SRP020366b	2	5
**NGP-105-02**	NCP003403	F08	C24H21F2NO3	4.094.338	4.094.338	4.091.489	Sequoia Research Products Ltd.	SRP04000e	2	4
**NGP-105-02**	NCP003403	G08	C20H25NO2S2.HCl	4.120.064	3.755.455	3.751.326	Sequoia Research Products Ltd.	SRP023505t	1	3
**NGP-105-02**	NCP003403	H08	C26H27NO9.HCl	5.339.642	4.975.033	4.971.685	Sequoia Research Products Ltd.	SRP000350i	6	10
**NGP-105-02**	NCP003403	A09	C16H12FN3O3	3.132.887	3.132.887	3.130.862	Sequoia Research Products Ltd.	SRP01021f	2	6
**NGP-105-02**	NCP003403	B09	C44H69NO12	8.040.355	8.040.355	8.034.819	Sequoia Research Products Ltd.	SRP00750t	3	13
**NGP-105-02**	NCP003403	C09	C13H20N6O4.HCl	3.608.017	3.243.408	3.241.546	Sequoia Research Products Ltd.	SRP00800v	5	10
**NGP-105-02**	NCP003403	D09	C38H69NO13	7.479.689	7.479.689	7.474.768	Sequoia Research Products Ltd.	SRP03660c	4	14
**NGP-105-02**	NCP003403	E09	C23H27Cl2N3O2	4.483.939	4.483.939	4.471.480	Sequoia Research Products Ltd.	SRP06795a	1	5
**NGP-105-02**	NCP003403	F09	C22H29NO5.C4H4O4	5.035.497	3.874.777	3.872.045	Sequoia Research Products Ltd.	SRP02875t	0	6
**NGP-105-02**	NCP003403	G09	C20H32O2	3.044.748	3.044.748	3.042.402	Sequoia Research Products Ltd.	SRP02260m	1	2
**NGP-105-02**	NCP003403	H09	C20H24N2O6	3.884.218	3.884.218	3.881.634	Sequoia Research Products Ltd.	SRP03410n	1	8
**NGP-105-02**	NCP003403	A10	C20H22O8	3.903.912	3.903.912	3.901.314	Sequoia Research Products Ltd.	SRP02318p	6	8
**NGP-105-02**	NCP003403	B10	C7H11N3O3	1.851.833	1.851.833	1.850.800	Sequoia Research Products Ltd.	SRP010965s	1	6
**NGP-105-02**	NCP003403	C10	C19H27N5O5.HCl	4.419.164	4.054.555	4.052.012	Sequoia Research Products Ltd.	SRP034084n	2	10
**NGP-105-02**	NCP003403	D10	C19H27NO3	3.174.299	3.174.299	3.171.990	Sequoia Research Products Ltd.	SRP03325n	2	4
**NGP-105-02**	NCP003403	E10	C24H32O4	3.845.176	3.845.176	3.842.300	Sequoia Research Products Ltd.	SRP01730m	0	4
**NGP-105-02**	NCP003403	F10	C14H18N4O2	2.743.236	2.743.236	2.741.429	Sequoia Research Products Ltd.	SRP01245o	4	6
**NGP-105-02**	NCP003403	G10	C11H12N2O2S	2.362.892	2.362.892	2.360.619	Sequoia Research Products Ltd.	SRP01100z	3	4
**NGP-105-02**	NCP003403	H10	C10H12N2O4	2.242.170	2.242.170	2.240.797	Sequoia Research Products Ltd.	SRP1625s	2	6
**NGP-105-02**	NCP003403	A11	C16H23N3O4.CH4O3S	4.174.834	3.213.777	3.211.688	Sequoia Research Products Ltd.	SRP00500g	4	7
**NGP-105-02**	NCP003403	B11	C18H13Cl4N3O.HNO3	4.921.463	4.291.335	4.269.812	Sequoia Research Products Ltd.	SRP012492o	0	4
**NGP-105-02**	NCP003403	C11	C42H69NO15	8.280.117	8.280.117	8.274.667	Sequoia Research Products Ltd.	SRP01340k	3	16
**NGP-105-02**	NCP003403	D11	C14H19N5O4	3.213.371	3.213.371	3.211.437	Sequoia Research Products Ltd.	SRP00400f	2	9
**NGP-105-02**	NCP003403	E11	C12H20N2O3S.HCl	3.088.245	2.723.636	2.721.194	Sequoia Research Products Ltd.	SRP01610s	3	5
**NGP-105-02**	NCP003403	F11	C17H18FN3O3S	3.634.077	3.634.077	3.631.052	Sequoia Research Products Ltd.	SRP01330r	1	6

Table S1. NIH Clinical collection

NCC PLATE	PLATE BARCODE	WELL ID	STRUCTURE REAL MF	STRUCTURE REAL AMW	PARENT AMW	PARENT EMW	SAMPLE SUPPLIER	SUPPLIER STRUCTURE ID	NUM H DONORS	NUM H ACCEPTORS
**NGP-105-02**	NCP003403	G11	C15H12O7	3.042.568	3.042.568	3.040.583	Sequoia Research Products Ltd.	SRP01175t	5	7
**NGP-105-02**	NCP003403	H11	C17H18N4O.HCl	3.308.181	2.943.572	2.941.480	Sequoia Research Products Ltd.	SRP04894a	1	5
**NGP-105-03**	NCP003483	A02	C16H24N2O4	3.083.790	3.083.790	3.081.736	Sequoia Research Products Ltd.	SRP00500u	5	6
**NGP-105-03**	NCP003483	B02	C26H28ClNO.C6H8O7	5.980.931	4.059.691	4.051.859	Sequoia Research Products Ltd.	SRP02530t	0	2
**NGP-105-03**	NCP003483	C02	C59H84N18O14.C2H4O2	13.294.852	12.694.332	12.686.414	Sequoia Research Products Ltd.	SRP02570g	20	32
**NGP-105-03**	NCP003483	D02	C20H26O6	3.624.244	3.624.244	3.621.729	Sequoia Research Products Ltd.	SRP01097s	4	6
**NGP-105-03**	NCP003483	E02	C21H22N4O6S	4.584.902	4.584.902	4.581.260	Sequoia Research Products Ltd.	SRP010115r	4	10
**NGP-105-03**	NCP003483	F02	C24H30N2O2.HCl	4.149.771	3.785.162	3.782.307	Sequoia Research Products Ltd.	SRP04574d	0	4
**NGP-105-03**	NCP003483	G02	C14H16N2O.0.5C4H6O4	2.873.388	2.282.948	2.281.262	Tocris Bioscience	912	2	3
**NGP-105-03**	NCP003483	H02	C23H26N2O4	3.944.720	3.944.720	3.941.892	Sigma Chemical Company	399027	0	6
**NGP-105-03**	NCP003483	A03	C11H12N2	1.722.304	1.722.304	1.721.000	Sigma Chemical Company	300764	2	2
**NGP-105-03**	NCP003483	B03	C22H26F3N3OS.2HCl	5.104.466	4.375.248	4.371.748	Sigma Chemical Company	F4765	1	4
**NGP-105-03**	NCP003483	C03	C19H24N2O.HCl	3.328.747	2.964.138	2.961.888	Sequoia Research Products Ltd.	SRP02460p	0	3
**NGP-105-03**	NCP003483	D03	C14H13O3.Na+	2.522.460	2.292.562	2.290.864	Sigma Chemical Company	M1275	0	3
**NGP-105-03**	NCP003483	E03	C15H22N2O.HCl	2.828.147	2.463.538	2.461.732	Sigma Chemical Company	M3189	1	3
**NGP-105-03**	NCP003483	F03	C17H12Br2O3	4.240.892	4.240.892	4.219.153	Sigma Chemical Company	B5774	1	3
**NGP-105-03**	NCP003483	G03	C21H26N2O7	4.184.482	4.184.482	4.181.740	Sigma Chemical Company	N149	1	9
**NGP-105-03**	NCP003483	H03	C27H33N3O8	5.275.763	5.275.763	5.272.267	Sigma Chemical Company	R2253	6	11
**NGP-105-03**	NCP003483	A04	C11H14N4O2	2.342.586	2.342.586	2.341.116	Sigma Chemical Company	M9017	0	6
**NGP-105-03**	NCP003483	B04	C8H11N3O6	2.451.925	2.451.925	2.450.647	Sigma Chemical Company	A1882	4	9
**NGP-105-03**	NCP003483	C04	C21H30O4	3.464.686	3.464.686	3.462.144	Sigma Chemical Company	R0500	2	4
**NGP-105-03**	NCP003483	D04	C6H7NO	1.091.281	1.091.281	1.090.527	Sigma Chemical Company	P66807	1	2
**NGP-105-03**	NCP003483	E04	C21H23ClFNO2.HCl	4.123.328	3.758.719	3.751.401	Tocris Bioscience	100330	1	3
**NGP-105-03**	NCP003483	F04	C14H18O3	2.342.962	2.342.962	2.341.255	Tocris Bioscience	100267	1	3
**NGP-105-03**	NCP003483	G04	C19H20FN3	3.093.875	3.093.875	3.091.641	Tocris Bioscience	100737	0	3
**NGP-105-03**	NCP003483	H04	C21H34NO3.Br-	4.284.119	3.485.079	3.482.538	Sigma Chemical Company	O5501	1	4
**NGP-105-03**	NCP003483	A05	C10H15NO2	1.812.355	1.812.355	1.811.102	Sigma Chemical Company	D136204	2	3
**NGP-105-03**	NCP003483	B05	C8H13N3O4S	2.472.697	2.472.697	2.470.626	Sigma Chemical Company	T3021	0	7
**NGP-105-03**	NCP003483	C05	C13H21N5O4.C6H5NO2	4.344.511	3.113.421	3.111.593	Sigma Chemical Company	X6750	2	9
**NGP-105-03**	NCP003483	D05	C9H13NO2	1.672.085	1.672.085	1.670.946	Sigma Chemical Company	s0752	3	3
**NGP-105-03**	NCP003483	E05	C14H12O3	2.282.482	2.282.482	2.280.786	Sigma Chemical Company	R5010	3	3
**NGP-105-03**	NCP003483	F05	C6H6O3	1.261.122	1.261.122	1.260.316	Sigma Chemical Company	H43407	1	3
**NGP-105-03**	NCP003483	G05	C7H7N3	1.331.531	1.331.531	1.330.639	Sigma Chemical Company	A59565	3	3
**NGP-105-03**	NCP003483	H05	C19H22FN3O3	3.594.017	3.594.017	3.591.645	Sigma Chemical Company	17849	1	6
**NGP-105-03**	NCP003483	A06	C24H34O5	4.025.330	4.025.330	4.022.406	Sigma Chemical Company	30830	1	5
**NGP-105-03**	NCP003483	B06	C15H14ClN3O4S	3.678.077	3.678.077	3.670.393	Sigma Chemical Company	C6895	4	7
**NGP-105-03**	NCP003483	C06	C10H10N2	1.582.034	1.582.034	1.580.843	Sigma Chemical Company	116416	0	2
**NGP-105-03**	NCP003483	D06	C18H19NOS.HCl	3.338.770	2.974.161	2.971.187	Sequoia Research Products Ltd.	SRP05005d	1	2
**NGP-105-03**	NCP003483	E06	C23H32N6O4S.C6H8O7	6.807.308	4.886.068	4.882.205	Sequoia Research Products Ltd.	SRP01005v	1	10

Table S1. NIH Clinical collection

NCC PLATE	PLATE BARCODE	WELL ID	STRUCTURE REAL MF	STRUCTURE REAL AMW	PARENT AMW	PARENT EMW	SAMPLE SUPPLIER	SUPPLIER STRUCTURE ID	NUM H DONORS	NUM H ACCEPTORS
**NGP-105-03**	NCP003483	F06	C17H26N2O.HCl	3.108.687	2.744.078	2.742.045	Sequoia Research Products Ltd.	SRP01307r	1	3
**NGP-105-03**	NCP003483	G06	C17H19N5	2.933.725	2.933.725	2.931.640	Sequoia Research Products Ltd.	SRP05647a	0	5
**NGP-105-03**	NCP003483	H06	C19H19NOS.C4H4O4	4.254.991	3.094.271	3.091.187	Tocris Bioscience	100029	0	2
**NGP-105-03**	NCP003483	A07	C22H32O3	3.444.962	3.444.962	3.442.351	Sigma Chemical Company	M6013	1	3
**NGP-105-03**	NCP003483	B07	C13H19N5.H2O	2.633.438	2.453.285	2.451.640	Sigma Chemical Company	P154	2	5
**NGP-105-03**	NCP003483	C07	C7H5N3O2	1.631.359	1.631.359	1.630.381	Sigma Chemical Company	N7778	1	5
**NGP-105-03**	NCP003483	D07	C11H14N2O	1.902.458	1.902.458	1.901.106	Sigma Chemical Company	286583	3	3
**NGP-105-03**	NCP003483	E07	C12H9NS	1.992.707	1.992.707	1.990.455	Sigma Chemical Company	P14831	1	1
**NGP-105-03**	NCP003483	F07	C10H12ClN5O3	2.856.907	2.856.907	2.850.628	Sequoia Research Products Ltd.	SRP03655c	4	8
**NGP-105-03**	NCP003483	G07	C18H24N4O.HCl	3.488.771	3.124.162	3.121.950	Sequoia Research Products Ltd.	SRP02575g	1	5
**NGP-105-03**	NCP003483	H07	C21H27N3.0.5H2O.2HCl	4.033.966	3.214.671	3.212.204	Tocris Bioscience	1497	1	3
**NGP-105-03**	NCP003483	A08	C22H27N3O2	3.654.769	3.654.769	3.652.103	Tocris Bioscience	1347	1	5
**NGP-105-03**	NCP003483	B08	C22H29FO4	3.764.700	3.764.700	3.762.049	Sigma Chemical Company	D6038	2	4
**NGP-105-03**	NCP003483	C08	C16H19ClN2.C4H4O4	3.908.664	2.747.944	2.741.236	Sequoia Research Products Ltd.	SRP02129d	0	2
**NGP-105-03**	NCP003483	D08	C12H17N5	2.313.015	2.313.015	2.311.483	Tocris Bioscience	1355	2	5
**NGP-105-03**	NCP003483	E08	C20H21N5O3S.2H2O	4.475.103	4.114.797	4.111.365	Tocris Bioscience	781	4	8
**NGP-105-03**	NCP003483	F08	C22H21Cl2IN4O	5.552.467	5.552.467	5.540.137	Tocris Bioscience	1117	1	5
**NGP-105-03**	NCP003483	G08	C19H25F3N4O.2C4H4O4	6.145.745	3.824.305	3.821.980	Tocris Bioscience	646	3	5
**NGP-105-03**	NCP003483	H08	C17H17NO2.1H2O.HCl	3.353.162	2.673.285	2.671.259	Tocris Bioscience	884	3	3
**NGP-105-03**	NCP003483	A09	C21H27N3O3S.2HCl	4.744.471	4.015.253	4.011.773	Tocris Bioscience	1808	1	6
**NGP-105-03**	NCP003483	B09	C26H21N3O.0.25H2O.2HC l	4.688.991	3.914.735	3.911.684	Tocris Bioscience	1999	0	4
**NGP-105-03**	NCP003483	C09	C22H29ClO5	4.089.240	4.089.240	4.081.703	Sigma Chemical Company	B0385	3	5
**NGP-105-03**	NCP003483	D09	C17H19N3O3S	3.454.173	3.454.173	3.451.147	Sigma Chemical Company	O104	1	6
**NGP-105-03**	NCP003483	E09	C19H20N2O3.CH4O3S	4.204.863	3.243.806	3.241.473	Sequoia Research Products Ltd.	SRP045724d	1	5
**NGP-105-03**	NCP003483	F09	C16H21N3O2	2.873.629	2.873.629	2.871.633	Sequoia Research Products Ltd.	SRP01300z	2	5
**NGP-105-03**	NCP003483	G09	C27H28O11	5.285.144	5.285.144	5.281.631	Sequoia Research Products Ltd.	SRP02720t	4	11
**NGP-105-03**	NCP003483	H09	C62H86N12O16	12.554.408	12.554.408	12.546.284	Sequoia Research Products Ltd.	SRP00450d	6	28
**NGP-105-03**	NCP003483	A10	C16H25NO2.HCl	2.998.424	2.633.815	2.631.885	Sequoia Research Products Ltd.	SRP02565t	1	3
**NGP-105-03**	NCP003483	B10	C16H14ClN3O	2.997.605	2.997.605	2.990.825	Sequoia Research Products Ltd.	SRP02170c	1	4
**NGP-105-03**	NCP003483	C10	C16H15N5O7S2.3H2O	5.074.912	4.534.453	4.530.412	Sequoia Research Products Ltd.	SRP018865c	5	12
**NGP-105-03**	NCP003483	D10	C14H13N5O5S2	3.954.085	3.954.085	3.950.358	Sequoia Research Products Ltd.	SRP018855c	5	10
**NGP-105-03**	NCP003483	E10	C11H12Cl2N2O.HCl	2.955.967	2.591.358	2.580.326	Sequoia Research Products Ltd.	SRP01565l	1	3
**NGP-105-03**	NCP003483	F10	C17H15N3O6	3.573.235	3.573.235	3.570.960	Sequoia Research Products Ltd.	SRP00900b	4	9
**NGP-105-03**	NCP003483	G10	C21H23NO3.HCl	3.738.808	3.374.199	3.371.677	Sequoia Research Products Ltd.	SRP01210o	1	4
**NGP-105-03**	NCP003483	H10	C50H46CaF2N2O8	8.810.035	8.810.035	8.804.022	Sequoia Research Products Ltd.	SRP02390i	4	10
**NGP-105-03**	NCP003483	A11	C21H28O5	3.604.520	3.604.520	3.601.936	Sigma Chemical Company	C2755	2	5
**NGP-105-03**	NCP003483	B11	C21H21N.HCl	3.238.666	2.874.057	2.871.673	Sigma Chemical Company	C6022	0	1
**NGP-105-03**	NCP003483	C11	C29H39NO9	5.456.323	5.456.323	5.452.624	Sequoia Research Products Ltd.	SRP02125h	3	10
**NGP-105-03**	NCP003483	D11	C21H30O4	3.464.686	3.464.686	3.462.144	Sigma Chemical Company	C2505	2	4

Table S1. NIH Clinical collection

NCC PLATE	PLATE BARCODE	WELL ID	STRUCTURE REAL MF	STRUCTURE REAL AMW	PARENT AMW	PARENT EMW	SAMPLE SUPPLIER	SUPPLIER STRUCTURE ID	NUM H DONORS	NUM H ACCEPTORS
**NGP-105-03**	NCP003483	E11	C34H57N2O4.Br-	6.377.450	5.578.410	5.574.318	Sequoia Research Products Ltd.	SRP01010v	0	6
**NGP-105-03**	NCP003483	F11	C21H28O2	3.124.538	3.124.538	3.122.089	Sequoia Research Products Ltd.	SRP02352t	1	2
**NGP-105-03**	NCP003483	G11	C6H6N2O	1.221.268	1.221.268	1.220.480	Sigma Chemical Company	240206	2	3
**NGP-105-03**	NCP003483	H11	C16H18N4O2	2.983.456	2.983.456	2.981.429	Sigma Chemical Company	252999	3	6
**NGP-105-04**	NCP003563	A02	C10H12ClN5O4	3.016.901	3.016.901	3.010.577	Sigma Chemical Company	C5134	5	9
**NGP-105-04**	NCP003563	B02	C7H10ClN3O3	2.196.283	2.196.283	2.190.410	Sigma Chemical Company	O5879	1	6
**NGP-105-04**	NCP003563	C02	C12H19N2.I-	3.182.014	1.912.974	1.911.548	Sigma Chemical Company	D5891	0	2
**NGP-105-04**	NCP003563	D02	C23H24FN3O2	3.934.623	3.934.623	3.931.852	Sigma Chemical Company	P126	0	5
**NGP-105-04**	NCP003563	E02	C21H26O2	3.104.378	3.104.378	3.101.932	Sigma Chemical Company	855871	1	2
**NGP-105-04**	NCP003563	F02	C20H25NO3.HCl	3.638.858	3.274.249	3.271.834	Sigma Chemical Company	B704	1	4
**NGP-105-04**	NCP003563	G02	C7H10N2	1.221.704	1.221.704	1.220.843	Sigma Chemical Company	A55306	2	2
**NGP-105-04**	NCP003563	H02	C2H2Cl2O2	1.289.428	1.289.428	1.279.431	Sigma Chemical Company	D54702	1	2
**NGP-105-04**	NCP003563	A03	C22H22FN3O2S.1H2O.HCl	4.749.792	4.114.953	4.111.416	Tocris Bioscience	1809	1	5
**NGP-105-04**	NCP003563	B03	C17H20N2O.HCl	3.048.207	2.683.598	2.681.575	Tocris Bioscience	1622	3	3
**NGP-105-04**	NCP003563	C03	C18H29NO3.HCl	3.438.958	3.074.349	3.072.147	Tocris Bioscience	906	2	4
**NGP-105-04**	NCP003563	D03	C16H10N2O2	2.622.682	2.622.682	2.620.742	Sequoia Research Products Ltd.	SRP00750i	2	4
**NGP-105-04**	NCP003563	E03	C17H20ClN3O3.HCl	3.862.792	3.498.183	3.491.193	Tocris Bioscience	380	1	6
**NGP-105-04**	NCP003563	F03	C19H25Cl2N3O3.C4H4O4	5.304.053	4.143.333	4.131.272	Tocris Bioscience	1483	0	6
**NGP-105-04**	NCP003563	G03	C25H31O8.Na+	4.825.080	4.595.182	4.592.018	Sigma Chemical Company	P4153	2	8
**NGP-105-04**	NCP003563	H03	C8H7ClN2O2S	2.306.692	2.306.692	2.299.916	Sigma Chemical Company	D9035	1	4
**NGP-105-04**	NCP003563	A04	C18H20N2O6	3.603.678	3.603.678	3.601.321	Sequoia Research Products Ltd.	SRP00418n	1	8
**NGP-105-04**	NCP003563	B04	C38H50N6O5.CH4O3S	7.669.609	6.708.552	6.703.842	Sequoia Research Products Ltd.	SRP01070s	6	11
**NGP-105-04**	NCP003563	C04	C22H18N2	3.103.994	3.103.994	3.101.469	Sequoia Research Products Ltd.	SRP02006b	0	2
**NGP-105-04**	NCP003563	D04	C14H21N3O2S.C4H6O4	4.134.889	2.954.009	2.951.354	Sequoia Research Products Ltd.	SRP01780s	2	5
**NGP-105-04**	NCP003563	E04	C20H24O2	2.964.108	2.964.108	2.961.776	Sequoia Research Products Ltd.	SRP03000e	0	2
**NGP-105-04**	NCP003563	F04	C12H9N3O5S	3.072.811	3.072.811	3.070.262	Sequoia Research Products Ltd.	SRP03412n	1	8
**NGP-105-04**	NCP003563	G04	C16H13ClN2O	2.847.458	2.847.458	2.840.716	Sequoia Research Products Ltd.	SRP02375d	0	3
**NGP-105-04**	NCP003563	H04	C21H25N3O2S.C4H4O4	4.995.819	3.835.099	3.831.667	Sequoia Research Products Ltd.	SRP01075q	1	5
**NGP-105-04**	NCP003563	A05	C27H30O16	6.105.274	6.105.274	6.101.533	Sequoia Research Products Ltd.	SRP01350r	10	16
**NGP-105-04**	NCP003563	B05	C10H15N5O3	2.532.617	2.532.617	2.531.174	Sequoia Research Products Ltd.	SRP01095p	5	8
**NGP-105-04**	NCP003563	C05	C27H44O3	4.166.472	4.166.472	4.163.290	Sequoia Research Products Ltd.	SRP01075c	3	3
**NGP-105-04**	NCP003563	D05	C30H32N2O2.HCl	4.890.591	4.525.982	4.522.463	Sequoia Research Products Ltd.	SRP04225d	0	4
**NGP-105-04**	NCP003563	E05	C11H14N2O4.0.33H2O	2.441.890	2.382.440	2.380.953	Tocris Bioscience	100813	4	6
**NGP-105-04**	NCP003563	F05	C22H22FN3O2	3.794.353	3.794.353	3.791.696	Tocris Bioscience	101276	1	5
**NGP-105-04**	NCP003563	G05	C13H18N4O3	2.783.120	2.783.120	2.781.378	Tocris Bioscience	101755	0	7
**NGP-105-04**	NCP003563	H05	C14H16N2O2	2.442.942	2.442.942	2.441.211	Tocris Bioscience	1471	0	4
**NGP-105-04**	NCP003563	A06	C37H48N6O5S2	7.209.482	7.209.482	7.203.127	Sequoia Research Products Ltd.	SRP01303r	4	11
**NGP-105-04**	NCP003563	B06	C45H54N4O8.2C4H6O6	10.791.258	7.789.490	7.783.941	Sequoia Research Products Ltd.	SRP01040v	2	12
**NGP-105-04**	NCP003563	C06	C16H20FN3O4	3.373.521	3.373.521	3.371.437	Sequoia Research Products Ltd.	SRP01440l	1	7

Table S1. NIH Clinical collection

NCC PLATE	PLATE BARCODE	WELL ID	STRUCTURE REAL MF	STRUCTURE REAL AMW	PARENT AMW	PARENT EMW	SAMPLE SUPPLIER	SUPPLIER STRUCTURE ID	NUM H DONORS	NUM H ACCEPTORS
**NGP-105-04**	NCP003563	D06	C27H30F2N2O3.2HCl	5.414.673	4.685.455	4.682.224	Sequoia Research Products Ltd.	SRP01568l	0	5
**NGP-105-04**	NCP003563	E06	C14H9ClF3NO2	3.156.798	3.156.798	3.150.273	Sequoia Research Products Ltd.	SRP01025e	1	3
**NGP-105-04**	NCP003563	F06	C25H28N6O	4.285.386	4.285.386	4.282.324	Sequoia Research Products Ltd.	SRP01502i	1	7
**NGP-105-04**	NCP003563	G06	C27H36N2O4	4.525.960	4.525.960	4.522.675	Sequoia Research Products Ltd.	SRP01040r	2	6
**NGP-105-04**	NCP003563	H06	C20H32O	2.884.754	2.884.754	2.882.453	Sequoia Research Products Ltd.	SRP02030e	1	1
**NGP-105-04**	NCP003563	A07	C16H16O3	2.563.022	2.563.022	2.561.099	Sequoia Research Products Ltd.	SRP03680p	1	3
**NGP-105-04**	NCP003563	B07	C19H28N2O4.HCl	3.849.049	3.484.440	3.482.049	Sequoia Research Products Ltd.	SRP01326r	1	6
**NGP-105-04**	NCP003563	C07	C16H19BrN2.C4H4O4	4.353.174	3.192.454	3.180.731	Sequoia Research Products Ltd.	SRP02128d	0	2
**NGP-105-04**	NCP003563	D07	C10H7Cl2N3O.HCl	2.925.524	2.560.915	2.549.966	Sequoia Research Products Ltd.	SRP05646a	1	4
**NGP-105-04**	NCP003563	E07	C16H23N5O.C4H4O4	4.174.649	3.013.929	3.011.902	Sequoia Research Products Ltd.	SRP01187t	4	6
**NGP-105-04**	NCP003563	F07	C12H9N3O	2.112.235	2.112.235	2.110.745	Sequoia Research Products Ltd.	SRP06575m	1	4
**NGP-105-04**	NCP003563	G07	C21H25ClN2O3	3.888.956	3.888.956	3.881.553	Sequoia Research Products Ltd.	SRP01384l	1	5
**NGP-105-04**	NCP003563	H07	C20H21FN2O.HBr	4.053.111	3.243.992	3.241.637	Sequoia Research Products Ltd.	SRP03585c	0	3
**NGP-105-04**	NCP003563	A08	C14H14ClNS.HCl	3.002.466	2.637.857	2.630.535	Sequoia Research Products Ltd.	SRP02355t	0	1
**NGP-105-04**	NCP003563	B08	C15H17O3.Na+	2.682.890	2.452.992	2.451.177	Sequoia Research Products Ltd.	SRP01595l	0	3
**NGP-105-04**	NCP003563	C08	C31H33N3O6S	5.756.815	5.756.815	5.752.090	Sequoia Research Products Ltd.	SRP00200z	2	9
**NGP-105-04**	NCP003563	D08	C21H25N.HCl	3.278.986	2.914.377	2.911.986	Sequoia Research Products Ltd.	SRP01197t	0	1
**NGP-105-04**	NCP003563	E08	C19H21N3O5	3.713.941	3.713.941	3.711.481	Sequoia Research Products Ltd.	SRP01970i	1	8
**NGP-105-04**	NCP003563	F08	C24H29N5O3	4.355.277	4.355.277	4.352.270	Sequoia Research Products Ltd.	SRP00900v	2	8
**NGP-105-04**	NCP003563	G08	C15H13N3O4S.0.25H2O	3.358.505	3.313.467	3.310.626	Tocris Bioscience	100486	2	7
**NGP-105-04**	NCP003563	H08	C19H28NO3.Br-	3.983.419	3.184.379	3.182.069	Sequoia Research Products Ltd.	SRP02450g	1	4
**NGP-105-04**	NCP003563	A09	C15H21N3O2.0.5H2O4S	3.243.912	2.753.519	2.751.633	Tocris Bioscience	100352	1	5
**NGP-105-04**	NCP003563	B09	C27H34ClNO2S	4.720.875	4.720.875	4.711.998	Tocris Bioscience	1311	1	3
**NGP-105-04**	NCP003563	C09	C17H22N2O.C4H6O4	3.884.638	2.703.758	2.701.732	Tocris Bioscience	100918	0	3
**NGP-105-04**	NCP003563	D09	C15H22N2O.HCl	2.828.147	2.463.538	2.461.732	Tocris Bioscience	100047	2	3
**NGP-105-04**	NCP003563	E09	C4H3FN2O	1.140.792	1.140.792	1.140.229	Tocris Bioscience	101322	1	3
**NGP-105-04**	NCP003563	F09	C16H19ClN2.C4H4O4	3.908.664	2.747.944	2.741.236	Tocris Bioscience	100728	0	2
**NGP-105-04**	NCP003563	G09	C19H27N3O5S2	4.415.621	4.415.621	4.411.392	Sequoia Research Products Ltd.	SRP045723d	2	8
**NGP-105-04**	NCP003563	H09	C19H24N2O4.2H2O.C4H4O4	4.965.146	3.444.120	3.441.736	Sequoia Research Products Ltd.	SRP02131f	4	6
**NGP-105-04**	NCP003563	A10	C15H19N5.C7H6O2	3.914.715	2.693.505	2.691.640	Sequoia Research Products Ltd.	SRP013035r	1	5
**NGP-105-04**	NCP003563	B10	C47H64N4O12	8.770.486	8.770.486	8.764.520	Sequoia Research Products Ltd.	SRP01280r	6	16
**NGP-105-04**	NCP003563	C10	C24H31ClO7	4.669.608	4.669.608	4.661.758	Sequoia Research Products Ltd.	SRP01583l	1	7
**NGP-105-04**	NCP003563	D10	C18H24N2O5	3.484.004	3.484.004	3.481.685	Sequoia Research Products Ltd.	SRP010855e	3	7
**NGP-105-04**	NCP003563	E10	C24H29NO3.HCl	4.159.618	3.795.009	3.792.147	Sequoia Research Products Ltd.	SRP04573d	0	4
**NGP-105-04**	NCP003563	F10	C16H13N3O3	2.952.983	2.952.983	2.950.956	Sequoia Research Products Ltd.	SRP034095n	0	6
**NGP-105-04**	NCP003563	G10	C8H9N3O4	2.111.777	2.111.777	2.110.593	Sequoia Research Products Ltd.	SRP034079n	1	7
**NGP-105-04**	NCP003563	H10	C33H30N4O2	5.146.286	5.146.286	5.142.368	Sequoia Research Products Ltd.	SRP01192t	1	6
**NGP-105-04**	NCP003563	A11	C20H26N2O4.HCl	3.948.999	3.584.390	3.581.892	Sequoia Research Products Ltd.	SRP02400i	1	6
**NGP-105-04**	NCP003563	B11	C43H51N3O11	7.858.945	7.858.945	7.853.523	Sequoia Research Products Ltd.	SRP01281r	5	14

Table S1. NIH Clinical collection

NCC PLATE	PLATE BARCODE	WELL ID	STRUCTURE REAL MF	STRUCTURE REAL AMW	PARENT AMW	PARENT EMW	SAMPLE SUPPLIER	SUPPLIER STRUCTURE ID	NUM H DONORS	NUM H ACCEPTORS
**NGP-105-04**	NCP003563	C11	C35H35ClNO3S.Na+	6.081.727	5.851.829	5.842.026	Sequoia Research Products Ltd.	SRP06641m	1	4
**NGP-105-04**	NCP003563	D11	C9H13N3O3	2.112.213	2.112.213	2.110.956	Sequoia Research Products Ltd.	SRP00250z	3	6
**NGP-105-04**	NCP003563	E11	C9H9Cl2N3.HCl	2.665.580	2.300.971	2.290.173	Tocris Bioscience	690	2	3
**NGP-105-04**	NCP003563	F11	C24H31N5O2	4.215.443	4.215.443	4.212.477	Tocris Bioscience	1105	1	7
**NGP-105-04**	NCP003563	G11	C10H13N5O3.0.25H2O	2.557.495	2.512.457	2.511.018	Tocris Bioscience	101399	4	8
**NGP-105-04**	NCP003563	H11	C21H27NO2.0.5H2O.0.5C4 H6O6	4.095.044	3.254.525	3.252.041	Tocris Bioscience	100657	2	3
**NGP-105-05**	NCP003643	A02	C7H7NO3	1.531.379	1.531.379	1.530.425	Tocris Bioscience	101661	4	4
**NGP-105-05**	NCP003643	B02	C19H20FNO3.C4H4O4	4.454.443	3.293.723	3.291.427	Tocris Bioscience	100038	1	4
**NGP-105-05**	NCP003643	C02	C22H27NO2.HCl	3.739.244	3.374.635	3.372.041	Tocris Bioscience	100344	1	3
**NGP-105-05**	NCP003643	D02	C7H16N4O2.C2H4O2	2.482.826	1.882.306	1.881.273	Tocris Bioscience	101931	6	6
**NGP-105-05**	NCP003643	E02	C15H13NO3.C4H11NO3	3.764.089	2.552.739	2.550.895	Sequoia Research Products Ltd.	SRP01182k	1	4
**NGP-105-05**	NCP003643	F02	C17H23ClO4	3.268.216	3.268.216	3.261.284	Tocris Bioscience	101478	0	4
**NGP-105-05**	NCP003643	G02	C22H18O11	4.583.794	4.583.794	4.580.849	Tocris Bioscience	100468	8	11
**NGP-105-05**	NCP003643	H02	C15H20Cl2N2O3	3.472.426	3.472.426	3.460.850	Tocris Bioscience	100155	2	5
**NGP-105-05**	NCP003643	A03	C15H20ClN3O2.H2O.HCl	3.642.731	3.097.969	3.091.244	Tocris Bioscience	100111	3	5
**NGP-105-05**	NCP003643	B03	C17H18BrNO.HBr	4.131.530	3.322.411	3.310.571	Tocris Bioscience	100174	1	2
**NGP-105-05**	NCP003643	C03	C26H37NO2	3.955.875	3.955.875	3.952.824	Tocris Bioscience	100122	2	3
**NGP-105-05**	NCP003643	D03	C21H27NO4.HCl	3.939.122	3.574.513	3.571.940	Tocris Bioscience	100313	3	5
**NGP-105-05**	NCP003643	E03	C11H16N2O2.HCl	2.447.221	2.082.612	2.081.211	Tocris Bioscience	101761	0	4
**NGP-105-05**	NCP003643	F03	C17H18N2O6	3.463.408	3.463.408	3.461.164	Tocris Bioscience	101709	1	8
**NGP-105-05**	NCP003643	G03	C15H13FO2	2.442.662	2.442.662	2.440.899	Tocris Bioscience	101507	1	2
**NGP-105-05**	NCP003643	H03	C7H9NO2	1.391.545	1.391.545	1.390.633	Tocris Bioscience	101427	1	3
**NGP-105-05**	NCP003643	A04	C18H18ClN3O.C4H6O4	4.459.025	3.278.145	3.271.138	Tocris Bioscience	100738	0	4
**NGP-105-05**	NCP003643	B04	C18H25NO.H2O.HBr	3.703.313	2.714.041	2.711.936	Tocris Bioscience	101433	0	2
**NGP-105-05**	NCP003643	C04	C18H19NOS	2.974.161	2.974.161	2.971.187	Tocris Bioscience	100318	1	2
**NGP-105-05**	NCP003643	D04	C21H23NO4S	3.854.793	3.854.793	3.851.347	Tocris Bioscience	101458	1	5
**NGP-105-05**	NCP003643	E04	C14H10Cl2NNaO2	3.181.352	3.181.352	3.169.986	Tocris Bioscience	101435	1	3
**NGP-105-05**	NCP003643	F04	C21H30O2	3.144.698	3.144.698	3.142.245	Tocris Bioscience	101771	0	2
**NGP-105-05**	NCP003643	G04	C8H15N7O2S3	3.374.337	3.374.337	3.370.449	Tocris Bioscience	101156	8	9
**NGP-105-05**	NCP003643	H04	C10H14ClN3.HCl	2.481.560	2.116.951	2.110.876	Tocris Bioscience	100714	2	3
**NGP-105-05**	NCP003643	A05	C35H60N2O4.2Br-	7.326.840	5.728.760	5.724.553	Tocris Bioscience	100349	0	6
**NGP-105-05**	NCP003643	B05	C6H9N3O3	1.711.563	1.711.563	1.710.643	Tocris Bioscience	101667	1	6
**NGP-105-05**	NCP003643	C05	C17H21NO4.3H2O.HBr	4.383.171	3.033.593	3.031.470	Tocris Bioscience	101890	1	5
**NGP-105-05**	NCP003643	D05	C27H38N2O4.0.5H2O.HCl	5.000.806	4.546.120	4.542.831	Tocris Bioscience	101617	0	6
**NGP-105-05**	NCP003643	E05	C13H17N.HCl	2.237.466	1.872.857	1.871.360	Tocris Bioscience	101891	0	1
**NGP-105-05**	NCP003643	F05	C18H27NO3	3.054.189	3.054.189	3.051.990	Tocris Bioscience	100605	2	4
**NGP-105-05**	NCP003643	G05	C13H21NO3.0.5H2O4S	2.883.552	2.393.159	2.391.521	Tocris Bioscience	101881	4	4
**NGP-105-05**	NCP003643	H05	C17H25NO.HCl	2.958.540	2.593.931	2.591.936	Tocris Bioscience	100353	1	2

Table S1. NIH Clinical collection

NCC PLATE	PLATE BARCODE	WELL ID	STRUCTURE REAL MF	STRUCTURE REAL AMW	PARENT AMW	PARENT EMW	SAMPLE SUPPLIER	SUPPLIER STRUCTURE ID	NUM H DONORS	NUM H ACCEPTORS
**NGP-105-05**	NCP003643	A06	C15H18O7.C15H16O6	6.025.887	3.103.048	3.101.052	Tocris Bioscience	100596	2	7
**NGP-105-05**	NCP003643	B06	C19H25N5O4.2H2O.HCl	4.599.316	3.874.401	3.871.906	Tocris Bioscience	100080	2	9
**NGP-105-05**	NCP003643	C06	C15H10O	2.062.444	2.062.444	2.060.731	Tocris Bioscience	101438	0	1
**NGP-105-05**	NCP003643	D06	C4H5NO3S	1.471.489	1.471.489	1.469.990	Tocris Bioscience	101743	2	4
**NGP-105-05**	NCP003643	E06	C21H26N2OS2.C6H6O3S	5.447.440	3.865.718	3.861.486	Tocris Bioscience	100739	0	3
**NGP-105-05**	NCP003643	F06	C12H10F2N2O3	2.682.205	2.682.205	2.680.659	Tocris Bioscience	101260	1	5
**NGP-105-05**	NCP003643	G06	C20H24ClN3S.2C4H4O4	6.060.891	3.739.451	3.731.379	Tocris Bioscience	101287	0	3
**NGP-105-05**	NCP003643	H06	C12H16N2	1.882.734	1.882.734	1.881.313	Tocris Bioscience	101561	2	2
**NGP-105-05**	NCP003643	A07	C22H17ClN2	3.448.444	3.448.444	3.441.080	Tocris Bioscience	101397	0	2
**NGP-105-05**	NCP003643	B07	C22H23ClN2O2	3.828.912	3.828.912	3.821.448	Tocris Bioscience	100099	0	4
**NGP-105-05**	NCP003643	C07	C8H12N2.H2O4S	2.342.759	1.361.974	1.361.000	Tocris Bioscience	100790	3	2
**NGP-105-05**	NCP003643	D07	C8H5F3N2OS.HCl	2.706.570	2.341.961	2.340.074	Tocris Bioscience	100955	2	3
**NGP-105-05**	NCP003643	E07	C26H26N2O3.1H2O.HCl	4.779.894	4.145.056	4.141.943	Tocris Bioscience	101181	3	5
**NGP-105-05**	NCP003643	F07	C9H12N2	1.482.084	1.482.084	1.481.000	Tocris Bioscience	100188	1	2
**NGP-105-05**	NCP003643	G07	C18H23NO.HCl	3.058.490	2.693.881	2.691.779	Tocris Bioscience	100103	1	2
**NGP-105-05**	NCP003643	H07	C13H8ClN5O	2.856.929	2.856.929	2.850.417	Tocris Bioscience	100341	2	6
**NGP-105-05**	NCP003643	A08	C20H24N2OS.0.25H2O.HCl	3.814.495	3.404.848	3.401.609	Tocris Bioscience	100354	1	3
**NGP-105-05**	NCP003643	B08	C23H29ClFN3O4.H2O	4.839.694	4.659.541	4.651.830	Tocris Bioscience	100112	3	7
**NGP-105-05**	NCP003643	C08	C16H15Cl2N.HCl	3.286.696	2.922.087	2.910.581	Tocris Bioscience	100118	1	1
**NGP-105-05**	NCP003643	D08	C19H22ClN5O.HCl	4.083.318	3.718.709	3.711.512	Tocris Bioscience	100843	0	6
**NGP-105-05**	NCP003643	E08	C19H21N5O4.0.75H2O.HC l	4.333.805	3.834.081	3.831.593	Tocris Bioscience	100730	2	9
**NGP-105-05**	NCP003643	F08	C20H29N5O3.HCl	4.239.446	3.874.837	3.872.270	Tocris Bioscience	100121	1	8
**NGP-105-05**	NCP003643	G08	C10H12N2O	1.762.188	1.762.188	1.760.949	Tocris Bioscience	100252	0	3
**NGP-105-05**	NCP003643	H08	C3H6N2O2	1.020.932	1.020.932	1.020.429	Tocris Bioscience	101840	3	4
**NGP-105-05**	NCP003643	A09	C15H21F3N2O2.C4H4O4	4.344.125	3.183.405	3.181.555	Tocris Bioscience	100328	2	4
**NGP-105-05**	NCP003643	B09	C19H21NO.HCl	3.158.440	2.793.831	2.791.623	Tocris Bioscience	100107	0	2
**NGP-105-05**	NCP003643	C09	C21H24F3N3S.2HCl	4.804.202	4.074.984	4.071.643	Tocris Bioscience	100741	0	3
**NGP-105-05**	NCP003643	D09	C17H23NO.C4H6O6	4.074.655	2.573.771	2.571.779	Tocris Bioscience	100721	1	2
**NGP-105-05**	NCP003643	E09	C16H22N4O3	3.183.770	3.183.770	3.181.691	Tocris Bioscience	100601	2	7
**NGP-105-05**	NCP003643	F09	C20H23N.HCl	3.138.716	2.774.107	2.771.830	Tocris Bioscience	100747	1	1
**NGP-105-05**	NCP003643	G09	C19H21NS.C4H4O4	4.115.157	2.954.437	2.951.394	Tocris Bioscience	100752	0	1
**NGP-105-05**	NCP003643	H09	C18H24O2	2.723.888	2.723.888	2.721.776	Tocris Bioscience	101474	2	2
**NGP-105-05**	NCP003643	A10	C10H20N2O2	2.002.822	2.002.822	2.001.524	Tocris Bioscience	101533	2	4
**NGP-105-05**	NCP003643	B10	C17H21NO.HCl	2.918.220	2.553.611	2.551.623	Tocris Bioscience	100917	0	2
**NGP-105-05**	NCP003643	C10	C17H21NO3.HBr	3.682.718	2.873.599	2.871.521	Tocris Bioscience	100060	1	4
**NGP-105-05**	NCP003643	D10	C19H16ClNO4.0.25H2O	3.622.981	3.577.943	3.570.767	Tocris Bioscience	100475	1	5
**NGP-105-05**	NCP003643	E10	C10H20N2S4	2.965.234	2.965.234	2.960.509	Tocris Bioscience	100320	0	2
**NGP-105-05**	NCP003643	F10	C16H18N4O2.0.5H2O.HCl	3.438.142	2.983.456	2.981.429	Tocris Bioscience	100731	0	6
**NGP-105-05**	NCP003643	G10	C26H28Cl2N4O4	5.314.404	5.314.404	5.301.487	Tocris Bioscience	100180	0	8

Table S1. NIH Clinical collection

NCC PLATE	PLATE BARCODE	WELL ID	STRUCTURE REAL MF	STRUCTURE REAL AMW	PARENT AMW	PARENT EMW	SAMPLE SUPPLIER	SUPPLIER STRUCTURE ID	NUM H DONORS	NUM H ACCEPTORS
**NGP-105-05**	NCP003643	H10	C16H21N3.HCl	2.918.250	2.553.641	2.551.735	Tocris Bioscience	100727	0	3
**NGP-105-05**	NCP003643	A11	C6H8ClN7O.2H2O.HCl	3.021.208	2.296.293	2.290.478	Tocris Bioscience	101735	8	8
**NGP-105-05**	NCP003643	B11	C13H14N2.HCl	2.347.293	1.982.684	1.981.156	Tocris Bioscience	101925	2	2
**NGP-105-05**	NCP003643	C11	C20H24O2	2.964.108	2.964.108	2.961.776	Tocris Bioscience	101476	2	2
**NGP-105-05**	NCP003643	D11	C9H13N3O5	2.432.201	2.432.201	2.430.855	Tocris Bioscience	101401	5	8
**NGP-105-05**	NCP003643	E11	C20H22N8O5.3H2O	5.084.925	4.544.466	4.541.713	Tocris Bioscience	101662	7	13
**NGP-105-05**	NCP003643	F11	C11H13F3N2.HCl	2.666.946	2.302.337	2.301.030	Tocris Bioscience	100743	1	2
**NGP-105-05**	NCP003643	G11	C10H17N3S	2.113.261	2.113.261	2.111.143	Tocris Bioscience	100677	3	3
**NGP-105-05**	NCP003643	H11	C14H22N2O	2.343.428	2.343.428	2.341.732	Tocris Bioscience	101620	1	3
**NGP-105-06**	NCP003723	A02	C26H26N2O3.H2O.HCl	4.689.818	4.145.056	4.141.943	Tocris Bioscience	1410	3	5
**NGP-105-06**	NCP003723	B02	C19H30N2O.HCl	3.389.227	3.024.618	3.022.358	Tocris Bioscience	100337	3	3
**NGP-105-06**	NCP003723	C02	C23H24N2O.H2O.2HBr	5.242.969	3.444.578	3.441.888	Tocris Bioscience	921	1	3
**NGP-105-06**	NCP003723	D02	C19H28O2	2.884.318	2.884.318	2.882.089	Tocris Bioscience	101473	1	2
**NGP-105-06**	NCP003723	E02	C17H15N5O	3.053.399	3.053.399	3.051.276	Sequoia Research Products Ltd.	SRP00300z	0	6
**NGP-105-06**	NCP003723	F02	C14H12F3NOS	2.993.114	2.993.114	2.990.591	Tocris Bioscience	100115	2	2
**NGP-105-06**	NCP003723	G02	C43H65N5O10	8.120.205	8.120.205	8.114.731	Sequoia Research Products Ltd.	SRP01191t	1	15
**NGP-105-06**	NCP003723	H02	C13H9NOSe	2.741.811	2.741.811	2.749.849	Tocris Bioscience	101457	0	2
**NGP-105-06**	NCP003723	A03	C17H12BrFO2S2	4.113.042	4.113.042	4.099.446	Tocris Bioscience	101416	0	2
**NGP-105-06**	NCP003723	B03	C25H37NO4	4.155.753	4.155.753	4.152.722	Tocris Bioscience	1660	4	5
**NGP-105-06**	NCP003723	C03	C17H18ClNO.HCl	3.242.510	2.877.901	2.871.076	Tocris Bioscience	925	1	2
**NGP-105-06**	NCP003723	D03	C19H28O2	2.884.318	2.884.318	2.882.089	Tocris Bioscience	101406	1	2
**NGP-105-06**	NCP003723	E03	C20H34O5	3.544.890	3.544.890	3.542.406	Sequoia Research Products Ltd.	SRP04907a	3	5
**NGP-105-06**	NCP003723	F03	C21H32N2O	3.284.998	3.284.998	3.282.514	Sequoia Research Products Ltd.	SRP01623s	2	3
**NGP-105-06**	NCP003723	G03	C15H20ClN5O4.H2O	3.878.244	3.698.091	3.691.203	Tocris Bioscience	100086	4	9
**NGP-105-06**	NCP003723	H03	C17H17F3N4.2C4H4O4	5.664.891	3.343.451	3.341.405	Tocris Bioscience	100333	0	4
**NGP-105-06**	NCP003723	A04	C43H55N5O7.H2O4S	8.520.208	7.539.423	7.534.101	Sequoia Research Products Ltd.	SRP01038v	6	12
**NGP-105-06**	NCP003723	B04	C46H56N4O10.H2O4S	9.230.533	8.249.748	8.243.996	Sequoia Research Products Ltd.	SRP01037v	3	14
**NGP-105-06**	NCP003723	C04	C26H33NO6	4.555.531	4.555.531	4.552.307	Sequoia Research Products Ltd.	SRP00900l	1	7
**NGP-105-06**	NCP003723	D04	C17H19N3	2.653.591	2.653.591	2.651.578	Sequoia Research Products Ltd.	SRP06582m	0	3
**NGP-105-06**	NCP003723	E04	C20H21N3O7S	4.474.639	4.474.639	4.471.100	Sequoia Research Products Ltd.	SRP05637a	1	10
**NGP-105-06**	NCP003723	F04	C24H34N4O5S	4.906.198	4.906.198	4.902.249	Sequoia Research Products Ltd.	SRP01525g	3	9
**NGP-105-06**	NCP003723	G04	C20H25ClN2O5	4.088.834	4.088.834	4.081.451	Sequoia Research Products Ltd.	SRP05624a	3	7
**NGP-105-06**	NCP003723	H04	C18H21N3O3S	3.594.443	3.594.443	3.591.303	Sequoia Research Products Ltd.	SRP00950r	1	6
**NGP-105-06**	NCP003723	A05	C27H22Cl2N4	4.734.058	4.734.058	4.721.221	Sigma Chemical Company	C8895	1	4
**NGP-105-06**	NCP003723	B05	C33H38N4O6.3H2O.HCl	6.771.970	5.866.902	5.862.791	Sequoia Research Products Ltd.	SRP01505i	1	10
**NGP-105-06**	NCP003723	C05	C16H14F3N3O2S	3.693.622	3.693.622	3.690.758	Sequoia Research Products Ltd.	SRP01175l	1	5
**NGP-105-06**	NCP003723	D05	C19H19ClN2	3.108.274	3.108.274	3.101.236	Sequoia Research Products Ltd.	SRP02090d	1	2
**NGP-105-06**	NCP003723	E05	C18H21O5S.Na+	3.724.128	3.494.230	3.491.109	Sigma Chemical Company	E0251	0	5
**NGP-105-06**	NCP003723	F05	C29H35NO2	4.296.045	4.296.045	4.292.667	Sigma Chemical Company	M8046	1	3
**NGP-105-06**	NCP003723	G05	C29H32O13	5.885.672	5.885.672	5.881.842	Sigma Chemical Company	E1383	3	13

Table S1. NIH Clinical collection

NCC PLATE	PLATE BARCODE	WELL ID	STRUCTURE REAL MF	STRUCTURE REAL AMW	PARENT AMW	PARENT EMW	SAMPLE SUPPLIER	SUPPLIER STRUCTURE ID	NUM H DONORS	NUM H ACCEPTORS
**NGP-105-06**	NCP003723	H05	C17H26ClN	2.798.547	2.798.547	2.791.753	Tocris Bioscience	2290	0	1
**NGP-105-06**	NCP003723	A06	C14H17ClN4S	3.088.298	3.088.298	3.080.862	Tocris Bioscience	752	3	4
**NGP-105-06**	NCP003723	B06	C15H18N2O	2.423.218	2.423.218	2.421.419	Sequoia Research Products Ltd.	SRP02175h	3	3
**NGP-105-06**	NCP003723	C06	C17H26ClN.H2O.HCl	3.343.309	2.798.547	2.791.753	Sequoia Research Products Ltd.	SRP01355s	0	1
**NGP-105-06**	NCP003723	D06	C15H10Cl2N2O2	3.211.632	3.211.632	3.200.119	Sequoia Research Products Ltd.	SRP01577l	2	4
**NGP-105-06**	NCP003723	E06	C20H26N2O4.2HBr	5.202.628	3.584.390	3.581.892	Tocris Bioscience	411	1	6
**NGP-105-06**	NCP003723	F06	C15H21N5O5.H2O.HCl	4.058.397	3.513.635	3.511.542	Tocris Bioscience	1957	5	10
**NGP-105-06**	NCP003723	G06	C25H29I2NO3.HCl	6.817.818	6.453.209	6.450.237	Sequoia Research Products Ltd.	SRP05610a	0	4
**NGP-105-06**	NCP003723	H06	C23H34O5	3.905.220	3.905.220	3.902.406	Sequoia Research Products Ltd.	SRP06551m	1	5
**NGP-105-06**	NCP003723	A07	C29H31N7O.CH4O3S	5.897.190	4.936.133	4.932.590	Sequoia Research Products Ltd.	SRP000530i	2	8
**NGP-105-06**	NCP003723	B07	C16H22FNO.HCl	2.998.174	2.633.565	2.631.685	Tocris Bioscience	100740	0	2
**NGP-105-06**	NCP003723	C07	C19H17N2O4S.Na+	3.924.058	3.694.160	3.690.909	Sequoia Research Products Ltd.	SRP010755P	0	6
**NGP-105-06**	NCP003723	D07	C19H26N2S.CH4O3S	4.105.961	3.144.904	3.141.816	Tocris Bioscience	101757	1	2
**NGP-105-06**	NCP003723	E07	C53H72N2O12.2C6H5O3S	12.434.936	9.291.652	9.285.085	Sequoia Research Products Ltd.	SRP07340a	0	14
**NGP-105-06**	NCP003723	F07	C16H26O5	2.983.810	2.983.810	2.981.780	Sequoia Research Products Ltd.	SRP07301a	0	5

Table S2. ENZO – Natural Product library

Plate number	Plate Location	Name	IUPAC	MW	CAS	Catalog number	Conc	Solvent	Plate part number	Plate description
1	1-A01	Acivicin	(2R)-2-amino-2-[(5S)-3-chloro-4,5-dihydro-1,2-oxazol-5-yl]acetic acid	178,6	42228-92-2	EI-113	2mg/ml	DMSO	2865	Natural Products Library
1	1-A02	Actinomycin D	2-amino-4,6-dimethyl-3-oxo-1-N,9-N-bis[7,11,14-trimethyl-2,5,9,12,15-pentaoxo-3,10-di(propan-2-yl)-8-oxa-1,4,11,14-tetrazabicyclo[14.3.0]nonadecan-6-yl]phenoxazine-1,9-dicarboxamide	1255,5	50-76-0	GR-300	2mg/ml	DMSO	2865	Natural Products Library
1	1-A03	Anisomycin	(2R,3S,4S)-4-hydroxy-2-[(3-methoxyphenyl)methyl]pyrrolidin-3-yl acetate	265,3	22862-76-6	ST-102	2mg/ml	DMSO	2865	Natural Products Library
1	1-A04	Antibiotic A-23187	5-(methylamino)-2-{[(2S,3S,8S,9R,11R)-3,9,11-trimethyl-8-[1-oxo-1-(1H-pyrrol-2-yl)propan-2-yl]-1,7-dioxaspiro[5.5]undecan-2-yl]methyl}-1,3-benzoxazole-4-carboxylic acid	523,6	52665-69-7	CA-100	2mg/ml	DMSO	2865	Natural Products Library
1	1-A05	Aristolochic acid A	6-methoxy-9-nitro-14,16-dioxatetracyclo[8.7.0.$0^{2,7}$.$0^{13,17}$]heptadeca-1(10),2(7),3,5,8,11,13(17)-heptaene-11-carboxylic acid	341,3	313-67-7	EI-175	2mg/ml	DMSO	2865	Natural Products Library
1	1-A06	Artesunate	4-oxo-4-{[(1S,4S,5R,8S,9R,10S,12R,13R)-1,5,9-trimethyl-11,14,15,16-tetraoxatetracyclo[10.3.1.$0^{4,13}$.$0^{8,13}$]hexadecan-10-yl]oxy}butanoic acid	384,4	88495-63-0	PR-117	2mg/ml	DMSO	2865	Natural Products Library
1	1-A07	Australine·HCl	(1R,2R,3R,7S,7aR)-3-(hydroxymethyl)-hexahydro-1H-pyrrolizine-1,2,7-triol hydrochloride	225,7	118396-02-4	S-105	2mg/ml	DMSO	2865	Natural Products Library
1	1-A08	Baicalein	5,6,7-trihydroxy-2-phenyl-4H-chromen-4-one	270,2	491-67-8	EI-106	2mg/ml	DMSO	2865	Natural Products Library
1	1-A09	Betulinic acid	(1R,2R,5S,8R,9R,10R,13R,14R,17S,19R)-17-hydroxy-1,2,14,18,18-pentamethyl-8-(prop-1-en-2-yl)pentacyclo[11.8.0.$0^{2,10}$.$0^{5,9}$.$0^{14,19}$]henicosane-5-carboxylic acid	456,7	472-15-1	AP-301	2mg/ml	DMSO	2865	Natural Products Library
1	1-A10	Bilobalide	(1S,4R,7R,8S,9R,11S)-9-tert-butyl-7,9-dihydroxy-3,5,12-trioxatetracyclo[6.6.0.$0^{1,11}$.$0^{4,8}$]tetradecane-2,6,13-trione	326,3	33570-04-6	N-140	2mg/ml	DMSO	2865	Natural Products Library
1	1-A11	Brefeldin A	(1R,6S,11aS,13S,14aR)-1,13-dihydroxy-6-methyl-1H,4H,6H,7H,8H,9H,11aH,12H,13H,14H,14aH-cyclopenta[f]oxacyclotridecan-4-one	280,4	20350-15-6	G-405	2mg/ml	DMSO	2865	Natural Products Library
1	1-A12	Bromocriptine mesylate	(4R,7R)-10-bromo-N-[(1S,2S,4R,7S)-2-hydroxy-7-(2-methylpropyl)-5,8-dioxo-4-(propan-2-yl)-3-oxa-6,9-diazatricyclo[7.3.0.$0^{2,6}$]dodecan-4-yl]-6-methyl-6,11-diazatetracyclo[7.6.1.$0^{2,7}$.$0^{12,16}$]hexadeca-1(15),2,9,12(16),13-pentaene-4-carboxamide methanesulfonate	749,7	22260-51-1	D-102	2mg/ml	DMSO	2865	Natural Products Library
1	1-B01	C2 Phytoceramide	N-[(2S,3S,4R)-1,3,4-trihydroxyoctadecan-2-yl]acetamide	359,5		SL-151	2mg/ml	DMSO	2865	Natural Products Library
1	1-B02	C6 Ceramide	N-[(2S,3R,4E)-1,3-dihydroxyoctadec-4-en-2-yl]hexanamide	397,6	124753-97-5	SL-110	2mg/ml	DMSO	2865	Natural Products Library
1	1-B03	Caffeic acid	(2E)-3-(3,4-dihydroxyphenyl)prop-2-enoic acid	180,2	331-39-5	EI-124	2mg/ml	DMSO	2865	Natural Products Library

Table S2. ENZO – Natural Product library

Plate number	Plate Location	Name	IUPAC	MW	CAS	Catalog number	Conc	Solvent	Plate part number	Plate description
1	1-B04	Camptothecin	(16S)-16-ethyl-16-hydroxy-18-oxa-1,10-diazapentacyclo$[11.8.0.0^{2,11}.0^{4,9}.0^{15,20}]$henicosa-2,4,6,8,10,13,15(20)-heptaene-17,21-dione	348,4	7689-03-4	GR-301	2mg/ml	DMSO	2865	Natural Products Library
1	1-B05	Cantharidin	2,6-dimethyl-4,10-dioxatricyclo$[5.2.1.0^{2,6}]$decane-3,5-dione	196,2	56-25-7	PR-105	2mg/ml	DMSO	2865	Natural Products Library
1	1-B06	CAPE	2-phenylethyl (2E)-3-(3,4-dihydroxyphenyl)prop-2-enoate	284,3	104594-70-9	FR-102	2mg/ml	DMSO	2865	Natural Products Library
1	1-B07	Capsaicin	(6E)-N-[(4-hydroxy-3-methoxyphenyl)methyl]-8-methylnon-6-enamide	305,4	404-86-4	EI-125	2mg/ml	DMSO	2865	Natural Products Library
1	1-B08	Castanospermine	(1S,6S,7R,8R,8aR)-octahydroindolizine-1,6,7,8-tetrol	189,2	79831-76-8	S-107	2mg/ml	DMSO	2865	Natural Products Library
1	1-B09	Cerulenin	(2R,3S)-3-[(4E,7E)-nona-4,7-dienoyl]oxirane-2-carboxamide	223,3	17397-89-6	G-237	2mg/ml	DMSO	2865	Natural Products Library
1	1-B10	Cevadine	(1R,2S,6S,9S,10R,11S,12S,14R,15S,18S,19S,22S,23S,25R)-1,10,11,12,14,23-hexahydroxy-6,10,19-trimethyl-24-oxa-4-azaheptacyclo$[12.12.0.0^{2,11}.0^{4,9}.0^{15,25}.0^{18,23}.0^{19,25}]$hexacosan-22-yl (2E)-2-methylbut-2-enoate	591,7	62-59-9	NA-104	2mg/ml	DMSO	2865	Natural Products Library
1	1-B11	Chaetomellic acid A	disodium (2Z)-2-methyl-3-tetradecylbut-2-enedioate	370,4	148796-51-4	G-229	2mg/ml	DMSO	2865	Natural Products Library
1	1-B12	Chelerythrine	17,18-dimethoxy-21-methyl-5,7-dioxa-21-azapentacyclo$[11.8.0.0^{2,10}.0^{4,8}.0^{14,19}]$henicosa-1(13),2(10),3,8,11,14,16,18,20-nonaen-21-ium chloride	383,8	3895-92-9	EI-225	2mg/ml	DMSO	2865	Natural Products Library
1	1-C01	Chromomycin A3	6-{[(6S,7S)-6-({4-[(4-{[5-(acetyloxy)-4-hydroxy-4,6-dimethyloxan-2-yl]oxy}-5-hydroxy-6-methyloxan-2-yl)oxy]-5-hydroxy-6-methyloxan-2-yl}oxy)-7-[(1S,3S,4R)-3,4-dihydroxy-1-methoxy-2-oxopentyl]-4,10-dihydroxy-3-methyl-5-oxo-5,6,7,8-tetrahydroanthracen-2-yl]oxy}-4-[(4-hydroxy-5-methoxy-6-methyloxan-2-yl)oxy]-2-methyloxan-3-yl acetate	1183,2	7059-24-7	GR-302	2mg/ml	DMSO	2865	Natural Products Library
1	1-C02	Citrinin	(3R,4S)-8-hydroxy-3,4,5-trimethyl-6-oxo-4,6-dihydro-3H-2-benzopyran-7-carboxylic acid	250,2	518-75-2	CM-116	2mg/ml	DMSO	2865	Natural Products Library
1	1-C03	Colchicine	N-[(10S)-3,4,5,14-tetramethoxy-13-oxotricyclo$[9.5.0.0^{2,7}]$hexadeca-1(16),2(7),3,5,11,14-hexaen-10-yl]acetamide	399,4	64-86-8	T-118	2mg/ml	DMSO	2865	Natural Products Library
1	1-C04	Coumermycin A1	(3S,4R,5S)-5-hydroxy-6-[(4-hydroxy-3-{5-[(4-hydroxy-7-{[(3R,4S,5R)-3-hydroxy-5-methoxy-6,6-dimethyl-4-(5-methyl-1H-pyrrole-2-carbonyloxy)oxan-2-yl]oxy}-8-methyl-2-oxo-2H-chromen-3-yl)carbamoyl]-4-methyl-1H-pyrrole-3-amido}-8-methyl-2-oxo-2H-chromen-7-yl)oxy]-3-methoxy-2,2-dimethyloxan-4-yl 5-methyl-1H-pyrrole-2-carboxylate	1110,1	4434-05-3	GR-317	2mg/ml	DMSO	2865	Natural Products Library

Table S2. ENZO – Natural Product library

Plate number	Plate Location	Name	IUPAC	MW	CAS	Catalog number	Conc	Solvent	Plate part number	Plate description
**1**	1-C05	Curcumin	1-(3-hydroxy-2-methoxyphenyl)-7-(3-hydroxy-4-methoxyphenyl)hepta-1,6-diene-3,5-dione	368,4	458-37-7	EI-135	2mg/ml	DMSO	2865	Natural Products Library
**1**	1-C06	Cycloheximide	4-[(2R)-2-[(1S,3S,5S)-3,5-dimethyl-2-oxocyclohexyl]-2-hydroxyethyl]piperidine-2,6-dione	281,3	66-81-9	GR-310	2mg/ml	DMSO	2865	Natural Products Library
**1**	1-C07	Cyclopamine	(3S,3'R,3'aS,6'S,6aS,6bS,7'aR,9R,11aS,11bR)-3',6',10,11b-tetramethyl-1,2,3,3'a,4,4',5',6,6',6a,6b,7,7',7'a,8,11,11a,11b-octadecahydro-3'H-spiro[cyclohexa[a]fluorene-9,2'-furo[3,2-b]pyridine]-3-ol	411,6	4449-51-8	GR-334	2mg/ml	DMSO	2865	Natural Products Library
**1**	1-C08	Cyclopiazonic acid	(2R,3S,5Z,9R)-5-(1-hydroxyethylidene)-8,8-dimethyl-7,16-diazapentacyclo[9.6.1.$0^{2,9}$.$0^{3,7}$.$0^{15,18}$]octadeca-1(17),11,13,15(18)-tetraene-4,6-dione	336,4	18172-33-3	CA-415	2mg/ml	DMSO	2865	Natural Products Library
**1**	1-C09	L-Cycloserine	(4R)-4-amino-1,2-oxazolidin-3-one	102,1	339-72-0	SL-200	2mg/ml	DMSO	2865	Natural Products Library
**1**	1-C10	Cyclosporin A	(3S,6S,9S,12R,15S,18S,21S,24S,30S,33S)-30-ethyl-33-[(1R,2R,4E)-1-hydroxy-2-methylhex-4-en-1-yl]-1,4,7,10,12,15,19,25,28-nonamethyl-6,9,18,24-tetrakis(2-methylpropyl)-3,21-bis(propan-2-yl)-1,4,7,10,13,16,19,22,25,28,31-undecaazacyclotritriacontan-2,5,8,11,14,17,20,23,26,29,32-undecone	1202,6	59865-13-3	A-195	2mg/ml	DMSO	2865	Natural Products Library
**1**	1-C11	Cytochalasin B	(5R,9R,13S,15S,15aS,16S,18aS,18bS)-16-benzyl-5,13-dihydroxy-9,15-dimethyl-14-methylidene-2H,5H,6H,7H,8H,9H,10H,13H,14H,15H,15aH,16H,17H,18H,18bH-oxacyclotetradeca[3,2-e]isoindole-2,18-dione	479,6	14930-96-2	T-108	2mg/ml	DMSO	2865	Natural Products Library
**1**	1-C12	Cytochalasin D	(3S,4S,6S,6aR,10S,12S,15R,15aR,15bR)-3-benzyl-6,12-dihydroxy-4,10,12-trimethyl-5-methylidene-1,11-dioxo-1H,2H,3H,4H,5H,6H,6aH,9H,10H,11H,12H,15H,15bH-cycloundeca[e]isoindol-15-yl acetate	507,6	22144-77-0	T-109	2mg/ml	DMSO	2865	Natural Products Library
**1**	1-D01	Cytochalasin E	(1S,5E,7S,9S,11E,13S,14S,16R,17S,18S,19S)-19-benzyl-7-hydroxy-7,9,16,17-tetramethyl-2,4,15-trioxa-20-azatetracyclo[11.8.0.$0^{1,18}$.$0^{14,16}$]henicosa-5,11-diene-3,8,21-trione	495,6	36011-19-5	CT-120	2mg/ml	DMSO	2865	Natural Products Library
**1**	1-D02	Daidzein	7-hydroxy-3-(4-hydroxyphenyl)-4H-chromen-4-one	254,2	486-66-8	ST-110	2mg/ml	DMSO	2865	Natural Products Library
**1**	1-D03	Daunorubicin	(8S,10S)-8-acetyl-10-[(4-amino-5-hydroxy-6-methyloxan-2-yl)oxy]-6,8,11-trihydroxy-1-methoxy-5,7,8,9,10,12-hexahydrotetracene-5,12-dione hydrochloride	564,0	23541-50-6	GR-318	2mg/ml	DMSO	2865	Natural Products Library
**1**	1-D04	Decoyinine	(2R,3R,4S)-2-(6-amino-9H-purin-9-yl)-2-(hydroxymethyl)-5-methylideneoxolane-3,4-diol	279,3	2004-04-8	A-230	2mg/ml	DMSO	2865	Natural Products Library
**1**	1-D05	Degeulin	(1S,14S)-17,18-dimethoxy-7,7-dimethyl-2,8,21-trioxapentacyclo[12.8.0.$0^{3,12}$.$0^{4,9}$.$0^{15,20}$]docosa-3(12),4(9),5,10,15(20),16,18-heptaen-13-one	394,4	522-17-8	EI-329	2mg/ml	DMSO	2865	Natural Products Library

Table S2. ENZO – Natural Product library

Plate number	Plate Location	Name	IUPAC	MW	CAS	Catalog number	Conc	Solvent	Plate part number	Plate description
1	1-D06	13-O-Acetylphorbol	(1R,2S,6R,10S,11R,13S,15R)-1,6-dihydroxy-8-(hydroxymethyl)-4,12,12,15-tetramethyl-5-oxotetracyclo[8.5.0.0^{2,6}.0^{11,13}]pentadeca-3,8-dien-13-yl acetate	390,5	60857-08-1	PE-187	2mg/ml	DMSO	2865	Natural Products Library
1	1-D07	12-Deoxyphorbol 13-phenylacetate 20-acetate	[(1R,2S,6R,10S,11R,13S,15R)-1,6-dihydroxy-4,12,12,15-tetramethyl-5-oxo-13-[(2-phenylacetyl)oxy]tetracyclo[8.5.0.0^{2,6}.0^{11,13}]pentadeca-3,8-dien-8-yl]methyl acetate	508,6	54662-30-5	PE-182	2mg/ml	DMSO	2865	Natural Products Library
1	1-D08	Dihydroergocristine mesylate	(2R,4R,7R)-N-[(1S,2S,4R,7S)-7-benzyl-2-hydroxy-5,8-dioxo-4-(propan-2-yl)-3-oxa-6,9-diazatricyclo[7.3.0.0^{2,6}]dodecan-4-yl]-6-methyl-6,11-diazatetracyclo[7.6.1.0^{2,7}.0^{12,16}]hexadeca-1(15),9,12(16),13-tetraene-4-carboxamide; methanesulfonic acid	707,8	24730-10-7	NS-108	2mg/ml	DMSO	2865	Natural Products Library
1	1-D09	Domoic acid	(2S,3S,4S)-4-[(2Z,4E,6R)-6-carboxy-6-methylhexa-2,4-dien-2-yl]-3-(carboxymethyl)pyrrolidine-2-carboxylic acid	311,3	14277-97-5	EA-117	2mg/ml	DMSO	2865	Natural Products Library
1	1-D10	Doxorubicin	(8S,10S)-10-[(4-amino-5-hydroxy-6-methyloxan-2-yl)oxy]-6,8,11-trihydroxy-8-(2-hydroxyacetyl)-1-methoxy-5,7,8,9,10,12-hexahydrotetracene-5,12-dione hydrochloride	580,0	25316-40-9	GR-319	2mg/ml	DMSO	2865	Natural Products Library
1	1-D11	E6 Berbamine	(1S,14R)-20,21,25-trimethoxy-15,30-dimethyl-7,23-dioxa-15,30-diazaheptacyclo[22.6.2.2^{3,6}.1^{8,12}.1^{14,18}.0^{27,31}.0^{22,33}]hexatriaconta-3,5,8(34),9,11,18(33),19,21,24,26,31,35-dodecaen-9-yl 4-nitrobenzoate	757,8	73885-53-7	CA-302	2mg/ml	DMSO	2865	Natural Products Library
1	1-D12	E-64	(2S,3S)-3-{[(1S)-1-[(4-carbamimidamidobutyl)carbamoyl]-3-methylbutyl]carbamoyl}oxirane-2-carboxylic acid	357,4	66701-25-5	PI-105	2mg/ml	DMSO	2865	Natural Products Library
1	1-E01	E-64-C	(2S,3S)-3-{[(1S)-3-methyl-1-[(3-methylbutyl)carbamoyl]butyl]carbamoyl}oxirane-2-carboxylic acid	314,4	76684-89-4	PI-106	2mg/ml	DMSO	2865	Natural Products Library
1	1-E02	E-64-D	ethyl (2S)-3-{[(1S)-3-methyl-1-[(3-methylbutyl)carbamoyl]butyl]carbamoyl}oxirane-2-carboxylate	342,4	88321-09-9	PI-107	2mg/ml	DMSO	2865	Natural Products Library
1	1-E03	Ebelactone B	(3S,4S)-3-ethyl-4-[(2S,4E,6R,8S,9R,10R)-9-hydroxy-4,6,8,10-tetramethyl-7-oxododec-4-en-2-yl]oxetan-2-one	352,5	76808-15-6	G-223	2mg/ml	DMSO	2865	Natural Products Library
1	1-E04	Ellipticine	5,11-dimethyl-6H-pyrido[4,3-b]carbazole	246,3	519-23-3	GR-315	2mg/ml	DMSO	2865	Natural Products Library
1	1-E05	Embelin	2,5-dihydroxy-3-undecylcyclohexa-2,5-diene-1,4-dione	294,4	550-24-3	CM-125	2mg/ml	DMSO	2865	Natural Products Library
1	1-E06	(±)-Epibatadine	2-(6-chloropyridin-3-yl)-7-azabicyclo[2.2.1]heptane	208,7	140111-52-0	C-114	2mg/ml	DMSO	2865	Natural Products Library
1	1-E07	Epigallocatechin	(2R,3R)-5,7-dihydroxy-2-(3,4,5-trihydroxyphenyl)-3,4-dihydro-2H-1-benzopyran-3-yl 3,4,5-trihydroxybenzoate	458,4	989-51-5	FR-109	2mg/ml	DMSO	2865	Natural Products Library

Table S2. ENZO – Natural Product library

Plate number	Plate Location	Name	IUPAC	MW	CAS	Catalog number	Conc	Solvent	Plate part number	Plate description
**1**	1-E08	Etoposide	(10R,11R,15R,16S)-16-({7,8-dihydroxy-2-methyl-hexahydro-2H-pyrano[3,2-d][1,3]dioxin-6-yl}oxy)-10-(4-hydroxy-3,5-dimethoxyphenyl)-4,6,13-trioxatetracyclo[7.7.0.0^{3,7}.0^{11,15}]hexadeca-1,3(7),8-trien-12-one	588,6	33419-42-0	GR-307	2mg/ml	DMSO	2865	Natural Products Library
**1**	1-E09	Forskolin	(3R,4aR,5S,6S,6aS,10R,10aR,10bS)-3-ethenyl-6,10,10b-trihydroxy-3,4a,7,7,10a-pentamethyl-1-oxo-dodecahydro-1H-naphtho[2,1-b]pyran-5-yl acetate	410,5	66575-29-9	CN-100	2mg/ml	DMSO	2865	Natural Products Library
**1**	1-E10	Fumagillin	(2Z,4E,6E,8E)-10-{[(3R,4S,5S)-5-methoxy-4-[(2R,3R)-2-methyl-3-(3-methylbut-2-en-1-yl)oxiran-2-yl]-1-oxaspiro[2.5]octan-6-yl]oxy}-10-oxodeca-2,4,6,8-tetraenoic acid	458,5	23110-15-8	CT-100	2mg/ml	DMSO	2865	Natural Products Library
**1**	1-E11	Fumonisin B2	(2R)-2-(2-{[(5R,6R,7S,9S,16R,18S,19S)-19-amino-6-{[(3R)-3,4-dicarboxybutanoyl]oxy}-16,18-dihydroxy-5,9-dimethylicosan-7-yl]oxy}-2-oxoethyl)butanedioic acid	705,8	116355-84-1	SL-219	2mg/ml	DMSO	2865	Natural Products Library
**1**	1-E12	Galanthamine·HBr	(1S,12S,14R)-9-methoxy-4-methyl-11-oxa-4-azatetracyclo[8.6.1.0^{1,12}.0^{6,17}]heptadeca-6,8,10(17),15-tetraen-14-ol hydrobromide	368,3	1953-04-4	C-115	2mg/ml	DMSO	2865	Natural Products Library
**1**	1-F01	Gambogic acid	(2Z)-4-[(1R,2S,8R,19R)-12-hydroxy-8,21,21-trimethyl-5-(3-methylbut-2-en-1-yl)-8-(4-methylpent-3-en-1-yl)-14,18-dioxo-3,7,20-trioxahexacyclo[15.4.1.0^{2,15}.0^{2,19}.0^{4,13}.0^{6,11}]docosa-4,6(11),9,12,15-pentaen-19-yl]-2-methylbut-2-enoic acid	628,8	2752-65-0	AP-305	2mg/ml	DMSO	2865	Natural Products Library
**1**	1-F02	Genistein	5,7-dihydroxy-3-(4-hydroxyphenyl)-4H-chromen-4-one	270,2	446-72-0	ALX-350-006	2mg/ml	DMSO	2865	Natural Products Library
**1**	1-F03	Streptozocin	3-methyl-3-nitroso-1-[(2S,5S)-2,4,5-trihydroxy-6-(hydroxymethyl)oxan-3-yl]urea	265,2	18883-66-4	ALX-380-010	2mg/ml	DMSO	2865	Natural Products Library
**1**	1-F04	Gingerol	(5S)-5-hydroxy-1-(4-hydroxy-3-methoxyphenyl)decan-3-one	294,4	23513-14-6	CA-422	2mg/ml	DMSO	2865	Natural Products Library
**1**	1-F05	Ginkgolide B	(3S,6R,7S,8S,11R,12R,13S,16S,17R)-8-tert-butyl-6,12,17-trihydroxy-16-methyl-2,4,14,19-tetraoxahexacyclo[8.7.2.0^{1,11}.0^{3,7}.0^{7,11}.0^{13,17}]nonadecane-5,15,18-trione	424,4	15291-77-7	L-135	2mg/ml	DMSO	2865	Natural Products Library
**1**	1-F06	Gliotoxin	(1R,7S,8S,11R)-7-hydroxy-11-(hydroxymethyl)-15-methyl-12,13-dithia-9,15-diazatetracyclo[9.2.2.0^{1,9}.0^{3,8}]pentadeca-3,5-diene-10,14-dione	326,4	67-99-2	PI-129	2mg/ml	DMSO	2865	Natural Products Library
**1**	1-F07	Gossypol	7-[8-formyl-1,6,7-trihydroxy-3-methyl-5-(propan-2-yl)naphthalen-2-yl]-2,3,8-trihydroxy-6-methyl-4-(propan-2-yl)naphthalene-1-carbaldehyde	518,6	303-45-7	EI-130	2mg/ml	DMSO	2865	Natural Products Library
**1**	1-F08	Grayanotoxin III	(1S,3R,4R,6S,8S,9R,10R,16R)-5,5,9,14-tetramethyltetracyclo[11.2.1.0^{1,10}.0^{4,8}]hexadecane-3,4,6,9,14,16-hexol; acetaldehyde	414,5	4678-45-9	NA-135	2mg/ml	DMSO	2865	Natural Products Library

Table S2. ENZO – Natural Product library

Plate number	Plate Location	Name	IUPAC	MW	CAS	Catalog number	Conc	Solvent	Plate part number	Plate description
**1**	1-F09	Himbacine	(3S,3aR,4R,4aS,8aR,9aS)-4-[(E)-2-[(2R,6S)-1,6-dimethylpiperidin-2-yl]ethenyl]-3-methyl-dodecahydronaphtho[2,3-c]furan-1-one	345,5	6879-74-9	C-116	2mg/ml	DMSO	2865	Natural Products Library
**1**	1-F10	(-)-Huperazine	(1R,13E)-1-amino-13-ethylidene-11-methyl-6-azatricyclo[7.3.1.$0^{2,7}$]trideca-2(7),3,10-trien-5-one	242,3	102518-79-6	C-117	2mg/ml	DMSO	2865	Natural Products Library
**1**	1-F11	10-Hydroxycamptothecin	(19S)-19-ethyl-8,19-dihydroxy-17-oxa-3,13-diazapentacyclo[11.8.0.$0^{2,11}$.$0^{4,9}$.$0^{15,20}$]henicosa-1(21),2,4,6,8,10,15(20)-heptaene-14,18-dione	364,4	64439-81-2	GR-316	2mg/ml	DMSO	2865	Natural Products Library
**1**	1-F12	Hypericin	5,7,11,18,22,24-hexahydroxy-13,16-dimethyloctacyclo[13.11.1.$1^{2,10}$.$0^{3,8}$.$0^{4,25}$.$0^{19,27}$.$0^{21,26}$.$0^{14,28}$]octacosa-1,3,5,7,10(28),11,13,15,17,19(27),21(26),22,24-tridecaene-9,20-dione	504,4	548-04-9	EI-226	2mg/ml	DMSO	2865	Natural Products Library
**1**	1-G01	Indirubin	2-(2-oxo-2,3-dihydro-1H-indol-3-ylidene)-2,3-dihydro-1H-indol-3-one	262,3	479-41-4	CC-206	2mg/ml	DMSO	2865	Natural Products Library
**1**	1-G02	Ingenol 3,20-dibenzoate	[(1S,4R,5S,6R,9S,10R,12R,14R)-3-(benzoyloxy)-4,5,6-trihydroxy-11,11,14-trimethyl-15-oxotetracyclo[7.5.1.$0^{1,5}$.$0^{10,12}$]pentadeca-2,7-dien-7-yl]methyl benzoate	558,6	59086-90-7	PE-186	2mg/ml	DMSO	2865	Natural Products Library
**1**	1-G03	Isotetrandrine	(1S,14R)-9,20,21,25-tetramethoxy-15,30-dimethyl-7,23-dioxa-15,30-diazaheptacyclo[22.6.2.$2^{3,6}$.$1^{8,12}$.$1^{14,18}$.$0^{27,31}$.$0^{22,33}$]hexatriaconta-3,5,8(34),9,11,18(33),19,21,24(32),25,27(31),35-dodecaene	622,7	477-57-6	G-520	2mg/ml	DMSO	2865	Natural Products Library
**1**	1-G04	Jervine	(2'R,3S,3'R,3'aS,6'S, 6aS,6bS,7'aR,11aS,11bR)-2,3,3'a,4,4',5',6,6',6a, 6b,7,7',7'a,8,11a,11b-Hexadecahydro- 3-hydroxy- 3',6',10,11b- tetramethyl-spiro [9H-benzo [a] fluorene- 9,2'(3'H)- furo [3,2-b] pyridin]- 11(1H)- one	425,6	469-59-0	GR-337	2mg/ml	DMSO	2865	Natural Products Library
**1**	1-G05	Kainic acid	(2S,3S,4S)-3-(carboxymethyl)-4-(prop-1-en-2-yl)pyrrolidine-2-carboxylic acid	213,2	487-79-6	EA-123	2mg/ml	DMSO	2865	Natural Products Library
**1**	1-G06	(±)-Kavain	4-methoxy-6-[(E)-2-phenylethenyl]-5,6-dihydro-2H-pyran-2-one	230,3	500-64-1	NA-136	2mg/ml	DMSO	2865	Natural Products Library
**1**	1-G07	Kenpaulone	14-bromo-8,18-diazatetracyclo[9.7.0.$0^{2,7}$.$0^{12,17}$]octadeca-1(11),2(7),3,5,12(17),13,15-heptaen-9-one	327,2	142273-20-9	EI-310	2mg/ml	DMSO	2865	Natural Products Library
**1**	1-G08	β-Lapachone	2,2-dimethyl-2H,3H,4H,5H,6H-naphtho[1,2-b]pyran-5,6-dione	242,3	4707-32-8	GR-308	2mg/ml	DMSO	2865	Natural Products Library
**1**	1-G09	Lincomycin	(2S,4R)-N-[(1R,2R)-2-hydroxy-1-[(2R,3R,4S,5R,6R)-3,4,5-trihydroxy-6-(methylsulfanyl)oxan-2-yl]propyl]-1-methyl-4-propylpyrrolidine-2-carboxamide	406,5	154-21-2	A-240	2mg/ml	DMSO	2865	Natural Products Library

Table S2. ENZO – Natural Product library

Plate number	Plate Location	Name	IUPAC	MW	CAS	Catalog number	Conc	Solvent	Plate part number	Plate description
1	1-G10	Lycorine	(1S,17S,18S,19S)-5,7-dioxa-12-azapentacyclo[10.6.1.0^{2},^{10}.0^{4},^{8}.0^{15},^{19}]nonadeca-2,4(8),9,15-tetraene-17,18-diol	287,3	476-28-8	GR-313	2mg/ml	DMSO	2865	Natural Products Library
1	1-G11	Mevastatin	(1S,7S,8S,8aR)-8-{2-[(2R,4R)-4-hydroxy-6-oxooxan-2-yl]ethyl}-7-methyl-1,2,3,7,8,8a-hexahydronaphthalen-1-yl (2S)-2-methylbutanoate	390,5	73573-88-3	G-233	2mg/ml	DMSO	2865	Natural Products Library
1	1-G12	3-Beta-Indoleacrylic acid	(2E)-3-(1H-indol-3-yl)prop-2-enoic acid	187,2	1204-06-4	NP-565	2mg/ml	DMSO	2865	Natural Products Library
1	1-H01	L-Mimosine	(2S)-2-amino-3-(3-hydroxy-4-oxo-1,4-dihydropyridin-1-yl)propanoic acid	198,2	500-44-7	CC-102	2mg/ml	DMSO	2865	Natural Products Library
1	1-H02	Mithramycin A	(2S,3S)-3-[(1S,3S,4R)-3,4-dihydroxy-1-methoxy-2-oxopentyl]-2-{[4-({4-[(4,5-dihydroxy-4,6-dimethyloxan-2-yl)oxy]-5-hydroxy-6-methyloxan-2-yl}oxy)-5-hydroxy-6-methyloxan-2-yl]oxy}-6-({4-[(4,5-dihydroxy-6-methyloxan-2-yl)oxy]-5-hydroxy-6-methyloxan-2-yl}oxy)-8,9-dihydroxy-7-methyl-1,2,3,4-tetrahydroanthracen-1-one	1085,1	18378-89-7	GR-305	2mg/ml	DMSO	2865	Natural Products Library
1	1-H03	Monensin	sodium (3R,4S)-4-[(2S,5R,7S,8R,9S)-2-[(2R,5S)-5-ethyl-5-[(2R,3S,5R)-5-[(2S,3S,5R,6R)-6-hydroxy-6-(hydroxymethyl)-3,5-dimethyloxan-2-yl]-3-methyloxolan-2-yl]oxolan-2-yl]-9-hydroxy-2,8-dimethyl-1,6-dioxaspiro[4.5]decan-7-yl]-3-methoxy-2-methylpentanoate	692,9	22373-78-0	A-248	2mg/ml	DMSO	2865	Natural Products Library
1	1-H04	Mycophenolic acid	(4E)-6-(4-hydroxy-6-methoxy-7-methyl-3-oxo-1,3-dihydro-2-benzofuran-5-yl)-4-methylhex-4-enoic acid	320,3	24280-93-1	A-249	2mg/ml	DMSO	2865	Natural Products Library
1	1-H05	Myriocin	(2S,3R,4R,6E)-2-amino-3,4-dihydroxy-2-(hydroxymethyl)-14-oxoicos-6-enoic acid	401,5	35891-70-4	SL-226	2mg/ml	DMSO	2865	Natural Products Library
1	1-H06	Neomycin	(2R,3S,4R,5R,6R)-5-amino-2-(aminomethyl)-6-{[(1R,2R,3S,4R,6S)-4,6-diamino-2-{[(2S,3R,4S,5R)-4-{[(3R,4R,5S,6S)-3-amino-6-(aminomethyl)-4,5-dihydroxyoxan-2-yl]oxy}-3-hydroxy-5-(hydroxymethyl)oxolan-2-yl]oxy}-3-hydroxycyclohexyl]oxy}oxane-3,4-diol	614,6	1405-10-3	EI-180	2mg/ml	DMSO	2865	Natural Products Library
1	1-H07	Nigericin·Na	sodium (2R)-2-[(2R,3S,6R)-6-{[(2S,4R,5R,7R,9R,10R)-2-[(2R,5S)-5-[(2R,3S,5R)-5-[(2S,3S,5R,6R)-6-hydroxy-6-(hydroxymethyl)-3,5-dimethyloxan-2-yl]-3-methyloxolan-2-yl]-5-methyloxolan-2-yl]-9-methoxy-2,4,10-trimethyl-1,6-dioxaspiro[4.5]decan-7-yl]methyl}-3-methyloxan-2-yl]propanoate	746,9	28380-24-7	CA-421	2mg/ml	DMSO	2865	Natural Products Library
1	1-H08	Oligomycin A	(1S,4E,5'R,6R,6'R,7S,8R,10S,11S,12R,14S,15R,16S,18E,20E,22S,25R,27S,28R,29S)-22-ethyl-7,11,14,15-tetrahydroxy-6'-[(2S)-2-hydroxypropyl]-5',6,8,10,12,14,16,28,29-nonamethyl-2,26-dioxaspiro[bicyclo[23.3.1]nonacosane-27,2'-oxane]-4,18,20-triene-3,9,13-trione	791,1	579-13-5	CM-111	2mg/ml	DMSO	2865	Natural Products Library

Table S2. ENZO – Natural Product library

Plate number	Plate Location	Name	IUPAC	MW	CAS	Catalog number	Conc	Solvent	Plate part number	Plate description
1	1-H09	(-)-Ouabain	4-[(1S,2R,3R,5S,7S,10R,11S,14R,15R,17R)-3,7,11,17-tetrahydroxy-2-(hydroxymethyl)-15-methyl-5-{[(2R,3R,4R,5R,6S)-3,4,5-trihydroxy-6-methyloxan-2-yl]oxy}tetracyclo[8.7.0.0^{2,7}.0^{11,15}]heptadecan-14-yl]-2,5-dihydrofuran-2-one	584,7	11018-89-6	CM-109	2mg/ml	DMSO	2865	Natural Products Library
1	1-H10	Parthenolide	(1S,2R,4R,7E,11S)-4,8-dimethyl-12-methylidene-3,14-dioxatricyclo[9.3.0.0^{2,4}]tetradec-7-en-13-one	248,3	20554-84-1	T-113	2mg/ml	DMSO	2865	Natural Products Library
1	1-H11	Perillic acid	(4S)-4-(prop-1-en-2-yl)cyclohex-1-ene-1-carboxylic acid	166,2	7694-45-3	G-210	2mg/ml	DMSO	2865	Natural Products Library
1	1-H12	Phloretin	3-(4-hydroxyphenyl)-1-(2,4,6-trihydroxyphenyl)propan-1-one	274,3	60-82-2	EI-154	2mg/ml	DMSO	2865	Natural Products Library
2	2-A01	Phorbol 12,13-dibutyrate	(1S,2S,6R,10S,11R,13S,14R,15R)-14-(butanoyloxy)-1,6-dihydroxy-8-(hydroxymethyl)-4,12,12,15-tetramethyl-5-oxotetracyclo[8.5.0.0^{2,6}.0^{11,13}]pentadeca-3,8-dien-13-yl butanoate	504,6	37558-16-0	PE-135	2mg/ml	DMSO	2865	Natural Products Library
2	2-A02	(-)-Guaiol	2-[(3S,5R,8S)-3,8-dimethyl-1,2,3,4,5,6,7,8-octahydroazulen-5-yl]propan-2-ol	222,4	489-86-1	NP-531	2mg/ml	DMSO	2865	Natural Products Library
2	2-A03	Phorbol 12-myristate 13-acetate	(1S,2S,6R,10S,11R,13S,14R,15R)-13-(acetyloxy)-1,6-dihydroxy-8-(hydroxymethyl)-4,12,12,15-tetramethyl-5-oxotetracyclo[8.5.0.0^{2,6}.0^{11,13}]pentadeca-3,8-dien-14-yl tetradecanoate	616,8	16561-29-8	PE-160	2mg/ml	DMSO	2865	Natural Products Library
2	2-A04	4α-Phorbol 12-myristate 13-acetate	(1S,2S,6S,10S,11R,13S,14R,15R)-13-(acetyloxy)-1,6-dihydroxy-8-(hydroxymethyl)-4,12,12,15-tetramethyl-5-oxotetracyclo[8.5.0.0^{2,6}.0^{11,13}]pentadeca-3,8-dien-14-yl tetradecanoate	616,8	63597-44-4	PE-162	2mg/ml	DMSO	2865	Natural Products Library
2	2-A05	Phytosphingosine	(2S,3S,4R)-2-aminooctadecane-1,3,4-triol	317,5	554-62-1	SL-150	2mg/ml	DMSO	2865	Natural Products Library
2	2-A06	Piceatannol	5-[(E)-2-(3,4-dihydroxyphenyl)ethenyl]benzene-1,3-diol	244,2	10083-24-6	EI-271	2mg/ml	DMSO	2865	Natural Products Library
2	2-A07	Prostaglandin A_1	7-[(1R,2S)-2-[(1E,3S)-3-hydroxyoct-1-en-1-yl]-5-oxocyclopent-3-en-1-yl]heptanoic acid	336,5	14152-28-4	PG-001	2mg/ml	DMSO	2865	Natural Products Library
2	2-A08	Prostaglandin B_1	7-{2-[(1E,3S)-3-hydroxyoct-1-en-1-yl]-5-oxocyclopent-1-en-1-yl}heptanoic acid	336,5	13345-51-2	PG-003	2mg/ml	DMSO	2865	Natural Products Library
2	2-A09	Prostaglandin E_1	7-[(1R,2R,3R)-3-hydroxy-2-[(1E,3S)-3-hydroxyoct-1-en-1-yl]-5-oxocyclopentyl]heptanoic acid	354,5	745-65-3	PG-006	2mg/ml	DMSO	2865	Natural Products Library
2	2-A10	Prostaglandin E_2	(5Z)-7-[(1R,2R,3R)-3-hydroxy-2-[(1E,3S)-3-hydroxyoct-1-en-1-yl]-5-oxocyclopentyl]hept-5-enoic acid	352,5	363-24-6	PG-007	2mg/ml	DMSO	2865	Natural Products Library
2	2-A11	Prostaglandin $F_{2\alpha}$	(5Z)-7-[(1R,2R,3R,5S)-3,5-dihydroxy-2-[(1E,3S)-3-hydroxyoct-1-en-1-yl]cyclopentyl]hept-5-enoic acid	354,5	38562-01-5	PG-008	2mg/ml	DMSO	2865	Natural Products Library
2	2-A12	Kahweol Acetate	[(1S,4S,12S,13R,16R,17R)-17-hydroxy-12-methyl-8-oxapentacyclo[14.2.1.0^{1,13}.0^{4,12}.0^{5,9}]nonadeca-5(9),6,10-trien-17-yl]methyl acetate	356,5	81760-47-6	NP-577	2mg/ml	DMSO	2865	Natural Products Library
2	2-B01	Quisqualic acid	(2S)-2-amino-3-(3,5-dioxo-1,2,4-oxadiazolidin-2-yl)propanoic acid	189,1	52809-07-1	EA-132	2mg/ml	DMSO	2865	Natural Products Library

Table S2. ENZO – Natural Product library

Plate number	Plate Location	Name	IUPAC	MW	CAS	Catalog number	Conc	Solvent	Plate part number	Plate description
**2**	2-B02	Radicicol	(4R,6R,8R,9Z,11E)-16-chloro-17,19-dihydroxy-4-methyl-3,7-dioxatricyclo[13.4.0.$0^{6,8}$]nonadeca-1(15),9,11,16,18-pentaene-2,13-dione	364,8	12772-57-5	EI-285	2mg/ml	DMSO	2865	Natural Products Library
**2**	2-B03	Rapamycin	(1R,9S,15R,16E,18R,19R,21R,23S,24E,26E,28E,30S,32S,35R)-1,18-dihydroxy-12-[(2R)-1-[(1R,3S,4S)-4-hydroxy-3-methoxycyclohexyl]propan-2-yl]-19,30-dimethoxy-15,17,21,23,29,35-hexamethyl-11,36-dioxa-4-azatricyclo[30.3.1.$0^{4,9}$]hexatriaconta-16,24,26,28-tetraene-2,3,10,14,20-pentone	914,2	53123-88-9	A-275	2mg/ml	DMSO	2865	Natural Products Library
**2**	2-B04	Rauwolscine	methyl (1S,15S,18S,19S,20S)-18-hydroxy-3,13-diazapentacyclo[11.8.0.$0^{2,10}$.$0^{4,9}$.$0^{15,20}$]henicosa-2(10),4(9),5,7-tetraene-19-carboxylate hydrochloride	390,9	6211-32-1	AR-106	2mg/ml	DMSO	2865	Natural Products Library
**2**	2-B05	Resveratrol	5-[(E)-2-(4-hydroxyphenyl)ethenyl]benzene-1,3-diol	228,2	501-36-0	FR-104	2mg/ml	DMSO	2865	Natural Products Library
**2**	2-B06	All trans retinoic acid	(2E,4E,6E,8E)-3,7-dimethyl-9-(2,6,6-trimethylcyclohex-1-en-1-yl)nona-2,4,6,8-tetraenoic acid	300,4	302-79-4	GR-100	2mg/ml	DMSO	2865	Natural Products Library
**2**	2-B07	13-cis-Retinoic acid	(2Z,4E,6E,8E)-3,7-dimethyl-9-(2,6,6-trimethylcyclohex-1-en-1-yl)nona-2,4,6,8-tetraenoic acid	300,4	4759-48-2	GR-102	2mg/ml	DMSO	2865	Natural Products Library
**2**	2-B08	9-cis-Retinoic acid	(2E,4E,6Z,8E)-3,7-dimethyl-9-(2,6,6-trimethylcyclohex-1-en-1-yl)nona-2,4,6,8-tetraenoic acid	300,4	5300-03-8	GR-101	2mg/ml	DMSO	2865	Natural Products Library
**2**	2-B09	Rifampicin	(7S,9E,11S,12R,13S,14R,15R,16R,17S,18S,19E,21Z)-2,15,17,27,29-pentahydroxy-11-methoxy-3,7,12,14,16,18,22-heptamethyl-26-[(E)-N-(4-methylpiperazin-1-yl)carboximidoyl]-6,23-dioxo-8,30-dioxa-24-azatetracyclo[23.3.1.$1^{4,7}$.$0^{5,28}$]triaconta-1,3,5(28),9,19,21,25(29),26-octaen-13-yl acetate	822,9	13292-46-1	GR-306	2mg/ml	DMSO	2865	Natural Products Library
**2**	2-B10	Rosmarinic acid	(2R)-3-(3,4-dihydroxyphenyl)-2-{[(2E)-3-(3,4-dihydroxyphenyl)prop-2-enoyl]oxy}propanoic acid	360,3	20283-92-5	EI-291	2mg/ml	DMSO	2865	Natural Products Library
**2**	2-B11	Rotenone	(1S,6R,13S)-16,17-dimethoxy-6-(prop-1-en-2-yl)-2,7,20-trioxapentacyclo[11.8.0.$0^{3,11}$.$0^{4,8}$.$0^{14,19}$]henicosa-3(11),4(8),9,14(19),15,17-hexaen-12-one	394,4	83-79-4	ALX-350-360	2mg/ml	DMSO	2865	Natural Products Library
**2**	2-B12	Rottlerin	(2E)-1-{6-[(3-acetyl-2,4,6-trihydroxy-5-methylphenyl)methyl]-5,7-dihydroxy-2,2-dimethyl-2H-chromen-8-yl}-3-phenylprop-2-en-1-one	516,5	82-08-6	ALX-350-075	2mg/ml	DMSO	2865	Natural Products Library
**2**	2-C01	Ryanodine	(1R,2R,3S,6S,7S,9S,10R,11R,12R,13S,14R)-2,6,9,11,13,14-hexahydroxy-3,7,10-trimethyl-11-(propan-2-yl)-15-oxapentacyclo[7.5.1.$0^{1,6}$.$0^{7,13}$.$0^{10,14}$]pentadecan-12-yl 1H-pyrrole-2-carboxylate	493,5	15662-33-6	CA-450	2mg/ml	DMSO	2865	Natural Products Library
**2**	2-C02	Shikonin	5,8-dihydroxy-2-[(1R)-1-hydroxy-4-methylpent-3-en-1-yl]-1,4-dihydronaphthalene-1,4-dione	288,3	517-89-5	CT-115	2mg/ml	DMSO	2865	Natural Products Library
**2**	2-C03	Spectinomycin	(1R,3S,5R,8R,10R,11S,12S,13R,14S)-8,12,14-trihydroxy-5-methyl-11,13-bis(methylamino)-2,4,9-trioxatricyclo[8.4.0.$0^{3,8}$]tetradecan-7-one	332,3	1695-77-8	A-281	2mg/ml	DMSO	2865	Natural Products Library

Table S2. ENZO – Natural Product library

Plate number	Plate Location	Name	IUPAC	MW	CAS	Catalog number	Conc	Solvent	Plate part number	Plate description
2	2-C04	Swainsonine	(1S,2R,8R,8aR)-octahydroindolizine-1,2,8-triol	173,2	72741-87-8	S-112	2mg/ml	DMSO	2865	Natural Products Library
2	2-C05	Tanshinone IIA	6,6,14-trimethyl-12-oxatetracyclo[8.7.0.$0^{2,7}$.$0^{11,15}$]heptadeca-1(10),2(7),8,11(15),13-pentaene-16,17-dione	294,3	568-73-0	GR-336	2mg/ml	DMSO	2865	Natural Products Library
2	2-C06	Taxol	(1S,2S,4S,7R,9S,10S,12R,15S)-4,12-bis(acetyloxy)-1,9-dihydroxy-15-{[(2R,3S)-2-hydroxy-3-phenyl-3-(phenylformamido)propanoyl]oxy}-10,14,17,17-tetramethyl-11-oxo-6-oxatetracyclo[11.3.1.$0^{3,10}$.$0^{4,7}$]heptadec-13-en-2-yl benzoate	853,9	33069-62-6	T-104	2mg/ml	DMSO	2865	Natural Products Library
2	2-C07	Tetrandrine	(1S,14S)-9,20,21,25-tetramethoxy-15,30-dimethyl-7,23-dioxa-15,30-diazaheptacyclo[22.6.2.$2^{3,6}$.$1^{8,12}$.$1^{14,18}$.$0^{27,31}$.$0^{22,33}$]hexatriaconta-3,5,8(34),9,11,18(33),19,21,24(32),25,27(31),35-dodecaene	622,7	518-34-3	CA-260	2mg/ml	DMSO	2865	Natural Products Library
2	2-C08	Thapsigargin	(3S,3aR,4S,6S,6aR,7S,8S,9bS)-6-(acetyloxy)-4-(butanoyloxy)-3,3a-dihydroxy-3,6,9-trimethyl-8-{[(2Z)-2-methylbut-2-enoyl]oxy}-2-oxo-2H,3H,3aH,4H,5H,6H,6aH,7H,8H,9bH-azuleno[4,5-b]furan-7-yl octanoate	650,8	67526-95-8	PE-180	2mg/ml	DMSO	2865	Natural Products Library
2	2-C09	Tomatidine	(1R,2S,4S,5'S,6S,7S,8R,9S,12S,13S,16S,18S)-5',7,9,13-tetramethyl-5-oxaspiro[pentacyclo[10.8.0.$0^{2,9}$.$0^{4,8}$.$0^{13,18}$]icosane-6,2'-piperidine]-16-ol	415,7	77-59-8	GR-335	2mg/ml	DMSO	2865	Natural Products Library
2	2-C10	Troleandomycin	(3S,5R,6S,7S,8R,11S,12S,13R,14S,15S)-14-{[3-(acetyloxy)-4-(dimethylamino)-6-methyloxan-2-yl]oxy}-12-{[5-(acetyloxy)-4-methoxy-6-methyloxan-2-yl]oxy}-5,7,8,11,13,15-hexamethyl-4,10-dioxo-1,9-dioxaspiro[2.13]hexadecan-6-yl acetate	814,0	2751-09-9	EI-249	2mg/ml	DMSO	2865	Natural Products Library
2	2-C11	Tunicamycin B	(2Z)-N-(6-{2-[5-(2,4-dioxo-1,2,3,4-tetrahydropyrimidin-1-yl)-3,4-dihydroxyoxolan-2-yl]-2-hydroxyethyl}-2-{[3-acetamido-4,5-dihydroxy-6-(hydroxymethyl)oxan-2-yl]oxy}-4,5-dihydroxyoxan-3-yl)-13-methyltetradec-2-enamide	830,9	11089-65-9	CC-104	2mg/ml	DMSO	2865	Natural Products Library
2	2-C12	Ursolic acid	(1S,2R,4aS,6aS,6bR,8aR,10S,12aR,12bR,14bS)-10-hydroxy-1,2,6a,6b,9,9,12a-heptamethyl-1,2,3,4,4a,5,6,6a,6b,7,8,8a,9,10,11,12,12a,12b,13,14b-icosahydropicene-4a-carboxylic acid	456,7	77-52-1	CT-105	2mg/ml	DMSO	2865	Natural Products Library
2	2-D01	Valinomycin	(3S,6S,9R,12R,15S,18S,21R,24R,27S,30S,33R,36R)-6,18,30-trimethyl-3,9,12,15,21,24,27,33,36-nonakis(propan-2-yl)-1,7,13,19,25,31-hexaoxa-4,10,16,22,28,34-hexaazacyclohexatriacontane-2,5,8,11,14,17,20,23,26,29,32,35-dodecone	1111,3	2001-95-8	KC-140	2mg/ml	DMSO	2865	Natural Products Library

Table S2. ENZO – Natural Product library

Plate number	Plate Location	Name	IUPAC	MW	CAS	Catalog number	Conc	Solvent	Plate part number	Plate description
**2**	2-D02	Aconitine	(1S,2R,3R,4R,5R,6S,7S,8R,10R,13R,14R,16S,17S,18R)-8-(acetyloxy)-11-ethyl-5,7,14-trihydroxy-6,16,18-trimethoxy-13-(methoxymethyl)-11-azahexacyclo[7.7.2.1^{2,5}.0^{1,10}.0^{3,8}.0^{13,17}]nonadecan-4-yl benzoate	645,7	302-27-2	AC-126	2mg/ml	DMSO	2865	Natural Products Library
**2**	2-D03	Veratridine	(1R,2S,6S,9S,10R,11S,12S,14R,15S,18S,19S,22S,23S,25R)-1,10,11,12,14,23-hexahydroxy-6,10,19-trimethyl-24-oxa-4-azaheptacyclo[12.12.0.0^{2,11}.0^{4,9}.0^{15,25}.0^{18,23}.0^{19,25}]hexacosan-22-yl 3,4-dimethoxybenzoate	673,8	71-62-5	NA-125	2mg/ml	DMSO	2865	Natural Products Library
**2**	2-D04	Vinblastine sulfate	sulfuric acid methyl (1R,9R,10S,11R,12R,19R)-11-(acetyloxy)-12-ethyl-4-[(1S,14S,15R)-17-ethyl-17-hydroxy-13-(methoxycarbonyl)-1,11-diazatetracyclo[13.3.1.0^{4,12}.0^{5,10}]nonadeca-4(12),5(10),6,8-tetraen-14-yl]-10-hydroxy-5-methoxy-8-methyl-8,16-diazapentacyclo[10.6.1.0^{1,9}.0^{2,7}.0^{16,19}]nonadeca-2(7),3,5,13-tetraene-10-carboxylate	909,1	143-67-9	T-116	2mg/ml	DMSO	2865	Natural Products Library
**2**	2-D05	Vincristine sulfate	sulfuric acid methyl (1R,9R,11R,12R,19R)-11-(acetyloxy)-12-ethyl-4-[(1S,13R,14S,15R)-17-ethyl-17-hydroxy-13-(methoxycarbonyl)-1,11-diazatetracyclo[13.3.1.0^{4,12}.0^{5,10}]nonadeca-4(12),5(10),6,8-tetraen-14-yl]-8-formyl-10-hydroxy-5-methoxy-8,16-diazapentacyclo[10.6.1.0^{1,9}.0^{2,7}.0^{16,19}]nonadeca-2(7),3,5,13-tetraene-10-carboxylate	923,0	2068-78-2	T-117	2mg/ml	DMSO	2865	Natural Products Library
**2**	2-D06	Vinpocetin	ethyl (15S,19S)-15-ethyl-1,11-diazapentacyclo[9.6.2.0^{2,7}.0^{8,18}.0^{15,19}]nonadeca-2(7),3,5,8(18),16-pentaene-17-carboxylate	350,5	42971-09-5	PD-185	2mg/ml	DMSO	2865	Natural Products Library
**2**	2-D07	Wedelolactone	3,13,14-trihydroxy-5-methoxy-8,17-dioxatetracyclo[8.7.0.0^{2,7}.0^{11,16}]heptadeca-1(10),2,4,6,11(16),12,14-heptaen-9-one	314,2	524-12-9	EI-316	2mg/ml	DMSO	2865	Natural Products Library
**2**	2-D08	Wortmannin	(1R,3R,5S,9R,18S)-18-(methoxymethyl)-1,5-dimethyl-6,11,16-trioxo-13,17-dioxapentacyclo[10.6.1.0^{2,10}.0^{5,9}.0^{15,19}]nonadeca-2(10),12(19),14-trien-3-yl acetate	428,4	19545-26-7	ST-415	2mg/ml	DMSO	2865	Natural Products Library
**2**	2-D09	Apigenin	5,7-dihydroxy-2-(4-hydroxyphenyl)-4H-chromen-4-one	270,2	520-36-5	EI-345	2mg/ml	DMSO	2865	Natural Products Library
**2**	2-D10	Arecoline·HBr	methyl 1-methyl-1,2,5,6-tetrahydropyridine-3-carboxylate hydrobromide	236,1	300-08-3	AC-784	2mg/ml	DMSO	2865	Natural Products Library
**2**	2-D11	Atropine sulfate	sulfuric acid bis(8-methyl-8-azabicyclo[3.2.1]octan-3-yl 3-hydroxy-2-phenylpropanoate) hydrate	694,8	55-48-1	AC-735	2mg/ml	DMSO	2865	Natural Products Library

Table S2. ENZO – Natural Product library

Plate number	Plate Location	Name	IUPAC	MW	CAS	Catalog number	Conc	Solvent	Plate part number	Plate description
**2**	2-D12	Berbamine·2HCl	(1S,14R)-20,21,25-trimethoxy-15,30-dimethyl-7,23-dioxa-15,30-diazaheptacyclo[22.6.2.2^{3,6}.1^{8,12}.1^{14,18}.0^{27,31}.0^{22,33}]hexatriaconta-3,5,8(34),9,11,18(33),19,21,24(32),25,27(31),35-dodecaen-9-ol dihydrochloride	681,6	6078-17-7	NP-403	2mg/ml	DMSO	2865	Natural Products Library
**2**	2-E01	(+)-Bicuculline	(10R)-10-[(5S)-6-methyl-2H,5H,6H,7H,8H-[1,3]dioxolo[4,5-g]isoquinolin-5-yl]-3,5,11-trioxatricyclo[7.3.0.0^{2,6}]dodeca-1(9),2(6),7-trien-12-one	367,4	485-49-4	NP-031	2mg/ml	DMSO	2865	Natural Products Library
**2**	2-E02	Bufalin	5-[(1S,2S,5S,7R,10R,11R,14R,15R)-5,11-dihydroxy-2,15-dimethyltetracyclo[8.7.0.0^{2,7}.0^{11,15}]heptadecan-14-yl]-2H-pyran-2-one	386,5	465-21-4	AP-303	2mg/ml	DMSO	2865	Natural Products Library
**2**	2-E03	Brucine-N-oxide	(1R,11S,18S,20R,21R,22S)-4,5-dimethoxy-12-oxa-8,17-diazaheptacyclo[15.5.2.0^{1,18}.0^{2,7}.0^{8,22}.0^{11,21}.0^{15,20}]tetracosa-2(7),3,5,14-tetraen-9-one	394,5	357-57-3	NP-038	2mg/ml	DMSO	2865	Natural Products Library
**2**	2-E04	Butein	(2E)-1-(2,4-dihydroxyphenyl)-3-(3,4-dihydroxyphenyl)prop-2-en-1-one	272,3	487-52-5	NP-040	2mg/ml	DMSO	2865	Natural Products Library
**2**	2-E05	Catalpol	(2S,3R,4S,5S,6R)-2-{[(1S,2S,4S,5S,6R,10S)-5-hydroxy-2-(hydroxymethyl)-3,9-dioxatricyclo[4.4.0.0^{2,4}]dec-7-en-10-yl]oxy}-6-(hydroxymethyl)oxane-3,4,5-triol	362,3	2415-24-9	NP-046	2mg/ml	DMSO	2865	Natural Products Library
**2**	2-E06	Chrysine	5,7-dihydroxy-2-phenyl-4H-chromen-4-one	254,2	480-40-0	NP-051	2mg/ml	DMSO	2865	Natural Products Library
**2**	2-E07	Desoxypeganine	1H,2H,3H,9H-pyrrolo[2,1-b]quinazoline dihydrate hydrochloride	244,7	61939-05-7	NP-073	2mg/ml	DMSO	2865	Natural Products Library
**2**	2-E08	Veratramine	(3R,5S)-2-[(1R)-1-[(3S,6aR,11aR,11bR)-3-hydroxy-10,11b-dimethyl-1H,2H,3H,4H,6H,6aH,11H,11aH,11bH-cyclohexa[a]fluoren-9-yl]ethyl]-5-methylpiperidin-3-ol	409,6	60-70-8	CA-214	2mg/ml	DMSO	2865	Natural Products Library
**2**	2-E09	Emodin	1,3,8-trihydroxy-6-methyl-9,10-dihydroanthracene-9,10-dione	270,2	518-82-1	NP-094	2mg/ml	DMSO	2865	Natural Products Library
**2**	2-E10	Gramine	(1H-indol-3-ylmethyl)dimethylamine	174,2	87-52-5	AC-913	2mg/ml	DMSO	2865	Natural Products Library
**2**	2-E11	Harmaline	7-methoxy-1-methyl-3H,4H,9H-pyrido[3,4-b]indole	214,3	304-21-2	AC-1051	2mg/ml	DMSO	2865	Natural Products Library
**2**	2-E12	Harmine	7-methoxy-1-methyl-9H-pyrido[3,4-b]indole	212,2	442-51-3	AC-1053	2mg/ml	DMSO	2865	Natural Products Library
**2**	2-F01	Hyoscyamine	8-methyl-8-azabicyclo[3.2.1]octan-3-yl (2S)-3-hydroxy-2-phenylpropanoate	289,4	101-31-5	AC-922	2mg/ml	DMSO	2865	Natural Products Library
**2**	2-F02	Ivermectin	(1'R,2R,4'S,5S,6R,8'R,10'E,13'S,14'E,16'E,20'R,21'R,24'S)-6-(butan-2-yl)-21',24'-dihydroxy-12'-{[(2R,4S,6S)-5-{[(2S,4S,5S,6S)-5-hydroxy-4-methoxy-6-methyloxan-2-yl]oxy}-4-methoxy-6-methyloxan-2-yl]oxy}-5,11',13',22'-tetramethyl-3',7',19'-trioxaspiro[oxane-2,6'-tetracyclo[15.6.1.1^{4,8}.0^{20,24}]pentacosane]-10',14',16',22'-tetraen-2'-one	875,1	70288-86-7	AC-238	2mg/ml	DMSO	2865	Natural Products Library

Table S2. ENZO – Natural Product library

Plate number	Plate Location	Name	IUPAC	MW	CAS	Catalog number	Conc	Solvent	Plate part number	Plate description
**2**	2-F03	Luteolin	2-(3,4-dihydroxyphenyl)-5,7-dihydroxy-4H-chromen-4-one	286,2	491-70-3	NP-176	2mg/ml	DMSO	2865	Natural Products Library
**2**	2-F04	Melatonin	N-[2-(5-methoxy-1H-indol-3-yl)ethyl]acetamide	232,3	73-31-4	NS-520	2mg/ml	DMSO	2865	Natural Products Library
**2**	2-F05	Morine	2-(2,4-dihydroxyphenyl)-3,5,7-trihydroxy-4H-chromen-4-one	302,2	480-16-0	NP-193	2mg/ml	DMSO	2865	Natural Products Library
**2**	2-F06	Myricetin	3,5,7-trihydroxy-2-(3,4,5-trihydroxyphenyl)-4H-chromen-4-one	318,2	529-44-2	NP-195	2mg/ml	DMSO	2865	Natural Products Library
**2**	2-F07	Naringenin	(2S)-5,7-dihydroxy-2-(4-hydroxyphenyl)-3,4-dihydro-2H-1-benzopyran-4-one	272,3	480-41-1	NP-200	2mg/ml	DMSO	2865	Natural Products Library
**2**	2-F08	(-)-Nicotine	3-[(2S)-1-methylpyrrolidin-2-yl]pyridine	162,2	54-11-5	AC-782	2mg/ml	DMSO	2865	Natural Products Library
**2**	2-F09	Nonactin	(1R,2R,5R,7R,10S,11S,14S,16S,19R,20R,23R,25R,28S,29S,32S,34S)-2,5,11,14,20,23,29,32-octamethyl-4,13,22,31,37,38,39,40-octaoxapentacyclo[32.2.1.1^{7,10}.1^{16,19}.1^{25,28}]tetracontane-3,12,21,30-tetrone	736,9	6833-84-7	NP-207	2mg/ml	DMSO	2865	Natural Products Library
**2**	2-F10	L-Penicillamine	(2R)-2-amino-3-methyl-3-sulfanylbutanoic acid	149,2	1113-41-3	NP-226	2mg/ml	DMSO	2865	Natural Products Library
**2**	2-F11	Picrotoxinin	(1R,3R,5S,8S,9R,12S,13R,14R)-1-hydroxy-13-methyl-14-(prop-1-en-2-yl)-4,7,10-trioxapentacyclo[6.4.1.1^{9,12}.0^{3,5}.0^{5,13}]tetradecane-6,11-dione	292,3	17617-45-7	NP-401	2mg/ml	DMSO	2865	Natural Products Library
**2**	2-F12	Pilocarpine	(3S,4R)-3-ethyl-4-[(1-methyl-1H-imidazol-5-yl)methyl]oxolan-2-one hydrochloride	244,7	54-71-7	AC-214	2mg/ml	DMSO	2865	Natural Products Library
**2**	2-G01	Quassin	(1S,2S,6S,7S,9R,13R,17S)-4,15-dimethoxy-2,6,14,17-tetramethyl-10-oxatetracyclo[7.7.1.0^{2,7}.0^{13,17}]heptadeca-4,14-diene-3,11,16-trione	388,5	76-78-8	NP-245	2mg/ml	DMSO	2865	Natural Products Library
**2**	2-G02	Quercetin·$2H_2O$	2-(3,4-dihydroxyphenyl)-3,5,7-trihydroxy-4H-chromen-4-one dihydrate	338,3	6151-25-3	AC-1142	2mg/ml	DMSO	2865	Natural Products Library
**2**	2-G03	Quinidine·HCl·H_2O	(S)-[(2R,5R)-5-ethenyl-1-azabicyclo[2.2.2]octan-2-yl](6-methoxyquinolin-4-yl)methanol hydrate hydrochloride	378,9	6151-40-2	AC-129	2mg/ml	DMSO	2865	Natural Products Library
**2**	2-G04	Quinine·HCl·$2H_2O$	(S)-[(1R,2S)-5-ethenyl-1-azabicyclo[2.2.2]octan-2-yl](6-methoxyquinolin-4-yl)methanol dihydrate hydrochloride	396,9	6119-47-7	AC-122	2mg/ml	DMSO	2865	Natural Products Library
**2**	2-G05	Robinetine	3,7-dihydroxy-2-(3,4,5-trihydroxyphenyl)-4H-chromen-4-one	302,2	490-31-3	NP-261	2mg/ml	DMSO	2865	Natural Products Library
**2**	2-G06	Menadione	2-methyl-1,4-dihydronaphthalene-1,4-dione	172,2	58-27-5	NP-528	2mg/ml	DMSO	2865	Natural Products Library
**2**	2-G07	Strychnine·HCl	(1R,11S,18S,20R,21R,22S)-12-oxa-8,17-diazaheptacyclo[15.5.2.0^{1,18}.0^{2,7}.0^{8,22}.0^{11,21}.0^{15,20}]tetracosa-2(7),3,5,14-tetraen-9-one hydrochloride	370,9	1421-86-9	AC-745	2mg/ml	DMSO	2865	Natural Products Library
**2**	2-G08	Tryptanthrin	6H,12H-indolo[2,1-b]quinazoline-6,12-dione	248,2	13220-57-0	NP-467	2mg/ml	DMSO	2865	Natural Products Library
**2**	2-G09	Yohimbine·HCl	methyl (1S,15R,18S,19R,20S)-18-hydroxy-3,13-diazapentacyclo[11.8.0.0^{2,10}.0^{4,9}.0^{15,20}]henicosa-2(10),4(9),5,7-tetraene-19-carboxylate hydrochloride	390,9	65-19-0	AC-161	2mg/ml	DMSO	2865	Natural Products Library

Table S2. ENZO – Natural Product library

Plate number	Plate Location	Name	IUPAC	MW	CAS	Catalog number	Conc	Solvent	Plate part number	Plate description
**2**	2-G10	(-)-Eburnamonine	(15S,19S)-15-ethyl-1,11-diazapentacyclo[9.6.2.$0^{2,7}$.$0^{8,18}$.$0^{15,19}$]nonadeca-2,4,6,8(18)-tetraen-17-one	294,4	4880-88-0	NP-088	2mg/ml	DMSO	2865	Natural Products Library
**2**	2-G11	Lysergol	[(4S,7S)-6-methyl-6,11-diazatetracyclo[7.6.1.$0^{2,7}$.$0^{12,16}$]hexadeca-1(16),2,9,12,14-pentaen-4-yl]methanol	254,3	602-85-7	NP-180	2mg/ml	DMSO	2865	Natural Products Library
**2**	2-G12	Monocrotaline	(1R,4R,5R,6R,16R)-5,6-dihydroxy-4,5,6-trimethyl-2,8-dioxa-13-azatricyclo[8.5.1.$0^{13,16}$]hexadec-10-ene-3,7-dione	325,4	315-22-0	NP-192	2mg/ml	DMSO	2865	Natural Products Library
**2**	2-H01	α-Apo-Oxytetracycline	(4R,5S)-4-{4,5-dihydroxy-9-methyl-3-oxo-1H,3H-naphtho[2,3-c]furan-1-yl}-3-(dimethylamino)-2,5-dihydroxy-6-oxocyclohex-1-ene-1-carboxamide	442,4	18695-01-7	NP-219	2mg/ml	DMSO	2865	Natural Products Library
**2**	2-H02	Pseudopelletierine·HCl	9-methyl-9-azabicyclo[3.3.1]nonan-3-one hydrochloride	189,7	6164-62-1	NP-244	2mg/ml	DMSO	2865	Natural Products Library
**2**	2-H03	Salsoninol·HBr	(1S)-1-methyl-1,2,3,4-tetrahydroisoquinoline-6,7-diol hydrobromide	260,1	38221-21-5	NP-268	2mg/ml	DMSO	2865	Natural Products Library
**2**	2-H04	β-Sitosterol	(1S,2R,5S,10S,11S,14R,15R)-14-[(2R,5R)-5-ethyl-6-methylheptan-2-yl]-2,15-dimethyltetracyclo[8.7.0.$0^{2,7}$.$0^{11,15}$]heptadec-7-en-5-ol	414,7	83-46-5	NP-294	2mg/ml	DMSO	2865	Natural Products Library
**2**	2-H05	Sterigmatocystin	(7R)-15-hydroxy-11-methoxy-6,8,20-trioxapentacyclo[10.8.0.$0^{2,9}$.$0^{3,7}$.$0^{14,19}$]icosa-1(12),2(9),4,10,14(19),15,17-heptaen-13-one	324,3	10048-13-2	NP-304	2mg/ml	DMSO	2865	Natural Products Library
**2**	2-H06	4,5',8-Trimethylpsoralen	2,5,9-trimethyl-7H-furo[3,2-g]chromen-7-one	228,2	3902-71-4	NP-323	2mg/ml	DMSO	2865	Natural Products Library
**2**	2-H07	Cinobufagin	(1R,2S,4R,5R,6S,7R,10S,11S,14S,16R)-14-hydroxy-7,11-dimethyl-6-(2-oxo-2H-pyran-5-yl)-3-oxapentacyclo[8.8.0.$0^{2,4}$.$0^{2,7}$.$0^{11,16}$]octadecan-5-yl acetate	442,5	470-37-1	NP-055	2mg/ml	DMSO	2865	Natural Products Library
**2**	2-H08	Emetine·2HCl	(1R)-1-{[(2S,3R,11bS)-3-ethyl-9,10-dimethoxy-1H,2H,3H,4H,6H,7H,11bH-pyrido[2,1-a]isoquinolin-2-yl]methyl}-6,7-dimethoxy-1,2,3,4-tetrahydroisoquinoline dihydrochloride	553,6	316-42-7	NP-093	2mg/ml	DMSO	2865	Natural Products Library
**2**	2-H09	Kaempferol	3,5,7-trihydroxy-2-(4-hydroxyphenyl)-4H-chromen-4-one	286,2	520-18-3	NP-156	2mg/ml	DMSO	2865	Natural Products Library
**2**	2-H10	Kanamycin sulfate	(3S,4R,6R)-2-(aminomethyl)-6-{[(1R,4R,6R)-4,6-diamino-3-{[(2S,4R,5S)-4-amino-3,5-dihydroxy-6-(hydroxymethyl)oxan-2-yl]oxy}-2-hydroxycyclohexyl]oxy}oxane-3,4,5-triol; sulfuric acid	582,6	25389-94-0	NP-160	2mg/ml	DMSO	2865	Natural Products Library
**2**	2-H11	Celastrol	(2R,4aS,6aS,12bR,14aS,14bR)-10-hydroxy-2,4a,6a,9,12b,14a-hexamethyl-11-oxo-1,2,3,4,4a,5,6,6a,11,12b,13,14,14a,14b-tetradecahydropicene-2-carboxylic acid	450,6	34157-83-0	NP-568	2mg/ml	DMSO	2865	Natural Products Library
**2**	2-H12	(+)-Taxifolin	(2R,3R)-2-(3,4-dihydroxyphenyl)-3,5,7-trihydroxy-3,4-dihydro-2H-1-benzopyran-4-one	304,3	480-18-2	NP-313	2mg/ml	DMSO	2865	Natural Products Library

Table S2. ENZO – Natural Product library

Plate number	Plate Location	Name	IUPAC	MW	CAS	Catalog number	Conc	Solvent	Plate part number	Plate description
**3**	3-A01	Theobromine	3,7-dimethyl-2,3,6,7-tetrahydro-1H-purine-2,6-dione	180,2	83-67-0	NP-317	2mg/ml	DMSO	2865	Natural Products Library
**3**	3-A02	Baccatin III	(1S,2S,3R,4S,7R,9S,10S,12R,15S)-4,12-bis(acetyloxy)-1,9,15-trihydroxy-10,14,17,17-tetramethyl-11-oxo-6-oxatetracyclo[11.3.1.0^{3,10}.0^{4,7}]heptadec-13-en-2-yl benzoate	586,6	27548-93-2	NP-402	2mg/ml	DMSO	2865	Natural Products Library
**3**	3-A03	Carminic acid	3,5,6,8-tetrahydroxy-1-methyl-9,10-dioxo-7-[(2R,3R,4R,5S,6R)-3,4,5-trihydroxy-6-(hydroxymethyl)oxan-2-yl]-9,10-dihydroanthracene-2-carboxylic acid	492,4	1260-17-9	NP-043	2mg/ml	DMSO	2865	Natural Products Library
**3**	3-A04	(-)-Cotinine	(5S)-1-methyl-5-(pyridin-3-yl)pyrrolidin-2-one	176,2	486-56-6	NP-064	2mg/ml	DMSO	2865	Natural Products Library
**3**	3-A05	Austricin	(3S,3aR,4S,9bR)-4-hydroxy-3,6,9-trimethyl-2H,3H,3aH,4H,5H,7H,9aH,9bH-azuleno[4,5-b]furan-2,7-dione	262,3	10180-88-8	NP-024	2mg/ml	DMSO	2865	Natural Products Library
**3**	3-A06	Condelphine	(4S,5R,8S,9S,10R,16S)-11-ethyl-8,16-dihydroxy-6-methoxy-13-(methoxymethyl)-11-azahexacyclo[7.7.2.1^{2,5}.0^{1,10}.0^{3,8}.0^{13,17}]nonadecan-4-yl acetate	449,6	7633-69-4	NP-057	2mg/ml	DMSO	2865	Natural Products Library
**3**	3-A07	Delcorine	(1S,4S,5R,6S,8R,16S,19S,21S)-14-ethyl-4,6,19-trimethoxy-16-(methoxymethyl)-9,11-dioxa-14-azaheptacyclo[10.7.2.1^{2,5}.0^{1,13}.0^{3,8}.0^{8,12}.0^{16,20}]docosan-21-ol	479,6	52358-55-1	AC-918	2mg/ml	DMSO	2865	Natural Products Library
**3**	3-A08	Deltaline	(1R,2S,4S,5R,8R,12S,16R,19S,21S)-14-ethyl-2-hydroxy-4,6,19-trimethoxy-16-methyl-9,11-dioxa-14-azaheptacyclo[10.7.2.1^{2,5}.0^{1,13}.0^{3,8}.0^{8,12}.0^{16,20}]docosan-21-yl acetate	507,6	6836-11-9	NP-071	2mg/ml	DMSO	2865	Natural Products Library
**3**	3-A09	Diacetylkorseveriline	(1S,2R,6R,9S,10R,11S,14R,15R,17R,18S,20R,23R,24S)-20-(acetyloxy)-14-hydroxy-6,10,23-trimethyl-4-azahexacyclo[12.11.0.0^{2,11}.0^{4,9}.0^{15,24}.0^{18,23}]pentacosan-17-yl acetate	515,7	21851-07-0	NP-074	2mg/ml	DMSO	2865	Natural Products Library
**3**	3-A10	Dubinidine	2-{4-methoxy-2H,3H-furo[2,3-b]quinolin-2-yl}propane-1,2-diol	275,3	22964-77-8	NP-087	2mg/ml	DMSO	2865	Natural Products Library
**3**	3-A11	Eudesmine	(1S,4S)-1,4-bis(3,4-dimethoxyphenyl)-hexahydrofuro[3,4-c]furan	386,4	526-06-7	NP-100	2mg/ml	DMSO	2865	Natural Products Library
**3**	3-A12	Feroline	(7E,9R,10R)-9-hydroxy-3,7-dimethyl-10-(propan-2-yl)cyclodeca-3,7-dien-1-yl 4-hydroxybenzoate	358,5	39380-12-6	NP-102	2mg/ml	DMSO	2865	Natural Products Library
**3**	3-B01	Fillalbin	8-methyl-8-azabicyclo[3.2.1]octan-3-yl 4-hydroxy-3-methoxybenzoate	291,3	4540-25-4	NP-103	2mg/ml	DMSO	2865	Natural Products Library
**3**	3-B02	Graveoline	2-(2H-1,3-benzodioxol-5-yl)-1-methyl-1,4-dihydroquinolin-4-one	279,3	485-61-0	NP-118	2mg/ml	DMSO	2865	Natural Products Library
**3**	3-B03	Heliotrine	[(1S,7aR)-1-hydroxy-2,3,5,7a-tetrahydro-1H-pyrrolizin-7-yl]methyl 2-hydroxy-3-methoxy-2-(propan-2-yl)butanoate	313,4	303-33-3	NP-125	2mg/ml	DMSO	2865	Natural Products Library

Table S2. ENZO – Natural Product library

Plate number	Plate Location	Name	IUPAC	MW	CAS	Catalog number	Conc	Solvent	Plate part number	Plate description
**3**	3-B04	Hernandezine	(1S,14S)-9,19,20,21,25-pentamethoxy-15,30-dimethyl-7,23-dioxa-15,30-diazaheptacyclo[22.6.2.2^{3,6}.1^{8,12}.1^{14,18}.0^{27,31}.0^{22,33}]hexatriaconta-3,5,8,10,12(34),18(33),19,21,24(32),25,27(31),35-dodecaene	652,8	6681-13-6	NP-126	2mg/ml	DMSO	2865	Natural Products Library
**3**	3-B05	Heteratisine	(1S,6S,9S,10R,11R,14R)-12-ethyl-9,19-dihydroxy-17-methoxy-14-methyl-5-oxa-12-azahexacyclo[8.7.2.1^{2,6}.0^{1,11}.0^{3,9}.0^{14,18}]icosan-4-one	391,5	3328-84-5	NP-129	2mg/ml	DMSO	2865	Natural Products Library
**3**	3-B06	Imperialine	(1R,2S,6S,9S,10S,11S,14S,15S,18S,20S,23R,24S)-10,20-dihydroxy-6,10,23-trimethyl-4-azahexacyclo[12.11.0.0^{2,11}.0^{4,9}.0^{15,24}.0^{18,23}]pentacosan-17-one	429,6	18059-10-4	AC-916	2mg/ml	DMSO	2865	Natural Products Library
**3**	3-B07	Karakoline	(1S,4S,5S,6S,8S,9S,10R,13R,16S)-11-ethyl-6-methoxy-13-methyl-11-azahexacyclo[7.7.2.1^{2,5}.0^{1,10}.0^{3,8}.0^{13,17}]nonadecane-4,8,16-triol	377,5	39089-30-0	AC-920	2mg/ml	DMSO	2865	Natural Products Library
**3**	3-B08	Lapidin	(3R,3aS,4S,8aS)-3-hydroxy-6,8a-dimethyl-8-oxo-3-(propan-2-yl)-1,2,3,3a,4,5,8,8a-octahydroazulen-4-yl (2Z)-2-methylbut-2-enoate	334,4	79863-24-4	NP-164	2mg/ml	DMSO	2865	Natural Products Library
**3**	3-B09	Lapiferine	(1aS,2S,5R,5aS,6S,7aR)-2-(acetyloxy)-5-hydroxy-2a,7a-dimethyl-5-(propan-2-yl)-decahydroazuleno[5,6-b]oxiren-6-yl (2Z)-2-methylbut-2-enoate	394,5	86992-41-8	NP-165	2mg/ml	DMSO	2865	Natural Products Library
**3**	3-B10	Nitrarine·2HCl	4,14,20-triazahexacyclo[13.6.2.0^{2,14}.0^{3,11}.0^{5,10}.0^{16,21}]tricosa-3(11),5(10),6,8-tetraene dihydrochloride	380,4	20069-05-0	NP-206	2mg/ml	DMSO	2865	Natural Products Library
**3**	3-B11	Norfluorocurarine	(1R,11S,12E,17S)-12-ethylidene-8,14-diazapentacyclo[9.5.2.0^{1,9}.0^{2,7}.0^{14,17}]octadeca-2(7),3,5,9-tetraene-10-carbaldehyde	292,4	6880-54-2	NP-209	2mg/ml	DMSO	2865	Natural Products Library
**3**	3-B12	Peganole	1H,2H,3H,9H-pyrrolo[2,1-b]quinazolin-9-ol	188,2	36101-54-9	NP-224	2mg/ml	DMSO	2865	Natural Products Library
**3**	3-C01	Pinocembrin	(2S)-5,7-dihydroxy-2-phenyl-3,4-dihydro-2H-1-benzopyran-4-one	256,3	480-39-7	NP-233	2mg/ml	DMSO	2865	Natural Products Library
**3**	3-C02	Protopine	15-methyl-7,9,19,21-tetraoxa-15-azapentacyclo[15.7.0.0^{4,12}.0^{6,10}.0^{18,22}]tetracosa-1(17),4(12),5,10,18(22),23-hexaen-3-one	353,4	130-86-9	NP-242	2mg/ml	DMSO	2865	Natural Products Library
**3**	3-C03	Remerine·HCl	(12R)-11-methyl-3,5-dioxa-11-azapentacyclo[10.7.1.0^{2,6}.0^{8,20}.0^{14,19}]icosa-1,6,8(20),14,16,18-hexaene hydrochloride	315,8	17669-16-8	NP-252	2mg/ml	DMSO	2865	Natural Products Library
**3**	3-C04	Sevedindione	(1S,2R,6R,9S,10R,11S,14R,15R,18S,23R,24S)-14-hydroxy-6,10,23-trimethyl-4-azahexacyclo[12.11.0.0^{2,11}.0^{4,9}.0^{15,24}.0^{18,23}]pentacosane-17,20-dione	427,6		NP-289	2mg/ml	DMSO	2865	Natural Products Library
**3**	3-C05	Skimmianine	4,7,8-trimethoxyfuro[2,3-b]quinoline	259,3	83-95-4	NP-295	2mg/ml	DMSO	2865	Natural Products Library

Table S2. ENZO – Natural Product library

Plate number	Plate Location	Name	IUPAC	MW	CAS	Catalog number	Conc	Solvent	Plate part number	Plate description
**3**	3-C06	Songorine	(1R,7R,8R,9R,10R,13R,16S)-11-ethyl-7,16-dihydroxy-13-methyl-6-methylidene-11-azahexacyclo[7.7.2.1^{5,8}.0^{1,10}.0^{2,8}.0^{13,17}]nonadecan-4-one	357,5	509-24-0	NP-300	2mg/ml	DMSO	2865	Natural Products Library
**3**	3-C07	Trichodesmine	(1R,4R,5R,6R,16R)-5,6-dihydroxy-5,6-dimethyl-4-(propan-2-yl)-2,8-dioxa-13-azatricyclo[8.5.1.0^{13,16}]hexadec-10-ene-3,7-dione	353,4	548-90-3	NP-321	2mg/ml	DMSO	2865	Natural Products Library
**3**	3-C08	Tschimganidin	(1S,7E,9R,10R)-9-hydroxy-3,7-dimethyl-10-(propan-2-yl)cyclodeca-3,7-dien-1-yl 4-hydroxy-3-methoxybenzoate	388,5	39380-16-0	NP-325	2mg/ml	DMSO	2865	Natural Products Library
**3**	3-C09	Tschimganine	(1R,2R)-1,7,7-trimethylbicyclo[2.2.1]heptan-2-yl 4-hydroxy-3-methoxybenzoate	304,4	38970-49-9	NP-326	2mg/ml	DMSO	2865	Natural Products Library
**3**	3-C10	Ungerine nitrate	(3R,9R,10S)-9-methoxy-4-methyl-11,16,18-trioxa-4-azapentacyclo[11.7.0.0^{2,10}.0^{3,7}.0^{15,19}]icosa-1(13),7,14,19-tetraen-12-one; nitric acid	392,4		NP-329	2mg/ml	DMSO	2865	Natural Products Library
**3**	3-C11	Genistin	5-hydroxy-3-(4-hydroxyphenyl)-7-{[(2S,3R,4S,5S,6R)-3,4,5-trihydroxy-6-(hydroxymethyl)oxan-2-yl]oxy}-4H-chromen-4-one	432,4	529-59-9	NP-111	2mg/ml	DMSO	2865	Natural Products Library
**3**	3-C12	Laudanosine methiodide	1-[(3,4-dimethoxyphenyl)methyl]-6,7-dimethoxy-2,2-dimethyl-1,2,3,4-tetrahydroisoquinolin-2-ium iodide	499,4	24770-59-0	NP-566	2mg/ml	DMSO	2865	Natural Products Library
**3**	3-D01	Apigenin-7-glucoside	5-hydroxy-2-(4-hydroxyphenyl)-7-{[(2S,3R,4S,5R,6R)-3,4,5-trihydroxy-6-(hydroxymethyl)oxan-2-yl]oxy}-4H-chromen-4-one	432,4	578-74-5	NP-013	2mg/ml	DMSO	2865	Natural Products Library
**3**	3-D02	Bavachinin A	(2S)-2-(4-hydroxyphenyl)-7-methoxy-6-(3-methylbut-2-en-1-yl)-3,4-dihydro-2H-1-benzopyran-4-one	338,4	19879-30-2	NP-025	2mg/ml	DMSO	2865	Natural Products Library
**3**	3-D03	Decylubiquinone	2-decyl-5,6-dimethoxy-3-methylcyclohexa-2,5-diene-1,4-dione	322,4	55486-00-5	CM-115	2mg/ml	DMSO	2865	Natural Products Library
**3**	3-D04	Convolvamine	8-methyl-8-azabicyclo[3.2.1]octan-3-yl 3,4-dimethoxybenzoate	305,4	500-56-1	NP-058	2mg/ml	DMSO	2865	Natural Products Library
**3**	3-D05	Daidzin	3-(4-hydroxyphenyl)-7-{[(2S,3R,4S,5S,6R)-3,4,5-trihydroxy-6-(hydroxymethyl)oxan-2-yl]oxy}-4H-chromen-4-one	416,4	552-66-9	NP-067	2mg/ml	DMSO	2865	Natural Products Library
**3**	3-D06	Datiscetin	3,5,7-trihydroxy-2-(2-hydroxyphenyl)-4H-chromen-4-one	286,2	480-15-9	NP-068	2mg/ml	DMSO	2865	Natural Products Library
**3**	3-D07	N-Formyl-Deacetylcolchicine	N-[(10S)-3,4,5,14-tetramethoxy-13-oxotricyclo[9.5.0.0^{2,7}]hexadeca-1(16),2(7),3,5,11,14-hexaen-10-yl]formamide	385,4	7411-12-3	NP-069	2mg/ml	DMSO	2865	Natural Products Library
**3**	3-D08	Oridonin	(1S,2S,5S,8R,9S,10S,11R,15S,18R)-9,10,15,18-tetrahydroxy-12,12-dimethyl-6-methylidene-17-oxapentacyclo[7.6.2.1^{5,8}.0^{1,11}.0^{2,8}]octadecan-7-one	364,4	16964-56-0	NP-567	2mg/ml	DMSO	2865	Natural Products Library
**3**	3-D09	Eriocitrin	(2S)-2-(3,4-dihydroxyphenyl)-5-hydroxy-7-{[(2S,3R,4S,5S,6R)-3,4,5-trihydroxy-6-({[(2R,3R,4R,5R,6S)-3,4,5-trihydroxy-6-methyloxan-2-yl]oxy}methyl)oxan-2-yl]oxy}-3,4-dihydro-2H-1-benzopyran-4-one	596,5	13463-28-0	NP-096	2mg/ml	DMSO	2865	Natural Products Library
**3**	3-D10	Eriodictyol	(3S)-3-(3,4-dihydroxyphenyl)-6,8-dihydroxy-3,4-dihydro-2H-1-benzopyran-4-one	288,3	4049-38-1	NP-097	2mg/ml	DMSO	2865	Natural Products Library

Table S2. ENZO – Natural Product library

Plate number	Plate Location	Name	IUPAC	MW	CAS	Catalog number	Conc	Solvent	Plate part number	Plate description
**3**	3-D11	Eriodictyol-7-O-glucoside	(2S)-2-(3,4-dihydroxyphenyl)-5-hydroxy-7-{[(2R,3S,4R,5R,6S)-3,4,5-trihydroxy-6-(hydroxymethyl)oxan-2-yl]oxy}-3,4-dihydro-2H-1-benzopyran-4-one	450,4	38965-51-4	NP-098	2mg/ml	DMSO	2865	Natural Products Library
**3**	3-D12	Homobutein	(2E)-1-(2,4-dihydroxyphenyl)-3-(4-hydroxy-3-methoxyphenyl)prop-2-en-1-one	286,3	34000-39-0	NP-132	2mg/ml	DMSO	2865	Natural Products Library
**3**	3-E01	Homoeriodictyol	(2S)-5,7-dihydroxy-2-(4-hydroxy-3-methoxyphenyl)-3,4-dihydro-2H-1-benzopyran-4-one	302,3	446-71-9	NP-133	2mg/ml	DMSO	2865	Natural Products Library
**3**	3-E02	Homoorientin	2-(3,4-dihydroxyphenyl)-5,7-dihydroxy-6-[(2S,3R,4R,5S,6R)-3,4,5-trihydroxy-6-(hydroxymethyl)oxan-2-yl]-4H-chromen-4-one	448,4	4261-42-1	NP-134	2mg/ml	DMSO	2865	Natural Products Library
**3**	3-E03	7-Hydroxyflavone	7-hydroxy-2-phenyl-4H-chromen-4-one	238,2	6665-86-7	NP-138	2mg/ml	DMSO	2865	Natural Products Library
**3**	3-E04	Isorhamnetine-3-glucoside	5,7-dihydroxy-2-(4-hydroxy-3-methoxyphenyl)-3-{[(2S,3R,4S,5S,6R)-3,4,5-trihydroxy-6-(hydroxymethyl)oxan-2-yl]oxy}-4H-chromen-4-one	478,4	5041-82-7	NP-149	2mg/ml	DMSO	2865	Natural Products Library
**3**	3-E05	Isorhoifolin	5-hydroxy-2-(4-hydroxyphenyl)-7-{[(2S,3S,4R,5S)-3,4,5-trihydroxy-6-{[(2S,3R,4R,5R,6S)-3,4,5-trihydroxy-6-methyloxan-2-yl]oxy}oxan-2-yl]methoxy}-4H-chromen-4-one	578,5	552-57-8	NP-151	2mg/ml	DMSO	2865	Natural Products Library
**3**	3-E06	Isosakuranetin	(2S)-5,7-dihydroxy-2-(4-methoxyphenyl)-3,4-dihydro-2H-1-benzopyran-4-one	286,3	480-43-3	NP-152	2mg/ml	DMSO	2865	Natural Products Library
**3**	3-E07	Isovitexin	5,7-dihydroxy-2-(4-hydroxyphenyl)-6-[(2S,3R,4R,5S,6R)-3,4,5-trihydroxy-6-(hydroxymethyl)oxan-2-yl]-4H-chromen-4-one	432,4	29702-25-8	NP-154	2mg/ml	DMSO	2865	Natural Products Library
**3**	3-E08	Dihydromethystici n	(6S)-6-[2-(2H-1,3-benzodioxol-5-yl)ethyl]-4-methoxy-5,6-dihydro-2H-pyran-2-one	276,3	19902-91-1	NP-537	2mg/ml	DMSO	2865	Natural Products Library
**3**	3-E09	(β,β-Dimethylacryl) Shikonin	(1R)-1-(5,8-dihydroxy-1,4-dioxo-1,4-dihydronaphthalen-2-yl)-4-methylpent-3-en-1-yl 3-methylbut-2-enoate	370,4	24502-79-2	NP-572	2mg/ml	DMSO	2865	Natural Products Library
**3**	3-E10	Kaempferol-7-neohesperidoside	7-{[(3R,4S,5S,6R)-4,5-dihydroxy-6-(hydroxymethyl)-3-{[(2S,3R,4R,5R,6S)-3,4,5-trihydroxy-6-methyloxan-2-yl]oxy}oxan-2-yl]oxy}-3,5-dihydroxy-2-(4-hydroxyphenyl)-4H-chromen-4-one	594,5	17353-03-6	NP-159	2mg/ml	DMSO	2865	Natural Products Library
**3**	3-E11	Luteolin-3',7-diglucoside	2-{3,4-dihydroxy-5-[(2S,3R,4R,5S,6R)-3,4,5-trihydroxy-6-(hydroxymethyl)oxan-2-yl]phenyl}-5-hydroxy-7-{[(2S,3R,4S,5S,6R)-3,4,5-trihydroxy-6-(hydroxymethyl)oxan-2-yl]oxy}-4H-chromen-4-one	610,5	52187-80-1	NP-177	2mg/ml	DMSO	2865	Natural Products Library
**3**	3-E12	Flavokawain B	(2E)-1-(2-hydroxy-4,6-dimethoxyphenyl)-3-phenylprop-2-en-1-one	284,3	1775-97-9	NP-539	2mg/ml	DMSO	2865	Natural Products Library
**3**	3-F01	Marein	(2E)-1-(2,3-dihydroxy-4-{[(2S,3R,4S,5S,6R)-3,4,5-trihydroxy-6-(hydroxymethyl)oxan-2-yl]oxy}phenyl)-3-(3,4-dihydroxyphenyl)prop-2-en-1-one	450,4	535-96-6	NP-182	2mg/ml	DMSO	2865	Natural Products Library

Table S2. ENZO – Natural Product library

Plate number	Plate Location	Name	IUPAC	MW	CAS	Catalog number	Conc	Solvent	Plate part number	Plate description
**3**	3-F02	Maritimein	(2E)-2-[(3,4-dihydroxyphenyl)methylidene]-7-hydroxy-6-{[(2S,3R,4S,5S,6R)-3,4,5-trihydroxy-6-(hydroxymethyl)oxan-2-yl]oxy}-2,3-dihydro-1-benzofuran-3-one	448,4	490-54-0	NP-183	2mg/ml	DMSO	2865	Natural Products Library
**3**	3-F03	Picropodophyllin	(10R,11S,15R,16R)-16-hydroxy-10-(3,4,5-trimethoxyphenyl)-4,6,13-trioxatetracyclo[7.7.0.0^{3,7}.0^{11,15}]hexadeca-1,3(7),8-trien-12-one	414,4	447-47-4	EI-372	2mg/ml	DMSO	2865	Natural Products Library
**3**	3-F04	Myricitrin	5,7-dihydroxy-3-{[(2S,3R,4R,5R,6S)-3,4,5-trihydroxy-6-methyloxan-2-yl]oxy}-2-(3,4,5-trihydroxyphenyl)-4H-chromen-4-one	464,4	17912-87-7	NP-196	2mg/ml	DMSO	2865	Natural Products Library
**3**	3-F05	Narigenin-7-O-glucoside	(2S)-5-hydroxy-2-(4-hydroxyphenyl)-7-{[(2S,3R,4S,5S,6R)-3,4,5-trihydroxy-6-(hydroxymethyl)oxan-2-yl]oxy}-3,4-dihydro-2H-1-benzopyran-4-one	434,4	529-55-5	NP-201	2mg/ml	DMSO	2865	Natural Products Library
**3**	3-F06	Narirutin	5-hydroxy-2-(4-hydroxyphenyl)-7-[(3,4,5-trihydroxy-6-{[(3,4,5-trihydroxy-6-methyloxan-2-yl)oxy]methyl}oxan-2-yl)oxy]-3,4-dihydro-2H-1-benzopyran-4-one	580,5	14259-46-2	NP-203	2mg/ml	DMSO	2865	Natural Products Library
**3**	3-F07	Picrotonin	(1R,3R,5S,8S,9R,12S,13R,14S)-1-hydroxy-14-(2-hydroxypropan-2-yl)-13-methyl-4,7,10-trioxapentacyclo[6.4.1.1^{9,12}.0^{3,5}.0^{5,13}]tetradecane-6,11-dione	310,3	21416-53-5	NP-400	2mg/ml	DMSO	2865	Natural Products Library
**3**	3-F08	Plumbagin	5-hydroxy-2-methyl-1,4-dihydronaphthalene-1,4-dione	188,2	481-42-5	NP-236	2mg/ml	DMSO	2865	Natural Products Library
**3**	3-F09	Ketopinic acid	(1S,4R)-7,7-dimethyl-2-oxobicyclo[2.2.1]heptane-1-carboxylic acid	182,2	40724-67-2	NP-543	2mg/ml	DMSO	2865	Natural Products Library
**3**	3-F10	Scopolamine N-butylbromide	(1R,2R,4S,5S,7R)-9-butyl-7-{[(2S)-3-hydroxy-2-phenylpropanoyl]oxy}-9-methyl-3-oxa-9-azatricyclo[3.3.1.0^{2,4}]nonan-9-ium bromide	440,4	149-64-4	NP-527	2mg/ml	DMSO	2865	Natural Products Library
**3**	3-F11	Rhamnetine	2-(3,4-dihydroxyphenyl)-3,5-dihydroxy-7-methoxy-4H-chromen-4-one	316,3	90-19-7	NP-257	2mg/ml	DMSO	2865	Natural Products Library
**3**	3-F12	Rhoifolin	7-{[(2S,3R,5S,6R)-4,5-dihydroxy-6-(hydroxymethyl)-3-{[(2S,3R,4R,5R,6S)-3,4,5-trihydroxy-6-methyloxan-2-yl]oxy}oxan-2-yl]oxy}-5-hydroxy-2-(4-hydroxyphenyl)-4H-chromen-4-one	578,5	17306-46-6	NP-259	2mg/ml	DMSO	2865	Natural Products Library
**3**	3-G01	Sanguinarine	24-methyl-5,7,18,20-tetraoxa-24-azahexacyclo[11.11.0.0^{2,10}.0^{4,8}.0^{14,22}.0^{17,21}]tetracosa-1(13),2(10),3,8,11,14(22),15,17(21),23-nonaen-24-ium	332,3	5578-73-4	NP-269	2mg/ml	DMSO	2865	Natural Products Library
**3**	3-G02	Saponarin	5-hydroxy-2-(4-hydroxyphenyl)-6-[(2S,3R,4R,5S,6R)-3,4,5-trihydroxy-6-(hydroxymethyl)oxan-2-yl]-7-{[(2S,3R,4S,5S,6R)-3,4,5-trihydroxy-6-(hydroxymethyl)oxan-2-yl]oxy}-4H-chromen-4-one	594,5	20310-89-8	NP-271	2mg/ml	DMSO	2865	Natural Products Library
**3**	3-G03	Manool	(3R)-5-[(1S,4aS,8aS)-5,5,8a-trimethyl-2-methylidene-decahydronaphthalen-1-yl]-3-methylpent-1-en-3-ol	290,5	596-85-0	NP-545	2mg/ml	DMSO	2865	Natural Products Library

Table S2. ENZO – Natural Product library

Plate number	Plate Location	Name	IUPAC	MW	CAS	Catalog number	Conc	Solvent	Plate part number	Plate description
**3**	3-G04	Citreoviridin	6-[(1E,3E,5E,7E)-8-(3,4-dihydroxy-2,4,5-trimethyloxolan-2-yl)-7-methylocta-1,3,5,7-tetraen-1-yl]-4-methoxy-5-methyl-2H-pyran-2-one	402,5	25425-12-1	NP-541	2mg/ml	DMSO	2865	Natural Products Library
**3**	3-G05	Sinensetine	2-(3,4-dimethoxyphenyl)-5,6,7-trimethoxy-4H-chromen-4-one	372,4	2306-27-6	NP-292	2mg/ml	DMSO	2865	Natural Products Library
**3**	3-G06	Sulfuretine	(2E)-2-[(3,4-dihydroxyphenyl)methylidene]-6-hydroxy-2,3-dihydro-1-benzofuran-3-one	270,2	120-05-8	NP-308	2mg/ml	DMSO	2865	Natural Products Library
**3**	3-G07	Atropine-N-oxide·HCl	(1R,3S,5S)-3-[(3-hydroxy-2-phenylpropanoyl)oxy]-8-methyl-8-azabicyclo[3.2.1]octan-8-ium-8-olate hydrochloride	341,8	4574-60-1	NP-544	2mg/ml	DMSO	2865	Natural Products Library
**3**	3-G08	Tamarixetine	3,5,7-trihydroxy-2-(4-hydroxy-3-methoxyphenyl)-4H-chromen-4-one	316,3	603-61-2	NP-311	2mg/ml	DMSO	2865	Natural Products Library
**3**	3-G09	Teterahydroalstonine	methyl (1S,15S,16S,20S)-16-methyl-17-oxa-3,13-diazapentacyclo[11.8.0.0^{2,10}.0^{4,9}.0^{15,20}]henicosa-2(10),4,6,8,18-pentaene-19-carboxylate	352,4	6474-90-4	NP-314	2mg/ml	DMSO	2865	Natural Products Library
**3**	3-G10	Diacetoxyscirpenol	(1'S,2R,2'R,7'R,9'R,10'R,11'S)-11'-(acetyloxy)-10'-hydroxy-1',5'-dimethyl-8'-oxaspiro[oxirane-2,12'-tricyclo[7.2.1.0^{2,7}]dodecan]-5'-en-2'-ylmethyl acetate	366,4	2270-40-8	NP-563	2mg/ml	DMSO	2865	Natural Products Library
**3**	3-G11	Vitexin-2"-O-rhamnoside	5,7-dihydroxy-8-[(2S,3R,4R,5S,6R)-3,4,5-trihydroxy-6-(hydroxymethyl)oxan-2-yl]-2-(2-{[(3R,4R,5R,6S)-3,4,5-trihydroxy-6-methyloxan-2-yl]oxy}phenyl)-4H-chromen-4-one	578,5	64820-99-1	NP-336	2mg/ml	DMSO	2865	Natural Products Library
**3**	3-G12	Laudanosoline·HBr·$3H_2O$	1-[(3,4-dihydroxyphenyl)methyl]-2-methyl-1,2,3,4-tetrahydroisoquinoline-6,7-diol trihydrate hydrobromide	436,3	485-33-6	NP-169	2mg/ml	DMSO	2865	Natural Products Library
**3**	3-H01	(±)-6'-Bromolaudanosine	1-[(2-bromo-4,5-dimethoxyphenyl)methyl]-6,7-dimethoxy-2-methyl-1,2,3,4-tetrahydroisoquinoline	436,3	53392-66-8	NP-037	2mg/ml	DMSO	2865	Natural Products Library
**3**	3-H02	Andrographolide	(3E,4S)-3-{2-[(1R,4aS,5R,6R,8aS)-6-hydroxy-5-(hydroxymethyl)-5,8a-dimethyl-2-methylidene-decahydronaphthalen-1-yl]ethylidene}-4-hydroxyoxolan-2-one	350,4	5508-58-7	NP-008	2mg/ml	DMSO	2865	Natural Products Library
**3**	3-H03	Ajmaline	(1R,9R,10S,12R,13S,14R,16S,17S,18R)-13-ethyl-8-methyl-8,15-diazahexacyclo[14.2.1.0^{1,9}.0^{2,7}.0^{10,15}.0^{12,17}]nonadeca-2,4,6-triene-14,18-diol	326,4	4360-12-7	NP-004	2mg/ml	DMSO	2865	Natural Products Library
**3**	3-H04	(+)-Chelidonine	(1S,12S,13R)-24-methyl-5,7,18,20-tetraoxa-24-azahexacyclo[11.11.0.0^{2,10}.0^{4,8}.0^{14,22}.0^{17,21}]tetracosa-2(10),3,8,14(22),15,17(21)-hexaen-12-ol	353,4	476-32-4	NP-050	2mg/ml	DMSO	2865	Natural Products Library
**3**	3-H05	6,7-Dihydroxyflavone	6,7-dihydroxy-2-phenyl-4H-chromen-4-one	254,2	38183-04-9	NP-081	2mg/ml	DMSO	2865	Natural Products Library
**3**	3-H06	Fisetin	2-(3,4-dihydroxyphenyl)-3,7-dihydroxy-4H-chromen-4-one	286,2	528-48-3	NP-104	2mg/ml	DMSO	2865	Natural Products Library

Table S2. ENZO – Natural Product library

Plate number	Plate Location	Name	IUPAC	MW	CAS	Catalog number	Conc	Solvent	Plate part number	Plate description
**3**	3-H07	Harmalol·HCl·$2H_2O$	1-methyl-3H,4H,9H-pyrido[3,4-b]indol-7-ol dihydrate hydrochloride	272,7	6028-00-8	NP-121	2mg/ml	DMSO	2865	Natural Products Library
**3**	3-H08	Harmol·HCl·2H2O	1-methyl-9H-pyrido[3,4-b]indol-7-ol dihydrate hydrochloride	270,7	40580-83-4	NP-124	2mg/ml	DMSO	2865	Natural Products Library
**3**	3-H09	Isorhamnetine-3-rutinoside	5,7-dihydroxy-2-(4-hydroxy-3-methoxyphenyl)-3-{[(2S,3S,4R,5S)-3,4,5-trihydroxy-6-{[(2S,3R,4R,5R,6S)-3,4,5-trihydroxy-6-methyloxan-2-yl]oxy}oxan-2-yl]methoxy}-4H-chromen-4-one	624,5	604-80-8	NP-150	2mg/ml	DMSO	2865	Natural Products Library
**3**	3-H10	Isoscopoletine	6-hydroxy-7-methoxy-2H-chromen-2-one	192,2	776-86-3	NP-153	2mg/ml	DMSO	2865	Natural Products Library
**3**	3-H11	5-Methoxyflavone	5-methoxy-2-phenyl-4H-chromen-4-one	252,3	42079-78-7	NP-186	2mg/ml	DMSO	2865	Natural Products Library
**3**	3-H12	Pratol	7-hydroxy-2-(4-methoxyphenyl)-4H-chromen-4-one	268,3	487-24-1	NP-241	2mg/ml	DMSO	2865	Natural Products Library
**4**	4-A01	Syringetine-3-glucoside	5,7-dihydroxy-2-(4-hydroxy-3,5-dimethoxyphenyl)-3-{[(2S,3R,4S,5S,6R)-3,4,5-trihydroxy-6-(hydroxymethyl)oxan-2-yl]oxy}-4H-chromen-4-one	508,4	40039-49-4	NP-309	2mg/ml	DMSO	2865	Natural Products Library
**4**	4-A02	Conessine	(1R,5S,6S,9R,13R,16S)-N,N,6,7,13-pentamethyl-7-azapentacyclo[10.8.0.0^{2,9}.0^{5,9}.0^{13,18}]icos-18-en-16-amine	356,6	546-06-5	NP-495	2mg/ml	DMSO	2865	Natural Products Library
**4**	4-A03	Sarsasapogenin	(1'R,2R,2'S,4'S,5S,7'S,8'R,9'S,12'S,13'S,16'S,18'R)-5,7',9',13'-tetramethyl-5'-oxaspiro[oxane-2,6'-pentacyclo[10.8.0.0^{2,9}.0^{4,8}.0^{13,18}]icosane]-16'-ol	416,6	126-19-2	NP-272	2mg/ml	DMSO	2865	Natural Products Library
**4**	4-A04	Strophantidin	(2S,5S,7S,11S,15R)-5,7,11-trihydroxy-15-methyl-14-(5-oxo-2,5-dihydrofuran-3-yl)tetracyclo[8.7.0.0^{2,7}.0^{11,15}]heptadecane-2-carbaldehyde	404,5	66-28-4	NP-306	2mg/ml	DMSO	2865	Natural Products Library
**4**	4-A05	Hordenine sulfate	bis(4-[2-(dimethylamino)ethyl]phenol); sulfuric acid	428,5	622-64-0	NP-494	2mg/ml	DMSO	2865	Natural Products Library
**4**	4-A06	Ferutinin	(3R,3aR,4S,8aR)-3-hydroxy-6,8a-dimethyl-3-(propan-2-yl)-1,2,3,3a,4,5,8,8a-octahydroazulen-4-yl 4-hydroxybenzoate	358,5		NP-534	2mg/ml	DMSO	2865	Natural Products Library
**4**	4-A07	Piperine	(2E,4E)-5-(2H-1,3-benzodioxol-5-yl)-1-(piperidin-1-yl)penta-2,4-dien-1-one	285,3	94-62-2	NP-235	2mg/ml	DMSO	2865	Natural Products Library
**4**	4-A08	Quercitrin	2-(3,4-dihydroxyphenyl)-5,7-dihydroxy-3-{[(2S,3S,4R,5S,6S)-3,4,5-trihydroxy-6-methyloxan-2-yl]oxy}-4H-chromen-4-one	448,4	522-12-3	NP-246	2mg/ml	DMSO	2865	Natural Products Library
**4**	4-A09	(-)-Scopolamine N-oxide	(1S,2R,4S,5S,7R)-7-[(3-hydroxy-2-phenylpropanoyl)oxy]-9-methyl-3-oxa-9-azatricyclo[3.3.1.0^{2,4}]nonan-9-ium-9-olate	319,4	6106-81-6	NP-278	2mg/ml	DMSO	2865	Natural Products Library
**4**	4-A10	Shikimic acid	(3R,4S,5R)-3,4,5-trihydroxycyclohex-1-ene-1-carboxylic acid	174,2	138-59-0	NP-290	2mg/ml	DMSO	2865	Natural Products Library
**4**	4-A11	Stachydrine·HCl	(2S)-1,1-dimethylpyrrolidin-1-ium-2-carboxylate hydrochloride	179,6	4136-37-2	NP-303	2mg/ml	DMSO	2865	Natural Products Library
**4**	4-A12	Ochratoxin A	(2S)-2-{[(3R)-5-chloro-8-hydroxy-3-methyl-1-oxo-3,4-dihydro-1H-2-benzopyran-7-yl]formamido}-3-phenylpropanoic acid	403,8	303-47-9	NP-212	2mg/ml	DMSO	2865	Natural Products Library
**4**	4-B01	Patulin	4-hydroxy-2H,4H,6H-furo[3,2-c]pyran-2-one	154,1	149-29-1	NP-223	2mg/ml	DMSO	2865	Natural Products Library

Table S2. ENZO – Natural Product library

Plate number	Plate Location	Name	IUPAC	MW	CAS	Catalog number	Conc	Solvent	Plate part number	Plate description
**4**	4-B02	Zearalenone	(3S)-13,15-dihydroxy-3-methyl-3,4,5,6,7,8,9,10-octahydro-1H-2-benzoxacyclotetradecine-1,7-dione	318,4	17924-92-4	NP-341	2mg/ml	DMSO	2865	Natural Products Library
**4**	4-B03	Isorhamnetine	3,5,7-trihydroxy-2-(4-hydroxy-3-methoxyphenyl)-4H-chromen-4-one	316,3	418-19-3	NP-148	2mg/ml	DMSO	2865	Natural Products Library
**4**	4-B04	(±)-Abscisic acid	(2Z,4E)-5-(1-hydroxy-2,6,6-trimethyl-4-oxocyclohex-2-en-1-yl)-3-methylpenta-2,4-dienoic acid	264,3	14375-45-2	NP-001	2mg/ml	DMSO	2865	Natural Products Library
**4**	4-B05	Rifamycin·Na	sodium (7S,9E,11R,12R,13R,14R,15R,16R,17R,18R,19E,21Z)-2,15,17,29-tetrahydroxy-11-methoxy-13-(methoxycarbonyl)-3,7,12,14,16,18,22-heptamethyl-6,23-dioxo-8,30-dioxa-24-azatetracyclo[23.3.1.$1^{4,7}$.$0^{5,28}$]triaconta-1(28),2,4,9,19,21,25(29),26-octaen-27-olate	719,8	14897-39-3	NP-526	2mg/ml	DMSO	2865	Natural Products Library
**4**	4-B06	Aloe-emodine	1,8-dihydroxy-3-(hydroxymethyl)-9,10-dihydroanthracene-9,10-dione	270,2	481-72-1	NP-005	2mg/ml	DMSO	2865	Natural Products Library
**4**	4-B07	Antimycin A1	(2R,3S,6S,7R,8R)-3-(3-formamido-2-hydroxybenzamido)-8-hexyl-2,6-dimethyl-4,9-dioxo-1,5-dioxonan-7-yl 3-methylbutanoate	548,6	642-15-9	NP-009	2mg/ml	DMSO	2865	Natural Products Library
**4**	4-B08	(-)-Asarinin	5-[(3aR,6aR)-4-(2H-1,3-benzodioxol-5-yl)-hexahydrofuro[3,4-c]furan-1-yl]-2H-1,3-benzodioxole	354,4	133-05-1	NP-017	2mg/ml	DMSO	2865	Natural Products Library
**4**	4-B09	Aucubin	(3R,4S,5S,6R)-2-{[(1S,4aR,5S,7aS)-5-hydroxy-7-(hydroxymethyl)-1H,4aH,5H,7aH-cyclopenta[c]pyran-1-yl]oxy}-6-(hydroxymethyl)oxane-3,4,5-triol	346,3	479-98-1	NP-021	2mg/ml	DMSO	2865	Natural Products Library
**4**	4-B10	Deoxyshikonin	5,8-dihydroxy-2-(4-methylpent-3-en-1-yl)-1,4-dihydronaphthalene-1,4-dione	272,3	43043-74-9	NP-022	2mg/ml	DMSO	2865	Natural Products Library
**4**	4-B11	Boldine	(9S)-4,16-dimethoxy-10-methyl-10-azatetracyclo[7.7.1.$0^{2,7}$.$0^{13,17}$]heptadeca-1(16),2(7),3,5,13(17),14-hexaene-5,15-diol	327,4	476-70-0	NP-036	2mg/ml	DMSO	2865	Natural Products Library
**4**	4-B12	Caryophylline	(1R,4R,6R,10S)-4,12,12-trimethyl-9-methylidene-5-oxatricyclo[8.2.0.$0^{4,6}$]dodecane	220,4	1139-30-6	NP-045	2mg/ml	DMSO	2865	Natural Products Library
**4**	4-C01	(+)-Catechine	(2R,3S)-2-(3,4-dihydroxyphenyl)-3,4-dihydro-2H-1-benzopyran-3,5,7-triol	290,3	225937-10-0	NP-047	2mg/ml	DMSO	2865	Natural Products Library
**4**	4-C02	(-)-Cinchonidine	(R)-[(2S,5R)-5-ethenyl-1-azabicyclo[2.2.2]octan-2-yl](quinolin-4-yl)methanol	294,4	485-71-2	NP-053	2mg/ml	DMSO	2865	Natural Products Library
**4**	4-C03	(+)-Cinchonine	[(1R,2R,5R)-5-ethenyl-1-azabicyclo[2.2.2]octan-2-yl](quinolin-4-yl)methanol	294,4	118-10-5	NP-054	2mg/ml	DMSO	2865	Natural Products Library
**4**	4-C04	Trans-4-Cotininecarboxylic acid	(3S)-1-methyl-5-oxo-2-(pyridin-3-yl)pyrrolidine-3-carboxylic acid	220,2	33224-01-0	NP-065	2mg/ml	DMSO	2865	Natural Products Library
**4**	4-C05	Demissidine	(1S,2R,5S,7S,10S,11S,14S,15R,16S,17R,20S,23S)-10,14,16,20-tetramethyl-22-azahexacyclo[12.10.0.$0^{2,11}$.$0^{5,10}$.$0^{15,23}$.$0^{17,22}$]tetracosan-7-ol	399,7	474-08-8	NP-072	2mg/ml	DMSO	2865	Natural Products Library

Table S2. ENZO – Natural Product library

Plate number	Plate Location	Name	IUPAC	MW	CAS	Catalog number	Conc	Solvent	Plate part number	Plate description
**4**	4-C06	Dipterocarpol	(1R,2R,7R,10R,11R,14S,15R)-14-[(2S)-2-hydroxy-6-methylhept-5-en-2-yl]-2,6,6,10,11-pentamethyltetracyclo$[8.7.0.0^{2,7}.0^{11,15}]$heptadecan-5-one	442,7	471-69-2	NP-085	2mg/ml	DMSO	2865	Natural Products Library
**4**	4-C07	Dehydrocostus lactone	(3aS,6aR,9aR,9bS)-3,6,9-trimethylidene-dodecahydroazuleno[4,5-b]furan-2-one	230,3	477-43-0	NP-542	2mg/ml	DMSO	2865	Natural Products Library
**4**	4-C08	Friedelin	(4R,4aS,6aS,6bR,8aR,12aR,12bS,14aS,14bS)-4,4a,6b,8a,11,11,12b,14a-octamethyl-docosahydropicen-3-one	426,7	559-74-0	NP-107	2mg/ml	DMSO	2865	Natural Products Library
**4**	4-C09	Indole-3-butyric acid	4-(1H-indol-3-yl)butanoic acid	203,2	133-32-4	NP-532	2mg/ml	DMSO	2865	Natural Products Library
**4**	4-C10	(+)-Gibberellic acid	(1R,2R,5S,8S,9S,10R,11S,12S)-5,12-dihydroxy-11-methyl-6-methylidene-16-oxo-15-oxapentacyclo$[9.3.2.1^{5,8}.0^{1,10}.0^{2,8}]$heptadec-13-ene-9-carboxylic acid	346,4	77-06-5	NP-113	2mg/ml	DMSO	2865	Natural Products Library
**4**	4-C11	Gitoxigenin	4-[(1S,2S,5S,7R,10R,11S,13S,14R,15R)-5,11,13-trihydroxy-2,15-dimethyltetracyclo$[8.7.0.0^{2,7}.0^{11,15}]$heptadecan-14-yl]-2,5-dihydrofuran-2-one	390,5	545-26-6	NP-114	2mg/ml	DMSO	2865	Natural Products Library
**4**	4-C12	Harmane	1-methyl-9H-pyrido[3,4-b]indole	182,2	486-84-0	AC-1050	2mg/ml	DMSO	2865	Natural Products Library
**4**	4-D01	6-Hydroxytropinone	6-hydroxy-8-methyl-8-azabicyclo[3.2.1]octan-3-one	155,2	5932-53-6	NP-139	2mg/ml	DMSO	2865	Natural Products Library
**4**	4-D02	(+)-Isocorydine hydrochloride	(9S)-4,15,16-trimethoxy-10-methyl-10-azatetracyclo$[7.7.1.0^{2,7}.0^{13,17}]$heptadeca-1(17),2(7),3,5,13,15-hexaen-3-ol hydrochloride	377,9	13552-72-2	NP-144	2mg/ml	DMSO	2865	Natural Products Library
**4**	4-D03	(-)-Isoreserpine	methyl (1S,15S,17R,18R,19S,20S)-6,18-dimethoxy-17-(3,4,5-trimethoxybenzoyloxy)-3,13-diazapentacyclo$[11.8.0.0^{2,10}.0^{4,9}.0^{15,20}]$henicosa-2(10),4(9),5,7-tetraene-19-carboxylate	608,7	482-85-9	NP-147	2mg/ml	DMSO	2865	Natural Products Library
**4**	4-D04	Leucomisine	(3S,3aS,9aS,9bS)-3,6,9-trimethyl-2H,3H,3aH,4H,5H,7H,9aH,9bH-azuleno[4,5-b]furan-2,7-dione	246,3	17946-87-1	NP-170	2mg/ml	DMSO	2865	Natural Products Library
**4**	4-D05	Methylergonovine maleate	(2Z)-but-2-enedioic acid; (4R,7R)-N-[(2S)-1-hydroxybutan-2-yl]-6-methyl-6,11-diazatetracyclo$[7.6.1.0^{2,7}.0^{12,16}]$hexadeca-1(15),2,9,12(16),13-pentaene-4-carboxamide	455,5	57432-61-8	NP-189	2mg/ml	DMSO	2865	Natural Products Library
**4**	4-D06	Corydaline	(12bR,13S)-3,4,10,11-tetramethoxy-13-methyl-7,8,12b,13-tetrahydro-5H-6-azatetraphene	369,5	518-69-4	NP-061	2mg/ml	DMSO	2865	Natural Products Library
**4**	4-D07	(+)-Muscarine chloride	{[(2S,4R,5S)-4-hydroxy-5-methyloxolan-2-yl]methyl}trimethylazanium chloride	209,7	2303-35-7	AC-742	2mg/ml	DMSO	2865	Natural Products Library
**4**	4-D08	Nalidixic acid	1-ethyl-7-methyl-4-oxo-1,4-dihydro-1,8-naphthyridine-3-carboxylic acid	232,2	389-08-2	NP-197	2mg/ml	DMSO	2865	Natural Products Library

Table S2. ENZO – Natural Product library

Plate number	Plate Location	Name	IUPAC	MW	CAS	Catalog number	Conc	Solvent	Plate part number	Plate description
**4**	4-D09	Narasin	(2R)-2-[(3S,5S,6R)-6-[(2S,3S,4S,6R)-6-[(2S,5S,7R,9S,10S,12R,15R)-2-[(2R,5R,6S)-5-ethyl-5-hydroxy-6-methyloxan-2-yl]-15-hydroxy-2,10,12-trimethyl-1,6,8-trioxadispiro[4.1.5^{7}.3^{5}]pentadec-13-en-9-yl]-3-hydroxy-4-methyl-5-oxooctan-2-yl]-3,5-dimethyloxan-2-yl]butanoic acid	765,0	55134-13-9	NP-198	2mg/ml	DMSO	2865	Natural Products Library
**4**	4-D10	Noreleagnine	1H,2H,3H,4H,9H-pyrido[3,4-b]indole	172,2	16502-01-5	NP-208	2mg/ml	DMSO	2865	Natural Products Library
**4**	4-D11	Norharmane	9H-pyrido[3,4-b]indole	168,2	244-63-3	NP-210	2mg/ml	DMSO	2865	Natural Products Library
**4**	4-D12	Palmatine chloride	3,4,10,11-tetramethoxy-7,8-dihydro-6λ^{5}-azatetraphen-6-ylium chloride	387,9	171869-95-7	NP-220	2mg/ml	DMSO	2865	Natural Products Library
**4**	4-E01	Peruvoside	(1S,2R,5S,7R,10R,11S,14R,15R)-5-{[(2R,4S,5S)-3,5-dihydroxy-4-methoxy-6-methyloxan-2-yl]oxy}-11-hydroxy-15-methyl-14-(5-oxo-2,5-dihydrofuran-3-yl)tetracyclo[8.7.0.0^{2,7}.0^{11,15}]heptadecane-2-carbaldehyde	548,7	1182-87-2	NP-227	2mg/ml	DMSO	2865	Natural Products Library
**4**	4-E02	Physostigmine	(3aS,8aR)-1,3a,8-trimethyl-1H,2H,3H,3aH,8H,8aH-pyrrolo[2,3-b]indol-5-yl N-methylcarbamate	275,3	57-47-6	AC-241	2mg/ml	DMSO	2865	Natural Products Library
**4**	4-E03	6-Acetamido-6-deoxy-castanospermine	N-[(1S,6S,7R,8R,8aR)-1,7,8-trihydroxy-octahydroindolizin-6-yl]acetamide	230,3	134100-29-1	NP-573	2mg/ml	DMSO	2865	Natural Products Library
**4**	4-E04	Podocarpic acid	(1S,4aS,10aR)-6-hydroxy-1,4a-dimethyl-1,2,3,4,4a,9,10,10a-octahydrophenanthrene-1-carboxylic acid	274,4	5947-49-9	NP-237	2mg/ml	DMSO	2865	Natural Products Library
**4**	4-E05	Retrorsine	(1R,4Z,6R,7S,17R)-4-ethylidene-7-hydroxy-7-(hydroxymethyl)-6-methyl-2,9-dioxa-14-azatricyclo[9.5.1.0^{14,17}]heptadec-11-ene-3,8-dione	351,4	480-54-6	NP-255	2mg/ml	DMSO	2865	Natural Products Library
**4**	4-E06	Rhapontin	(2S,3R,4S,5S,6R)-2-{3-hydroxy-5-[(E)-2-(3-hydroxy-4-methoxyphenyl)ethenyl]phenoxy}-6-(hydroxymethyl)oxane-3,4,5-triol	420,4	155-58-8	NP-258	2mg/ml	DMSO	2865	Natural Products Library
**4**	4-E07	(3aR)-(+)-Sclaerolide	(3aR,5aS,9aS,9bR)-3a,6,6,9a-tetramethyl-dodecahydronaphtho[2,1-b]furan-2-one	250,4	564-20-5	NP-275	2mg/ml	DMSO	2865	Natural Products Library
**4**	4-E08	Streptonigrin	5-amino-6-(7-amino-6-methoxy-5,8-dioxo-5,8-dihydroquinolin-2-yl)-4-(2-hydroxy-3,4-dimethoxyphenyl)-3-methylpyridine-2-carboxylic acid	506,5	3930-19-6	NP-305	2mg/ml	DMSO	2865	Natural Products Library
**4**	4-E09	Tetrahydropapaverine·HCl	1-[(3,4-dimethoxyphenyl)methyl]-6,7-dimethoxy-1,2,3,4-tetrahydroisoquinoline hydrochloride	379,9	6429-04-5	NP-315	2mg/ml	DMSO	2865	Natural Products Library
**4**	4-E10	Ingenol	(4S,5R,6R,10R,12R,14R)-4,5,6-trihydroxy-7-(hydroxymethyl)-3,11,11,14-tetramethyltetracyclo[7.5.1.0^{1,5}.0^{10,12}]pentadeca-2,7-dien-15-one	348,4	30220-46-3	NP-525	2mg/ml	DMSO	2865	Natural Products Library
**4**	4-E11	Syrosingopine	methyl (1R,15S,17R,18R,19S,20S)-17-{4-[(ethoxycarbonyl)oxy]-3,5-dimethoxybenzoyloxy}-6,18-dimethoxy-3,13-diazapentacyclo[11.8.0.0^{2,10}.0^{4,9}.0^{15,20}]henicosa-2(10),4,6,8-tetraene-19-carboxylate	666,7	84-36-6	NP-574	2mg/ml	DMSO	2865	Natural Products Library
**4**	4-E12	Visnagin	4-methoxy-7-methyl-5H-furo[3,2-g]chromen-5-one	230,2	82-57-5	NP-334	2mg/ml	DMSO	2865	Natural Products Library

Table S2. ENZO – Natural Product library

Plate number	Plate Location	Name	IUPAC	MW	CAS	Catalog number	Conc	Solvent	Plate part number	Plate description
4	4-F01	Wogonin	5,7-dihydroxy-8-methoxy-2-phenyl-4H-chromen-4-one	284,3	632-85-9	NP-337	2mg/ml	DMSO	2865	Natural Products Library
4	4-F02	β-Zearalanol	(3S,7S)-7,14,16-trihydroxy-3-methyl-3,4,5,6,7,8,9,10,11,12-decahydro-1H-2-benzoxacyclotetradecin-1-one	322,4	42422-68-4	NP-340	2mg/ml	DMSO	2865	Natural Products Library
4	4-F03	4-Methylumbelliferone	7-hydroxy-4-methyl-2H-chromen-2-one	176,2	90-33-5	NP-547	2mg/ml	DMSO	2865	Natural Products Library
4	4-F04	Caffeine	1,3,7-trimethyl-2,3,6,7-tetrahydro-1H-purine-2,6-dione	194,2	58-08-2	ALX-550-322	2mg/ml	DMSO	2865	Natural Products Library
4	4-F05	Ellagic acid	6,7,13,14-tetrahydroxy-2,9-dioxatetracyclo$[6.6.2.0^{4,16}.0^{11,15}]$hexadeca-1(15),4,6,8(16),11,13-hexaene-3,10-dione	302,2	476-66-4	NP-091	2mg/ml	DMSO	2865	Natural Products Library
4	4-F06	(-)-Epicatechin	(2R,3R)-2-(3,4-dihydroxyphenyl)-3,4-dihydro-2H-1-benzopyran-3,5,7-triol	290,3	490-46-0	NP-095	2mg/ml	DMSO	2865	Natural Products Library
4	4-F07	Puromycin	(2S)-2-amino-N-[(2S,3S,4R,5R)-5-[6-(dimethylamino)-9H-purin-9-yl]-4-hydroxy-2-(hydroxymethyl)oxolan-3-yl]-3-(4-methoxyphenyl)propanamide	471,5	58-58-2	GR-312	2mg/ml	DMSO	2865	Natural Products Library
4	4-F08	18-β-Glycyrrhetinic acid	(2S,4aS,6aS,6bR,8aR,10S,12aS,12bR,14bR)-10-hydroxy-2,4a,6a,6b,9,9,12a-heptamethyl-13-oxo-1,2,3,4,4a,5,6,6a,6b,7,8,8a,9,10,11,12,12a,12b,13,14b-icosahydropicene-2-carboxylic acid	470,7	471-53-4	NP-116	2mg/ml	DMSO	2865	Natural Products Library
4	4-F09	(+)-Griseofulvin	(2S,6'R)-7-chloro-2',4,6-trimethoxy-6'-methyl-3H-spiro[1-benzofuran-2,1'-cyclohexan]-2'-ene-3,4'-dione	352,8	126-07-8	NP-119	2mg/ml	DMSO	2865	Natural Products Library
4	4-F10	Isoquercitrine	3-{[(2S,3R,4R)-5-[(1R)-1,2-dihydroxyethyl]-3,4-dihydroxyoxolan-2-yl]oxy}-2-(3,4-dihydroxyphenyl)-5,7-dihydroxy-4H-chromen-4-one	464,4	21637-25-2	NP-146	2mg/ml	DMSO	2865	Natural Products Library
4	4-F11	Kinetin	N-(furan-2-ylmethyl)-7H-purin-6-amine	215,2	525-79-1	NP-163	2mg/ml	DMSO	2865	Natural Products Library
4	4-F12	Lasalocid A	sodium 6-[(3R,4S,5S,7R)-7-[(3S,5S)-5-ethyl-5-[(5R,6S)-5-ethyl-5-hydroxy-6-methyloxan-2-yl]-3-methyloxolan-2-yl]-4-hydroxy-3,5-dimethyl-6-oxononyl]-2-hydroxy-3-methylbenzoate	612,8	25999-20-6	NP-167	2mg/ml	DMSO	2865	Natural Products Library
4	4-G01	Vannilylacetone	4-(4-hydroxy-3-methoxyphenyl)butan-2-one	194,2	122-48-5	NP-560	2mg/ml	DMSO	2865	Natural Products Library
4	4-G02	Sclareol	(1R,2R,4aS,8aS)-1-[(3S)-3-hydroxy-3-methylpent-4-en-1-yl]-2,5,5,8a-tetramethyl-decahydronaphthalen-2-ol	308,5	515-03-7	NP-274	2mg/ml	DMSO	2865	Natural Products Library
4	4-G03	Trigonelline·HCl	3-carboxy-1-methylpyridin-1-ium hydrochloride	174,6	6138-41-6	NP-322	2mg/ml	DMSO	2865	Natural Products Library
4	4-G04	Tubericidin	(2R,4R,5R)-2-{4-amino-7H-pyrrolo[2,3-d]pyrimidin-7-yl}-5-(hydroxymethyl)oxolane-3,4-diol	266,3	69-33-0	NP-327	2mg/ml	DMSO	2865	Natural Products Library
4	4-G05	(+)-Usnic acid	(2R)-4,10-diacetyl-3,11,13-trihydroxy-2,12-dimethyl-8-oxatricyclo$[7.4.0.0^{2,7}]$trideca-1(9),3,6,10,12-pentaen-5-one	344,3	7562-61-0	NP-330	2mg/ml	DMSO	2865	Natural Products Library
4	4-G06	Vitexin	5,7-dihydroxy-2-(4-hydroxyphenyl)-8-[(2S,3R,4R,5S,6R)-3,4,5-trihydroxy-6-(hydroxymethyl)oxan-2-yl]-4H-chromen-4-one	432,4	3681-93-4	NP-335	2mg/ml	DMSO	2865	Natural Products Library
4	4-G07	Acacetine	5,7-dihydroxy-2-(4-methoxyphenyl)-4H-chromen-4-one	284,3	480-44-4	NP-002	2mg/ml	DMSO	2865	Natural Products Library

Table S2. ENZO – Natural Product library

Plate number	Plate Location	Name	IUPAC	MW	CAS	Catalog number	Conc	Solvent	Plate part number	Plate description
**4**	4-G08	Capreomycin	3,6-diamino-N-{[(8Z)-15-amino-8-[(carbamoylamino)methylidene]-11-(2-imino-1,3-diazinan-4-yl)-2-methyl-3,6,9,12,16-pentaoxo-1,4,7,10,13-pentaazacyclohexadecan-5-yl]methyl}hexanamide	652,7	1405-37-4	NP-042	2mg/ml	DMSO	2865	Natural Products Library
**4**	4-G09	(±)-Carnitine	3-hydroxy-4-(trimethylazaniumyl)butanoate hydrochloride	197,7	461-05-2	NP-044	2mg/ml	DMSO	2865	Natural Products Library
**4**	4-G10	Cephadrine	(6R,7R)-7-[(2R)-2-amino-2-(cyclohexa-1,4-dien-1-yl)acetamido]-3-methyl-8-oxo-5-thia-1-azabicyclo[4.2.0]oct-2-ene-2-carboxylic acid	349,4	38821-53-3	NP-048	2mg/ml	DMSO	2865	Natural Products Library
**4**	4-G11	Vasicine	1H,2H,3H,9H-pyrrolo[2,1-b]quinazolin-3-ol	188,2	50591-64-5	NP-450	2mg/ml	DMSO	2865	Natural Products Library
**4**	4-G12	Homatropine·HBr	8-methyl-8-azabicyclo[3.2.1]octan-3-yl 2-hydroxy-2-phenylacetate hydrobromide	356,3	51-56-9	NP-131	2mg/ml	DMSO	2865	Natural Products Library
**4**	4-H01	D-β-Hydrastine	(3S)-6,7-dimethoxy-3-[(5R)-6-methyl-2H,5H,6H,7H,8H-[1,3]dioxolo[4,5-g]isoquinolin-5-yl]-1,3-dihydro-2-benzofuran-1-one	383,4	118-08-1	NP-136	2mg/ml	DMSO	2865	Natural Products Library
**4**	4-H02	Khellin	4,9-dimethoxy-7-methyl-5H-furo[3,2-g]chromen-5-one	260,2	82-02-0	NP-162	2mg/ml	DMSO	2865	Natural Products Library
**4**	4-H03	Lobeline·HCl	2-[(2R,6S)-6-[(2S)-2-hydroxy-2-phenylethyl]-1-methylpiperidin-2-yl]-1-phenylethan-1-one hydrochloride	373,9	134-63-4	AC-227	2mg/ml	DMSO	2865	Natural Products Library
**4**	4-H04	Osthole	7-methoxy-8-(3-methylbut-2-en-1-yl)-2H-chromen-2-one	244,3	484-12-8	NP-522	2mg/ml	DMSO	2865	Natural Products Library
**4**	4-H05	Tetrahydrolipistatin	(2S)-1-[(2S,3S)-3-hexyl-4-oxooxetan-2-yl]tridecan-2-yl (2S)-2-formamido-4-methylpentanoate	495,7	96829-58-2	NP-521	2mg/ml	DMSO	2865	Natural Products Library
**4**	4-H06	Neohesperidin	(2S)-7-{[(2S,3R,4S,5S,6R)-4,5-dihydroxy-6-(hydroxymethyl)-3-{[(3R,4R,5R,6S)-3,4,5-trihydroxy-6-methyloxan-2-yl]oxy}oxan-2-yl]oxy}-5-hydroxy-2-(3-hydroxy-4-methoxyphenyl)-3,4-dihydro-2H-1-benzopyran-4-one	610,6	13241-33-3	NP-205	2mg/ml	DMSO	2865	Natural Products Library
**4**	4-H07	(±)-Noscapine	(3S)-6,7-dimethoxy-3-[(5R)-4-methoxy-6-methyl-2H,5H,6H,7H,8H-[1,3]dioxolo[4,5-g]isoquinolin-5-yl]-1,3-dihydro-2-benzofuran-1-one	413,4	128-62-1	NP-211	2mg/ml	DMSO	2865	Natural Products Library
**4**	4-H08	Oleanolic acid	(4aS,6aS,6bR,8aR,10S,12aR,12bR,14bS)-10-hydroxy-2,2,6a,6b,9,9,12a-heptamethyl-1,2,3,4,4a,5,6,6a,6b,7,8,8a,9,10,11,12,12a,12b,13,14b-icosahydropicene-4a-carboxylic acid	456,7	508-02-1	NP-213	2mg/ml	DMSO	2865	Natural Products Library
**4**	4-H09	Papaverine·HCl	1-[(3,4-dimethoxyphenyl)methyl]-6,7-dimethoxyisoquinoline hydrochloride	375,8	61-25-6	NP-221	2mg/ml	DMSO	2865	Natural Products Library
**4**	4-H10	Phlorizine	1-(2,4-dihydroxy-6-{[(2S,3R,4S,5S,6R)-3,4,5-trihydroxy-6-(hydroxymethyl)oxan-2-yl]oxy}phenyl)-3-(4-hydroxyphenyl)propan-1-one	436,4	60-81-1	NP-229	2mg/ml	DMSO	2865	Natural Products Library
**4**	4-H11	Protoveratrine B	(1S,2S,6S,9S,10S,11R,12R,13S,14S,15S,16S,17R,18R,19S,22S,23R,25R)-16,17-bis(acetyloxy)-10,12,14,23-tetrahydroxy-6,10,19-trimethyl-13-{[(2R)-2-methylbutanoyl]oxy}-24-oxa-4-azaheptacyclo[12.12.0.$0^{2,11}$.$0^{4,9}$.$0^{15,25}$.$0^{18,23}$.$0^{19,25}$]hexacosan-22-yl 2,3-dihydroxy-2-methylbutanoate	809,9	124-97-0	NP-243	2mg/ml	DMSO	2865	Natural Products Library

Table S2. ENZO – Natural Product library

Plate number	Plate Location	Name	IUPAC	MW	CAS	Catalog number	Conc	Solvent	Plate part number	Plate description
**4**	4-H12	Reserpine	methyl (1R,15S,17R,18R,19S,20S)-6,18-dimethoxy-17-(3,4,5-trimethoxybenzoyloxy)-3,13-diazapentacyclo[11.8.0.0^{2,10}.0^{4,9}.0^{15,20}]henicosa-2(10),4,6,8-tetraene-19-carboxylate	608,7	50-55-5	NP-254	2mg/ml	DMSO	2865	Natural Products Library
**5**	5-A01	Salinomycin	(2R)-2-[(2R,5S,6R)-6-[(2S,3S,4S,6R)-6-[(2S,5S,7R,9S,10S,12R,15R)-2-[(2R,5R,6S)-5-ethyl-5-hydroxy-6-methyloxan-2-yl]-15-hydroxy-2,10,12-trimethyl-1,6,8-trioxadispiro[4.1.5^{7}.3^{5}]pentadec-13-en-9-yl]-3-hydroxy-4-methyl-5-oxooctan-2-yl]-5-methyloxan-2-yl]butanoic acid	751,0	53003-10-4	NP-267	2mg/ml	DMSO	2865	Natural Products Library
**5**	5-A02	Xanthotoxin	9-methoxy-7H-furo[3,2-g]chromen-7-one	216,2	298-81-7	NP-554	2mg/ml	DMSO	2865	Natural Products Library
**5**	5-A03	Scopoletin	7-hydroxy-6-methoxy-2H-chromen-2-one	192,2	92-61-5	NP-282	2mg/ml	DMSO	2865	Natural Products Library
**5**	5-A04	Digitoxin	4-[(1S,2S,5S,7R,10R,11S,14R,15R)-5-{[(2R,4S,5S,6R)-5-{[(2S,4S,5S,6R)-5-{[(2S,4S,5S,6R)-4,5-dihydroxy-6-methyloxan-2-yl]oxy}-4-hydroxy-6-methyloxan-2-yl]oxy}-4-hydroxy-6-methyloxan-2-yl]oxy}-11-hydroxy-2,15-dimethyltetracyclo[8.7.0.0^{2,7}.0^{11,15}]heptadecan-14-yl]-2,5-dihydrofuran-2-one	764,9	71-63-6	NP-548	2mg/ml	DMSO	2865	Natural Products Library
**5**	5-A05	α-Solanine	(2R,3R,4R,5R,6S)-2-{[(2R,3R,4S,5S,6R)-5-hydroxy-6-(hydroxymethyl)-2-{[(1S,2S,7S,10R,11S,14S,15R,16S,17R,20S,23S)-10,14,16,20-tetramethyl-22-azahexacyclo[12.10.0.0^{2,11}.0^{5,10}.0^{15,23}.0^{17,22}]tetracos-4-en-7-yl]oxy}-4-{[(2S,3R,4S,5S,6R)-3,4,5-trihydroxy-6-(hydroxymethyl)oxan-2-yl]oxy}oxan-3-yl]oxy}-6-methyloxane-3,4,5-triol	868,1	20562-02-1	NP-298	2mg/ml	DMSO	2865	Natural Products Library
**5**	5-A06	Solasodine	(1S,2S,4S,5'R,7R,8R,9S,12S,13R,16S)-5',7,9,13-tetramethyl-5-oxaspiro[pentacyclo[10.8.0.0^{2,9}.0^{4,8}.0^{13,18}]icosane-6,2'-piperidin]-18-en-16-ol	413,6	80-78-4	NP-299	2mg/ml	DMSO	2865	Natural Products Library
**5**	5-A07	Tropine	8-methyl-8-azabicyclo[3.2.1]octan-3-ol	141,2	120-29-6	NP-324	2mg/ml	DMSO	2865	Natural Products Library
**5**	5-A08	(+)-Tubocurarine chloride	(1S,16R)-9,21-dihydroxy-10,25-dimethoxy-15,15,30-trimethyl-7,23-dioxa-15,30-diazaheptacyclo[22.6.2.2^{3,6}.1^{8,12}.1^{18,22}.0^{27,31}.0^{16,34}]hexatriaconta-3,5,8,10,12(34),18,20,22(33),24(32),25,27(31),35-dodecaene-15,30-diium chloride hydrochloride	682,7	57-94-3	AC-746	2mg/ml	DMSO	2865	Natural Products Library
**5**	5-A09	Myristicin	4-methoxy-6-(prop-2-en-1-yl)-2H-1,3-benzodioxole	192,2	607-91-0	NP-520	2mg/ml	DMSO	2865	Natural Products Library
**5**	5-A10	Vincamine	methyl (15S,17S,19S)-15-ethyl-17-hydroxy-1,11-diazapentacyclo[9.6.2.0^{2,7}.0^{8,18}.0^{15,19}]nonadeca-2(7),3,5,8(18)-tetraene-17-carboxylate	354,4	1617-90-9	NP-332	2mg/ml	DMSO	2865	Natural Products Library
**5**	5-A11	(±)-Anabasine	3-(piperidin-2-yl)pyridine	162,2	15251-47-5	NP-007	2mg/ml	DMSO	2865	Natural Products Library
**5**	5-A12	Cephaeline·HBr	(1R)-1-{[(2S,3R,11bS)-3-ethyl-9,10-dimethoxy-1H,2H,3H,4H,6H,7H,11bH-pyrido[2,1-a]isoquinolin-2-yl]methyl}-7-methoxy-1,2,3,4-tetrahydroisoquinolin-6-ol hydrobromide	547,5	483-17-0	NP-569	2mg/ml	DMSO	2865	Natural Products Library

Table S2. ENZO – Natural Product library

Plate number	Plate Location	Name	IUPAC	MW	CAS	Catalog number	Conc	Solvent	Plate part number	Plate description
5	5-B01	Dicoumarol	4-hydroxy-3-[(4-hydroxy-2-oxo-2H-chromen-3-yl)methyl]-2H-chromen-2-one	336,3	66-76-2	NP-535	2mg/ml	DMSO	2865	Natural Products Library
5	5-B02	Artemesinin	(1S,4S,5R,8S,9R,12S,13R)-1,5,9-trimethyl-11,14,15,16-tetraoxatetracyclo[10.3.1.$0^{4,13}$.$0^{8,13}$]hexadecan-10-one	282,3	63968-64-9	NP-016	2mg/ml	DMSO	2865	Natural Products Library
5	5-B03	Asiatic acid	(1S,2R,4aS,6aS,6bR,8aR,9R,10R,11R,12aR,12bR,14bS)-10,11-dihydroxy-9-(hydroxymethyl)-1,2,6a,6b,9,12a-hexamethyl-1,2,3,4,4a,5,6,6a,6b,7,8,8a,9,10,11,12,12a,12b,13,14b-icosahydropicene-4a-carboxylic acid	488,7	464-92-6	NP-412	2mg/ml	DMSO	2865	Natural Products Library
5	5-B04	Auraptene	7-{[(2E)-3,7-dimethylocta-2,6-dien-1-yl]oxy}-2H-chromen-2-one	298,4	495-02-3	NP-413	2mg/ml	DMSO	2865	Natural Products Library
5	5-B05	Vulpinic acid	methyl 2-[(2E)-3-hydroxy-5-oxo-4-phenyl-2,5-dihydrofuran-2-ylidene]-2-phenylacetate	322,3	521-52-8	NP-553	2mg/ml	DMSO	2865	Natural Products Library
5	5-B06	Berberine·HCl	16,17-dimethoxy-5,7-dioxa-13λ^5-azapentacyclo[11.8.0.$0^{2,10}$.$0^{4,8}$.$0^{15,20}$]henicosa-1(21),2,4(8),9,13,15(20),16,18-octaen-13-ylium hydrochloride	372,8	633-65-8	NP-027	2mg/ml	DMSO	2865	Natural Products Library
5	5-B07	Bergenin	(2S,4R,5S,6S,7R)-5,6,12,14-tetrahydroxy-4-(hydroxymethyl)-13-methoxy-3,8-dioxatricyclo[8.4.0.$0^{2,7}$]tetradeca-1(14),10,12-trien-9-one	328,3	477-90-7	NP-414	2mg/ml	DMSO	2865	Natural Products Library
5	5-B08	Biochanin A	5,7-dihydroxy-3-(4-methoxyphenyl)-4H-chromen-4-one	284,3	491-80-5	NP-034	2mg/ml	DMSO	2865	Natural Products Library
5	5-B09	Bulleyaconotine A	(4R,5S,8R,9R,10S,18R)-8-(acetyloxy)-11-ethyl-5-hydroxy-6,16,18-trimethoxy-13-(methoxymethyl)-11-azahexacyclo[7.7.2.$1^{2,5}$.$0^{1,10}$.$0^{3,8}$.$0^{13,17}$]nonadecan-4-yl 4-methoxybenzoate	643,8	107668-79-1	NP-416	2mg/ml	DMSO	2865	Natural Products Library
5	5-B10	Cafestol	(1S,4S,12S,13R,16R,17R)-17-(hydroxymethyl)-12-methyl-8-oxapentacyclo[14.2.1.$0^{1,13}$.$0^{4,12}$.$0^{5,9}$]nonadeca-5(9),6-dien-17-ol	316,4	469-83-0	NP-418	2mg/ml	DMSO	2865	Natural Products Library
5	5-B11	Cafestol acetate	[(4S,12S,13R,16R,17R)-17-hydroxy-12-methyl-8-oxapentacyclo[14.2.1.$0^{1,13}$.$0^{4,12}$.$0^{5,9}$]nonadeca-5(9),6-dien-17-yl]methyl acetate	358,5	81760-48-7	NP-419	2mg/ml	DMSO	2865	Natural Products Library
5	5-B12	Zerumbone	(2E,6E,10E)-2,6,9,9-tetramethylcycloundeca-2,6,10-trien-1-one	218,3	471-05-6	NP-570	2mg/ml	DMSO	2865	Natural Products Library
5	5-C01	Catharanthine	methyl (1R)-17-ethyl-3,13-diazapentacyclo[13.3.1.$0^{2,10}$.$0^{4,9}$.$0^{13,18}$]nonadeca-2(10),4(9),5,7,16-pentaene-1-carboxylate	336,4	2468-21-5	NP-421	2mg/ml	DMSO	2865	Natural Products Library
5	5-C02	Cepharanthine	(14S,27R)-22,33-dimethoxy-13,28-dimethyl-2,5,7,20-tetraoxa-13,28-diazaoctacyclo[25.6.2.$2^{16,19}$.$1^{3,10}$.$1^{21,25}$.$0^{4,8}$.$0^{31,35}$.$0^{14,39}$]nonatriaconta-1(34),3,8,10(39),16,18,21,23,25(36),31(35),32,37-dodecaene	606,7	481-49-2	NP-404	2mg/ml	DMSO	2865	Natural Products Library
5	5-C03	Cryptotanshinone	(14R)-6,6,14-trimethyl-12-oxatetracyclo[8.7.0.$0^{2,7}$.$0^{11,15}$]heptadeca-1,7,9,11(15)-tetraene-16,17-dione	296,4	35825-57-1	NP-422	2mg/ml	DMSO	2865	Natural Products Library
5	5-C04	5,6-Dehydrokawain	4-methoxy-6-[(E)-2-phenylethenyl]-2H-pyran-2-one	228,2	15345-89-8	NP-423	2mg/ml	DMSO	2865	Natural Products Library

Table S2. ENZO – Natural Product library

Plate number	Plate Location	Name	IUPAC	MW	CAS	Catalog number	Conc	Solvent	Plate part number	Plate description
**5**	5-C05	4'-Demethylpipodophyllotoxin	(10R,11R,15R,16S)-16-hydroxy-10-(4-hydroxy-3,5-dimethoxyphenyl)-4,6,13-trioxatetracyclo[7.7.0.0^{3,7}.0^{11,15}]hexadeca-1,3(7),8-trien-12-one	400,4	6559-91-7	NP-424	2mg/ml	DMSO	2865	Natural Products Library
**5**	5-C06	Mitomycin C	[(4S,6S,7R,8S)-11-amino-7-methoxy-12-methyl-10,13-dioxo-2,5-diazatetracyclo[7.4.0.0^{2,7}.0^{4,6}]trideca-1(9),11-dien-8-yl]methyl carbamate	334,3	50-07-7	GR-311	2mg/ml	DMSO	2865	Natural Products Library
**5**	5-C07	Methysticin	(6S)-6-[(E)-2-(2H-1,3-benzodioxol-5-yl)ethenyl]-4-methoxy-5,6-dihydro-2H-pyran-2-one	274,3	495-85-2	NP-518	2mg/ml	DMSO	2865	Natural Products Library
**5**	5-C08	Thymoquinone	2-methyl-5-(propan-2-yl)cyclohexa-2,5-diene-1,4-dione	164,2	490-91-5	NP-536	2mg/ml	DMSO	2865	Natural Products Library
**5**	5-C09	Dihydrotanshinone	(14R)-6,14-dimethyl-12-oxatetracyclo[8.7.0.0^{2,7}.0^{11,15}]heptadeca-1(10),2(7),3,5,8,11(15)-hexaene-16,17-dione	278,3	20958-18-3	NP-428	2mg/ml	DMSO	2865	Natural Products Library
**5**	5-C10	Azomycin	2-nitro-1H-imidazole	113,1	527-73-1	NP-533	2mg/ml	DMSO	2865	Natural Products Library
**5**	5-C11	Diosmetine	5,7-dihydroxy-2-(3-hydroxy-4-methoxyphenyl)-4H-chromen-4-one	300,3	520-34-3	NP-082	2mg/ml	DMSO	2865	Natural Products Library
**5**	5-C12	Diosmin	5-hydroxy-2-(3-hydroxy-4-methoxyphenyl)-7-{[(2S,3R,4S,5S,6R)-3,4,5-trihydroxy-6-({[(2R,3R,4R,5R,6S)-3,4,5-trihydroxy-6-methyloxan-2-yl]oxy}methyl)oxan-2-yl]oxy}-4H-chromen-4-one	608,5	520-27-4	NP-084	2mg/ml	DMSO	2865	Natural Products Library
**5**	5-D01	Ecdysone	(1R,2R,4S,5R,7R,11S,14R,15R)-14-[(2S,3R)-3,6-dihydroxy-6-methylheptan-2-yl]-4,5,11-trihydroxy-2,15-dimethyltetracyclo[8.7.0.0^{2,7}.0^{11,15}]heptadec-9-en-8-one	464,6	3604-87-3	NP-089	2mg/ml	DMSO	2865	Natural Products Library
**5**	5-D02	β-Ecdysone	(1R,2R,4S,5R,7R,11S,14S,15R)-4,5,11-trihydroxy-2,15-dimethyl-14-[(2S,3R)-2,3,6-trihydroxy-6-methylheptan-2-yl]tetracyclo[8.7.0.0^{2,7}.0^{11,15}]heptadec-9-en-8-one	480,6	5289-74-7	NP-430	2mg/ml	DMSO	2865	Natural Products Library
**5**	5-D03	Euphorbiasteroid	(1'R,2R,3'E,5'R,7'S,11'S,12'R,13'S,14'S)-1',11'-bis(acetyloxy)-3',6',6',14'-tetramethyl-2'-oxospiro[oxirane-2,10'-tricyclo[10.3.0.0^{5,7}]pentadecan]-3'-en-13'-yl 2-phenylacetate	552,7	28649-59-4	NP-431	2mg/ml	DMSO	2865	Natural Products Library
**5**	5-D04	Flavokawain A	(2E)-1-(4-hydroxy-2,6-dimethoxyphenyl)-3-(4-methoxyphenyl)prop-2-en-1-one	314,3	3420-72-2	NP-432	2mg/ml	DMSO	2865	Natural Products Library
**5**	5-D05	Lupinine	(1R,9aR)-octahydro-1H-quinolizin-1-ylmethanol	169,3	545-47-1	NP-516	2mg/ml	DMSO	2865	Natural Products Library
**5**	5-D06	Formononetin	7-hydroxy-3-(4-methoxyphenyl)-4H-chromen-4-one	268,3	485-72-3	NP-106	2mg/ml	DMSO	2865	Natural Products Library
**5**	5-D07	Ginkgolide A	(3S,6R,7S,8S,11S,13S,16S,17R)-8-tert-butyl-6,17-dihydroxy-16-methyl-2,4,14,19-tetraoxahexacyclo[8.7.2.0^{1,11}.0^{3,7}.0^{7,11}.0^{13,17}]nonadecane-5,15,18-trione	408,4	15291-75-5	NP-342	2mg/ml	DMSO	2865	Natural Products Library
**5**	5-D08	Harringtonine	(2R,3S,6R)-4-methoxy-16,18-dioxa-10-azapentacyclo[11.7.0.0^{2,6}.0^{6,10}.0^{15,19}]icosa-1(13),4,14,19-tetraen-3-yl 1-methyl (3S)-3-hydroxy-3-(3-hydroxy-3-methylbutyl)butanedioate	531,6	26833-85-2	NP-435	2mg/ml	DMSO	2865	Natural Products Library
**5**	5-D09	Hesperitine	(2S)-5,7-dihydroxy-2-(3-hydroxy-4-methoxyphenyl)-3,4-dihydro-2H-1-benzopyran-4-one	302,3	520-33-2	NP-127	2mg/ml	DMSO	2865	Natural Products Library

Table S2. ENZO – Natural Product library

Plate number	Plate Location	Name	IUPAC	MW	CAS	Catalog number	Conc	Solvent	Plate part number	Plate description
**5**	5-D10	Hesperidine	(2S)-5-hydroxy-2-(3-hydroxy-4-methoxyphenyl)-7-{[(2S,3R,4S,5S,6R)-3,4,5-trihydroxy-6-({[(2R,3R,4R,5R,6S)-3,4,5-trihydroxy-6-methyloxan-2-yl]oxy}methyl)oxan-2-yl]oxy}-3,4-dihydro-2H-1-benzopyran-4-one	610,6	520-26-3	NP-128	2mg/ml	DMSO	2865	Natural Products Library
**5**	5-D11	Honokiol	2-[4-hydroxy-3-(prop-2-en-1-yl)phenyl]-4-(prop-2-en-1-yl)phenol	266,3	35354-74-6	NP-135	2mg/ml	DMSO	2865	Natural Products Library
**5**	5-D12	Hypocrellin A	(12S,13R)-12-acetyl-9,13,19-trihydroxy-5,10,16,21-tetramethoxy-13-methylhexacyclo[13.8.0.0^{2},^{11}.0^{3},^{8}.0^{4},^{22}.0^{18},^{23}]tricosa-1,3,5,8,10,15,18(23),19,21-nonaene-7,17-dione	546,5	77029-83-5	NP-437	2mg/ml	DMSO	2865	Natural Products Library
**5**	5-E01	Hypocrellin B	7-acetyl-14,21-dihydroxy-6,12,17,19-tetramethoxy-9-methylhexacyclo[13.8.0.0^{2},^{11}.0^{3},^{8}.0^{4},^{22}.0^{18},^{23}]tricosa-1(23),2,4(22),6,8,11,14,16,18,20-decaene-5,13-dione	528,5	123940-54-5	NP-438	2mg/ml	DMSO	2865	Natural Products Library
**5**	5-E02	Lagochiline	(1S,2R,5R,6S,8aS)-5'-(2-hydroxyethyl)-5,5'-bis(hydroxymethyl)-2,5,8a-trimethyl-octahydro-2H-spiro[naphthalene-1,2'-oxolane]-6-ol	356,5	23554-81-6	NP-439	2mg/ml	DMSO	2865	Natural Products Library
**5**	5-E03	Lappaconitine	(1S,4S,5R,8S,9S,10R,13S)-11-ethyl-3,8-dihydroxy-4,6,16-trimethoxy-11-azahexacyclo[7.7.2.1^{2},^{5}.0^{1},^{10}.0^{3},^{8}.0^{13},^{17}]nonadecan-13-yl 2-acetamidobenzoate	584,7	32854-75-4	NP-166	2mg/ml	DMSO	2865	Natural Products Library
**5**	5-E04	Limonin	(1R,2R,7S,10R,13R,14R,19R,20S)-19-(furan-3-yl)-9,9,13,20-tetramethyl-4,8,15,18-tetraoxahexacyclo[11.9.0.0^{2},^{7}.0^{2},^{10}.0^{14},^{16}.0^{14},^{20}]docosane-5,12,17-trione	470,5	1180-71-8	NP-440	2mg/ml	DMSO	2865	Natural Products Library
**5**	5-E05	Madecassic acid	(1S,2R,4aS,6aS,6bR,8R,8aR,9R,10R,11R,12aR,12bR,14bS)-8,10,11-trihydroxy-9-(hydroxymethyl)-1,2,6a,6b,9,12a-hexamethyl-1,2,3,4,4a,5,6,6a,6b,7,8,8a,9,10,11,12,12a,12b,13,14b-icosahydropicene-4a-carboxylic acid	504,7	18449-41-7	NP-442	2mg/ml	DMSO	2865	Natural Products Library
**5**	5-E06	Magnolol	2-[2-hydroxy-5-(prop-2-en-1-yl)phenyl]-4-(prop-2-en-1-yl)phenol	266,3	528-43-8	NP-181	2mg/ml	DMSO	2865	Natural Products Library
**5**	5-E07	Matrine	(1R,2R,9S,17S)-7,13-diazatetracyclo[7.7.1.0^{2},^{7}.0^{13},^{17}]heptadecan-6-one	248,4	519-02-8	NP-443	2mg/ml	DMSO	2865	Natural Products Library
**5**	5-E08	Minocycline·HCl	(4S,4aS,5aR,12aS)-4,7-bis(dimethylamino)-3,10,12,12a-tetrahydroxy-1,11-dioxo-1,4,4a,5,5a,6,11,12a-octahydrotetracene-2-carboxamide hydrochloride	493,9	13614-98-7	NP-191	2mg/ml	DMSO	2865	Natural Products Library
**5**	5-E09	Naringin	(2S)-7-{[(2S,3R,4S,5S,6R)-4,5-dihydroxy-6-(hydroxymethyl)-3-{[(2S,3R,4R,5R,6S)-3,4,5-trihydroxy-6-methyloxan-2-yl]oxy}oxan-2-yl]oxy}-5-hydroxy-2-(4-hydroxyphenyl)-3,4-dihydro-2H-1-benzopyran-4-one	580,5	10236-47-2	NP-202	2mg/ml	DMSO	2865	Natural Products Library
**5**	5-E10	Indole-3-acetic acid	2-(1H-indol-3-yl)acetic acid	175,2	87-51-4	NP-546	2mg/ml	DMSO	2865	Natural Products Library

Table S2. ENZO – Natural Product library

Plate number	Plate Location	Name	IUPAC	MW	CAS	Catalog number	Conc	Solvent	Plate part number	Plate description
**5**	5-E11	16-Oxocafestol	(1S,4S,12S,13R)-12-methyl-8-oxapentacyclo[14.2.1.0^{1,13}.0^{4,12}.0^{5,9}]nonadeca-5(9),6-dien-17-one	284,4	108664-98-8	NP-446	2mg/ml	DMSO	2865	Natural Products Library
**5**	5-E12	16-Oxokahweol	(1S,4S,12S,13R)-12-methyl-8-oxapentacyclo[14.2.1.0^{1,13}.0^{4,12}.0^{5,9}]nonadeca-5(9),6,10-trien-17-one	282,4	108664-99-9	NP-447	2mg/ml	DMSO	2865	Natural Products Library
**5**	5-F01	Panaxadiol	(1R,2R,5S,7R,10R,11R,14S,15R,16R)-2,6,6,10,11-pentamethyl-14-[(2S)-2,6,6-trimethyloxan-2-yl]tetracyclo[8.7.0.0^{2,7}.0^{11,15}]heptadecane-5,16-diol	460,7	19666-76-3	NP-448	2mg/ml	DMSO	2865	Natural Products Library
**5**	5-F02	Panaxatriol	(1R,2R,5S,7R,8S,10R,11R,14S,15R,16R)-2,6,6,10,11-pentamethyl-14-[(2S)-2,6,6-trimethyloxan-2-yl]tetracyclo[8.7.0.0^{2,7}.0^{11,15}]heptadecane-5,8,16-triol	476,7	32791-84-7	NP-449	2mg/ml	DMSO	2865	Natural Products Library
**5**	5-F03	GERI-BP002-A	2-tert-butyl-6-[(3-tert-butyl-2-hydroxy-5-methylphenyl)methyl]-4-methylphenol	340,5	119-47-1	EI-363	2mg/ml	DMSO	2865	Natural Products Library
**5**	5-F04	Pimaricin	(1S,3R,5S,7S,8E,12R,14E,16E,18E,20E,24R,25S,26R)-22-{[(2R,3R,5S)-4-amino-3,5-dihydroxy-6-methyloxan-2-yl]oxy}-1,3,26-trihydroxy-12-methyl-10-oxo-6,11,28-trioxatricyclo[22.3.1.0^{5,7}]octacosa-8,14,16,18,20-pentaene-25-carboxylic acid	665,7	7681-93-8	NP-232	2mg/ml	DMSO	2865	Natural Products Library
**5**	5-F05	Podophyllotoxin	(10R,11R,16R)-16-hydroxy-10-(3,4,5-trimethoxyphenyl)-4,6,13-trioxatetracyclo[7.7.0.0^{3,7}.0^{11,15}]hexadeca-1(9),2,7-trien-12-one	414,4	518-28-5	NP-238	2mg/ml	DMSO	2865	Natural Products Library
**5**	5-F06	Rubescensin A	(1S,2S,5S,8R,9R,10R,11R,15S,18R)-9,10,15,18-tetrahydroxy-12,12-dimethyl-6-methylidene-17-oxapentacyclo[7.6.2.1^{5,8}.0^{1,11}.0^{2,8}]octadecan-7-one	364,4	28957-04-2	NP-451	2mg/ml	DMSO	2865	Natural Products Library
**5**	5-F07	Rutaecarpine	3,13,21-triazapentacyclo[11.8.0.0^{2,10}.0^{4,9}.0^{15,20}]henicosa-1(21),2(10),4(9),5,7,15,17,19-octaen-14-one	287,3	84-26-4	NP-452	2mg/ml	DMSO	2865	Natural Products Library
**5**	5-F08	Rutin	2-(3,4-dihydroxyphenyl)-5,7-dihydroxy-3-{[(2S,3R,4S,5S,6R)-3,4,5-trihydroxy-6-({[(2R,3R,4R,5R,6S)-3,4,5-trihydroxy-6-methyloxan-2-yl]oxy}methyl)oxan-2-yl]oxy}-4H-chromen-4-one	610,5	153-18-4	NP-266	2mg/ml	DMSO	2865	Natural Products Library
**5**	5-F09	Salsolodine	(1R)-6,7-dimethoxy-1-methyl-1,2,3,4-tetrahydroisoquinoline	207,3	493-48-1	NP-453	2mg/ml	DMSO	2865	Natural Products Library
**5**	5-F10	Salsoline	(1R)-7-methoxy-1-methyl-1,2,3,4-tetrahydroisoquinolin-6-ol	193,2	101467-40-7	NP-454	2mg/ml	DMSO	2865	Natural Products Library
**5**	5-F11	Santonin	(3S,3aS,5aS,9bS)-3,5a,9-trimethyl-2H,3H,3aH,4H,5H,5aH,8H,9bH-naphtho[1,2-b]furan-2,8-dione	246,3	481-06-1	NP-270	2mg/ml	DMSO	2865	Natural Products Library
**5**	5-F12	R(+)-Schisandrin A	3,4,5,14,15,16-hexamethoxy-9,10-dimethyltricyclo[10.4.0.0^{2,7}]hexadeca-1(12),2(7),3,5,13,15-hexaene	416,5	61281-38-7	NP-455	2mg/ml	DMSO	2865	Natural Products Library
**5**	5-G01	S(-)-Schisandrin A	3,4,5,19-tetramethoxy-9,10-dimethyl-15,17-dioxatetracyclo[10.7.0.0^{2,7}.0^{14,18}]nonadeca-1(12),2(7),3,5,13,18-hexaene	400,5	61281-37-6	NP-456	2mg/ml	DMSO	2865	Natural Products Library

Table S2. ENZO – Natural Product library

Plate number	Plate Location	Name	IUPAC	MW	CAS	Catalog number	Conc	Solvent	Plate part number	Plate description
**5**	5-G02	Schisantherin A	9-hydroxy-3,4,5,19-tetramethoxy-9,10-dimethyl-15,17-dioxatetracyclo[10.7.0.0^{2,7}.0^{14,18}]nonadeca-1(12),2(7),3,5,13,18-hexaen-8-yl benzoate	536,6	58546-56-8	NP-457	2mg/ml	DMSO	2865	Natural Products Library
**5**	5-G03	Securinine	(1S,2R,8S)-14-oxa-7-azatetracyclo[6.6.1.0^{1,11}.0^{2,7}]pentadeca-9,11-dien-13-one	217,3	5610-40-2	NP-285	2mg/ml	DMSO	2865	Natural Products Library
**5**	5-G04	Sedanolide	3-(2-methylpropyl)-1,3,3a,4,5,6-hexahydro-2-benzofuran-1-one	194,3	6415-59-4	NP-458	2mg/ml	DMSO	2865	Natural Products Library
**5**	5-G05	Silybine	(2R,3R)-3,5,7-trihydroxy-2-[(2R,3R)-3-(4-hydroxy-3-methoxyphenyl)-2-(hydroxymethyl)-2,3-dihydro-1,4-benzodioxin-6-yl]-3,4-dihydro-2H-1-benzopyran-4-one	482,4	22888-70-6	NP-291	2mg/ml	DMSO	2865	Natural Products Library
**5**	5-G06	Silymarin	(2S,3R)-3,5,7-trihydroxy-2-[(2R,3R)-3-(4-hydroxy-3-methoxyphenyl)-2-(hydroxymethyl)-2,3-dihydro-1,4-benzodioxin-6-yl]-3,4-dihydro-2H-1-benzopyran-4-one	482,4	65666-07-1	NP-459	2mg/ml	DMSO	2865	Natural Products Library
**5**	5-G07	Sinomenine	(1R,9S,10S)-3-hydroxy-4,12-dimethoxy-17-methyl-17-azatetracyclo[7.5.3.0^{1,10}.0^{2,7}]heptadeca-2(7),3,5,11-tetraen-13-one	329,4	115-53-7	NP-293	2mg/ml	DMSO	2865	Natural Products Library
**5**	5-G08	Solanesol	(2E,6E,10E,14E,18E,22E,26E,30E)-3,7,11,15,19,23,27,31,35-nonamethylhexatriaconta-2,6,10,14,18,22,26,30,34-nonaen-1-ol	631,1	13190-97-1	NP-296	2mg/ml	DMSO	2865	Natural Products Library
**5**	5-G09	Vindoline	methyl (1R,9R,10S,11R,12R,19R)-11-(acetyloxy)-12-ethyl-10-hydroxy-5-methoxy-8-methyl-8,16-diazapentacyclo[10.6.1.0^{1,9}.0^{2,7}.0^{16,19}]nonadeca-2(7),3,5,13-tetraene-10-carboxylate	456,5	2182-14-1	NP-460	2mg/ml	DMSO	2865	Natural Products Library
**5**	5-G10	Vinorelbine	methyl (1R,9R,10S,11R,12R,19R)-11-(acetyloxy)-12-ethyl-4-[(1R,12S,14R)-16-ethyl-12-(methoxycarbonyl)-1,10-diazatetracyclo[12.3.1.0^{3,11}.0^{4,9}]octadeca-3(11),4(9),5,7,15-pentaen-12-yl]-10-hydroxy-5-methoxy-8-methyl-8,16-diazapentacyclo[10.6.1.0^{1,9}.0^{2,7}.0^{16,19}]nonadeca-2(7),3,5,13-tetraene-10-carboxylate	778,9	71486-22-1	NP-461	2mg/ml	DMSO	2865	Natural Products Library
**5**	5-G11	Yangonin	4-methoxy-6-[(E)-2-(4-methoxyphenyl)ethenyl]-2H-pyran-2-one	258,3	500-62-9	NP-462	2mg/ml	DMSO	2865	Natural Products Library
**5**	5-G12	Bergapten	4-methoxy-7H-furo[3,2-g]chromen-7-one	216,2	484-20-8	NP-028	2mg/ml	DMSO	2865	Natural Products Library
**5**	5-H01	Betulin	(1R,2R,5S,8R,9R,10R,13R,14R,17S,19R)-5-(hydroxymethyl)-1,2,14,18,18-pentamethyl-8-(prop-1-en-2-yl)pentacyclo[11.8.0.0^{2,10}.0^{5,9}.0^{14,19}]henicosan-17-ol	442,7	473-98-3	NP-030	2mg/ml	DMSO	2865	Natural Products Library
**5**	5-H02	Corynanthine	methyl (1S,15R,18S,19S,20S)-18-hydroxy-3,13-diazapentacyclo[11.8.0.0^{2,10}.0^{4,9}.0^{15,20}]henicosa-2(10),4,6,8-tetraene-19-carboxylate	354,4	483-10-3	NP-062	2mg/ml	DMSO	2865	Natural Products Library
**5**	5-H03	(-)-Cytisine	(1R,9S)-7,11-diazatricyclo[7.3.1.0^{2,7}]trideca-2,4-dien-6-one	190,2	485-35-8	NP-066	2mg/ml	DMSO	2865	Natural Products Library
**5**	5-H04	Spartein sulfate·5H_2O	(1S,2R,9S,10S)-7,15-diazatetracyclo[7.7.1.0^{2,7}.0^{10,15}]heptadecane sulfuric acid pentahydrate	422,5	6160-12-9	NP-301	2mg/ml	DMSO	2865	Natural Products Library
**5**	5-H05	Brassinin	N-(1H-indol-3-ylmethyl)(methylsulfanyl)carbothioamide	236,4	105748-59-2	NP-415	2mg/ml	DMSO	2865	Natural Products Library

Table S2. ENZO – Natural Product library

Plate number	Plate Location	Name	IUPAC	MW	CAS	Catalog number	Conc	Solvent	Plate part number	Plate description
**5**	5-H06	Dihydrorobinetine	(2R,3R)-3,7-dihydroxy-2-(3,4,5-trihydroxyphenyl)-3,4-dihydro-2H-1-benzopyran-4-one	304,3	4382-33-6	NP-079	2mg/ml	DMSO	2865	Natural Products Library
**5**	5-H07	Flavanomarein	(2S)-2-(3,4-dihydroxyphenyl)-8-hydroxy-7-{[(2S,3R,4S,5S,6R)-3,4,5-trihydroxy-6-(hydroxymethyl)oxan-2-yl]oxy}-3,4-dihydro-2H-1-benzopyran-4-one	450,4	577-38-8	NP-105	2mg/ml	DMSO	2865	Natural Products Library
**5**	5-H08	Lavendustin B	5-{bis[(2-hydroxyphenyl)methyl]amino}-2-hydroxybenzoic acid	365,4	125697-91-8	EI-254	2mg/ml	DMSO	2865	Natural Products Library
**5**	5-H09	Evodiamine	21-methyl-3,13,21-triazapentacyclo[11.8.0.$0^{2,10}$.$0^{4,9}$.$0^{15,20}$]henicosa-2(10),4(9),5,7,15(20),16,18-heptaen-14-one	303,4	518-17-2	NP-101	2mg/ml	DMSO	2865	Natural Products Library
**5**	5-H10	Oxyacanthine sulfate	(1R,14S)-20,26-dimethoxy-15,31-dimethyl-8,24-dioxa-15,31-diazaheptacyclo[23.6.2.$2^{9,12}$.$1^{3,7}$.$1^{14,18}$.$0^{28,32}$.$0^{22,34}$]heptatriaconta-3,5,7(37),9,11,18,20,22(34),25(33),26,28(32),35-dodecaene-6,21-diol; sulfuric acid	706,8	548-40-3	NP-217	2mg/ml	DMSO	2865	Natural Products Library
**5**	5-H11	Galangine	3,5,7-trihydroxy-2-phenyl-4H-chromen-4-one	270,2	548-83-4	NP-109	2mg/ml	DMSO	2865	Natural Products Library
**5**	5-H12	Lavendustin A	5-{[(2,5-dihydroxyphenyl)methyl][(2-hydroxyphenyl)methyl]amino}-2-hydroxybenzoic acid	381,4	125697-92-9	EI-185	2mg/ml	DMSO	2865	Natural Products Library
**6**	6-A01	Verruculogen	(9S,14S,17S,23R,24S)-23,24-dihydroxy-5-methoxy-12,12-dimethyl-9-(2-methylprop-1-en-1-yl)-10,11-dioxa-8,15,21-triazahexacyclo[12.10.1.$0^{2,7}$.$0^{8,25}$.$0^{15,23}$.$0^{17,21}$]pentacosa-2(7),3,5-triene-16,22-dione	513,6	12771-72-1	NP-575	2mg/ml	DMSO	2865	Natural Products Library
**6**	6-A02	Gelsemine·HCl	(2'S,3S,6'S)-2'-ethenyl-4'-methyl-1,2-dihydro-9'-oxa-4'-azaspiro[indole-3,7'-tetracyclo[6.3.1.$0^{2,6}$.$0^{5,11}$]dodecane]-2-one hydrochloride	358,9	35306-33-3	NP-110	2mg/ml	DMSO	2865	Natural Products Library
**6**	6-A03	Hydrocotarnine HBr	4-methoxy-6-methyl-2H,5H,6H,7H,8H-[1,3]dioxolo[4,5-g]isoquinoline hydrobromide	302,2	550-10-7	NP-137	2mg/ml	DMSO	2865	Natural Products Library
**6**	6-A04	Senecionine	(1R,4Z,6R,7R,17R)-4-ethylidene-7-hydroxy-6,7-dimethyl-2,9-dioxa-14-azatricyclo[9.5.1.$0^{14,17}$]heptadec-11-ene-3,8-dione	335,4	130-01-8	NP-286	2mg/ml	DMSO	2865	Natural Products Library
**6**	6-A05	Bis demethoxycurcumin	(1E,6E)-1,7-bis(4-hydroxyphenyl)hepta-1,6-diene-3,5-dione	308,3	24939-16-0	NP-576	2mg/ml	DMSO	2865	Natural Products Library
**6**	6-A06	9,10-Dihydrolysergol	[(4R,7R)-6-methyl-6,11-diazatetracyclo[7.6.1.$0^{2,7}$.$0^{12,16}$]hexadeca-1(16),9,12,14-tetraen-4-yl]methanol	256,3	18051-16-6	NP-077	2mg/ml	DMSO	2865	Natural Products Library
**6**	6-A07	Amphotericin B	(1R,3R,5S,6S,9S,11S,15S,16R,17R,18S,19E,21E,23E,25E,27E,29E,31E,33R,35S,36R,37S)-33-{[(2R,3S,4S,5S,6R)-4-amino-3,5-dihydroxy-6-methyloxan-2-yl]oxy}-1,3,5,6,9,11,17,37-octahydroxy-15,16,18-trimethyl-13-oxo-14,39-dioxabicyclo[33.3.1]nonatriaconta-19,21,23,25,27,29,31-heptaene-36-carboxylic acid	924,1	1397-89-3	NP-500	2mg/ml	DMSO	2865	Natural Products Library

Table S2. ENZO – Natural Product library

Plate number	Plate Location	Name	IUPAC	MW	CAS	Catalog number	Conc	Solvent	Plate part number	Plate description
**6**	6-A08	Amygdalin	2-phenyl-2-{[(2R,3R,4S,5S,6R)-3,4,5-trihydroxy-6-({[(2R,3R,4S,5S,6R)-3,4,5-trihydroxy-6-(hydroxymethyl)oxan-2-yl]oxy}methyl)oxan-2-yl]oxy}acetonitrile	457,4	29883-15-6	NP-501	2mg/ml	DMSO	2865	Natural Products Library
**6**	6-A09	Anisodamine	6-hydroxy-8-methyl-8-azabicyclo[3.2.1]octan-3-yl 3-hydroxy-2-phenylpropanoate hydrobromide	386,3	55869-99-3	NP-502	2mg/ml	DMSO	2865	Natural Products Library
**6**	6-A10	Aphidicolin	(1S,2S,5R,6R,7R,10S,12R,13R)-6,13-bis(hydroxymethyl)-2,6-dimethyltetracyclo[10.3.1.$0^{1,10}$.$0^{2,7}$]hexadecane-5,13-diol	338,5	38966-21-1	CC-101	2mg/ml	DMSO	2865	Natural Products Library
**6**	6-A11	Arbutin	(2R,3S,4S,5R,6S)-2-(hydroxymethyl)-6-(4-hydroxyphenoxy)oxane-3,4,5-triol	272,3	497-76-7	NP-504	2mg/ml	DMSO	2865	Natural Products Library
**6**	6-A12	Sclerotiorin	(7S)-5-chloro-3-[(1E,3E,5S)-3,5-dimethylhepta-1,3-dien-1-yl]-7-methyl-6,8-dioxo-7,8-dihydro-6H-isochromen-7-yl acetate	390,9	549-23-5	NP-276	2mg/ml	DMSO	2865	Natural Products Library
**6**	6-B01	Bleomycin sulfate	(3-{[2-(2-{2-[(2R,3R)-2-[(2S,3S,4R)-4-[(2R,3R)-2-({6-amino-2-[(1S)-1-{[(2S)-2-amino-2-carbamoylethyl]amino}-2-carbamoylethyl]-5-methylpyrimidin-4-yl}formamido)-3-{[(2R,3S,4S,5S,6S)-3-{[(2S,3S,4R,5R,6R)-4-(carbamoyloxy)-3,5-dihydroxy-6-(hydroxymethyl)oxan-2-yl]oxy}-4,5-dihydroxy-6-(hydroxymethyl)oxan-2-yl]oxy}-3-(1H-imidazol-4-yl)propanamido]-3-hydroxy-2-methylpentanamido]-3-hydroxybutanamido]ethyl}-1,3-thiazol-4-yl)-1,3-thiazol-5-yl]formamido}propyl)dimethylsulfanium sulfate	1511,6	9041-93-4	AP-302	2mg/ml	DMSO	2865	Natural Products Library
**6**	6-B02	Chartreusin	3-{[(2S,3R,4S,5R,6R)-3-{[(2R,3R,4S,5S,6R)-3,5-dihydroxy-4-methoxy-6-methyloxan-2-yl]oxy}-4,5-dihydroxy-6-methyloxan-2-yl]oxy}-8-hydroxy-15-methyl-11,18-dioxapentacyclo[10.6.2.$0^{2,7}$.$0^{9,19}$.$0^{16,20}$]icosa-1(19),2(7),3,5,8,12(20),13,15-octaene-10,17-dione	640,6	6377-18-0	NP-506	2mg/ml	DMSO	2865	Natural Products Library
**6**	6-B03	Chlorogenic acid	(1S,3R,4R,5R)-3-{[(2E)-3-(3,4-dihydroxyphenyl)prop-2-enoyl]oxy}-1,4,5-trihydroxycyclohexane-1-carboxylic acid	354,3	327-97-9	NP-507	2mg/ml	DMSO	2865	Natural Products Library
**6**	6-B04	Geraldol	3,7-dihydroxy-2-(4-hydroxy-3-methoxyphenyl)-4H-chromen-4-one	300,3	21511-25-1	NP-112	2mg/ml	DMSO	2865	Natural Products Library
**6**	6-B05	Coumestrol	5,14-dihydroxy-8,17-dioxatetracyclo[8.7.0.$0^{2,7}$.$0^{11,16}$]heptadeca-1(10),2(7),3,5,11,13,15-heptaen-9-one	268,2	479-13-0	S-180	2mg/ml	DMSO	2865	Natural Products Library
**6**	6-B06	Diindolylmethane	3-(1H-indol-3-ylmethyl)-1H-indole	246,3	01968-05-4	GR-207	2mg/ml	DMSO	2865	Natural Products Library
**6**	6-B07	Ferulic acid	(2E)-3-(4-hydroxy-3-methoxyphenyl)prop-2-enoic acid	194,2	1135-24-6	NP-510	2mg/ml	DMSO	2865	Natural Products Library
**6**	6-B08	Bakuchiol	4-[(1E,3S)-3-ethenyl-3,7-dimethylocta-1,6-dien-1-yl]phenol	256,4	10309-37-2	NP-571	2mg/ml	DMSO	2865	Natural Products Library
**6**	6-B09	L-Theanine	(2R)-2-amino-4-(ethylcarbamoyl)butanoic acid	174,2	3081-61-6	AC-1565	2mg/ml	DMSO	2865	Natural Products Library
**6**	6-B10	Indole-3-carbinol	1H-indol-3-ylmethanol	147,2	700-06-1	NP-512	2mg/ml	DMSO	2865	Natural Products Library
