# Supplementary material for: Inhibiting eukaryotic ribosome biogenesis
Source: BMC Biol. 2019 Jun 10;17:46. doi: 10.1186/s12915-019-0664-2 (PMC6558755; doi:10.1186/s12915-019-0664-2)
Supplement: Supplementary file 3 — Table S3. All hits of the microscopy screen including a complete list of all changing pre-rRNAs (± > 1.5x) for substances listed in Table 1 and documented references to activities against cancer cells. (PDF 78 kb) [file 12915_2019_664_MOESM3_ESM.pdf]

Table S3: Complete list of identified substances.

*CAS-Nr. for the Enzo collection and the sample identifier for the NIH collection

**Complete list of all changing pre-rRNAs (± >1.5x) for substances listed in Table 1

***Documented activity against cancer cells. Only listed for substances showing the strongest rRNA processing defects (see Table 1)

No.	Substance	collection	ID*	Screen	rRNA pcocessing defects**	(reference)***
**1**	Acivicin	ENZO	42228-92-2	both	all precursors gone	(43)
**2**	Streptonigrin	ENZO	3930-19-6	both	all precursors gone	(44, 45)
**3**	Idarubicin HCl	NIH	SAM001246676	both	7S ↑	(46, 47)
**4**	(+)-Usnic acid	ENZO	7562-61-0	both	27S, 27SA$_2$, 20S, 7S and A$_2$-A$_3$ sp. ↑	(48, 49)
**5**	Antibiotic A-23187 (Calcimycin)	ENZO	52665-69-7	both	20S ↓	(50, 51)
**6**	Curcumin	ENZO	458-37-7	both	A$_2$-A$_3$ sp. ↓	(52)
**7**	Tanshinone IIA	ENZO	568-73-0	both	27S A$_2$ ↓, 23S ↑ 35S ↑	(53-55)
**8**	Morine	ENZO	480-16-0	both	35S ↑	(56)
**9**	Nonactin	ENZO	6833-84-7	both	20S ↓ and 35S ↑	(57)
**10**	Quercetin.2H_2O	ENZO	6151-25-3	both		
**11**	Kaempferol	ENZO	520-18-3	both		
**12**	Sinensetine	ENZO	2306-27-6	both		
**13**	Syringetine-3-glucoside	ENZO	40039-49-4	both	27S ↑	not documented
**14**	Berberine.HCl	ENZO	633-65-8	both	23S ↑	(58)
**15**	Senecionine	ENZO	130-01-8	both	35S ↑	not documented
**16**	Bleomycin sulfate	ENZO	9041-93-4	both	35S ↑	(59)
**17**	Carmofur	NIH	SAM001246692	60S	27S A$_2$ and 23S ↑	(60-62)
**18**	Vulpinic acid	ENZO	521-52-8	60S	7S, 23S and A$_2$-A$_3$ sp. ↑	(63)
**19**	Mycophenolic acid	ENZO	24280-93-1	60S	all precursors gone	(64, 65)
**20**	Sulfasalazine	NIH	SAM001246530	60S		
**21**	Epirubicin.HCl	NIH	SAM001246559	60S		

Table S3: Complete list of identified substances (continued)

*CAS-Nr. for the Enzo collection and the sample identifier for the NIH collection

**Complete list of all changing pre-rRNAs (± >1.5x) for substances listed in Table 1

***Documented activity against cancer cells. Only listed for substances showing the strongest rRNA processing defects (see Table 1)

No.	Substance	collection	ID*	Screen	rRNA pcocessing defects**	(reference)***
**22**	Itraconazole	NIH	SAM001246679	60S		
**23**	Clotrimazole	NIH	SAM001247056	60S		
**24**	Parecoxib.Na	NIH	SAM001246773	60S	35S ↑	(66, 67)
**25**	Artemether	NIH	SAM001246799	60S		
**26**	Bromocriptine mesylate	ENZO	22260-51-1	60S		
**27**	Cantharidin	ENZO	56-25-7	60S		
**28**	Chelerythrine	ENZO	3895-92-9	60S		
**29**	Daunorubicin	ENZO	23541-50-6	60S		
**30**	Doxorubicin	ENZO	25316-40-9	60S		
**31**	Caryophylline	ENZO	1139-30-6	60S		
**32**	Zerumbone	ENZO	471-05-6	60S	35S ↑	(68)
**33**	Valsartan	NIH	SAM001246581	40S	$27SA_2$ ↑	(69)
**34**	All trans retinoic acid	ENZO	302-79-4	40S	20S ↓	(70)
**35**	Visnagin	ENZO	82-57-5	40S	35S ↑	(71)
**36**	Rosiglitazone maleate	NIH	SAM001246610	40S		
**37**	Olanzapine	NIH	SAM001246652	40S		
**38**	Viramune (Nevirapine)	NIH	SAM001246551	40S		
**39**	Icariin	NIH	SAM001246560	40S		
**40**	Ipriflavone	NIH	SAM001246714	40S	27S and $27SA_2$ ↓	(72)
**41**	Sertraline	NIH	SAM001246666	40S		
**42**	Pantoprazole.Na	NIH	SAM001246591	40S		
**43**	Fluticasone Propionate	NIH	SAM001246583	40S		
**44**	Methyltestosterone	NIH	SAM001246682	40S		
**45**	Flubendazole	NIH	SAM001246685	40S	$27SA_2$ ↓	(73)
**46**	Nifekalant HCl	NIH	SAM001246717	40S		
**47**	Rofecoxib	NIH	SAM001246617	40S		
**48**	Famciclovir	NIH	SAM001246732	40S		

Table S3: Complete list of identified substances (continued)

*CAS-Nr. for the Enzo collection and the sample identifier for the NIH collection

**Complete list of all changing pre-rRNAs (± >1.5x) for substances listed in Table 1

***Documented activity against cancer cells. Only listed for substances showing the strongest rRNA processing defects (see Table 1)

No.	Substance	collection	ID*	Screen	rRNA pcocessing defects**	(reference)***
**49**	Megestrol acetate	NIH	SAM001246722	40S	20S and $27SA_2$ ↓	(74-76)
**50**	Trimebutine Maleate	NIH	SAM001246747	40S		
**51**	Mestanolone	NIH	SAM001246746	40S		
**52**	Zileuton	NIH	SAM001246738	40S	35S ↑	(77)
**53**	Fluphenazine.2HCl	NIH	SAM001246863	40S	7S and A_2-A_3 sp. ↑	(78, 79)
**54**	Cefaclor	NIH	SAM001246884	40S	7S ↑	not documented
**55**	Desoximetasone	NIH	SAM001246885	40S	7S ↑	not documented
**56**	Nicotinamide	NIH	SAM001246860	40S		not documented
**57**	Rimcazole	NIH	SAM001247094	40S	A_2-A_3 sp.	(80)
**58**	Itavastatin Ca	NIH	SAM001246803	40S		
**59**	Nialamide	NIH	SAM001246861	40S		
**60**	Donepezil	NIH	SAM001246627	40S		
**61**	GR 89696	NIH	SAM001247087	40S		
**62**	Irbesartan	NIH	SAM001246548	40S		
**63**	Levocetirizine	NIH	SAM001246575	40S	$27SA_2$ ↑	not documented
**64**	Pancuronium	NIH	SAM001247003	40S		
**65**	Cinanserin	NIH	SAM001246974	40S	$27SA_2$ ↑	not documented
**66**	Hexamethylenebisacetamide	NIH	SAM001247019	40S	35S ↑	not documented
**67**	Cisapride.H_2O	NIH	SAM001246975	40S		
**68**	Doxepin	NIH	SAM001246976	40S	$27SA_2$ ↑	not documented
**69**	Tacrine.HCl	NIH	SAM001247002	40S		
**70**	Indatraline.HCl	NIH	SAM001246981	40S	35S ↑	not documented
**71**	Cytarabine	NIH	SAM001247012	40S	35S and $27SA_2$ ↑	(81-83)

Table S3: Complete list of identified substances (continued)

*CAS-Nr. for the Enzo collection and the sample identifier for the NIH collection

**Complete list of all changing pre-rRNAs (± >1.5x) for substances listed in Table 1

***Documented activity against cancer cells. Only listed for substances showing the strongest rRNA processing defects (see Table 1)

No.	Substance	collection	ID*	Screen	rRNA pcocessing defects**	(reference)***
**72**	Naltrindole	NIH	SAM001247004	40S	35S ↑	(84, 85)
**73**	Methotrexate.H_2O	NIH	SAM001246985	40S		
**74**	Urapidil.HCl	NIH	SAM001247001	40S	35S ↑	not documented
**75**	Maprotiline.HCl	NIH	SAM001246989	40S		
**76**	Piribedil	NIH	SAM001246994	40S		
**77**	(-)-Cotinine	NIH	486-56-6	40S		
**78**	Pizotyline	NIH	SAM001247038	40S		
**79**	Pramipexole	NIH	SAM001247006	40S		
**80**	β-Estradiol	NIH	SAM001247032	40S		
**81**	DuP 697	NIH	SAM001247101	40S	35S ↑	(86)
**82**	Vindesine sulfate	NIH	SAM001246568	40S	35S ↑	(87-89)
**83**	Clobenpropit	NIH	SAM001247107	40S	35S ↑	(90)
**84**	Pergolide Mesylate	NIH	SAM001247070	40S	7S and 35S ↑	(91)
**85**	Ginkgolide B	ENZO	15291-77-7	40S		
**86**	Kainic acid	ENZO	487-79-6	40S		
**87**	(±)-Kavain	ENZO	500-64-1	40S		
**88**	3-Beta-Indoleacrylic acid	ENZO	1204-06-4	40S		
**89**	Parthenolide	ENZO	20554-84-1	40S		
**90**	Prostaglandin E1	ENZO	745-65-3	40S		
**91**	Radicicol	ENZO	12772-57-5	40S		
**92**	Rosmarinic acid	ENZO	20283-92-5	40S		
**93**	Rotenone	ENZO	83-79-4	40S	20S ↓	(92-94)
**94**	Thapsigargin	ENZO	67526-95-8	40S	A_2-A_3 sp.↑	(95)

Table S3: Complete list of identified substances (continued)

*CAS-Nr. for the Enzo collection and the sample identifier for the NIH collection

**Complete list of all changing pre-rRNAs (± >1.5x) for substances listed in Table 1

***Documented activity against cancer cells. Only listed for substances showing the strongest rRNA processing defects (see Table 1)

No.	Substance	collection	ID*	Screen	rRNA pcocessing defects**	(reference)***
**95**	Troleandomycin	ENZO	2751-09-9	40S	A_2-A_3 sp. ↑	not documented
**96**	Tunicamycin B	ENZO	11089-65-9	40S	7S ↓	(96)
**97**	Catalpol	ENZO	2415-24-9	40S	7S ↓ and 35S ↑	(97)
**98**	Veratramine	ENZO	60-70-8	40S	A_2-A_3 sp. and 35S ↑	(98)
**99**	Ivermectin	ENZO	70288-86-7	40S	35S ↑	(99)
**100**	(-)-Nicotine	ENZO	54-11-5	40S	A_2-A_3 sp. and 35S ↑	not documented
**101**	L-Penicillamine	ENZO	1113-41-3	40S	A_2-A_3 sp. ↑	(100-103)
**102**	Picrotoxinin	ENZO	17617-45-7	40S	A_2-A_3 sp. ↑	not documented
**103**	Tryptanthrin	ENZO	13220-57-0	40S	35S ↑	(104-106)
**104**	Yohimbine·HCl	ENZO	65-19-0	40S		
**105**	(-)-Eburnamonine	ENZO	4880-88-0	40S		
**106**	Celastrol	ENZO	34157-83-0	40S	35S ↑	(107)
**107**	Condelphine	ENZO	7633-69-4	40S		
**108**	Isorhoifolin	ENZO	552-57-8	40S	35S ↑	not documented
**109**	Picropodophyllin	ENZO	447-47-4	40S		
**110**	Narigenin-7-O-glucoside	ENZO	529-55-5	40S	35S ↑	not documented
**111**	Aloe-emodine	ENZO	481-72-1	40S		
**112**	Trans-4-Cotininecarboxylic acid	ENZO	33224-01-0	40S	$27SA_2$ ↑	not documented
**113**	Gitoxigenin	ENZO	545-26-6	40S	$27SA_2$ ↑	(108)
**114**	Harmane	ENZO	486-84-0	40S		
**115**	Leucomisine	ENZO	17946-87-1	40S	35S ↑	not documented
**116**	Tetrahydropapaverine·HCl	ENZO	6429-04-5	40S	35S ↑	not documented

Table S3: Complete list of identified substances (continued)

*CAS-Nr. for the Enzo collection and the sample identifier for the NIH collection

**Complete list of all changing pre-rRNAs (± >1.5x) for substances listed in Table 1

***Documented activity against cancer cells. Only listed for substances showing the strongest rRNA processing defects (see Table 1)

No.	Substance	collection	ID*	Screen	rRNA pcocessing defects**	(reference)***
**117**	Caffeine	ENZO	58-08-2	40S		
**118**	Lasalocid A	ENZO	25999-20-6	40S		
**119**	Cephadrine	ENZO	38821-53-3	40S		
**120**	Vasicine	ENZO	50591-64-5	40S		
**121**	Tetrahydrolipstatin (Orlistat)	ENZO	96829-58-2	40S	35S ↑	(109, 110)
**122**	Oleanolic acid	ENZO	508-02-1	40S		
**123**	Phlorizine	ENZO	60-81-1	40S	35S ↑	not documented
**124**	Diosmin	ENZO	520-27-4	40S	35S ↑	(111, 112)
**125**	Minocycline.HCl	ENZO	13614-98-7	40S		
**126**	16-Oxocafestol	ENZO	108664-98-8	40S		
**127**	Yangonin	ENZO	500-62-9	40S	23S ↑	(113)
**128**	Arbutin	ENZO	497-76-7	40S		
