# Supplementary material for: Inhibiting eukaryotic ribosome biogenesis
Source: BMC Biol. 2019 Jun 10;17:46. doi: 10.1186/s12915-019-0664-2 (PMC6558755; doi:10.1186/s12915-019-0664-2)
Supplement: Supplementary file 5 — Table S5. Saccharomyces cerevisiae strains used in this study. (PDF 244 kb) [file 12915_2019_664_MOESM5_ESM.pdf]

Table S5: ***Saccharomyces cerevisiae*** **strains**

Name	Genotype	Source
W303a	*ade2, his3, leu2, trp1, ura3, can1-100, MATa*	(148)
C303a	*ADE2, his3, leu2, trp1, ura3, can1-100, MATa*	this study
C303a Rpl7a-GFP	*RPL7A*-GFP::*HIS3*MX6, *ADE2, his3, leu2, trp1, ura3, can1-100, MATa*	this study
C303a Rpl7a-GFP Nic96-3x-mCherry	*RPL7A*-GFP::*HIS3*MX6, *ADE2, his3, leu2, trp1, ura3, can1-100, MATa NIC96-3xmCherry::hphNT1*	this study
C303a Rpl7a-GFP Nop58-3x-mCherry	*RPL7A*-GFP::*HIS3*MX6, *ADE2, his3, leu2, trp1, ura3, can1-100, MATa NOP58-3xmCherry::hphNT1*	this study
C303a Rpl7a-GFP Hho1-3x-mCherry	*RPL7A*-GFP::*HIS3*MX6, *ADE2, his3, leu2, trp1, ura3, can1-100, MATa HHO1-3xmCherry::hphNT1*	this study
C303a Rps9a-GFP	*RPS9A*-GFP::*HIS3*MX6, *ADE2, his3, leu2, trp1, ura3, can1-100, MATa*	this study
C303a Rps9a-GFP Nic96-3x-mCherry	*RPS9A*-GFP::*HIS3*MX6, *ADE2, his3, leu2, trp1, ura3, can1-100, MATa NIC96-3xmCherry::hphNT1*	this study
C303a Rps9a-GFP Nop58-3x-mCherry	*RPS9A*-GFP::*HIS3*MX6, *ADE2, his3, leu2, trp1, ura3, can1-100, MATa NOP58-3xmCherry::hphNT1*	this study
BY4741	*MATa; ura3Δ0; leu2Δ0; his3Δ1; met15Δ0*	Euroscarf
BY4741 Δ*rrp6*	*Mat alpha; his3Δ1; leu2Δ0; lys2Δ0; ura3Δ0; YOR001w::kanMX4*	Euroscarf
BY4743	*Mata/alpha; his3Δ1/his3Δ1; leu2Δ0/leu2Δ0; met15Δ0/MET15; LYS2/lys2Δ0; ura3Δ0/ ura3Δ0*	Euroscarf
BY4743 Δ*rrp43/RRP43*	*BY4743; MATa/MATα; ura3Δ0/ura3Δ0; leu2Δ0/leu2Δ0; his3Δ1/his3Δ1; met15Δ0/MET15; LYS2/lys2Δ0; YCR035c/YCR035c::kanMX4*	Euroscarf
BY4743 Δ*rrp42/RRP42*	*BY4743; MATa/MATα; ura3Δ0/ura3Δ0; leu2Δ0/leu2Δ0; his3Δ1/his3Δ1; met15Δ0/MET15; LYS2/lys2Δ0; YDL111c/YDL111c::kanMX4*	Euroscarf
BY4743 Δ*rrp41/RRP41*	*BY4743; MATa/MATα; ura3Δ0/ura3Δ0; leu2Δ0/leu2Δ0; his3Δ1/his3Δ1; met15Δ0/MET15; LYS2/lys2Δ0; YGR195w/YGR195w::kanMX4*	Euroscarf
BY4743 Δ*mtr4/MTR4*	*BY4743; MATa/MATα; ura3Δ0/ura3Δ0; leu2Δ0/leu2Δ0; his3Δ1/his3Δ1; met15Δ0/MET15; LYS2/lys2Δ0; YJL050w/YJL050w::kanMX4*	Euroscarf
BY4743 Δ*nop53/NOP53*	*BY4743; MATa/MATα; ura3Δ0/ura3Δ0; leu2Δ0/leu2Δ0; his3Δ1/his3Δ1; met15Δ0/MET15; LYS2/lys2Δ0; YPL146c/YPL146c::kanMX4*	Euroscarf
